# Oxygenated Cyclopentenones via the Pauson–Khand Reaction of Silyl Enol Ether Substrates

**DOI:** 10.1021/acs.orglett.2c00856

**Published:** 2022-04-04

**Authors:** Paul Shaw, Storm J. Hassell-Hart, Gayle E. Douglas, Andrew G. Malcolm, Alan R. Kennedy, Gemma V. White, Laura C. Paterson, William J. Kerr

**Affiliations:** † Department of Pure and Applied Chemistry, University of Strathclyde, 295 Cathedral Street, Glasgow G1 1XL, Scotland, U.K.; ‡ Medicines Research Centre, GlaxoSmithKline, Gunnels Wood Road, Stevenage, Hertfordshire SG1 2NY, England, U.K.

## Abstract

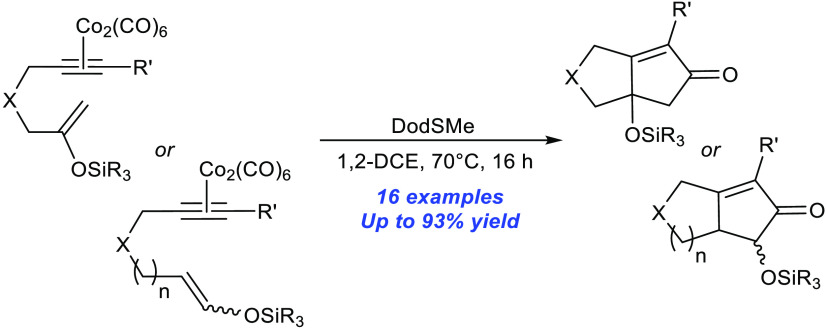

We report here the application of silyl enol ether moieties as efficient alkene coupling partners within cobalt-mediated intramolecular Pauson–Khand reactions. This cyclization strategy delivers synthetically valuable oxygenated cyclopentenone products in yields of ≤93% from both ketone- and aldehyde-derived silyl enol ethers, incorporates both terminal and internal alkyne partners, and delivers a variety of decorated systems, including more complex tricyclic structures. Facile removal of the silyl protecting group reveals oxygenated sites for potential further elaboration.

The preparation of suitably functionalized polycyclic systems in a direct and efficient manner remains a widely explored area within organic synthesis. Most commonly, metal-mediated transformations are being applied to access increasingly more diverse and desirable structural frameworks in a preparatively concise fashion. In this regard, a key method for constructing molecular complexity in a single transformation is the Pauson–Khand reaction (PKR) (Scheme 1A).^1^ Traditionally mediated by cobalt, the PKR brings an alkene, alkyne (present as its dicobalthexacarbonyl complex), and a carbon monoxide moiety together to construct a five-membered cyclopentenone ring. Since its discovery, this organocobalt cyclization process has been developed into an effective synthetic method, which has found increasing use as the key transformation in the synthesis of natural products and other cyclic compounds possessing varied skeletal frameworks.^2^ Having stated this, we acknowledge that the substrate scope remains somewhat limited, with bicyclic motifs derived from unelaborated alkyne and alkene components being most readily prepared. More specifically and with specific regard to the alkene component of this cycloaddition reaction, there are limited examples of more functionalized partners, such as those containing additional heteroatoms, which would provide more diverse cyclopentenone products with potentially useful functionality.^3^ Indeed, this specific limitation in the current Pauson–Khand methodology was highlighted in a recent publication by Micalizio and co-workers in which they described an elegant method, which is complementary to the PKR, for providing access to more heavily substituted and oxygenated cyclopentenone products.^4^ As part of our own continuing efforts to further develop the effectiveness of the PKR toward delivering a wide range of desirable and elaborated chemical scaffolds,^5^ we sought to probe alternative functionalized alkene components to the more standard and typically employed olefin substrates. While we have previously described the use of vinyl ethers and esters as alkene components in the intermolecular PKR, the former reacted only very inefficiently under the methods described,^5h^ and the latter resulted in cleavage of the oxygenated functionality under the reaction conditions, ultimately providing cyclopentenone products whereby the vinyl ester alkene partner had acted as an ethylene equivalent.^5h,5i^

**Scheme 1:**
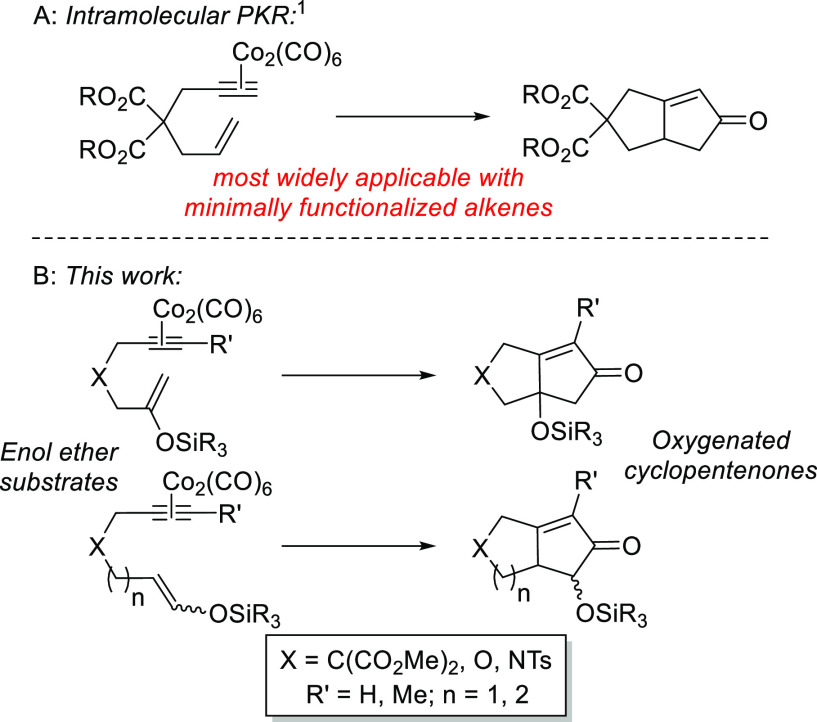
Pauson–Khand Reaction

Herein, we report the first use of silyl enol ether substrates as alkene partners within the Pauson–Khand annulation process (Scheme 1B).^6^ This protocol allows the retention of the heteroatom functionality and represents a general and practically efficient transformation that can deliver a range of desirable cyclized scaffolds notably possessing oxygenated sites. Depending on the nature of the starting substrate, this process allows the construction of synthetically demanding C–O quaternary carbon centers and desirable α-oxygenated cyclopentenone frameworks.

Naturally, we envisaged that each required silyl enol ether substrate would be prepared from the corresponding ketone or aldehyde. To initiate these studies, ketone **1** was prepared via a short synthetic sequence^7^ and reacted with diisopropylethylamine (DIPEA) and *tert-*butyldimethylsilyl triflate (TBSOTf) to generate the corresponding silyl enol ether **2** exclusively and in good yield (Scheme 2). Subsequently, and to provide the starting substrate for the key PKR, **2** was reacted with Co_2_(CO)_8_ to deliver the requisite dicobalthexacarbonyl complex **3** in 98% isolated yield.

**Scheme 2:**
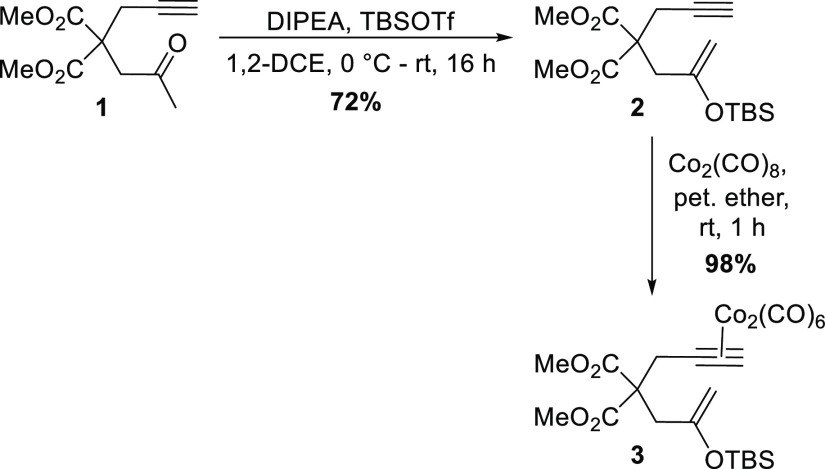
Preparation PKR Precursor **3**

With the requisite substrate in hand, the use of such a silyl enol ether in the Pauson–Khand annulation process was explored (Table 1). Our initial conditions used the common PKR promoter, trimethylamine *N*-oxide dihydrate (TMANO·2H_2_O),^8^ which, pleasingly, afforded the desired oxygenated cyclopentenone **4** in 31% yield after 16 h at room temperature (Table 1, entry 1). Following this proof-of-concept result, our attention turned to improving the effectiveness of the cyclization and, to this end, dodecylmethyl sulfide (DodSMe) was employed as an additive; we have shown previously that this inexpensive and non-noxious promotor works extremely well for more standard PKRs.^5f^ Gratifyingly, the use of DodSMe (at levels marginally above those employed in previous studies in our laboratory^5f^) delivered **4** in a much improved 78% yield after 16 h, under the refluxing conditions in 1,2-DCE frequently required with sulfide promotion (entry 2).^5f,9^ Monitoring these conditions more closely showed that the reaction was, indeed, relatively efficient, delivering the desired product in only 2 h (entry 3). Further improvement was noted when the temperature was decreased slightly to 70 °C, whereby the desired oxygenated cyclopentenone was isolated in a very good 88% yield (entry 4). Additional experiments considered the use of cyclohexylamine^10^ (entry 5) and tetramethylthiourea (TMTU)^11^ (entry 6) as alternative additives; however, these did not match the effectiveness of the sulfide-promoted system. Notably, significant amounts of decomplexed starting material were recovered on these occasions (23% and 29%, respectively). A final experiment tested simple heating, in the absence of an additive, but with no beneficial effect (entry 7).

**Table 1:**
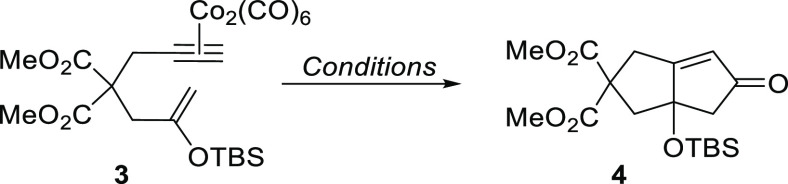
Optimization of PKR Conditions with a Silyl Enol Ether

entry	conditions	yield (%)^a^
1	TMANO·2H_2_O (6.8 equiv), 1,2-DCE, rt, 16 h	31
2	DodSMe (4.75 equiv), 1,2-DCE, reflux, 16 h	78
3	DodSMe (4.75 equiv), 1,2-DCE, reflux, 2 h	63
4	DodSMe (4.75 equiv), 1,2-DCE, 70 °C, 16 h	88
5	CyNH_2_ (3.5 equiv), 1,2-DCE, 70 °C, 16 h	2
6	TMTU (4.75 equiv), 1,2-DCE, 70 °C, 16 h	21
7	no additive, 1,2-DCE, 70 °C, 16 h	18

a Isolated yields.

Despite the successful application of enol ether substrate **3**, we were also keen to explore the generality of the process with respect to the silyl moiety to gauge the tolerance for these groups within our emerging system. To this end, trimethylsilyl (TMS), triethylsilyl (TES), and triisopropylsilyl (TIPS) enol ether substrates of type **5** were considered. While we were unable to access the TMS enol ether due to its instability upon isolation, the TES and TIPS derivatives were prepared in good to excellent yields from our starting ketone **1** (Table 2). Subsequent complexation of compounds **5a** and **5b** provided the corresponding dicobalthexacarbonyl complexes **6** in 85% and 64% yields, respectively. At this stage, employing our developed PKR protocol, we were pleased to find that TES derivative **6a** performed well, and in line with the previously used TBS analogue, delivering the desired framework in an appreciable 77% yield. Unfortunately, TIPS compound **6b** did not perform as desired; there was evidence of decomposition of the starting material, and none of the desired oxygenated cyclopentenone product was identified after 16 h at 70 °C. This limitation is likely due to the increased steric demand of the larger isopropyl units.

**Table 2:**
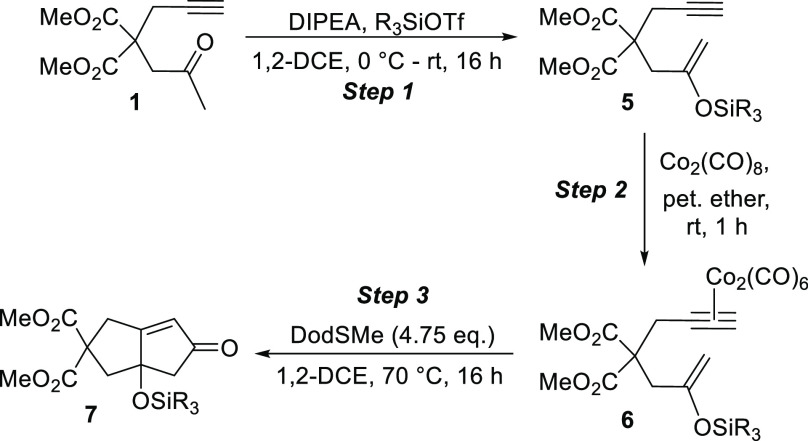
Investigation of the Silyl Moiety

entry	R	yield of step 1 (%)	yield of step 2 (%)	yield of step 3 (%)
1	Et	90 (**5a**)	85 (**6a**)	77 (**7a**)
2	^i^Pr	69 (**5b**)	64 (**6b**)	0 (**7b**)

With this knowledge, we embarked upon the application of our developed protocol to the intramolecular cyclization of a range of ketone-derived silyl enol ether substrates. The requisite cobalt complexes **8a–j** were synthesized efficiently^7^ and, as shown in Scheme 3, the developed PKR method allowed the construction of a range of bi- and tricyclic systems in good to excellent yields. This establishes a practically accessible cyclization protocol of good synthetic potential, notably providing the ability to directly access this class of structures, possessing such a challenging quaternary oxygenated center. More specifically, in addition to terminal substrates, this approach allows the very effective application of cyclization precursors containing internal alkynes, as shown by oxygenated cyclopentenones **9c–h**; such substrates are typically more challenging in the PKR domain.^1^ Furthermore, tricyclic cyclopentenones **9i** and **9j** were also accessed through the developed method; **9i** was furnished with excellent efficiency for such a conformationally rigid structure, and **9j** representing the central core of (−)-presilphiperfolan-1-ol, an intriguing tricyclo[5.3.1.0^4,11^]undecane sesquiterpene.^12^

**Scheme 3:**
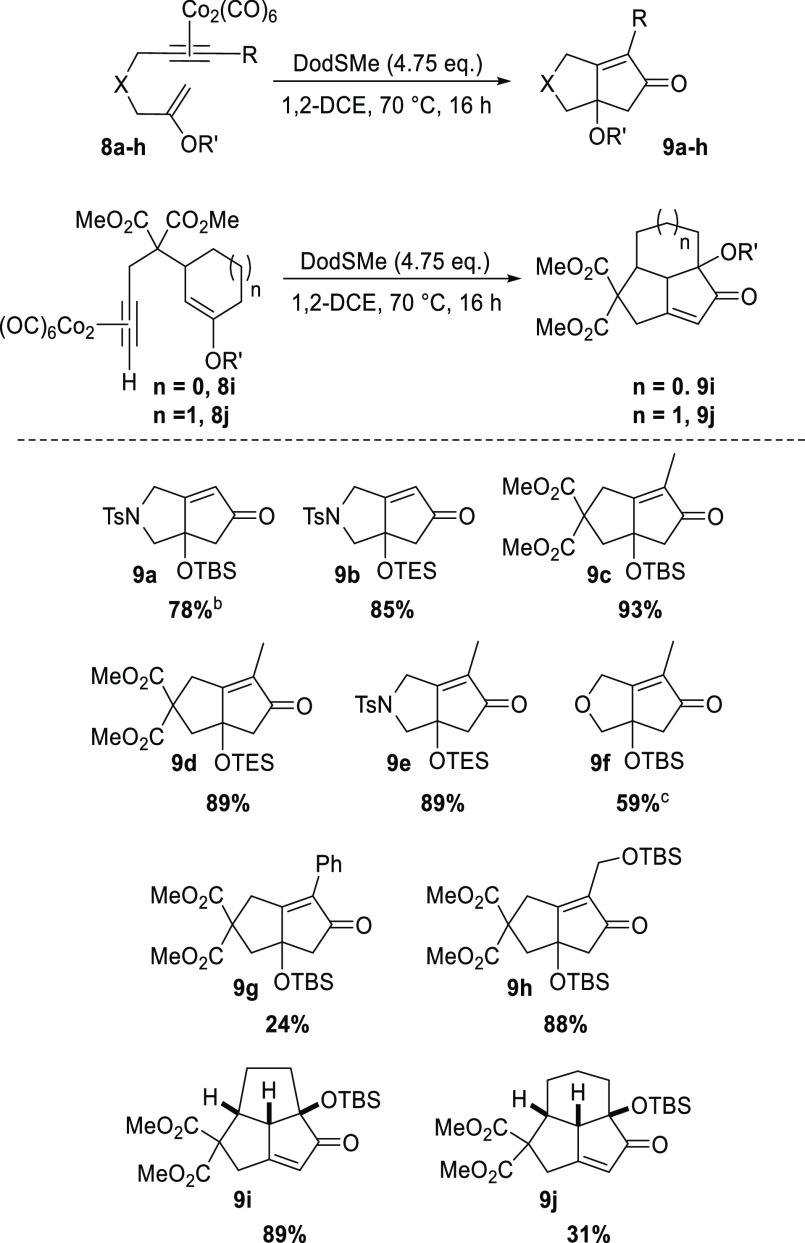
Substrate Scope with Ketone-Derived Silyl Enol Ethers in the Intramolecular PKR Isolated yields. Reaction time of 2 h. Reaction time of 48 h.

In light of the positive preparative outputs to this stage, we next explored the capacity of the developed protocol to accommodate silyl enol ether substrates derived from aldehydes, which, in turn, would produce α-oxygenated cyclopentenone products (Scheme 4). Such compounds, or simple derivatives thereof, are important structural motifs in many areas of chemistry and biology and are particularly prevalent in biologically active natural products and pharmaceuticals.^13^ In this regard, starting complexes of type **10** could be readily accessed via short individual synthetic sequences.^7^ Subsequent exposure to our identified PKR protocol gratifyingly delivered the desired bicyclic products with good levels of effectiveness. In particular, high yields of compounds **11a** and **11b** were achieved (88% and 86%, respectively), with N-linked derivative **11c** obtained in a more moderate yield of 66%. In addition to these examples, the 6,5-fused oxygenated cyclopentenone structure **11d** was accessed in 55% yield, albeit employing a prolonged reaction time of 24 h. More generally, this latter structural class arises from a more challenging cyclization based on standard PKR methodology.^1^ It should also be noted that the *anti*:*syn* ratio of each α-oxygenated cyclopentenone product did not always correlate closely with the *E*:*Z* ratio presented in the starting silyl enol ether compound, with varying degrees of epimerization having resulted across the products obtained.^7^

**Scheme 4:**
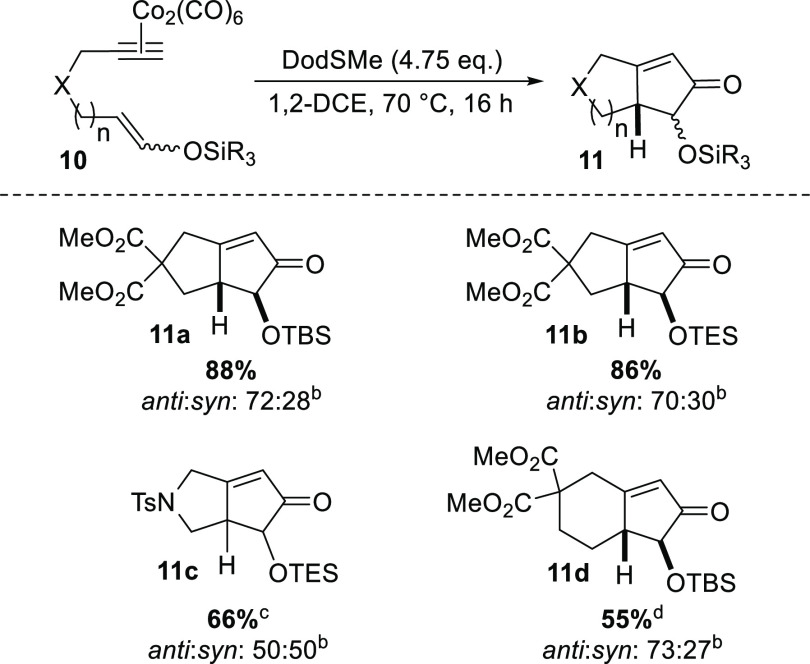
Substrate Scope Using Aldehyde-Derived Silyl Enol Ethers in the Intramolecular PKR Isolated yields. Determined by ^1^H NMR analysis. Reaction time of 3 h. Reaction time of 24 h.

In an attempt to deliver an even more practically accessible method toward such oxygenated cyclopentenone scaffolds, we have demonstrated that the dicobalthexacarbonyl complex can be generated and subsequently cyclized via a one-pot process. As shown in Scheme 5, silyl enol ether **2** performed extremely well as part of this protocol, delivering the desired, and suitably functionalized, bicyclic enone in an excellent 93% yield. We believe that this marginal increase in yield over that realized via the use of the preformed and isolated Co_2_(CO)_6_-alkyne substrate results from the avoidance of any slow decomposition arising from exposure of this complex during mechanical transfer.

**Scheme 5:**
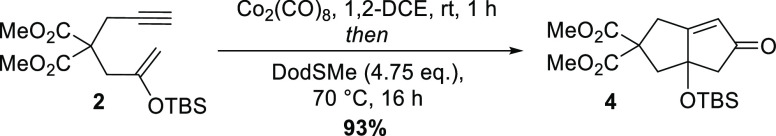
One-Pot Complexation/Pauson–Khand Reaction

Having established the PKR protocol described with the scope to deliver a range of bi- and tricyclic oxygenated cyclopentenone systems, we attempted the deprotection of a selection of the prepared silyl ethers to reveal the corresponding free hydroxy products (Scheme 6). In this regard, specifically tuned acidic conditions afforded the desired deprotected products in, generally, good to moderate yields. To deliver compounds **12a–e**, the protecting group was removed from the quaternary center with relative ease, with the exception of compound **12e**, for which a prolonged reaction time and moderately increased temperature were required. α-Hydroxycyclopentenones **13a** and **13b** were also accessed albeit in lower yields of 34% and 20%, respectively, and isolated as single diastereomers.^14^

**Scheme 6:**
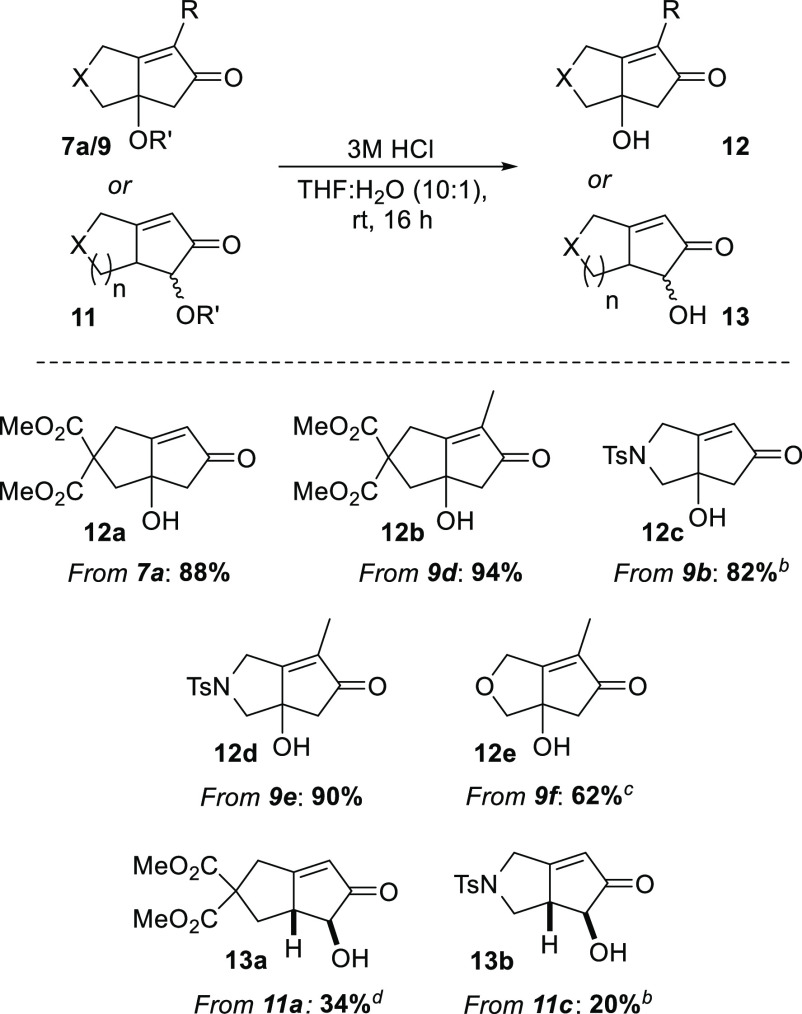
Cyclopentenone Silyl Ether Deprotection Isolated yields. Reaction time of 5 h. Reaction temperature of 40 °C and reaction time of 100 h. Reaction time of 18 h.

In summary, we have established the first examples of efficient PKRs incorporating silyl enol ether moieties as the alkene component in this annulation process. This development has widened the scope and utility of the PKR, providing access to a range of oxygenated cyclopentenone units through an accessible and effective cyclization protocol; such product structures have been deemed previously unattainable via existing Pauson–Khand methodology. Notably, silyl enol ethers derived from ketones and aldehydes have both been applied effectively, and deprotection of a selection of the resultant compounds has been successful in affording various cyclopentenones possessing an oxygen-containing functionality of potential further preparative and biological interest.

## Supporting Information Available

Detailed synthetic procedures, compound characterization, and full X-ray crystallography data for compounds **13a** and **13b** (PDF)

## Accession Codes

CCDC 2105307–2105308 contain the supplementary crystallographic data for this paper. These data can be obtained free of charge via www.ccdc.cam.ac.uk/data_request/cif, or by emailing data_request@ccdc.cam.ac.uk, or by contacting The Cambridge Crystallographic Data Centre, 12 Union Road, Cambridge CB2 1EZ, UK; fax: +44 1223 336033.
