# Supplementary material for: Oxygenated Cyclopentenones via the Pauson–Khand Reaction of Silyl Enol Ether Substrates
Source: Org Lett. 2022 Apr 4;24(14):2750–5. doi: 10.1021/acs.orglett.2c00856 (PMC9016766; doi:10.1021/acs.orglett.2c00856)
Supplement: Supplementary file 1 — ol2c00856_si_001.pdf [file ol2c00856_si_001.pdf]

Supporting Information for

Oxygenated Cyclopentenones *via* the Pauson-Khand Reaction of Silyl Enol Ether Substrates

Paul Shaw,[a] Storm J. Hassell-Hart,[a,b] Gayle E. Douglas,[a] Andrew G. Malcolm,[a] Alan R. Kennedy,[a] Gemma V. White,[b] Laura C. Paterson,[a] and William J. Kerr[a,*]

[a]Department of Pure and Applied Chemistry, University of Strathclyde, 295 Cathedral Street, Glasgow G1 1XL, Scotland, U.K.

[b]GlaxoSmithKline, Medicines Research Centre, Gunnels Wood Road, Stevenage, Hertfordshire SG1 2NY, England, U.K.

*Corresponding Author: E-mail: w.kerr@strath.ac.uk

Contents

**I**	**General**	**S2**
**II**	**Reagent and substrate synthesis**	**S3**
	**a. Carbonyl compounds**	**S3**
	**b. Silyl enol ether substrates**	**S17**
	**c. Dicobalthexacarbonyl complexes**	**S28**
**III**	**Pauson-Khand reactions**	**S38**
**IV**	**Deprotection of silyl-protected cyclopentenones**	**S49**
**V**	**X-Ray crystallography data for 13a and 13b**	**S53**
**VI**	**^{1}H and ^{13}C NMR spectra for all novel compounds**	**S55**
**VII**	**References**	**S127**

I General

All reagents were obtained from commercial suppliers and used without further purification, unless otherwise stated. All reactions were carried out under an inert, dry argon atmosphere, unless otherwise stated. Purification was carried out according to standard laboratory methods.[1]

For reactions that require heating, the stated temperature was reached using an oil bath and temperature probe.

Dry DCM, Et_2O, THF, and toluene were obtained from an Innovative Technology, Pure Solv, SPS-400-5 solvent purification system. All other solvents were used as purchased unless required dry, wherein distillation under argon, and over calcium hydride, was performed prior to use.

Petroleum ether refers to petroleum ether in the boiling point (b.p.) range 40- 60 °C unless otherwise stated. DCE refers to 1,2-dichloroethane. NaH refers to sodium hydride 60% dispersion in mineral oil unless otherwise stated. Propargyl bromide refers to an 80% by weight solution of propargyl bromide in toluene. Di-*iso*propylamine and di-*iso*propylethylamine were distilled under argon, and over calcium hydride, and stored over barium oxide.

Thin layer chromatography was carried out using Camlab silica plates coated with fluorescent indicator UV254. Plates were analysed using a Mineralight UVGL-25, lamp or developed using a vanillin solution.

Flash column chromatography was carried out using Prolabo silica gel (230-400 mesh).

Melting points were obtained (uncorrected) on a Gallenkamp Griffin melting point apparatus.

IR spectra were obtained on a Shimadzu IRAffinity-1 machine.

^{1}H and ^{13}C NMR spectra were obtained on a Bruker DPX 400 spectrometer at 400 MHz and 101 MHz, respectively, or a Bruker AVANCE DRX 500 spectrometer at 500 MHz and 125 MHz, respectively, as indicated. Chemical shifts are reported in ppm, and coupling constants are reported in Hz and refer to $^{3}J_{H\text{-}H}$ interactions, unless otherwise specified.

High resolution mass spectra were recorded on a Thermo Scientific LTQ Orbitrap XL instrument at the EPSRC Mass Spectrometry facility at the University of Wales, Swansea using the electrospray ionisation (ESI), nanospray ionisation (NSI) or atmospheric-pressure chemical ionisation (APCI) techniques.

Details related to X-ray crystallography are discussed on page S53.

II Reagent and substrate synthesis

a. Carbonyl compounds

Silyl enol ether substrates were prepared from their corresponding ketone or aldehyde compounds. The following section details the short synthetic sequences towards each ketone or aldehyde intermediate. Full experimental details and characterisation data are provided for all intermediate compounds.

Towards dimethyl 2-(2-oxopropyl)-2-(prop-2-yn-1-yl)malonate 1 and dimethyl 2-(but-2-yn-1-yl)-2-(2-oxopropyl)malonate S2.

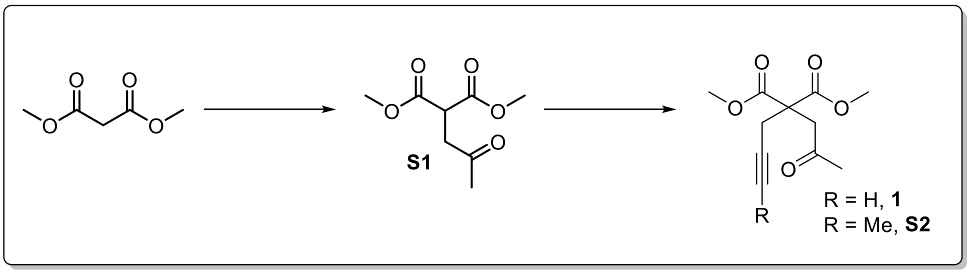

Preparation of dimethyl 2-(2-oxopropyl)malonate.[2]

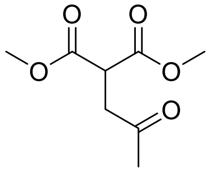

S1

NaH (1.17 g, 31.9 mmol) and distilled DMF (30 mL) were added to a flame-dried, round-bottom flask equipped with a stirrer bar. The solution was cooled to 0 °C and dimethylmalonate (3.05 mL, 26.6 mmol) was added dropwise. The resulting mixture was stirred at 0 °C for 1 h. At this point, the reaction mixture was warmed to room temperature and chloroacetone (2.55 mL, 31.9 mmol) was added dropwise. The reaction mixture was stirred for 16 h and then quenched by the addition of distilled water (30 mL). Following this, Et_2O (30 mL) was added to create a biphasic mixture, which was separated, and the aqueous layer washed with Et_2O (3 × 30 mL). The combined organic extracts were washed with brine (30 mL), dried over Na_2SO_4, filtered, and concentrated *in vacuo* to provide the crude product as a yellow oil. The crude material was purified by flash column chromatography (pet. ether:Et_2O, 70:30) and concentrated *in vacuo* to provide dimethyl 2-(2-oxopropyl)malonate **S1** (2.52 g, 13.37 mmol, **50%**) as a colourless oil.

^{1}H NMR ($CDCl_3$, 400 MHz): δ_H 3.91 (1H, t, *J* = 7.1 Hz), 3.77 (6H, s), 3.09 (2H, d, *J* = 7.2 Hz), 2.22 (3H, s). **^{13}C NMR ($CDCl_3$, 101 MHz)**: δ_C 204.3, 168.7, 52.2, 46.0, 41.5, 29.1. **IR (v_{max}/cm^{-1})**: 2957, 1732, 1716, 1156.

Preparation of dimethyl 2-(2-oxopropyl)-2-(prop-2-yn-1-yl)malonate.[3]

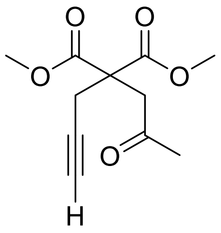

1

NaH (95% dispersion in mineral oil, 0.16 g, 6.12 mmol) and distilled DMF (20 mL) were added to a flame-dried, round-bottom flask equipped with a stirrer bar. The solution was cooled to 0 °C and dimethyl 2-(2-oxopropyl)malonate **S1** (1.05 g, 5.57 mmol) was added dropwise. The resulting mixture was stirred at 0 °C for 1 h. At this point, the reaction mixture was warmed to room temperature and propargyl bromide (0.75 mL, 6.68 mmol) was added dropwise. The reaction mixture was stirred for 16 h and then quenched by the addition of 2M HCl (20 mL). Following this, Et_2O (20 mL) was added to create a biphasic mixture. The organic phase was separated and the aqueous phase washed with Et_2O (3 × 20 mL). The combined organic extracts were washed with brine (15 mL), dried over Na_2SO_4, filtered, and concentrated *in vacuo* to provide the crude product as an orange oil. The crude material was purified by flash column chromatography (pet. ether:Et_2O, 70:30) and concentrated *in vacuo* to provide dimethyl 2-(2-oxopropyl)-2-(prop-2-yn-1-yl)malonate **1** (1.17 g, 5.16 mmol, **93%**) as a colourless oil.

^{1}H NMR ($CDCl_3$, 400 MHz): δ_H 3.72 (6H, s), 3.33 (2H, s), 2.98 (2H, d, ^4J = 2.7 Hz), 2.17 (3H, s), 2.02 (1H, t, ^4J = 2.7). **^{13}C NMR ($CDCl_3$, 101 MHz)**: δ_C 204.9, 169.1, 79.2, 71.2, 53.8, 52.6, 44.9, 29.7, 22.8. **IR (ν_{max}/cm^{-1})**: 3278, 2954, 1738, 1718, 1201.

Preparation of dimethyl 2-(but-2-yn-1-yl)-2-(2-oxopropyl)malonate.[4]

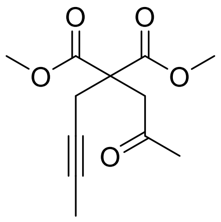

S2

NaH (0.29 g, 7.34 mmol) and distilled DMF (15 mL) were added to a flame-dried, round-bottom flask equipped with a stirrer bar. The solution was cooled to 0 °C and dimethyl 2-(2-oxopropyl)malonate **S1** (1.26 g, 6.67 mmol) was added dropwise. The resulting mixture was stirred at 0 °C for 1 h. At this point, the reaction mixture was warmed to room temperature and 1-bromobut-2-yne (0.7 mL, 8.00 mmol) was added dropwise. The reaction mixture was stirred for 16 h and then quenched by the addition of 2M HCl (20 mL). Following this, Et_2O (20 mL) was added to create a biphasic mixture. The organic phase was separated and the aqueous phase washed with Et_2O (3 × 20 mL). The combined organic extracts were washed with brine (15 mL), dried over Na_2SO_4, filtered, and concentrated *in vacuo* to provide the crude product as an orange oil. The crude material was purified by flash column chromatography (pet. ether:Et_2O, 70:30) and concentrated *in vacuo* to provide dimethyl 2-(but-2-yn-1-yl)-2-(2-oxopropyl)malonate **S2** (1.05 g, 4.35 mmol, **65%**) as a white solid.

^{1}H NMR (CDCl$_3$, 400 MHz): δ$_H$ 3.74 (6H, s), 3.34 (2H, s), 2.94 (2H, q, ^5J = 2.6 Hz), 2.20 (3H, s), 1.77 (3H, t, ^5J = 2.6 Hz). **^{13}C NMR (CDCl$_3$, 101 MHz)**: δ$_C$ 204.9, 169.4, 78.7, 73.1, 54.3, 52.6, 45.2, 29.8, 23.3, 3.0. **IR (ν$_{max}$/cm^{-1})**: 3278, 2954, 1738, 1718, 1201. **Melting point**: 67 - 69 °C.

Towards *N*-(2-oxopropyl)-*N*-(prop-2-yn-1-yl)toluenesulfonamide S6 and *N*-(but-2-yn-1-yl)-*N*-(2-oxopropyl)toluenesulfonamide S7.

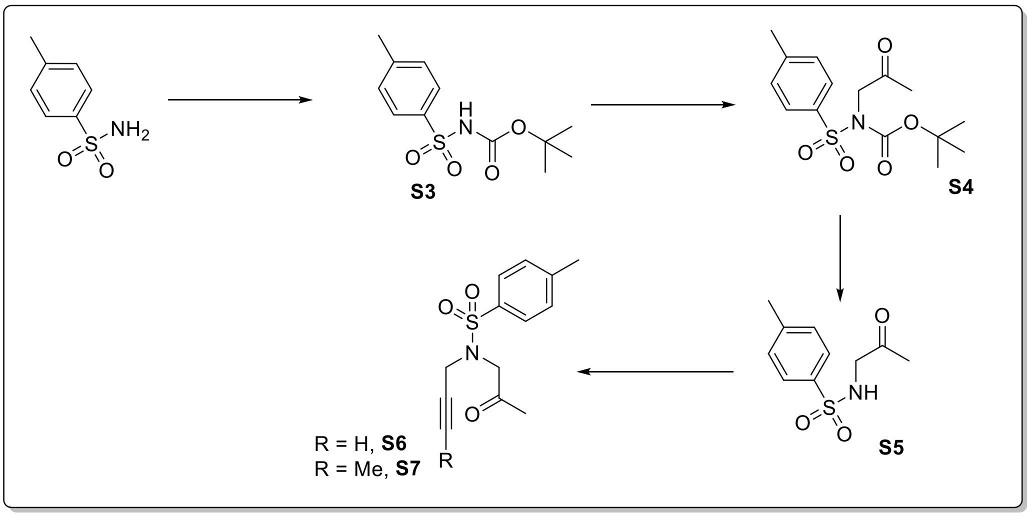

Preparation of *tert*-butyl tosylcarbamate.[5]

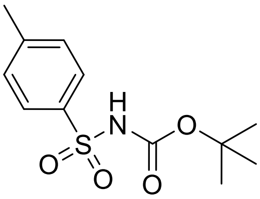

S3

p-Toluenesulfonamide (5 g, 29.2 mmol), Et$_3$N (4.5 mL, 32.1 mmol), DMAP (0.36 g, 2.92 mmol), and distilled DCM (25 mL) were added to a flame-dried, round-bottom flask equipped with a stirrer bar. Di-*tert*-butyl dicarbonate (7.5 mL, 32.1 mmol) was added to this solution and the resulting mixture was stirred at room temperature for 14 h. After this time, the mixture was concentrated *in vacuo* and the residue was dissolved in EtOAc (50 mL). 1M HCl (50 mL) was added to create a biphasic mixture, which was separated, and the organic phase washed with distilled water (50 mL) and brine (50 mL). The organic phase was dried over Na$_2$SO$_4$, filtered, and concentrated *in vacuo* to provide the crude product as a colourless oil. The crude was dissolved in a small amount of Et$_2$O and triturated with pet. ether to give *tert*-butyl tosylcarbamate **S3** as a white crystalline solid (7.35 g, 27.08 mmol, **93%**).

^{1}H NMR (CDCl$_3$, 400 MHz): δ$_H$ 7.92 – 7.88 (2H, m), 7.36 – 7.31 (2H, m), 7.12 (1H, s), 2.48 (3H, s), 1.41 (9H, s). **^{13}C NMR (CDCl$_3$, 101 MHz)**: δ$_C$ 148.6, 144.2, 135.5, 129.0, 127.7, 83.6, 27.4, 21.2. **IR (ν$_{max}$/cm^{-1})**: 3212, 2979, 1750, 1436, 1152. **Melting point:** 117 – 119 °C.

Preparation of *tert*-butyl (2-oxopropyl)(tosyl)carbamate.[6]

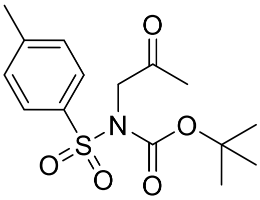

S4

Tert-butyl tosylcarbamate **S3** (1.5 g, 5.53 mmol) and distilled DMF (20 mL) were added to a flame-dried, round-bottom flask equipped with a stirrer bar. The mixture was cooled to 0 °C, NaH (0.23 g, 5.81 mmol) was added, and the resulting mixture was stirred for 1 h. At this point, the reaction mixture was warmed to room temperature and chloroacetone (0.48 mL, 6.08 mmol) was added rapidly. The reaction mixture was stirred for 16 h at room temperature then quenched by the addition of brine (25 mL). Following this, Et_2O (25 mL) was added to create a biphasic mixture, which was separated and the aqueous layer washed with Et_2O (3 × 25 mL). The combined organic extracts were dried over Na_2SO_4, filtered and concentrated *in vacuo* to provide the crude product as a yellow oil. The crude material was purified by flash column chromatography (pet. ether:Et_2O, 50:50) and concentrated *in vacuo* to provide *tert*-butyl (2-oxopropyl)(tosyl)carbamate **S4** as a white solid (0.80 g, 2.43 mmol, **44%**).

^{1}H NMR ($CDCl_3$, 400 MHz): δ_H 7.96 – 7.91 (2H, m), 7.36 – 7.31 (2H, m), 4.64 (2H, s), 2.46 (3H, s), 2.24 (3H, s), 1.32 (9H, s). **^{13}C NMR ($CDCl_3$, 101 MHz)**: δ_C 201.0, 150.0, 144.0, 136.2, 128.7, 128.1, 84.4, 54.1, 27.3, 26.2, 21.2. **IR (ν_{max}/cm^{-1})**: 3277, 1738, 1719, 1600, 1349, 1149. **Melting point**: 72 – 74 °C.

Preparation of *N*-(2-oxopropyl)toluenesulfonamide.[6]

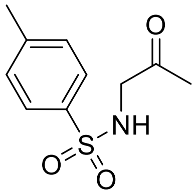

S5

Tert-butyl (2-oxopropyl)(tosyl)carbamate **S4** (0.74 g, 2.25 mmol) and distilled DCM (10 mL) were added to a flame-dried, round-bottom flask equipped with a stirrer bar. Trifluoroacetic acid (0.52 mL, 6.75 mmol) was added to this solution and the resulting mixture was stirred at room temperature for 16 h. At this point, the solvent was removed *in vacuo* and toluene added to the residue. This was concentrated *in vacuo* once more to give *N*-(2-oxopropyl)toluenesulfonamide **S5** (0.4 g, 1.76 mmol, **78%**) as a white powder.

^{1}H NMR ($CDCl_3$, 400 MHz): δ_H 7.78 – 7.73 (2H, m), 7.36 – 7.30 (2H, m), 5.31 (1H, t, *J* = 4.4 Hz), 3.87 (2H, d, *J* = 4.7 Hz), 2.44 (3H, s), 2.13 (3H, s). **^{13}C NMR ($CDCl_3$, 101 MHz)**: δ_C 200.5, 143.3, 135.6, 129.3, 126.7, 51.6, 26.6, 21.0. **IR (ν_{max}/cm^{-1})**: 3277, 1716, 1600, 1325, 1160. **Melting point**: 94 – 96 °C.

Preparation of *N*-(2-oxopropyl)-*N*-(prop-2-yn-1-yl)toluenesulfonamide.[7]

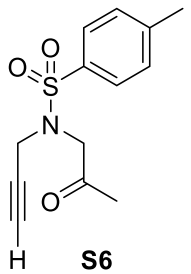

S6

N-(2-Oxopropyl)toluenesulfonamide **S5** (0.15 g, 0.66 mmol), K_2CO_3 (0.1 g, 0.73 mmol), and DMF (10 mL) were added to a flame-dried, round-bottom flask equipped with a stirrer bar. The suspension was stirred at room temperature for 30 min. At this point, propargyl bromide (0.1 mL, 0.86 mmol) was added dropwise. The reaction mixture was stirred for 16 h at room temperature before a further 0.5 equivalents of K_2CO_3 (0.05 g, 0.37 mmol) and propargyl bromide (0.05 mL, 0.43 mmol) were added. The reaction mixture was stirred for a further 8 h and then filtered through celite and concentrated *in vacuo* to provide the crude product as a yellow oil. The crude material was purified by flash column chromatography (pet. ether:Et_2O, 80:20 – 40:60) and concentrated *in vacuo* to provide *N*-(2-oxopropyl)-*N*-(prop-2-yn-1-yl)toluenesulfonamide **S6** (0.13 g, 0.51 mmol, **77%**) as a white solid.

^{1}H NMR ($CDCl_3$, 400 MHz): δ_H 7.76 – 7.70 (2H, m), 7.36 – 7.31 (2H, m), 4.19 (2H, d, ^4J = 2.2 Hz), 4.06 (2H, s), 2.45 (3H, s), 2.25 (3H, s), 2.15 (1H, t, ^4J = 2.5 Hz). **^{13}C NMR ($CDCl_3$, 101 MHz)**: δ_C 202.7, 143.5, 135.0, 129.2, 127.1, 75.7, 74.1, 54.7, 37.4, 26.6, 21.1. **IR (ν_{max}/cm^{-1})**: 3277, 3249, 1715, 1600, 1161. **Melting point**: 60 - 62 °C.

Preparation of *N*-(but-2-yn-1-yl)-*N*-(2-oxopropyl)toluenesulfonamide.[8]

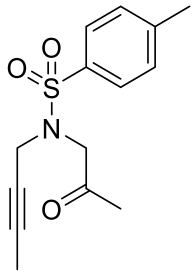

S7

N-(2-Oxopropyl)toluenesulfonamide **S5** (0.16 g, 0.70 mmol), K_2CO_3 (0.19 g, 1.4 mmol) and distilled DMF (6 mL) were added to a flame-dried, round-bottom flask equipped with a stirrer bar. The suspension was stirred at room temperature for 30 min. At this point, 1-bromobutyne (0.08 mL, 0.91 mmol) was added dropwise. The reaction mixture was stirred for 5 h at room temperature and then quenched by the addition of distilled H_2O (15 mL). Following this, Et_2O (15 mL) was added to create a biphasic mixture. The organic phase was separated and the aqueous phase was washed with Et_2O (3 × 15 mL). The combined organic extracts were washed with brine (10 mL), dried over Na_2SO_4, filtered, and then concentrated *in vacuo* to provide the crude product as a yellow oil. The crude material was purified by flash column chromatography (pet. ether:Et_2O, 80:20 – 40:60) and concentrated *in vacuo* to provide *N*-(but-2-yn-1-yl)-*N*-(2-oxopropyl)toluenesulfonamide **S7** (0.16 g, 0.57 mmol, **82%**) as a white solid.

^{1}H NMR ($CDCl_3$, 400 MHz): δ_H 7.75 – 7.71 (2H, m), 7.35 – 7.31 (2H, m), 4.09 (2H, q, ^5J = 2.3 Hz), 3.97 (2H, s), 2.44 (3H, s), 2.25 (3H, s), 1.62 (3H, t, ^5J = 2.4 Hz). **^{13}C NMR ($CDCl_3$, 101 MHz)**: δ_C 203.4, 143.3, 135.0, 129.1, 127.2, 82.1,

71.0, 55.1, 38.2, 26.6, 21.1, 2.8. **IR (v_{max}/cm^{-1})**: 2922, 1708, 1600, 1348, 1158. **Melting point**: 59 - 61 °C.

Towards 1-(but-2-yn-1-yloxy)propan-2-one S9.

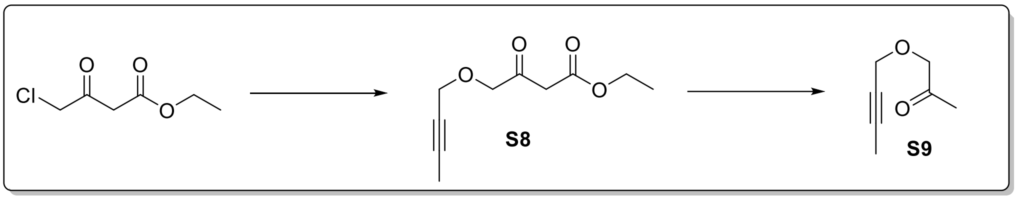

Preparation of ethyl 4-(but-2-yn-1-yloxy)-3-oxobutanoate.

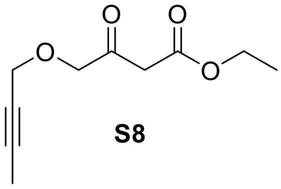

S8

NaH (1.22 g, 30.5 mmol) and dry THF (20 mL) were added to a flame-dried, round-bottom flask equipped with a stirrer bar. The suspension was cooled to 0 °C and ethyl 4-chloro-3-oxobutanoate (3.1 mL, 23.0 mmol) was added dropwise over 3 h using a syringe pump. At this point, but-2-yn-1-ol (1.15 mL, 19.2 mmol) was added dropwise over 2 h using a syringe pump. The reaction mixture was left to stir for 14 h at room temperature and then quenched by the addition of 2 M HCl (15 mL). Following this, EtOAc (20 mL) was added to create a biphasic mixture, which was separated, and the aqueous layer washed with EtOAc (3 × 20 mL). The combined organic extracts were washed with brine (15 mL), dried over Na_2SO_4, filtered, and concentrated *in vacuo* to provide the crude product as a yellow oil. The crude material was purified by flash column chromatography (pet. ether:Et_2O, 90:10) and concentrated *in vacuo* to provide ethyl 4-(but-2-yn-1-yloxy)-3-oxobutanoate **S8** (3.12 g, 15.7 mmol, **82%**) as a pale yellow oil.

^{1}H NMR (CDCl$_3$, 400 MHz): δ_H 4.24 – 4.18 (6H, m), 3.56 (2H, s), 1.86 (3H, t, ^5J = 2.3 Hz), 1.29 (3H, t, J = 7.1 Hz). **^{13}C NMR (CDCl$_3$, 101 MHz)**: δ_C 201.1, 166.5, 88.6, 83.5, 73.5, 60.9, 58.7, 45.6, 13.6, 3.0. **IR (v_{max}/cm^{-1})**: 2982, 1719, 1657, 1094. **HRMS m/z (NSI) Calc. for $C_{10}H_{15}O_4$ (M+H)**: 199.0965; **found**: 199.0964.

Preparation of 1-(but-2-yn-1-yloxy)propan-2-one.

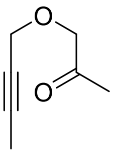

S9

Ethyl 4-(but-2-yn-1-yloxy)-3-oxobutanoate **S8** (1.10 g, 5.55 mmol), *p*-toluenesulfonic acid monohydrate (0.21 g, 1.11 mmol), ethanol (15 mL), and distilled water (11.2 mL) were added to a flame-dried, round-bottom flask equipped with a stirrer bar. This mixture was heated to reflux and stirred for 68 h before being quenched by the addition of sat. $NaHCO_3$ (15 mL). Following this, EtOAc (15 mL) was added to create a biphasic mixture, which was separated, and the aqueous layer washed with EtOAc (3 × 15 mL). The combined organic extracts were washed with brine (10 mL), dried over

Na_2SO_4, filtered, and concentrated *in vacuo* to 1-(but-2-yn-1-yloxy)propan-2-one **S9** (0.65 g, 5.15 mmol, **93%**) as a pale yellow oil.

^{1}H NMR ($CDCl_3$, 400 MHz): δ_H 4.23 (2H, q, ^5J = 2.3 Hz), 4.14 (2H, s), 2.19 (3H, s), 1.87 (3H, t, ^5J = 2.3 Hz). **^{13}C NMR ($CDCl_3$, 101 MHz)**: δ_C 205.9, 83.2, 74.0, 73.6, 58.5, 26.0, 3.0. **IR (v_{max}/cm^{-1})**: 2922, 2857, 1717, 1099. **HRMS m/z (NSI) Calc. for $C_7H_{14}NO_2$ (M+NH_4)**: 144.1019; **found**: 144.1016.

Towards dimethyl 2-(2-oxopropyl)-2-(3-phenylprop-2-yn-1-yl)malonate S10.

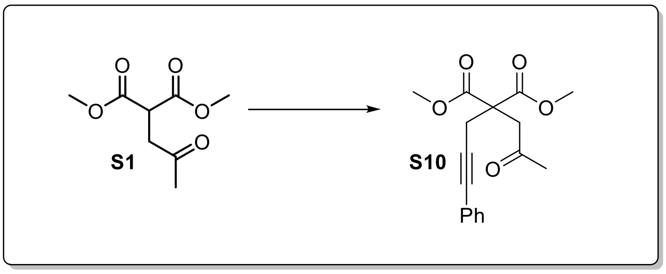

Preparation of dimethyl 2-(2-oxopropyl)-2-(3-phenylprop-2-yn-1-yl)malonate.

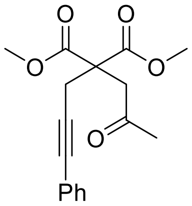

S10

NaH (0.22 g, 5.5 mmol) and distilled THF (17 mL) were added to a flame-dried, round-bottom flask equipped with a stirrer bar. The solution was cooled to 0 °C and dimethyl 2-(2-oxopropyl)malonate **S1** (0.94 g, 5 mmol) was added dropwise. The resulting mixture was stirred at 0 °C for 1 h. At this point, the reaction mixture was warmed to room temperature and (3-bromoprop-1-yn-1-yl)benzene (1.17 g, 6 mmol) was added dropwise. The reaction mixture was stirred for 16 h and then quenched by the addition of 2M HCl (20 mL). Following this, Et_2O (20 mL) was added to create a biphasic mixture. The organic phase was separated and the aqueous phase washed with Et_2O (3 × 20 mL). The combined organic extracts were washed with brine (15 mL), dried over Na_2SO_4, filtered, and concentrated *in vacuo* to provide the crude product as an orange oil. The crude material was purified by flash column chromatography (pet. ether:Et_2O, 70:30 – 50:50) and concentrated *in vacuo* to provide the desired product **S10** (1.27 g, 4.2 mmol, **84%**) as a colourless oil.

^{1}H NMR ($CDCl_3$, 400 MHz): δ_H 7.39 – 7.30 (5H, m), 3.78 (6H, s), 3.43 (2H, s), 3.24 (2H, s) 2.22 (3H, s). **^{13}C NMR ($CDCl_3$, 101 MHz):** δ_C 204.9, 169.2, 131.1, 127.8, 127.6, 122.5, 83.9, 83.3, 54.2, 52.6, 45.1, 29.7, 23.8. **IR (v_{max}/cm^{-1}):** 2953, 1736, 1717. **HRMS m/z (NSI/ion trap) Calc. for $C_{17}H_{19}O_5$ (M+H):** 303.1233**; found** 303.1230.

Towards dimethyl 2-(4-hydroxybut-2-yn-1-yl)-2-(2-oxopropyl)malonate S11.

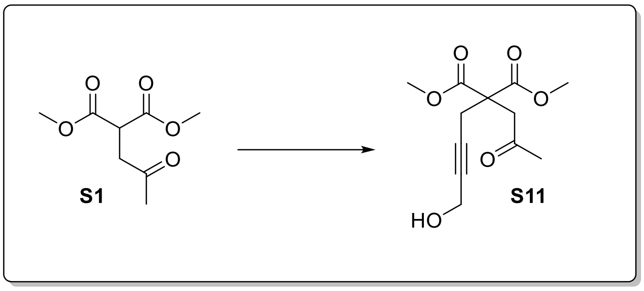

Preparation of dimethyl 2-(4-hydroxybut-2-yn-1-yl)-2-(2-oxopropyl)malonate.

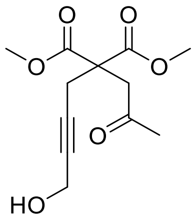

S11

NaH (0.11 g, 2.75 mmol) and dry THF (7 mL) were added to a flame-dried round-bottom flask equipped with a stirrer bar. The solution was cooled to 0 °C and dimethyl 2-(2-oxopropyl)malonate **S1** (0.47 g, 2.5 mmol) was added dropwise as a solution in THF (2.5 mL). The resulting mixture was stirred at 0 °C for 1 h. At this point, 4-bromobut-2-yn-1-ol (0.447 g, 3.0 mmol) was added dropwise as a solution in THF (2 mL) and the reaction mixture was warmed to rt. The reaction mixture was stirred for 3 h then quenched by the addition of saturated aqueous NH_4Cl solution (20 mL). EtOAc (20 mL) was added to create a biphasic mixture, which was separated, and the aqueous layer was washed with further quantities of EtOAc (3 × 20 mL). The combined organic extracts were washed with brine (15 mL), dried over Na_2SO_4, filtered, and concentrated *in vacuo* to give the crude product as a yellow oil. The crude material was purified by flash column chromatography (pet. ether 40 – 60:EtOAc, 70:30 – 30:70) and concentrated *in vacuo* to provide the desired product **S11** (0.59 g, 2.28 mmol, **91%**) as a pale yellow oil.

^{1}H NMR ($CDCl_3$, 400 MHz): δ_H 4.23 (2H, t, ^5J = 2.2 Hz), 3.75 (6H, s), 3.35 (2H, s), 3.05 (2H, t, ^5J = 2.2 Hz), 2.21 (3H, s), 1.90 (1H, s). **^{13}C NMR ($CDCl_3$, 101 MHz):** δ_C 204.8, 169.2, 81.4, 80.3, 53.9, 52.6, 50.6, 45.0, 29.7, 23.2. **IR (ν_{max}/cm^{-1}):** 3449, 2955, 1736, 1717. **HRMS m/z (NSI/ion trap) Calc. for $C_{12}H_{17}O_6$ (M+H)**: 257.1022; **found**: 257.1020.

Towards dimethyl 2-(3-oxopropyl)-2-(prop-2-yn-1-yl)malonate S14.

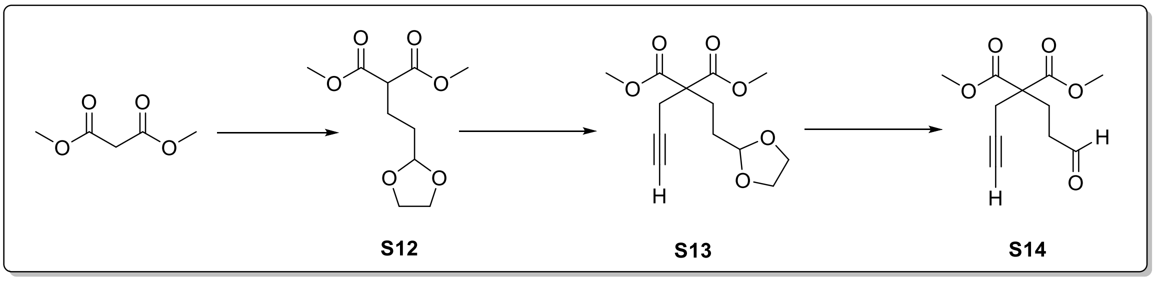

Preparation of dimethyl 2-(2-(1,3-dioxolan-2-yl)ethyl)malonate.

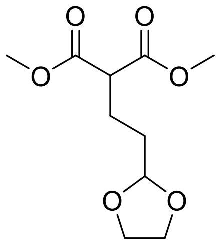

S12

NaH (0.30 g, 7.54 mmol) and distilled THF (20 mL) were added to a flame-dried, round-bottom flask equipped with a stirrer bar. This solution was cooled to 0 °C and dimethylmalonate (0.82 mL, 7.18 mmol) was added dropwise. The resulting mixture was stirred at 0 °C for 1 h. At this point, *tetra*-butylammonium iodide (0.66 g, 1.79 mmol) and 2-(2-bromoethyl)-1,3-dioxolane (0.76 mL, 6.46 mmol) were added dropwise and the reaction mixture was heated to reflux and stirred for 17 h. This was quenched by the addition of saturated aqueous ammonium chloride solution (20 mL). Following this, Et_2O (20 mL) was added to create a biphasic mixture, which was separated, and the aqueous layer washed with Et_2O (3 × 20 mL). The combined organic extracts were washed with brine (15 mL), dried over Na_2SO_4, filtered and concentrated *in vacuo* to provide the crude product as a yellow oil. The crude material was purified by flash column chromatography (pet. ether:Et_2O, 70:30 – 50:50) and concentrated *in vacuo* to provide dimethyl 2-(2-(1,3-dioxolan-2-yl)ethyl)malonate **S12** (1.36 g, 5.85 mmol, **91%**) as a colourless oil.

^{1}H NMR ($CDCl_3$, 400 MHz): δ_H 4.90 (1H, t, *J* = 4.5 Hz), 3.99 – 3.84 (4H, m), 3.75 (6H, s), 3.48 (1H, t, *J* = 7.6 Hz), 2.09 – 2.03 (2H, m), 1.75 – 1.70 (2H, m). **^{13}C NMR ($CDCl_3$, 101 MHz)**: δ_C 169.2, 103.2, 64.4, 52.0, 50.8, 30.7, 22.6. **IR (v_{max}/cm^{-1})**: 2955, 2890, 1731, 1438, 1141. **HRMS m/z (NSI) Calc. for $C_{10}H_{17}O_6$ (M+H)**: 233.1020; **found**: 233.1018.

Preparation of dimethyl 2-(2-(1,3-dioxolan-2-yl)ethyl)-2-(prop-2-yn-1-yl)malonate.[9]

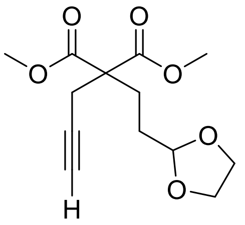

S13

NaH (0.27 g, 6.65 mmol) and dry THF (15 mL) were added to a flame-dried, round-bottom flask equipped with a stirrer bar. The solution was cooled to 0 °C and dimethyl 2-(2-(1,3-dioxolan-2-yl)ethyl)malonate **S12** (1.40 g, 6.05 mmol) was added dropwise. The resulting mixture was stirred at 0 °C for 1 h. At this point, the reaction mixture was warmed to room temperature and propargyl bromide (0.59 mL, 6.65 mmol) was added dropwise. The reaction mixture was stirred for 19 h and then quenched by the addition of distilled water (25 mL). Following this, Et_2O (25 mL) was added to create a biphasic mixture. The organic phase was separated and the aqueous phase washed with Et_2O (3 × 25 mL). The combined organic extracts were washed with brine (20 mL), dried over Na_2SO_4, filtered, and concentrated *in vacuo* to provide the crude product as an orange oil. The crude material was purified by flash column chromatography (pet. ether:Et_2O, 30:70) and concentrated *in vacuo* to provide dimethyl 2-(2-(1,3-dioxolan-2-yl)ethyl)-2-(prop-2-yn-1-yl)malonate **S13** (0.64 g, 2.35 mmol, **39%**) as a colourless oil.

^{1}H NMR ($CDCl_3$, 400 MHz): δ_H 4.88 (1H, t, *J* = 4.5 Hz), 4.02 – 3.82 (4H, m), 3.74 (6H, s), 2.82 (2H, d, ^4J = 2.7 Hz), 2.22 – 2.15 (2H, m), 2.02 (1H, t, ^4J = 2.7 Hz), 1.63 – 1.55 (2H, m). **^{13}C NMR ($CDCl_3$, 101 MHz)**: δ_C 170.0, 103.4, 78.0, 71.0, 64.4, 56.0, 52.3, 28.1, 26.0, 22.6. **IR (ν_{max}/cm^{-1})**: 3286, 2955, 1732, 1438, 1201.

Preparation of dimethyl 2-(3-oxopropyl)-2-(prop-2-yn-1-yl)malonate.[10]

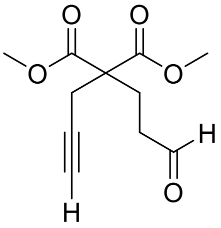

S14

Dimethyl 2-(2-(1,3-dioxolan-2-yl)ethyl)-2-(prop-2-yn-1-yl)malonate **S13** (0.64 g, 2.35 mmol) and dry THF (10 mL) were added to a flame-dried, round-bottom flask equipped with a stirrer bar. 6M HCl (48 mL) and H_2O:AcOH (1:1, 32 mL) were added to the solution and the reaction mixture was stirred at room temperature for 16 h. This was quenched by the addition of solid K_2CO_3 (35 g) and then by the addition of saturated aqueous $NaHCO_3$ solution (20 mL). Et_2O (25 mL) was added, the organic phase separated, and the aqueous phase washed with Et_2O (3 × 25 mL). The combined organic extracts were washed with brine (15 mL), dried over Na_2SO_4, and concentrated *in vacuo* to provide the crude product as a yellow oil. The crude material was purified by flash column chromatography (pet. ether:Et_2O, 70:30 – 50:50) and concentrated *in vacuo* to provide dimethyl 2-(3-oxopropyl)-2-(prop-2-yn-1-yl)malonate **S14** (0.44 g, 1.92 mmol, **82%**) as a pale yellow oil.

^{1}H NMR (CDCl$_3$, 400 MHz): δ_H 9.77 (1H, t, J = 1.2 Hz), 3.77 (6H, s), 2.85 (2H, d, ^4J = 2.7 Hz), 2.58 – 2.51 (2H, m), 2.45 – 2.38 (2H, m), 2.06 (1H, t, ^4J = 2.7 Hz). **^{13}C NMR (CDCl$_3$, 101 MHz)**: δ_C 199.9, 169.7, 77.7, 71.5, 55.5, 52.5, 38.5, 24.4, 23.2. **IR (ν_{max}/cm^{-1})**: 3282, 2956, 1437, 1202.

Towards *N*-(3-oxopropyl)-*N*-(prop-2-yn-1-yl)toluenesulfonamide S17.

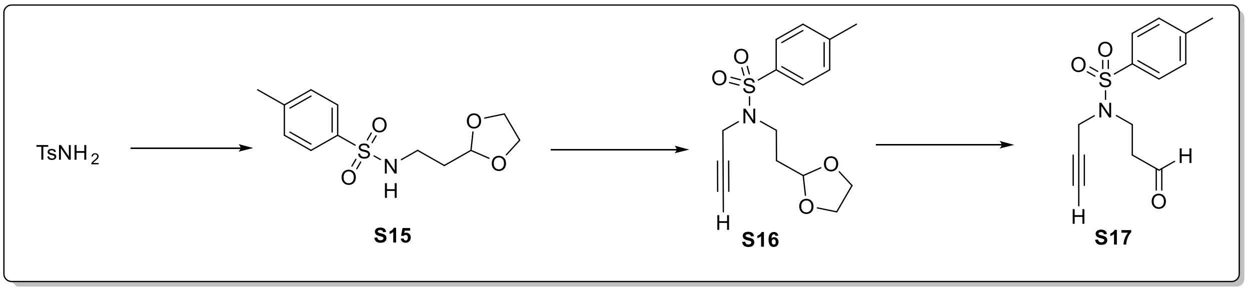

Preparation of *N*-(2-(1,3-dioxolan-2-yl)ethyl)toluenesulfonamide.[11]

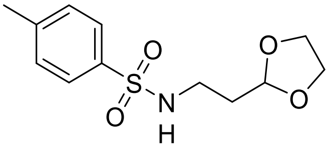

S15

2-(2-Bromoethyl)-1,3-dioxolane (1.3 mL, 11.06 mmol) and MeCN (25 mL) were added to a flame-dried, round-bottom flask equipped with a stirrer bar. *p*-Toluenesulfonamide (3.79 g, 22.12 mmol) and K_2CO_3 (3.06 g, 22.12 mmol) were added to this solution and the reaction mixture heated to reflux for 3 h. At this point, the reaction mixture was filtered through celite and concentrated *in vacuo*. The crude material was purified by flash column chromatography (pet. ether:Et_2O, 30:70) and concentrated *in vacuo* to provide *N*-(2-(1,3-dioxolan-2-yl)ethyl)toluenesulfonamide **S15** (2.40g, 8.85 mmol, **80%**) as a white solid.

^{1}H NMR (CDCl$_3$, 400 MHz): δ_H 7.78 – 7.72 (2H, m), 7.34 – 7.29 (2H, m), 5.24 (1H, t, J = 5.7 Hz), 4.84 (1H, t, J = 4.1 Hz), 3.94 – 3.75 (4H, m), 3.14 – 3.06 (2H, m), 2.43 (3H, s), 1.86 – 1.80 (2H, m). **^{13}C NMR (CDCl$_3$, 101 MHz)**: δ_C 142.8, 136.5, 129.2, 126.6, 102.8, 64.4, 37.9, 31.6, 21.0. **IR (ν_{max}/cm^{-1})**: 3257, 2961, 2895, 1595. **Melting point**: 59 – 61 °C.

Preparation of *N*-(2-(1,3-dioxolan-2-yl)ethyl)-*N*-(prop-2-yn-1-yl)toluenesulfonamide.[12]

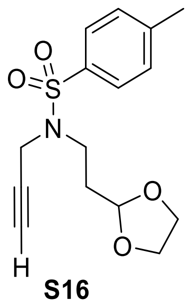

S16

N-(2-(1,3-Dioxolan-2-yl)ethyl)toluenesulfonamide **S15** (0.30 g, 1.1 mmol) was added to a flame-dried, round-bottom flask and dissolved in DMF (20 mL). NaH (0.06 g, 1.43 mmol) was added to this solution and the reaction mixture was stirred at room temperature for 30 min. At this point, propargyl bromide (0.20 mL, 1.81 mmol) was added dropwise and the reaction mixture was stirred at room temperature for 16 h. This was quenched by the addition of distilled water (20 mL). Et_2O (20 mL) was added, the organic phase separated, and the aqueous phase washed with Et_2O (3 × 20 mL). The combined organic extracts were washed with brine (15 mL), dried over Na_2SO_4, and concentrated *in vacuo* to provide the crude product as a yellow oil. The crude material was purified by flash column chromatography (pet. ether:Et_2O, 10:90) and concentrated *in vacuo* to provide *N*-(2-(1,3-dioxolan-2-yl)ethyl)-*N*-(prop-2-yn-1-yl)toluenesulfonamide **S16** (0.28 g, 0.9 mmol, **82%**) as a white solid.

^{1}H NMR ($CDCl_3$, 400 MHz): δ_H 7.78 – 7.73 (2H, m), 7.33 – 7.29 (2H, m), 4.94 (1H, t, *J* = 4.6 Hz), 4.18 (2H, d, ^4J = 2.5 Hz), 4.01 – 3.84 (4H, m), 3.40 – 3.33 (2H, m), 2.44 (3H, s), 2.05 (1H, t, ^4J = 2.5 Hz), 2.02 – 1.96 (2H, m). **^{13}C NMR ($CDCl_3$, 101 MHz)**: δ_C 143.0, 135.4, 129.0, 127.3, 101.8, 76.2, 73.2, 64.5, 41.5, 36.2, 31.8, 21.0. **IR (ν_{max}/cm^{-1})**: 3269, 2952, 2886, 1597. **Melting point**: 60 – 62 °C.

Preparation of *N*-(3-oxopropyl)-*N*-(prop-2-yn-1-yl)toluenesulfonamide.

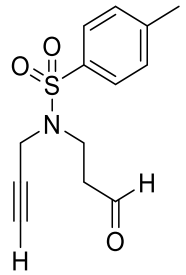

S17

N-(2-(1,3-Dioxolan-2-yl)ethyl)-*N*-(prop-2-yn-1-yl)toluenesulfonamide **S16** (0.92 g, 2.96 mmol) and dry THF (15 mL) were added to a flame-dried, round-bottom flask equipped with a stirrer bar. 6M HCl (60 mL) and H_2O:AcOH (1:1, 40 mL) were added to the solution and the reaction mixture was stirred at room temperature for 16 h. This was quenched by the addition of solid K_2CO_3 (40 g) and then by the addition of saturated aqueous $NaHCO_3$ solution (20 mL). Et_2O (25 mL) was added, the organic phase separated, and the aqueous phase washed with Et_2O (3 × 25 mL). The combined organic extracts were washed with brine (15 mL), dried over Na_2SO_4 and concentrated *in vacuo* to provide the crude product as a yellow oil. The crude material was purified by flash column chromatography (pet. ether:Et_2O, 70:30 – 80:20) and concentrated *in vacuo* to provide *N*-(3-oxopropyl)-*N*-(prop-2-yn-1-yl)toluenesulfonamide **S17** (0.69 g, 2.62 mmol, **88%**) as a pale yellow oil.

^{1}H NMR (CDCl$_3$, 400 MHz): δ_H 9.82 (1H, t, J = 1.1 Hz), 7.77 – 7.75 (2H, m), 7.34 – 7.32 (2H, m), 4.16 (2H, d, ^4J = 2.4 Hz), 3.54 (2H, t, J = 6.8 Hz), 2.89 (2H, td, J = 6.9 Hz, J = 1.1 Hz), 2.46 (3H, s), 2.09 (1H, t, ^4J = 2.5 Hz). **^{13}C NMR (CDCl$_3$, 101 MHz)**: δ_C 199.5, 143.4, 134.8, 129.1, 127.3, 76.5, 73.5, 42.6, 40.1, 37.2, 21.1. **IR (v_{max}/cm^{-1})**: 3279, 1721, 1597, 1493. **HRMS m/z (NSI) Calc. for $C_{13}H_{16}NO_3S$ (M+H)**: 266.0845; **found**: 266.0844.

Towards dimethyl 2-(4-oxobutyl)-2-(prop-2-yn-1-yl)malonate S20.

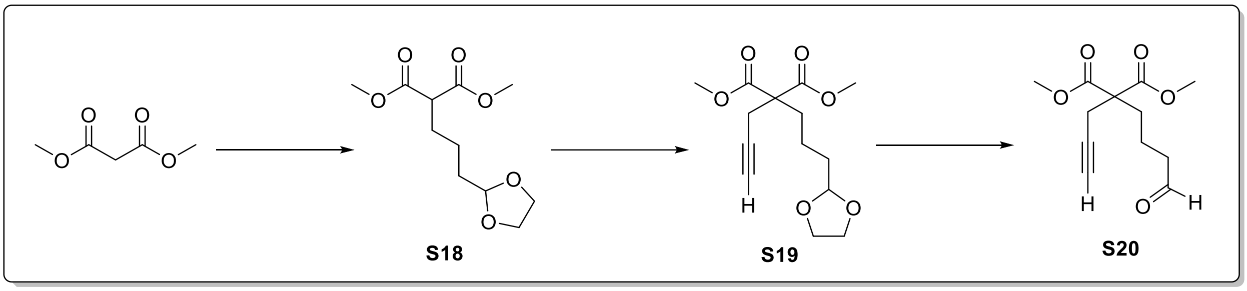

Preparation of dimethyl 2-(3-(1,3-dioxolan-2-yl)propyl)malonate.

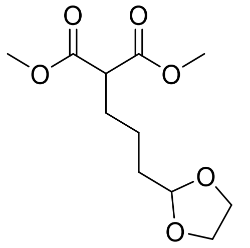

S18

NaH (0.12 g, 2.97 mmol) and THF:*N*-methyl-2-pyrrolidone (3:1, 12 mL) were added to a flame-dried, round-bottom flask equipped with a stirrer bar. The solution was cooled to 0 °C and dimethylmalonate (0.31 mL, 2.70 mmol) was added dropwise. The resulting mixture was stirred at 0 °C for 1 h. At this point, 2-(3-chloropropyl)-1,3-dioxolane (0.32 mL, 2.43 mmol) was added dropwise followed by TBAI (0.25 g, 0.68 mmol) and the reaction mixture was heated to reflux. The reaction mixture was stirred for 24 h and then quenched by the addition of brine (10 mL). Following this, EtOAc (50 mL) was added to create a biphasic mixture, which was separated, and the aqueous layer washed with EtOAc (3 × 50 mL). The combined organic extracts were washed with brine (15 mL), dried over Na_2SO_4, filtered, and concentrated *in vacuo* to provide the crude product as a yellow oil. The crude material was purified by flash column chromatography (pet. ether:Et_2O, 30:70) and concentrated *in vacuo* to provide dimethyl 2-(3-(1,3-dioxolan-2-yl)propyl)malonate **S18** (0.38 g, 1.56 mmol, **64%**) as a colourless oil.

^{1}H NMR (CDCl$_3$, 400 MHz): δ_H 4.86 (1H, t, J = 4.7 Hz), 3.99 – 3.83 (4H, m), 3.75 (6H, s), 3.39 (1H, t, J = 7.5 Hz), 2.00 – 1.94 (2H, m), 1.73 – 1.69 (2H, m), 1.51 – 1.45 (2H, m). **^{13}C NMR (CDCl$_3$, 101 MHz)**: δ_C 169.3, 103.6, 64.4, 52.0, 51.2, 32.9, 28.2, 21.3. **IR (v_{max}/cm^{-1})**: 2953, 2878, 1749, 1732, 1435, 1142. **HRMS m/z (NSI) Calc. for $C_{11}H_{19}O_6$ (M+H)**: 247.1176; **found**: 247.1178.

Preparation of dimethyl 2-(3-(1,3-dioxolan-2-yl)propyl)-2-(prop-2-yn-1-yl)malonate.[13]

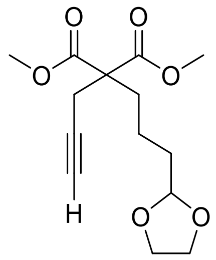

S19

NaH (0.12 g, 2.94 mmol) and dry THF (12 mL) were added to a flame-dried, round-bottom flask equipped with a stirrer bar. The solution was cooled to 0 °C and dimethyl 2-(3-(1,3-dioxolan-2-yl)propyl)malonate **S18** (0.60 g, 2.45 mmol) was added dropwise. The resulting mixture was stirred at 0 °C for 1 h. At this point, propargyl bromide (0.33 mL, 3.67 mmol) was added dropwise and the reaction mixture was warmed to rt. The reaction mixture was stirred for 3 h and then quenched by the addition of NH_4Cl (20 mL). Following this, EtOAc (20 mL) was added to create a biphasic mixture, which was separated, and the aqueous layer washed with EtOAc (3 × 20 mL). The combined organic extracts were washed with brine (15 mL), dried over Na_2SO_4, filtered, and concentrated *in vacuo* to provide the crude product as a yellow oil. The crude material was purified by flushing through a plug of silica gel and concentrated *in vacuo* to provide dimethyl 2-(3-(1,3-dioxolan-2-yl)propyl)-2-(prop-2-yn-1-yl)malonate **S19** (0.68g, 2.40 mmol, **98%**) as a pale yellow oil.

^{1}H NMR ($CDCl_3$, 400 MHz): δ_H 4.86 (1H, t, *J* = 4.6 Hz), 3.99 – 3.83 (4H, m), 3.75 (6H, s), 2.84 (2H, d, ^4J = 2.8 Hz), 2.15 – 2.07 (2H, m), 2.01 (1H, t, ^4J = 2.7 Hz), 1.74 – 1.66 (2H, m), 1.39 – 1.28 (2H, m). **^{13}C NMR ($CDCl_3$, 101 MHz)**: δ_C 170.1, 103.5, 78.3, 70.8, 64.4, 56.5, 52.3, 33.4, 31.5, 22.3, 18.1. **IR (ν_{max}/cm^{-1})**: 3281, 2955, 2880, 1730, 1435, 1200.

Preparation of dimethyl 2-(4-oxobutyl)-2-(prop-2-yn-1-yl)malonate.[10]

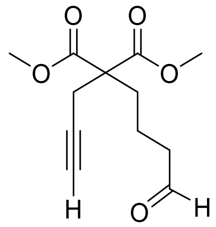

S20

Dimethyl 2-(3-(1,3-dioxolan-2-yl)propyl)-2-(prop-2-yn-1-yl)malonate **S19** (0.64 g, 2.23 mmol) and dry THF (10 mL) were added to a flame-dried, round-bottom flask equipped with a stirrer bar. 6M HCl (40 mL) and H_2O:AcOH (1:1, 25 mL) were added to the solution and the reaction mixture was stirred at room temperature for 24 h. This was quenched by the addition of solid K_2CO_3 (25 g) and then by the addition of saturated aqueous $NaHCO_3$ solution (20 mL). Et_2O (25 mL) was added, the organic phase separated, and the aqueous phase washed with Et_2O (3 × 25 mL). The combined organic extracts were washed with brine (15 mL), dried over Na_2SO_4, and concentrated *in vacuo* to provide dimethyl 2-(4-oxobutyl)-2-(prop-2-yn-1-yl)malonate **S20** (0.45 g, 1.87 mmol, **84%**) as a yellow oil.

^{1}H NMR ($CDCl_3$, 400 MHz): δ_H 9.78 (1H, t, *J* = 1.4 Hz), 3.77 (6H, s), 2.88 (2H, d, ^4J = 2.7 Hz), 2.50 (2H, td, *J* = 7.3 Hz, *J* = 1.4 Hz), 2.13 – 2.06 (2H, m), 2.03 (1H, t, ^4J = 2.7 Hz), 1.62 – 1.54 (2H, m). **^{13}C NMR ($CDCl_3$, 101 MHz)**: δ_C 201.0, 169.9, 78.1, 71.1, 56.3, 52.4, 43.2, 31.1, 22.4, 16.4. **IR (ν_{max}/cm^{-1})**: 3281, 2955, 1726, 1200, 1179.

b. Silyl enol ether substrates

Typical Procedure A for the preparation of silyl enol ethers

Preparation of dimethyl 2-(2-((*tert*-butyldimethylsilyl)oxy)allyl)-2-(prop-2-yn-1-yl)malonate.

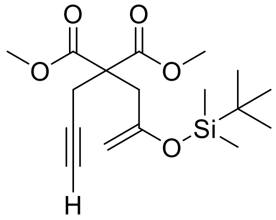

2

Dimethyl 2-(2-oxopropyl)-2-(prop-2-yn-1-yl)malonate **1** (0.57 g, 2.52 mmol) and distilled DCE (25 mL) were added to a flame-dried, round-bottom flask equipped with a stirrer bar. The solution was cooled to 0 °C and DIPEA (0.49 mL, 2.78 mmol) was added dropwise. The mixture was stirred at 0 °C for 1 h. At this point, the reaction mixture was allowed to warm to room temperature and TBSOTf (0.64 mL, 2.78 mmol) was added dropwise. The reaction was stirred for 16 h at room temperature then quenched by the addition of saturated aqueous $NaHCO_3$ solution (25 mL). Et_2O (25 mL) was added, the organic phase separated, and the aqueous phase washed with Et_2O (3 × 25 mL). The combined organic extracts were washed with brine (20 mL), dried over Na_2SO_4, and concentrated *in vacuo* to provide the crude product as a yellow oil. The crude material was purified by flash column chromatography (pet. ether:Et_2O, 80:20) and concentrated *in vacuo* to provide dimethyl 2-(2-((*tert*-butyldimethylsilyl)oxy)allyl)-2-(prop-2-yn-1-yl)malonate **2** (0.62 g, 1.81 mmol, **72%**) as a colourless oil.

^{1}H NMR ($CDCl_3$, 400 MHz): δ_H 4.18 (1H, d, ^2J = 1.0 Hz), 4.17 (1H, d, ^2J = 1.0 Hz), 3.75 (6H, s), 2.96 (2H, d, ^4J = 2.7 Hz), 2.87 (2H, s), 2.02 (1H, t, ^4J = 2.7 Hz), 0.94 (9H, s), 0.18 (6H, s). **^{13}C NMR ($CDCl_3$, 101 MHz)**: δ_C 169.6, 154.2, 93.5, 79.0, 70.8, 55.8, 52.2, 38.5, 25.3, 22.2, 17.8, -5.1. **IR (ν_{max}/cm^{-1})**: 3289, 2954, 1742, 1632, 1182. **HRMS m/z (ESI) Calc. for $C_{17}H_{29}O_5Si$ (M+H)**: 341.1779; **found** 341.1778.

The following experiments were carried out according to ***Typical Procedure A****.*

Data are reported as: (a) carbonyl compound; (b) volume of DCE; (c) DIPEA; (d) silylating agent; (e) isolated yield; and (f) compound appearance. Individual analysis for each silyl enol ether compound is provided.

Preparation of dimethyl 2-(prop-2-yn-1-yl)-2-(2-((triethylsilyl)oxy)allyl)malonate.

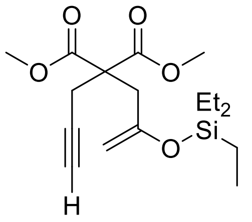

5a

(a) Dimethyl 2-(2-oxopropyl)-2-(prop-2-yn-1-yl)malonate **1**, 0.50 g, 2.21 mmol; (b) 22 mL; (c) DIPEA, 0.42 mL, 2.43 mmol; (d) TESOTf, 0.55 mL, 2.43 mmol; (e) dimethyl 2-(prop-2-yn-1-yl)-2-(2-((triethylsilyl)oxy)allyl)malonate **5a**, 0.68 g, 2.00 mmol, **90%**; and (f) colourless oil.

^{1}H NMR (CDCl$_3$, 400 MHz): δ_H 4.11 (1H, d, ^2J = 1.1 Hz), 4.09 (1H, d, ^2J = 1.1 Hz), 3.68 (6H, s), 2.86 (2H, d, ^4J = 2.7 Hz), 2.81 (2H, s), 1.97 (1H, t, ^4J = 2.7 Hz), 0.93 (9H, t, J = 7.9 Hz), 0.64 (6H, q, J = 8.0 Hz). **^{13}C NMR (CDCl$_3$, 101 MHz)**: δ_C 169.5, 153.9, 92.3, 78.8, 70.7, 55.2, 52.0, 38.5, 21.9, 6.0, 4.0. **IR (ν_{max}/cm^{-1})**: 3290, 2955, 2931, 2858, 1742, 1631, 1182. **HRMS m/z (ESI) Calc. for $C_{17}H_{29}O_5Si$ (M+H)**: 341.1779; **found** 341.1777.

Preparation of dimethyl 2-(prop-2-yn-1-yl)-2-(2-((triisopropylsilyl)oxy)allyl)malonate.

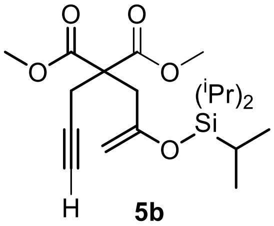

5b

(a) Dimethyl 2-(2-oxopropyl)-2-(prop-2-yn-1-yl)malonate **1**, 0.079 g, 0.35 mmol; (b) 3.5 mL; (c) DIPEA, 0.16 mL, 0.38 mmol; (d) TIPSOTf, 0.05 mL, 0.38 mmol; (e) dimethyl 2-(prop-2-yn-1-yl)-2-(2-((triisopropylsilyl)oxy)allyl)malonate **5b**, 0.09 g, 0.24 mmol, **69%**; and (f) colourless oil.

^{1}H NMR (**400 MHz, CDCl$_3$**): 4.07 (2H, s), 3.66 (6H, s), 2.89 (2H, d, ^4J = 2.6 Hz), 2.81 (2H, s), 1.93 (1H, t, ^4J = 2.6 Hz), 1.16 – 1.11 (3H, m) 1.01 (18H, d, J = 7.0 Hz). **^{13}C NMR** (**100 MHz, CDCl$_3$**): 169.7, 154.4, 92.7, 79.0, 70.8, 55.9, 52.2, 38.5, 22.2, 17.4, 12.2. **IR (ν_{max}/cm^{-1})**: 3320, 2950, 1743, 1625. **HRMS m/z** (**ESI**): **Calc. for $C_{20}H_{35}O_5Si$ (M+H):** 383.2248. **found:** 383.2246.

Preparation of *N*-(2-((*tert*-butyldimethylsilyl)oxy)allyl)-4-methyl-*N*-(prop-2-yn-1-yl)benzenesulfonamide.

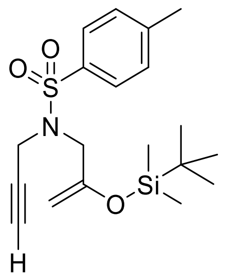

S21

(a) 4-Methyl-*N*-(2-oxopropyl)-*N*-(prop-2-yn-1-yl)benzenesulfonamide **S6**, 0.25 g, 0.94 mmol; (b) 9.5 mL; (c) DIPEA, 0.18 mL, 1.04 mmol; (d) TBSOTf, 0.24 mL, 1.04 mmol; (e) *N*-(2-((*tert*-butyldimethylsilyl)oxy)allyl)-4-methyl-*N*-(prop-2-yn-1-yl)benzenesulfonamide **S21**, 0.28 g, 0.74 mmol, **79%**; and (f) white gum.

^{1}H NMR (CDCl$_3$, 400 MHz): δ_H 7.77 – 7.74 (2H, m), 7.31 – 7.29 (2H, m), 4.35 (1H, d, ^2J = 1.3 Hz), 4.27 (1H, d, ^2J = 1.3 Hz), 4.18 (2H, d, ^4J = 2.4 Hz), 3.76 (2H, s), 2.44 (3H, s), 2.02 (1H, t, ^4J = 2.5 Hz), 0.94 (9H, s), 0.20 (6H, s). **^{13}C NMR (CDCl$_3$, 101 MHz)**: δ_C 152.2, 142.9, 135.9, 128.9, 127.2, 92.7, 76.2, 73.2, 49.7, 35.7, 25.1, 21.0, 17.5, -5.3. **IR (ν_{max}/cm^{-1})**: 3304, 2930, 1634, 1165. **HRMS m/z (NSI) Calc. for $C_{19}H_{30}NO_3SSi$ (M+H)**: 380.1710; **found**: 380.1703.

Preparation of *N*-(prop-2-yn-1-yl)-*N*-(2-((triethylsilyl)oxy)allyl)toluenesulfonamide.

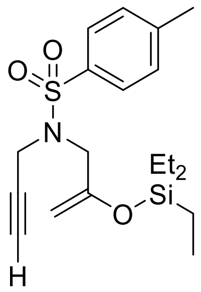

S22

(a) *N*-(2-Oxopropyl)-*N*-(prop-2-yn-1-yl)toluenesulfonamide **S6**, 0.5 g, 1.89 mmol; (b) 19 mL; (c) DIPEA, 0.36 mL, 2.08 mmol; (d) TESOTf, 0.47 mL, 2.08 mmol; (e) *N*-(prop-2-yn-1-yl)-*N*-(2-((triethylsilyl)oxy)allyl)toluenesulfonamide **S22**, 0.585 g, 1.54 mmol, **82%**; and (f) colourless oil.

^{1}H NMR (CDCl$_3$, 400 MHz): δ_H 7.78 – 7.74 (2H, m), 7.32 – 7.28 (2H, m), 4.36 (1H, d, ^2J = 1.5 Hz), 4.28 (1H, d, ^2J = 1.5 Hz) 4.18 (2H, d, ^4J = 2.4 Hz), 3.77 (2H, s), 2.43 (3H, s), 2.03 (1H, t, ^4J = 2.5 Hz), 0.99 (9H, t, J = 7.9 Hz), 0.71 (6H, q, J = 7.9 Hz). **^{13}C NMR (CDCl$_3$, 101 MHz)**: δ_C 152.3, 142.9, 135.9, 128.9, 127.3, 92.0, 76.3, 73.2, 49.6, 35.8, 21.0, 6.1, 4.2. **IR (ν_{max}/cm^{-1})**: 3273, 2965, 2912, 2877, 1639, 1600, 1162. **HRMS m/z (ESI) Calc. for $C_{19}H_{30}NO_3SSi$ (M+H)**: 380.1710; **found** 380.1707.

Preparation of dimethyl 2-(but-2-yn-1-yl)-2-(2-((*tert*-butyldimethylsilyl)oxy)allyl)malonate.

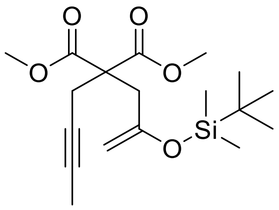

S23

(a) Dimethyl 2-(but-2-yn-1-yl)-2-(2-oxopropyl)malonate **S2**, 2.01 g, 8.37 mmol; (b) 8.5 mL; (c) DIPEA, 1.60 mL, 9.20 mmol; (d) TBSOTf, 2.11 mL, 9.20 mmol; (e) dimethyl 2-(but-2-yn-1-yl)-2-(2-((*tert*-butyldimethylsilyl)oxy)allyl)malonate **S23**, 2.80 g, 7.90 mmol, **94%**; and (f) colourless oil.

^{1}H NMR ($CDCl_3$, 400 MHz): δ_H 4.15 (1H, d, ^{2}J = 1.0 Hz), 4.14 (1H, d, ^{2}J = 1.0 Hz), 3.73 (6H, s), 2.88 (2H, q, ^{5}J = 2.6 Hz), 2.83 (2H, s), 1.76 (3H, t, ^{5}J = 2.5 Hz), 0.93 (9H, s), 0.17 (6H, s). **^{13}C NMR ($CDCl_3$, 101 MHz)**: δ_C 170.0, 154.4, 93.3, 78.2, 73.5, 56.2, 52.1, 38.5, 25.2, 22.5, 17.7, 2.9, -5.1. **IR (v_{max}/cm^{-1})**: 2954, 1742, 1632, 1182. **HRMS m/z (NSI) Calc. for $C_{18}H_{31}O_5Si$ (M+H)**: 355.1935; **found** 355.1935.

Preparation of dimethyl 2-(but-2-yn-1-yl)-2-(2-((triethylsilyl)oxy)allyl)malonate.

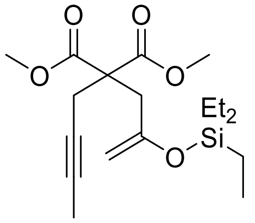

S24

(a) Dimethyl 2-(but-2-yn-1-yl)-2-(2-oxopropyl)malonate **S2**, 0.96 g, 4.00 mmol; (b) 40 mL; (c) DIPEA, 0.77 mL, 4.4 mmol; (d) TESOTf, 1.00 mL, 4.4 mmol; (e) dimethyl 2-(but-2-yn-1-yl)-2-(2-((triethylsilyl)oxy)allyl)malonate **S24**, 1.02 g, 2.88 mmol, **72 %**; and (f) colourless oil.

^{1}H NMR ($CDCl_3$, 400 MHz): δ_H 4.16 (1H, d, ^{2}J = 0.9 Hz), 4.13 (1H, d, ^{2}J = 0.9 Hz), 3.73 (6H, s), 2.86 (2H, q, ^{5}J = 2.4 Hz), 2.85 (2H, s), 1.77 (3H, t, ^{5}J = 2.5 Hz), 0.99 (9H, t, J = 7.9 Hz), 0.70 (6H, q, J = 7.9 Hz). **^{13}C NMR ($CDCl_3$, 101 MHz)**: δ_C 170.0, 154.2, 92.3, 78.1, 73.4, 55.6, 52.1, 38.6, 22.3, 6.1, 4.1, 3.0. **IR (v_{max}/cm^{-1})**: 2955, 2916, 2879, 1742, 1629, 1182. **HRMS m/z (ESI) Calc. for $C_{18}H_{31}O_5Si$ (M+H)**: 355.1935; **found** 355.1936.

Preparation of *N*-(but-2-yn-1-yl)-*N*-(2-((triethylsilyl)oxy)allyl)toluenesulfonamide.

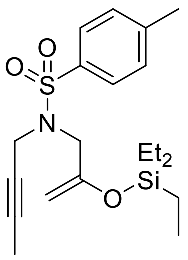

S25

(a) *N*-(2-Oxopropyl)-*N*-(but-2-yn-1-yl)toluenesulfonamide **S7**, 0.87 g, 3.10 mmol; (b) 31 mL; (c) DIPEA, 0.59 mL, 3.41 mmol; (d) TESOTf, 0.73 mL, 3.41 mmol; (e) *N*-(but-2-yn-1-yl)-*N*-(2-((triethylsilyl)oxy)allyl)toluenesulfonamide **S25**, 1.09 g, 2.76 mmol, **89%**; (f) colourless oil.

^{1}H NMR ($CDCl_3$, 400 MHz): δ_H 7.76 (2H, d, J = 8.0 Hz), 7.30 (2H, d, J = 8.0 Hz), 4.33 (1H, d, ^{2}J = 1.3 Hz), 4.26 (1H, d, ^{2}J = 1.3 Hz), 4.11 (2H, q, ^{5}J = 2.3 Hz), 3.73 (2H, s), 2.44 (3H, s), 1.56 (3H, t, ^{5}J = 2.4 Hz), 0.99 (9H, t, J = 7.9 Hz), 0.71 (6H, q, J = 7.8 Hz). **^{13}C NMR ($CDCl_3$, 101 MHz)**: δ_C 152.6, 142.6, 136.1, 128.7, 127.3, 91.7, 81.0, 71.4, 49.6, 36.4, 21.0, 6.1, 4.3, 2.7. **IR (ν_{max}/cm^{-1})**: 2955, 2914, 2977, 1639, 1352, 1162. **HRMS m/z (ESI) Calc. for $C_{20}H_{32}NO_3SSi$ (M+H)**: 394.1867; **found** 394.1865.

Preparation of ((3-(but-2-yn-1-yloxy)prop-1-en-2-yl)oxy)(*tert*-butyl)dimethylsilane.

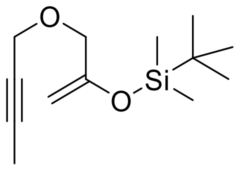

S26

(a) 1-(But-2-yn-1-yloxy)propan-2-one **S9**, 0.613 g, 4.86 mmol; (b) 49 mL; (c) DIPEA, 1.02 mL, 5.83 mmol; (d) TBSOTf, 1.34 mL, 5.83 mmol; (e) ((3-(but-2-yn-1-yloxy)prop-1-en-2-yl)oxy)(*tert*-butyl)dimethylsilane **S26**, 0.89 g, 3.68 mmol, **76%**; and (f) colourless oil.

^{1}H NMR ($CDCl_3$, 400 MHz): δ_H 4.40 – 4.38 (1H, m), 4.28 (1H, apparent s), 4.16 (2H, q, ^{5}J = 2.3 Hz), 3.90 (2H, apparent s), 1.87 (3H, t, ^{5}J = 2.3 Hz), 0.95 (9H, s), 0.19 (6H, s). **^{13}C NMR ($CDCl_3$, 101 MHz)**: δ_C 154.5, 91.4, 81.9, 74.5, 70.0, 57.5, 25.1, 17.6, 3.1, -5.2. **IR (ν_{max}/cm^{-1})**: 2955, 2928, 2857, 1638, 1250, 826. **HRMS m/z (NSI) Calc. for $C_{13}H_{25}O_2Si$ (M+H)**: 241.1618; **found**: 241.1619.

Preparation of dimethyl 2-(2-((*tert*-butyldimethylsilyl)oxy)allyl)-2-(3-phenylprop-2-yn-1-yl)malonate.

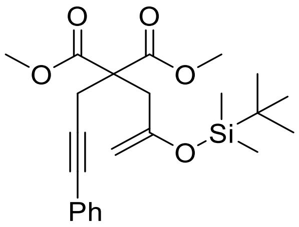

S27

(a) Dimethyl 2-(2-oxopropyl)-2-(3-phenylprop-2-yn-1-yl)malonate **S10,** 1.26 g, 4.17 mmol; (b) 42 mL; (c) DIPEA, 0.88 mL, 6 mmol; (d) TBSOTf, 1.15 mL, 5.01 mmol; (e) dimethyl 2-(2-((*tert*-butyldimethylsilyl)oxy)allyl)-2-(3-phenylprop-2-yn-1-yl)malonate **S27**, 1.58 g, 3.8 mmol, **91%**; and (f) colourless oil.

^{1}H NMR ($CDCl_3$, 400 MHz): δ_H 7.40 – 7.27 (5H, m), 4.21 (1H, d, ^2J = 1.0 Hz), 4.18 (1H, d, ^2J = 1.0 Hz), 3.77 (6H, s), 3.18 (2H, s), 2.92 (2H, s), 0.95 (9H, s), 0.19 (6H, s). **^{13}C NMR ($CDCl_3$, 101 MHz):** δ_C 169.8, 154.3, 131.1, 127.7, 127.3, 122.9, 93.5, 84.5, 83.0, 56.3, 52.3, 38.7, 25.3, 23.1, 17.8, -5.1. **IR (v_{max}/cm^{-1}):** 1738, 1628. **HRMS m/z (NSI/ion trap) m/z Calc. for $C_{23}H_{33}O_5Si$ (M+H):** 417.2097**; found** 417.2098.

Preparation of dimethyl 2-(2-((*tert*-butyldimethylsilyl)oxy)allyl)-2-(4-((*tert*-butyldimethylsilyl)oxy)but-2-yn-1-yl)malonate.

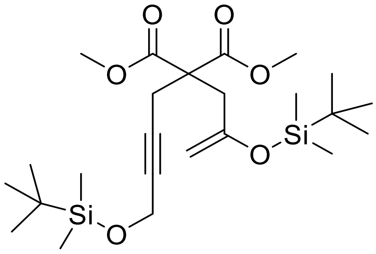

S28

(a) Dimethyl 2-(4-hydroxybut-2-yn-1-yl)-2-(2-oxopropyl)malonate **S11**, 0.43 g, 1.42 mmol; (b) 14 mL; (c) DIPEA, 0.54 mL, 3.11 mmol; (d) TBSOTf, 0.72 mL, 3.11 mmol; (e) dimethyl 2-(2-((*tert*-butyldimethylsilyl)oxy)allyl)-2-(4-((*tert*-butyldimethylsilyl)oxy)but-2-yn-1-yl)malonate **S28**, 0.65 g, 1.33 mmol, **94%**; and (f) colourless oil.

^{1}H NMR ($CDCl_3$, 400 MHz): δ_H 4.28 (2H, t, ^5J = 2.1 Hz), 4.17 (1H, d, ^2J = 1.0 Hz), 4.15 (1H, d, ^2J = 1.0 Hz), 3.73 (6H, s), 2.98 (2H, t, ^5J = 2.1 Hz), 2.84 (2H, s), 0.93 (9H, s), 0.91 (9H, s), 0.17 (6H, s), 0.11 (6H, s). **^{13}C NMR ($CDCl_3$, 101 MHz)**: δ_C 169.7 154.2, 93.5, 81.3, 79.5, 55.8, 52.2, 51.2, 38.6, 25.3, 22.5, 17.8, -3.5, -5.1, -5.6, -5.7. **IR (v_{max}/cm^{-1}):** 2953, 2930, 2859, 1744. **HRMS m/z (APCI) Calc. for $C_{24}H_{45}O_6Si_2$ (M+H)$^+$**: 485.2749; **found** 485.2735.

Towards the preparation of dimethyl 2-(3-((*tert*-butyldimethylsilyl)oxy)cyclopent-2-en-1-yl)-2-(prop-2-yn-1-yl)malonate S30 and dimethyl 2-(3-((*tert*-butyldimethylsilyl)oxy)cyclohex-2-en-1-yl)-2-(prop-2-yn-1-yl)malonate S31.

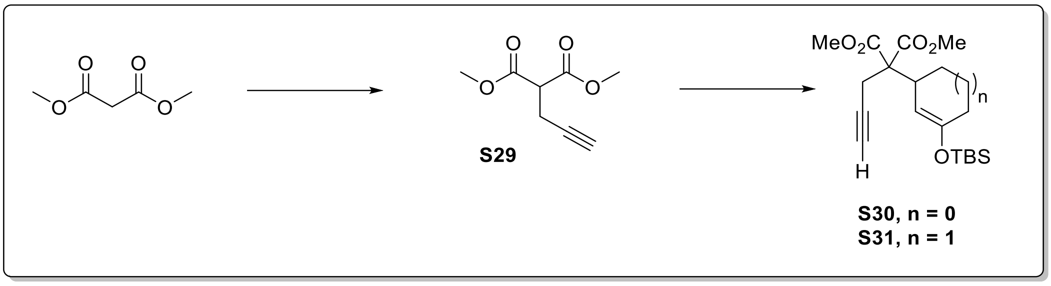

Preparation of dimethyl 2-(prop-2-yn-1-yl)malonate.[14]

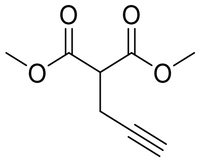

S29

Propargyl bromide, (3.72 g, 25 mmol) was added to a suspension of dimethyl malonate (4.95 g, 37.5 mmol) and K_2CO_3 (10.4 g, 75 mmol) in acetone (125 mL). The mixture was stirred at room temperature for 24 h and then quenched with distilled H_2O (60 mL). The mixture was extracted with EtOAc (3 × 50 mL), washed with brine, then dried over Na_2SO_4, filtered, and the solvent was removed *in vacuo* to give the crude product as a yellow oil. The crude material was purified by flash column chromatography (pet. ether:Et_2O, 90:10) and concentrated *in vacuo* to provide the desired product **S29** (2.44 g, 14.3 mmol, **57%**) as a colourless oil.

^{1}H NMR ($CDCl_3$, 400 MHz): δ_H 3.79 (6H, s), 3.63 (1H, t, ^3J = 7.6 Hz), 2.81 (2H, dd, ^3J = 7.7 Hz, ^4J = 2.7 Hz), 2.04 (1H, t, ^4J = 2.7 Hz). **^{13}C NMR ($CDCl_3$, 101 MHz):** δ_C 167.7, 79.3, 70.0, 52.4, 50.4, 18.0. **IR (ν_{max}/cm^{-1}):** 3287, 2957, 2359, 1732. **HRMS m/z (NSI) Calc. for $C_8H_{11}O_4$ $(M+H)^+$**: 171.0652; **found** 171.0650.

Preparation of dimethyl 2-(3-((*tert*-butyldimethylsilyl)oxy)cyclopent-2-en-1-yl)-2-(prop-2-yn-1-yl)malonate.

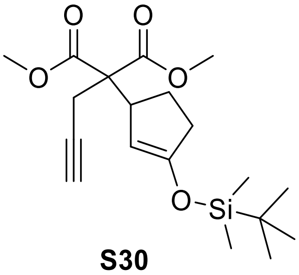

S30

NaH (0.13 g, 3.3 mmol) and dry THF (15 mL) were added to a flame-dried round-bottom flask equipped with a stirrer bar. The solution was cooled to 0 °C and dimethyl 2-(prop-2-yn-1-yl)malonate **S29** (0.51 g, 3 mmol) was added dropwise as a solution in THF (5 mL). The resulting mixture was stirred at 0 °C for 1 h. At this point, 2-cyclopentene-1-one (0.24 mL, 3 mmol) and TBSOTf (0.76 mL, 3.3 mmol) were added and the reaction mixture warmed to rt and stirred for 2 h. The reaction was quenched by pouring into a saturated aqueous solution of KH_2PO_4 and this was extracted with Et_2O (2 x 10 mL). The combined organic extracts were washed with brine, dried over Na_2SO_4, filtered, and concentrated *in*

vacuo to give the crude product as a yellow oil. The crude material was purified by flash column chromatography (pet. ether:Et_2O, 95:5 – 90:10) and concentrated *in vacuo* to provide the dimethyl 2-(3-((*tert*-butyldimethylsilyl)oxy)cyclopent-2-en-1-yl)-2-(prop-2-yn-1-yl)malonate **S30** (0.336 g, 0.92 mmol, **31%**) as a pale yellow oil.

^{1}H NMR ($CDCl_3$, 400 MHz): δ_H 4.66 (1H, dt, J = 1.9 Hz, ^4J = 1.9 Hz), 3.75 (3H, s), 3.73 (3H, s), 3.64 – 3.57 (1H, m), 2.80 (2H, ABqd, ^2J = 17.1 Hz, ^4J = 2.7 Hz), 2.33 – 2.19 (2H, m), 2.14 – 2.03 (1H, m), 2.00 (1H, t, ^4J = 2.7 Hz), 1.90 – 1.79 (1H, m) 0.92 (9H, s), 0.17 (3H, s) 0.16 (3H, s). **^{13}C NMR ($CDCl_3$, 101 MHz):** δ_C 169.9, 156.7, 101.9 79.1, 70.4, 60.2, 51.9, 45.2, 32.3, 25.1, 23.0, 22.1, 17.5, -5.1, -5.3. **IR (v_{max}/cm^{-1})**: 2953, 2930, 2857, 1757, 1732, 1643. **HRMS m/z (NSI/ion trap) Calc. for $C_{19}H_{31}O_5Si$ (M+H):** 367.1941; **found:** 367.1933.

Preparation of dimethyl 2-(3-((*tert*-butyldimethylsilyl)oxy)cyclohex-2-en-1-yl)-2-(prop-2-yn-1-yl)malonate.

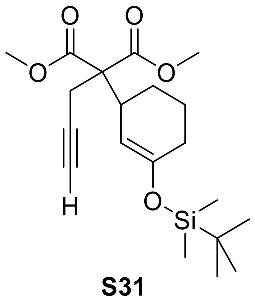

S31

2-Cyclohexen-1-one (0.39 mL, 4 mmol) was added to a flame-dried, round-bottom flask, dissolved in dry THF (20 mL) and cooled to -78 °C. TBSOTf (1.1 mL, 4.8 mmol) and dimethyl sulfide (0.89 mL, 12 mmol) were added sequentially and the reaction was stirred at -78 °C for 3 h. In a separate flame-dried, round-bottom flask NaH (0.18 g, 4.4 mmol) was suspended in dry THF (20 mL) and cooled to 0 °C then dimethyl 2-(prop-2-yn-1-yl)malonate **S29** (0.68 g, 4 mmol) was added and the mixture was stirred for 1 h at 0 °C. This mixture was then transferred to the 2-cyclohexen-1-one/TBSOTf/dimethyl sulfide mixture slowly and stirred at -78 °C for 2 h then warmed to room temperature. The reaction was quenched by addition of saturated aqueous $NaHCO_3$ solution, Et_2O was added to create a biphasic mixture, which was separated, and the aqueous layer was washed with Et_2O. The combined organic extracts were washed with brine, dried over Na_2SO_4, filtered, and concentrated *in vacuo* to give the crude product as a yellow oil. The crude material was purified by flash column chromatography (pet. ether:Et_2O, 90:10) and concentrated *in vacuo* to provide the dimethyl 2-(3-((*tert*-butyldimethylsilyl)oxy)cyclohex-2-en-1-yl)-2-(prop-2-yn-1-yl)malonate **S31** (0.551 g, 1.45 mmol, **36%**) as a pale yellow oil.

^{1}H NMR ($CDCl_3$, 400 MHz): δ_H 4.90 – 4.87 (1H, m), 3.76 (3H, s), 3.74 (3H, s), 3.23 – 3.15 (1H, m) 2.90 – 2.79 (2H, m), 2.09 – 2.89 (2H, m), 2.01 (1H, t, ^4J = 2.7 Hz), 1.88 – 1.76 (2H, m), 1.66 – 1.53 (1H, m), 1.36 – 1.25 (1H, m), 0.92 (9H, s), 0.15 (3H, s). 0.13 (3H, s). **^{13}C NMR ($CDCl_3$, 101 MHz):** δ_C 169.73, 169.68, 152.3, 103.8, 79.3, 70.5, 60.2, 51.97, 51.93, 38.5, 29.1, 25.2, 23.7, 22.0, 21.8, 17.5, -4.8, -5.1. **IR (v_{max}/cm^{-1})**: 2953, 2930, 2857, 1732, 1672. **HRMS m/z (NSI/ion trap) Calc. for $C_{20}H_{33}O_5Si$ (M+H):** 381.2092; **found:** 381.2092.

*The following experiments were carried out according to **Typical Procedure A**.*

Data are reported as: (a) carbonyl compound; (b) volume of DCE; (c) DIPEA; (d) silylating agent; (e) isolated yield; and (f) compound appearance. Individual analysis for each silyl enol ether compound is provided.

Preparation of dimethyl 2-(3-((*tert*-butyldimethylsilyl)oxy)allyl)-2-(prop-2-yn-1-yl)malonate.

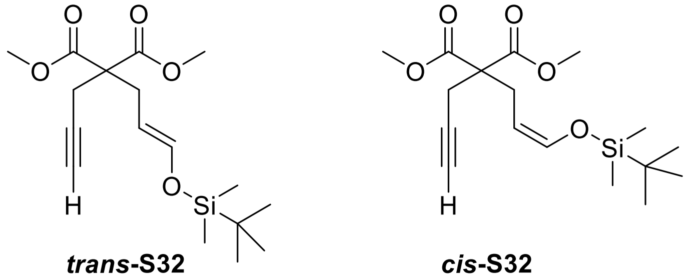

***trans*-S32** ***cis*-S32**

(a) Dimethyl 2-(3-oxopropyl)-2-(prop-2-yn-1-yl)malonate **S14**, 0.48 g, 2.13 mmol; (b) 21 mL; (c) DIPEA, 0.45 mL, 2.56 mmol; (d) TBSOTf, 0.59 mL, 2.56 mmol; (e) dimethyl-2-(3-((*tert*-butyldimethylsilyl)oxy)allyl)-2-(prop-2-yn-1-yl)malonate **S32**, 0.52 g, 1.53 mmol, **72%** as a *trans*:*cis* mixture, 67:33; and (f) colourless oil.

trans

^{1}H NMR (CDCl$_3$, 400 MHz): δ_H 6.35 (1H, dt, *J* = 11.9 Hz, ^4J = 1.1 Hz), 4.77 (1H, dt, *J* = 11.9 Hz, *J* = 8.1 Hz), 3.75 (6H, s), 2.83 – 2.80 (2H, d, ^4J = 2.4 Hz), 2.68 (2H, dd, *J* = 8.1 Hz, ^4J = 1.1 Hz), 2.02 (1H, t, ^4J = 2.4 Hz), 0.93 (9H, s), 0.14 (6H, s). **^{13}C NMR (CDCl$_3$, 101 MHz)**: δ_C 169.8, 143.6, 103.0, 78.4, 70.8, 56.8, 52.2, 30.1, 25.1, 22.0, 17.8, -5.8.

cis

^{1}H NMR (CDCl$_3$, 400 MHz): δ_H 6.34 (1H, dt, *J* = 6.1 Hz, ^4J = 1.4 Hz), 4.23 (1H, td, *J* = 7.5 Hz, *J* = 6.0 Hz), 3.75 (6H, s), 2.89 (2H, dd, *J* = 7.6 Hz, ^4J = 1.4 Hz), 2.80 (2H, t, ^4J = 2.7 Hz), 1.98 (1H, t, ^4J = 2.7 Hz), 0.94 (9H, s), 0.14 (6H, s). **^{13}C NMR (CDCl$_3$, 101 MHz)**: δ_C 170.1, 141.7, 101.8, 78.9, 70.4, 56.7, 52.2, 26.4, 25.1, 22.4, 17.7, -5.9.

IR (v_{max}/cm^{-1}): 3286, 2953, 2931, 1738, 1438, 1171. **HRMS m/z (ESI) Calc. for $C_{17}H_{28}O_5NaSi$ (M+Na)**: 363.1598; **found** 363.1598.

Preparation of dimethyl 2-(prop-2-yn-1-yl)-2-(3-((triethylsilyl)oxy)allyl)malonate.

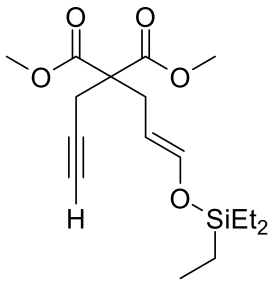

***trans*-S33**

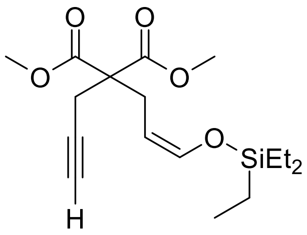

***cis*-S33**

(a) Dimethyl 2-(3-oxopropyl)-2-(prop-2-yn-1-yl)malonate **S14**, 0.59 g, 2.60 mmol; (b) 26 mL; (c) DIPEA, 0.54 mL, 3.12 mmol; (d) TESOTf, 0.67 mL, 3.12 mmol; (e) dimethyl 2-(prop-2-yn-1-yl)-2-(3-((triethylsilyl)oxy)allyl)malonate **S33**, 0.55 g, 1.62 mmol, **62%**, as a *trans*:*cis* mixture, 71:29; and (f) colourless oil.

trans

^{1}H NMR (CDCl$_3$, 400 MHz): δ_H 6.36 (1H, dt, *J* = 11.9 Hz, ^4J = 1.1 Hz), 4.77 (1H, dt, *J* = 11.8 Hz, *J* = 8.1 Hz), 3.75 (6H, s), 2.80 (2H, d, ^4J = 2.6 Hz), 2.68 (2H, dd, *J* = 8.1 Hz, ^4J = 1.1 Hz), 2.02 (1H, t, ^4J = 2.7 Hz), 0.98 (9H, t, *J* = 7.9 Hz), 0.67 (6H, q, *J* = 8.0 Hz). **^{13}C NMR (CDCl$_3$, 101 MHz)**: δ_C 169.8, 143.4, 102.9, 78.4, 70.8, 56.8, 52.2, 30.0, 21.9, 5.9, 3.9.

cis

^{1}H NMR (CDCl$_3$, 400 MHz): δ_H 6.35 (1H, dt, *J* = 5.7 Hz, ^4J = 1.5 Hz), 4.22 (1H, td, *J* = 7.6 Hz, *J* = 5.9 Hz), 3.74 (6H, s), 2.89 (2H, dd, *J* = 7.6 Hz, ^4J = 1.3 Hz), 2.80 (2H, d, ^4J = 2.1 Hz), 1.98 (1H, t, ^4J = 2.1 Hz), 0.99 (9H, t, *J* = 7.9 Hz), 0.67 (6H, q, *J* = 7.9 Hz). **^{13}C NMR (CDCl$_3$, 101 MHz)**: δ_C 170.1, 141.6, 101.7, 78.9, 70.4, 56.7, 52.1, 26.2, 22.3, 6.0, 3.9.

IR (ν_{max}/cm^{-1}): 3291, 2953, 2878, 1736, 1661, 1169. **HRMS m/z (ESI) Calc. for $C_{17}H_{28}O_5NaSi$ (M+Na)**: 363.1598; **found** 363.1597.

Preparation of *N*-(prop-2-yn-1-yl)-*N*-(3-((triethylsilyl)oxy)allyl)toluenesulfonamide.

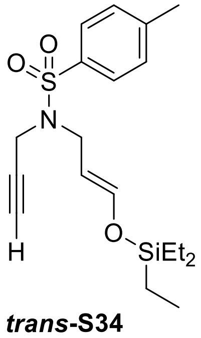

***trans*-S34**

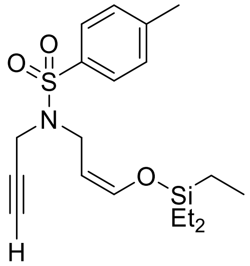

***cis*-S34**

(a) *N*-(3-Oxopropyl)-*N*-(prop-2-yn-1-yl)toluenesulfonamide **S17**, 0.63 g, 2.38 mmol; (b) 24 mL; (c) DIPEA, 0.46 mL, 2.62 mmol; (d) TESOTf, 0.56 mL, 2.62 mmol; (e) *N*-(prop-2-yn-1-yl)-*N*-(3-((triethylsilyl)oxy)allyl)toluenesulfonamide **S34** 0.65 g, 1.71 mmol, **72%** as a *trans*:*cis* mixture, 71:29; and (f) colourless oil.

trans

^{1}H NMR (CDCl$_3$, 400 MHz): δ$_H$ 7.74 – 7.72 (2H, m), 7.31 – 7.28 (2H, m), 6.44 (1H, dd, *J* = 12.0 Hz, ^4J = 1.0 Hz), 4.85 (1H, dt, *J* = 12.0 Hz, *J* = 7.8 Hz), 4.09 (2H, d, ^4J = 2.5 Hz), 3.74 (2H, dd, *J* = 7.8 Hz, ^4J = 0.8 Hz), 2.42 (3H, s), 2.01 (1H, t, ^4J = 2.5 Hz), 0.96 (9H, t, *J* = 7.9 Hz), 0.65 (6H, q, *J* = 7.9 Hz). **^{13}C NMR (CDCl$_3$, 101 MHz)**: δ$_C$ 145.9, 143.3, 136.0, 129.3, 127.6, 103.5, 76.5, 73.5, 44.4, 34.8, 21.4, 6.3, 4.2.

cis

^{1}H NMR (CDCl$_3$, 400 MHz): δ$_H$ 7.76 – 7.74 (2H, m), 7.30 – 7.27 (2H, m), 6.40 (1H, dt, *J* = 5.8 Hz, ^4J = 1.2 Hz), 4.43 (2H, td, *J* = 7.3 Hz, *J* = 5.8 Hz), 4.07 (2H, d, ^4J = 2.4 Hz), 3.97 (2H, dd, *J* = 7.3 Hz, ^4J = 1.1 Hz), 2.42 (3H, s), 1.99 (1H, t, ^4J = 2.4 Hz), 0.96 (9H, t, *J* = 7.9 Hz), 0.65 (6H, q, *J* = 7.9 Hz). **^{13}C NMR (CDCl$_3$, 101 MHz)**: δ$_C$ 143.3, 143.1, 136.5, 129.2, 127.6, 103.2, 77.3, 72.7, 40.3, 35.6, 21.4, 6.3, 4.2.

IR (v_{max}/cm^{-1}): 3271, 2955, 2911, 2876, 1659. **HRMS m/z (NSI) Calc. for $C_{19}H_{33}N_2O_3SSi$ (M+NH$_4$)**: 397.1976; **found**: 397.1977; **(M+Na) Calc. for $C_{19}H_{29}NNaO_3SSi$**: 402.1530; **found**: 402.1529.

Preparation of dimethyl 2-(4-((*tert*-butyldimethylsilyl)oxy)but-3-en-1-yl)-2-(prop-2-yn-1-yl)malonate.

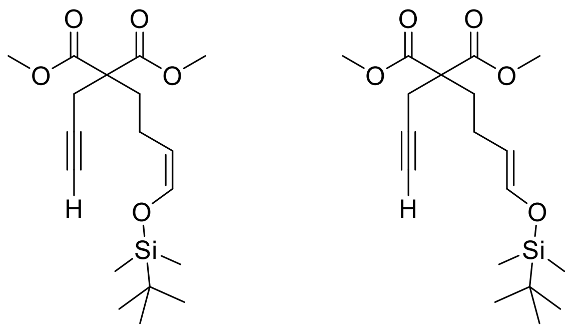

***cis*-S35** ***trans*-S35**

(a) Dimethyl 2-(4-oxobutyl)-2-(prop-2-yn-1-yl)malonate **S20**, 0.45 g, 1.87 mmol; (b) 19 mL; (c) DIPEA, 0.36 mL, 2.06 mmol; (d) TBSOTf, 0.46 mL, 2.06 mmol; (e) dimethyl 2-(4-((*tert*-butyldimethylsilyl)oxy)but-3-en-1-yl)-2-(prop-2-yn-1-yl)malonate **S35**, 0.58 g, 1.63 mmol, **87%**, as a *cis*:*trans* mixture, 92:8; and (f) colourless oil. To note, this reaction run deviates from the typical procedure whereby the reaction time was 2 h as opposed to the more standard 16 h.

cis

^{1}H NMR (CDCl$_3$, 400 MHz): δ$_H$ 6.20 (1H, dt, *J* = 5.8 Hz, *J* = 1.4 Hz), 4.42 (1H, td, *J* = 10.5 Hz, *J* = 5.9 Hz), 3.75 (6H, s), 2.89 (2H, d, ^4J = 2.7 Hz), 2.18 – 2.11 (2H, m), 2.06 – 1.99 (2H, m), 2.01 (1H, t, ^4J = 2.7 Hz), 0.94 (9H, s), 0.14 (6H, s). **^{13}C NMR (CDCl$_3$, 101 MHz)**: δ$_C$ 170.2, 138.8, 108.1, 78.5, 70.7, 56.4, 52.2, 31.2, 25.1, 22.2, 18.0, 17.8, - 5.9.

Note: ^{1}H and ^{13}C NMR chemical shift signals relating to the trans-*isomer were not sufficiently strong to fully assign.*

IR (v_{max}/cm^{-1}): 3292, 2953, 2930, 2857, 1736, 1655. **HRMS m/z (NSI) Calc. for $C_{18}H_{31}O_5Si$ (M+H)**: 355.1935; **found**: 355.1940.

c. Dicobalthexacarbonyl complexes

Typical Procedure B for the preparation of dicobalthexacarbonyl complexes.

Preparation of dimethyl 2-(2-((*tert*-butyldimethylsilyl)oxy)allyl)-2-(prop-2-yn-1-yl)malonate dicobalthexacarbonyl complex.

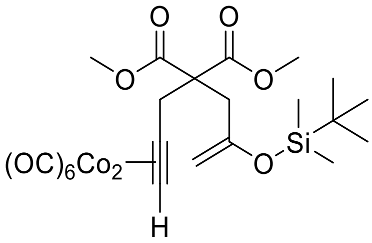

3

Dimethyl 2-(2-((*tert*-butyldimethylsilyl)oxy)allyl)-2-(prop-2-yn-1-yl)malonate **2** (0.34 g, 1.00 mmol) and petroleum ether (10 mL) were added to a flame-dried, round-bottom flask equipped with a stirrer bar. $Co_2(CO)_8$ (0.36 g, 1.05 mmol) was added and the mixture stirred at room temperature for 1 h. At this point, the reaction mixture was filtered through celite and concentrated *in vacuo* to provide the crude product as a red oil. The crude material was purified by flash column chromatography (pet. ether:Et_2O, 95:5 – 80:20) and concentrated *in vacuo* to provide the dicobalthexacarbonyl complex **3** (0.61 g, 0.98 mmol, **98%**) as a red gum.

^{1}H NMR ($CDCl_3$, 400 MHz): δ_H 5.97 (1H, t, ^4J = 0.8 Hz), 4.18 (1H, d, ^2J = 1.3 Hz), 4.15 (1H, d, ^2J = 1.3 Hz), 3.76 (6H, s), 3.73 (2H, d, ^4J = 0.8 Hz), 2.86 (2H, s), 0.94 (9H, s), 0.19 (6H, s). **^{13}C NMR ($CDCl_3$, 101 MHz)**: δ_C 199.2, 169.9, 154.2, 93.3, 87.8, 73.1, 57.0, 52.1, 39.9, 37.6, 25.4, 17.9, -5.0. **IR (ν_{max}/cm^{-1})**: 2953, 2931, 2856, 2092, 2050, 2000, 1738, 1628. **HRMS m/z (NSI) Calc. for $C_{23}H_{29}Co_2O_{11}Si$ (M+H)**: 627.0138; **found**: 627.0131.

The following experiments were carried out according to ***Typical Procedure B****.*

Data are reported as: (a) silyl enol ether; (b) volume of petroleum ether; (c) $Co_2(CO)_8$; (d) isolated yield; and (e) product appearance. Individual analysis for each dicobalthexacarbonyl compound is provided.

Preparation of dimethyl 2-(2-((triethylsilyl)oxy)allyl)-2-(prop-2-yn-1-yl)malonate dicobalthexacarbonyl complex.

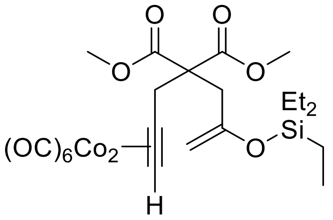

6a

(a) Dimethyl 2-(2-((triethylsilyl)oxy)allyl)-2-(prop-2-yn-1-yl)malonate **5a**, 0.85 g, 2.49 mmol; (b) 25 mL; (c) $Co_2(CO)_8$, 0.90 g, 2.62 mmol; (d) dicobalthexacarbonyl complex **6a**, 1.33 g, 2.12 mmol, **85%**; and (e) red oil.

^{1}H NMR (CDCl$_3$, 400 MHz): δ_H 5.98 (1H, s), 4.16 (1H, s), 4.14 (1H ,s), 3.75 (6H, s), 3.72 (2H, s), 2.87 (2H, s), 0.98 (9H, t, J = 7.9 Hz), 0.71 (6H, q, J = 7.9 Hz). **^{13}C NMR (CDCl$_3$, 101 MHz)**: δ_C 199.1, 170.0, 154.2, 92.2, 87.8, 73.1, 56.7, 52.0, 39.5, 37.1, 6.0, 4.0. **IR (ν_{max}/cm^{-1})**: 3107, 2953, 2914, 2879, 2092, 2050, 2032, 2011, 1993, 1732. **HRMS m/z (NSI) Calc. for $C_{23}H_{29}Co_2O_{11}Si$ (M+H)**: 627.0138; **found**: 627.0132.

Preparation of dimethyl 2-(prop-2-yn-1-yl)-2-(2-((triisopropylsilyl)oxy)allyl)malonate dicobalthexacarbonyl complex.

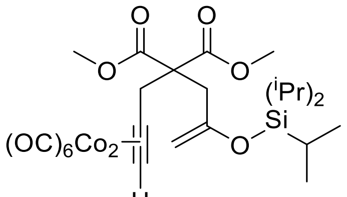

6b

(a) Dimethyl 2-(prop-2-yn-1-yl)-2-(2-((triisopropylsilyl)oxy)allyl)malonate **5b**, 0.09 g, 0.24 mmol; (b) 2.5 mL; (c) $Co_2(CO)_8$, 0.86 g, 0.25 mmol; (d) dicobalthexacarbonyl complex **6b**, 0.103 g, 015. mmol, **64%**; and (e) red oil.

^{1}H NMR (**400 MHz, CDCl$_3$**): 5.90 (1H, s), 4.13 (1H, s), 4.07 (1H, s), 3.70 (6H, s), 3.68 (2H, s), 2.81 (2H, s), 1.19 – 1.12 (3H, m), 1.04 (18H, d, J = 7.3 Hz). **^{13}C NMR** (**101 MHz, CDCl$_3$**): 199.2, 170.0, 154.3, 92.8, 88.0, 73.1, 57.3, 52.0, 40.5, 37.8, 17.4, 12.2. **IR (ν_{max}/cm^{-1})**: 2960, 2010, 1740, 1680, 1620. **HRMS *m*/*z*** (**ESI**): **Calc. for $C_{26}H_{35}Co_2O_{11}Si$ (M+H):** 669.0607; **found:** 669.0603.

Preparation of *N*-(prop-2-yn-1-yl)-*N*-(2-((*tert*-dimethylsilyl)oxy)allyl)toluenesulfonamide dicobalthexacarbonyl complex.

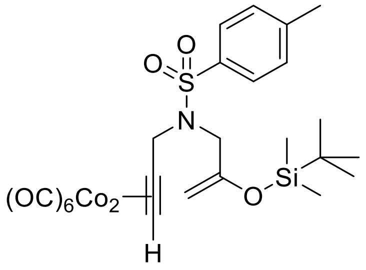

8a

(a) *N*-(Prop-2-yn-1-yl)-*N*-(2-((*tert*-butyldimethylsilyl)oxy)allyl)toluenesulfonamide **S21**, 0.21 g, 0.55 mmol; (b) 5.5 mL; (c) $Co_2(CO)_8$, 0.2 g, 0.58 mmol; (d) dicobalthexacarbonyl complex **8a**, 0.36 g, 0.54 mmol, **98%**; and (e) red oil.

^{1}H NMR (CDCl$_3$, 400 MHz): δ_H 7.79 – 7.76 (2H, m), 7.32 – 7.28 (2H, m), 6.00 (1H, s), 4.69 (2H, s), 4.18 (1H, d, ^2J = 1.7 Hz), 4.17 (1H, d, ^2J = 1.7 Hz), 3.96 (2H, s), 2.44 (3H, s), 0.88 (9H, s), 0.12 (6H, s). **^{13}C NMR (CDCl$_3$, 101 MHz)**: δ_C 199.2, 153.0, 143.2, 137.9, 129.5, 127.4, 93.2, 89.3, 73.8, 50.9, 49.6, 25.5, 21.5, 18.0, -4.9. **IR (ν_{max}/cm^{-1})**: 3077, 2095, 2052, 2000, 1952, 1732. **HRMS m/z (APCI) Calc. for $C_{25}H_{30}Co_2NO_9SSi$ (M+H)**: 666.0069; **found** 666.0075.

Preparation of *N*-(prop-2-yn-1-yl)-*N*-(2-((triethylsilyl)oxy)allyl)toluenesulfonamide dicobalthexacarbonyl complex.

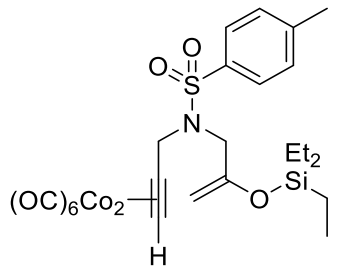

8b

(a) *N*-(Prop-2-yn-1-yl)-*N*-(2-((triethylsilyl)oxy)allyl)toluenesulfonamide **S22**, 0.50 g, 1.31 mmol; (b) 13 mL; (c) $Co_2(CO)_8$, 0.47 g, 1.37 mmol; (d) dicobalthexacarbonyl complex **8b**, 0.83 g, 1.24 mmol, **94%**; and (e) red oil.

^{1}H NMR ($CDCl_3$, 400 MHz): δ_H 7.80 – 7.74 (2H, m), 7.33 – 7.26 (2H, m), 6.00 (1H, s), 4.67 (2H, s), 4.17 (2H, d, *J* = 2.8 Hz), 3.98 (2H, s), 2.44 (3H, s), 0.93 (9H, t, *J* = 7.9 Hz), 0.63 (6H, q, *J* = 7.9 Hz). **^{13}C NMR ($CDCl_3$, 101 MHz)**: δ_C 198.7, 152.5, 142.7, 137.4, 129.0, 126.9, 92.2, 88.7, 73.3, 50.2, 48.6, 21.0, 6.1, 4.1. **IR (v_{max}/cm^{-1})**: 3102, 2955, 2935, 2912, 2875, 2098, 2052, 2036, 2019, 2002, 1622, 1160. **HRMS m/z Calc. for $C_{25}H_{30}Co_2NO_9SSi$ (M+H)**: 666.0069; **found** 666.0075.

Preparation of dimethyl 2-(but-2-yn-1-yl)-2-(2-((*tert*-butyldimethylsilyl)oxy)allyl)malonate dicobalthexacarbonyl complex.

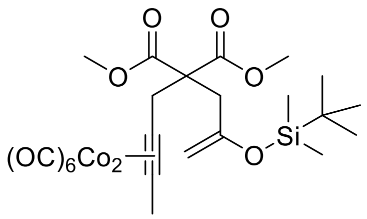

8c

(a) Dimethyl 2-(but-2-yn-1-yl)-2-(2-((*tert*-butyldimethylsilyl)oxy)allyl)malonate **S23**, 0.35 g, 0.99 mmol; (b) 10 mL; (c) $Co_2(CO)_8$, 0.35 g, 1.04 mmol; (d) dicobalthexacarbonyl complex **8c**, 0.59 g, 0.92 mmol, **93%**; and (e) red oil.

^{1}H NMR ($CDCl_3$, 400 MHz): δ_H 4.18 (1H, s), 4.14 (1H, s) 3.78 (6H, s), 3.77 (2H, s), 2.85 (2H, s), 2.70 (3H, s), 0.95 (9H, s), 0.19 (6H, s). **^{13}C NMR ($CDCl_3$, 101 MHz)**: δ_C 199.4, 170.0, 154.2, 93.6, 93.1, 89.5, 57.1, 52.1, 40.5, 38.7, 25.4, 21.5, 17.9, -5.1. **IR (v_{max}/cm^{-1})**: 2933, 2046, 2006, 1739. Note that, due to the sensitivity of this dicobalthexacarbonyl complex, HRMS analysis was not obtained for this compound; however, following the PKR of this substrate, the resultant cyclopentenone product **9c** was fully characterised.

Preparation of dimethyl 2-(but-2-yn-1-yl)-2-(2-((triethylsilyl)oxy)allyl)malonate dicobalthexacarbonyl complex.

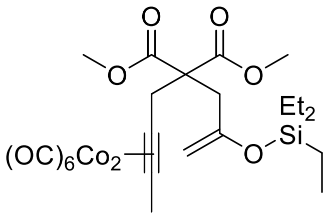

8d

(a) Dimethyl 2-(but-2-yn-1-yl)-2-(2-((triethylsilyl)oxy)allyl)malonate **S24**, 0.87 g, 2.46 mmol; (b) 25 mL; (c) $Co_2(CO)_8$, 0.93 g, 2.71 mmol; (d) dicobalthexacarbonyl complex **8d**, 1.33 g, 2.08 mmol, **84%**; and (e) red oil.

^{1}H NMR (CDCl$_3$, 400 MHz): δ_H 4.16 (1H, d, ^2J = 1.5 Hz), 4.13 (1H, d, ^2J = 1.5 Hz), 3.77 (6H, s), 3.75 (2H, s), 2.88 (2H, s), 2.71 (3H, s), 0.99 (9H, t, J = 7.9 Hz), 0.71 (6H, q, J = 8.0 Hz). **^{13}C NMR (CDCl$_3$, 101 MHz)**: δ_C 199.5, 170.1, 154.2, 93.5, 92.1, 89.5, 56.7, 52.0, 40.0, 38.3, 21.6, 6.1, 4.0. **IR (ν_{max}/cm^{-1})**: 2955, 2877, 2087, 2043, 1998, 1738, 1626, 1202. **HRMS m/z (NSI) Calc. for $C_{24}H_{31}Co_2O_{11}Si$ (M+H)**: 641.0294; **found**: 641.0281.

Preparation of *N*-(but-2-yn-1-yl)-*N*-(2-((triethylsilyl)oxy)allyl)toluenesulfonamide dicobalthexacarbonyl complex.

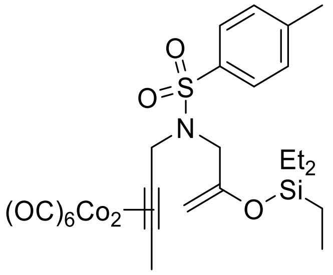

8e

(a) *N*-(But-2-yn-1-yl)-4-methyl-*N*-(2-((triethylsilyl)oxy)allyl)benzenesulfonamide **S25**, 620 mg, 1.57 mmol; (b) 16 mL; (c) dicobalt octacarbonyl, 567 mg, 1.65 mmol; (d) dicobalthexacarbonyl complex **8e**, 1.07 g, 1.57 mmol, **100%**; and (e) red oil.

^{1}H NMR (CDCl$_3$, 400 MHz): δ_H 7.79 – 7.76 (2H, m), 7.30 – 7.27 (2H, m), 4.71 (2H, s), 4.11 (2H, d, ^2J = 13.5 Hz), 4.00 (2H, s), 2.75 (3H, s), 2.44 (3H, s), 0.90 (9H, t, J = 7.7 Hz), 0.60 (6H, q, J = 7.7 Hz). **^{13}C NMR (CDCl$_3$, 101 MHz)**: δ_C 199.0, 152.5, 142.6, 137.4, 128.9, 127.0, 93.7, 91.8, 91.4, 50.2, 49.1, 21.0, 20.5, 6.0, 4.1. **IR (ν_{max}/cm^{-1})**: 2958, 2092, 2049, 2001, 1632, 1348, 1159. **HRMS m/z (NSI) Calc. for $C_{26}H_{32}Co_2NO_9SSi$ (M+H)**: 680.0226; **found**: 680.0223.

Preparation of ((3-(but-2-yn-1-yloxy)prop-1-en-2-yl)oxy)(*tert*-butyl)dimethylsilane dicobalthexacarbonyl complex.

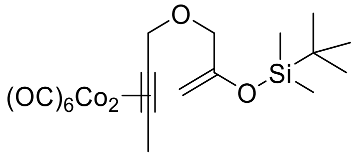

8f

(a) ((3-(But-2-yn-1-yloxy)prop-1-en-2-yl)oxy)(*tert*-butyl)dimethylsilane **S26**, 0.81 g, 3.38 mmol; (b) 34 mL; (c) $Co_2(CO)_8$, 1.2 g, 3.55 mmol; (d) dicobalthexacarbonyl complex **8f**, 1.78 g, 3.38 mmol, **100%**; and (e) red oil.

^{1}H NMR ($CDCl_3$, 400 MHz): δ_H 4.71 (2H, s), 4.44 – 4.42 (1H, m), 4.29 (1H, s), 3.98 (2H, s), 2.69 (3H, s), 0.96 (9H, s), 0.21 (6H, s). **^{13}C NMR ($CDCl_3$, 101 MHz)**: δ_C 199.1, 154.6, 90.5, 71.4, 70.1, 40.9, 25.1, 22.1, 19.9, 18.1, -5.2. **IR (ν_{max}/cm^{-1})**: 2955, 2930, 2859, 2089, 2046, 1992, 1632, 1250, 825. **HRMS m/z (APCI) Calc. for $C_{19}H_{24}Co_2O_8Si$ (M+H)**: 526.9983; **found**: 526.9988.

Preparation of dimethyl 2-(2-((*tert*-butyldimethylsilyl)oxy)allyl)-2-(3-phenylprop-2-yn-1-yl)malonate dicobalthexacarbonyl complex.

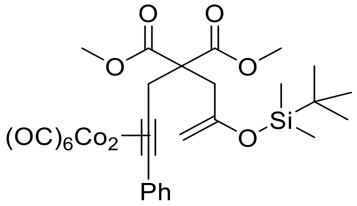

8g

(a) Dimethyl 2-(2-((*tert*-butyldimethylsilyl)oxy)allyl)-2-(3-phenylprop-2-yn-1-yl)malonate **S27**, 1.53 g, 3.67 mmol; (b) 36.5 mL; (c) $Co_2(CO)_8$, 1.32 g, 3.86 mmol; (d) dicobalthexacarbonyl complex **8g**, 2.57 g, 3.66 mmol, **100%**; and (e) red oil.

^{1}H NMR ($CDCl_3$, 400 MHz): δ_H 7.50 – 7.21 (5H, m), 4.13 (1H, s), 4.04 (1H, s), 3.87 (2H, s), 3.54 (6H, s), 2.90 (2H, s), 0.94 (9H, s), 0.18 (6H, s). **^{13}C NMR ($CDCl_3$, 101 MHz):** δ_C 198.8, 170.0, 154.3, 138.0, 128.5, 128.1 127.0, 94.3, 93.1, 90.9, 57.2, 51.8, 41.4, 38.0, 25.3, 17.8, -5.2. **IR (ν_{max}/cm^{-1}):** 2089, 2047, 1994, 1734, 1626. **HRMS m/z (NSI/ion trap) Calc. for $C_{29}H_{33}Co_2O_{11}Si$ (M+H):** 703.0456; **found** 703.0460.

Preparation of dimethyl 2-(2-((*tert*-butyldimethylsilyl)oxy)allyl)-2-(4-((*tert*-butyldimethylsilyl)oxy)but-2-yn-1-yl)malonate dicobalthexacarbonyl complex.

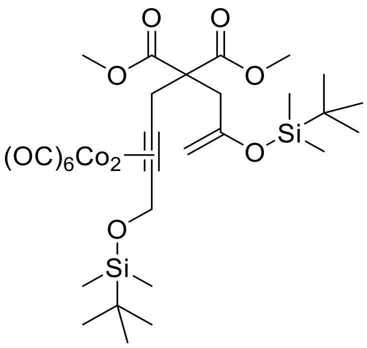

8h

(a) Dimethyl 2-(2-((*tert*-butyldimethylsilyl)oxy)allyl)-2-(4-((*tert*-butyldimethylsilyl)oxy)but-2-yn-1-yl)malonate **S28**, 0.62 g, 1.29 mmol; (b) 13 mL; (c) $Co_2(CO)_8$, 0.46 g, 1.35 mmol; (d) dicobalthexacarbonyl complex **8h**, 0.95 g, 1.23 mmol, **95%**; and (e) red oil.

^{1}H NMR ($CDCl_3$, 400 MHz): δ_H 4.76 (2H, s), 4.17 (1H, d, ^2J = 1.4 Hz), 4.14 (1H, d, ^2J = 1.4 Hz), 3.76 (6H, s), 3.74 (2H, s), 2.84 (2H, s), 0.96 (9H, s), 0.95 (9H, s), 0.19 (6H, s), 0.14 (6H, s). **^{13}C NMR ($CDCl_3$, 101 MHz):** δ_C 199.4, 170.0, 154.2, 99.0, 93.2, 87.2, 63.2, 57.1, 52.1, 40.3, 38.3, 25.4, 18.0, 17.9, -5.1, -6.1. **IR (ν_{max}/cm^{-1}):** 2953, 2930, 2859, 2089, 2045, 2010, 1740. **LRMS m/z (Electrospray) Calc. for $C_{28}H_{44}Co_2O_{10}Si_2$ (M-2CO):** 714.1; **found:** 714.1. Note that, due to the sensitivity of this dicobalthexacarbonyl complex, HRMS analysis was not obtained for this compound; however, following the PKR of this substrate, the resultant cyclopentenone product **9h** was fully characterised.

Preparation of dimethyl 2-(3-((*tert*-butyldimethylsilyl)oxy)cyclopent-2-en-1-yl)-2-(prop-2-yn-1-yl)malonate dicobalthexacarbonyl complex.

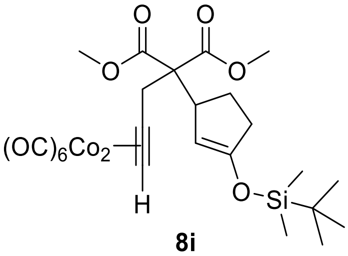

8i

(a) Dimethyl 2-(3-((*tert*-butyldimethylsilyl)oxy)cyclopent-2-en-1-yl)-2-(prop-2-yn-1-yl)malonate **S30**, 0.279 g, 0.76 mmol; (b) 7.5 mL; (c) $Co_2(CO)_8$, 0.274 g, 0.8 mmol; (d) dicobalthexacarbonyl complex **8i**, 0.494 g, 0.76 mmol, **100%**; and (e) red oil.

^{1}H NMR ($CDCl_3$, 400 MHz): δ_H 5.96 (1H, t, ^4J = 0.8 Hz), 4.67 (1H, dt, J = 1.7 Hz, ^4J = 1.7 Hz), 3.76 (3H, s), 3.75 (3H, s), 3.63 (2H, ABqd, ^2J = 16.7 Hz, ^4J = 0.8 Hz), 3.49 – 3.43 (1H, m) 2.33 – 2.17 (2H, m), 2.14 – 2.03 (1H, m), 1.90 – 1.79 (1H, m) 0.93 (9H, s), 0.18 (3H, s) 0.17 (3H, s). **^{13}C NMR ($CDCl_3$, 101 MHz):** δ_C 199.1, 170.12, 170.09, 156.8, 101.6, 87.9, 73.0, 61.9. 51.82, 51.76, 45.7, 38.2, 32.3, 25.1, 23.3, 17.6, -5.2, -5.4. **IR (ν_{max}/cm^{-1})**: 2953, 2932, 2897, 2857, 2091, 2048, 2002, 1730, 1643. **HRMS m/z (NSI/ion trap) Calc. for $C_{25}H_{31}Co_2O_{11}Si$ (M+H):** 653.0300; **found:** 653.0298.

Preparation of dimethyl 2-(3-((*tert*-butyldimethylsilyl)oxy)cyclohex-2-en-1-yl)-2-(prop-2-yn-1-yl)malonate dicobalthexacarbonyl complex.

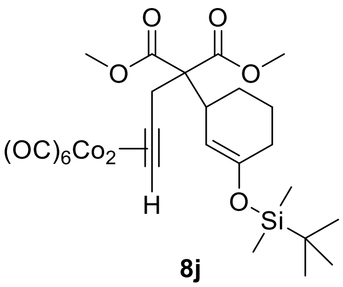

8j

(a) Dimethyl 2-(3-((*tert*-butyldimethylsilyl)oxy)cyclohex-2-en-1-yl)-2-(prop-2-yn-1-yl)malonate **S31**, 0.216 g, 0.57 mmol; (b) 5.7 mL; (c) $Co_2(CO)_8$, 0.205 g, 0.6 mmol; (d) dicobalthexacarbonyl complex **8j**, 0.35 g, 0.53 mmol, **93%**; and (e) red oil.

^{1}H NMR ($CDCl_3$, 400 MHz): δ_H 5.93 (1H, s), 4.92 (1H, s), 3.76 (3H, s), 3.73 (3H, s), 3.65 (2H, s), 3.03 – 3.01 (1H, m), 2.01 – 1.81 (4H, m), 1.38 – 1.22 (2H, s), 0.92 (9H, s), 0.15 (3H, s), 0.12 (3H, s). **^{13}C NMR ($CDCl_3$, 101 MHz):** δ_C 199.6, 170.3, 170.2, 152.8, 104.2, 88.5, 73.6, 62.6, 52.3, 52.2, 39.9, 38.6, 29.6, 25.6, 24.5, 22.1, 17.9, -4.3, -4.7. **IR (ν_{max}/cm^{-1})**: 2953, 2930, 2093, 2048, 1730.2, 1672 **HRMS m/z (NSI/ion trap) Calc. for $C_{26}H_{33}Co_2O_{11}Si$ (M+H):** 667.0451; **found:** 667.0446.

Preparation of dimethyl 2-(3-((*tert*-butyldimethylsilyl)oxy)allyl)-2-(prop-2-yn-1-yl)malonate dicobalthexacarbonyl complex.

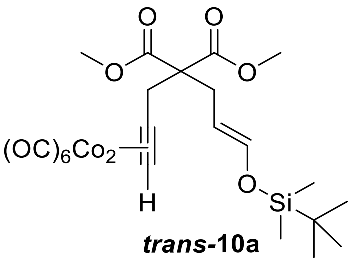

***trans*-10a**

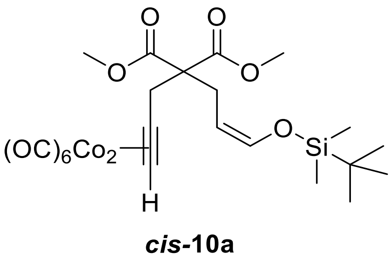

***cis*-10a**

(a) Dimethyl 2-(3-((*tert*-butyldimethylsilyl)oxy)allyl)-2-(prop-2-yn-1-yl)malonate **S32**, 0.47 g, 1.37 mmol, *trans*:*cis*, 67:33; (b) 13.7 mL; (c) $Co_2(CO)_8$, 0.49 g, 1.43 mmol; (d) dicobalthexacarbonyl complexes **10a**, 0.77 g, 1.23 mmol, **90%**, as a *trans*:*cis* mixture 67:33; and (e) red oil.

trans

^{1}H NMR ($CDCl_3$, 400 MHz): δ_H 6.32 (1H, apparent s), 5.96 (1H, s), 4.88 – 4.80 (1H, m), 3.75 (6H, s), 3.64 (2H, s), 2.62 (2H, d, J = 7.5 Hz), 0.92 (9H, s), 0.14 (6H, s).

cis

^{1}H NMR ($CDCl_3$, 400 MHz): δ_H 6.30 (1H, apparent s), 6.04 (1H, s), 4.33 – 4.27 (1H, m), 3.75 (6H, s), 3.64 (2H, s), 2.86 (2H, d, J = 7.0 Hz), 0.96 (9H, s), 0.16 (6H, s).

The following data refers to the mixture of isomers.

^{13}C NMR (CDCl$_3$, 101 MHz): δ_C 199.0, 170.4, 170.1, 143.4, 141.3, 103.1, 101.9, 87.8, 87.4, 73.2, 72.7, 58.7, 57.8, 52.1, 37.5, 37.1, 37.2, 30.7, 27.1, 25.1, 17.8, -5.8, -5.9. **IR (ν_{max}/cm^{-1})**: 2956, 2093, 2052, 2002, 1991, 1727, 1666, 1171. **HRMS (NSI) m/z Calc. for $C_{23}H_{29}Co_2O_{11}Si$ (M+H)**: 627.0138; **found** 627.0131.

Preparation of dimethyl 2-(3-((triethylsilyl)oxy)allyl)-2-(prop-2-yn-1-yl)malonate dicobalthexacarbonyl complex.

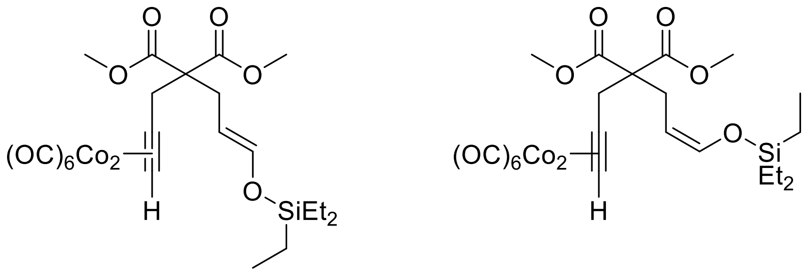

***trans*-10b** ***cis*-10b**

(a) Dimethyl 2-(3-((*tert*-butyldimethylsilyl)oxy)allyl)-2-(prop-2-yn-1-yl)malonate **S33**, 0.29 g, 0.84 mmol, *trans*:*cis*, 71:29; (b) 8.4 mL; (c) $Co_2(CO)_8$, 0.3 g, 0.88 mmol; (d) dicobalthexacarbonyl complexes **10b**, 0.38 g, 0.61 mmol, **72%**, as a *trans*:*cis* mixture, 68:32; and (e) red oil.

trans

^{1}H NMR (CDCl$_3$, 400 MHz): δ_H 6.38 – 6.34 (1H, m), 6.00 (1H, s), 4.89 (1H, dt, *J* = 11.8 Hz, *J* = 8.0 Hz), 3.80 (6H, s), 3.67 (2H, s), 2.66 (2H, d, *J* = 8.1 Hz), 1.02 (9H, t, *J* = 7.9 Hz), 0.71 (6H, q, *J* = 7.8 Hz).

cis

^{1}H NMR (CDCl$_3$, 400 MHz): δ_H 6.40 – 6.37 (1H, m), 6.10 (1H, s), 4.34 (1H, dt, *J* = 6.9 Hz, *J* = 7.2 Hz), 3.80 (6H, s), 3.69 (2H, s), 2.91 (2H, d, *J* = 7.2 Hz), 1.05 (9H, t, *J* = 7.9 Hz), 0.74 (6H, q, *J* = 7.8 Hz).

The following data refers to the mixture of isomers.

^{13}C NMR (CDCl$_3$, 101 MHz): δ_C 199.6, 170.9, 170.6,143.7, 141.7, 103.5, 102.4, 88.4, 87.9, 73.7, 73.2, 59.2, 58.3, 52, 38.0, 37.6, 31.3, 29.7, 27.6, 25.6, 6.5, 6.4, 4.42, 4.40. **IR (ν_{max}/cm^{-1})**: 2955, 2879, 2099, 2053, 2015, 1732, 1438, 1174. **HRMS (NSI) m/z Calc. for $C_{23}H_{32}Co_2NO_{11}Si$ (M+NH$_4$)**: 644.0403; **found** 644.0401.

Preparation of *N*-(prop-2-yn-1-yl)-*N*-(3-((triethylsilyl)oxy)allyl)toluenesulfonamide dicobalthexacarbonyl complex.

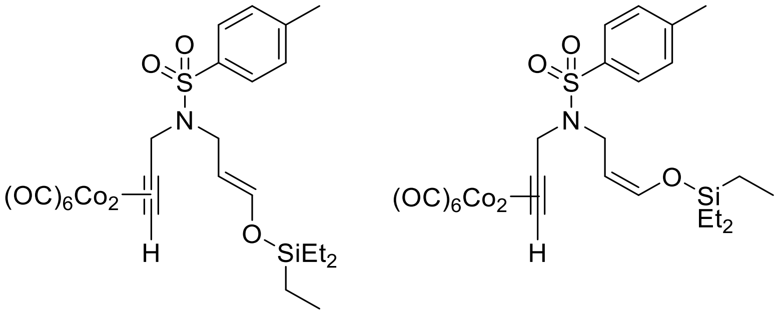

***trans*-10c** ***cis*-10c**

(a) *N*-(Prop-2-yn-1-yl)-*N*-(3-((triethylsilyl)oxy)allyl)toluenesulfonamide **S34**, 0.41 g, 1.07 mmol, *trans*:*cis,* 71:29; (b) 11 mL; (c) $Co_2(CO)_8$, 0.40 g, 1.12 mmol (d) dicobalthexacarbonyl complexes **10c**, 0.59 g, 0.88 mmol, **83%**, *trans*:*cis*, 70:30; and (e) red oil.

trans

^{1}H NMR (CDCl$_3$, 400 MHz): δ_H 7.76 – 7.73 (2H, m), 7.35 – 7.31 (2H, m), 6.36 (1H, dt, *J* = 11.9 Hz, ^4J = 0.9 Hz), 6.05 (1H, t, ^4J = 0.9 Hz), 4.64 (1H, dt, *J* = 12.0 Hz, *J* = 7.8 Hz), 4.49 (2H, s), 3.90 (2H, d, *J* = 7.6 Hz), 2.45 (3H, s), 0.94 (9H, t, *J* = 7.9 Hz), 0.61 (6H, q, *J* = 7.9 Hz).

cis

^{1}H NMR (CDCl$_3$, 400 MHz): δ_H 7.78 – 7.75 (2H, m), 7.36 – 7.31 (2H, m), 6.34 (1H, dt, *J* = 5.5 Hz, ^4J = 1.6 Hz), 6.11 (1H, t, ^4J = 1.0 Hz), 4.47 (2H, s), 4.27 – 4.21 (1H, m), 4.11 (2H, d, *J* = 7.4 Hz), 2.45 (3H, s), 0.97 (9H, t, *J* = 7.9 Hz), 0.67 (6H, q, *J* = 8.0 Hz).

The following data refers to the mixture of isomers.

^{13}C NMR (CDCl$_3$, 101 MHz): δ_C 198.8, 145.0, 142.9, 142.4, 137.3, 129.3, 129.2, 126.8, 126.7, 103.1, 102.6, 89.8, 72.9, 48.3, 47.0, 45.1, 41.1, 21.0, 5.89, 5.85, 3.80, 3.76. **IR (ν_{max}/cm^{-1})**: 2957, 2913, 2878, 2083, 2052, 2012, 2000. **HRMS m/z (NSI/ion trap) Calc. for $C_{25}H_{30}Co_2NO_9SSi$ (M+H)**: 666.0069; **found**: 666.0058.

Preparation of dimethyl 2-(4-((*tert*-butyldimethylsilyl)oxy)but-3-en-1-yl)-2-(prop-2-yn-1-yl)malonate dicobalthexacarbonyl complex.

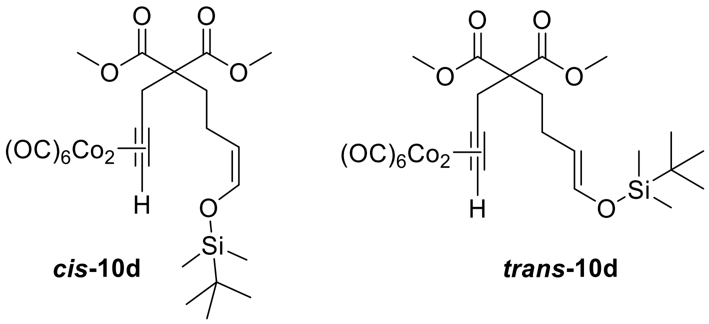

***cis*-10d** ***trans*-10d**

(a) Dimethyl 2-(4-((*tert*-butyldimethylsilyl)oxy)but-3-en-1-yl)-2-(prop-2-yn-1-yl)malonate **S35**, 0.54 g, 1.51 mmol, *cis*:*trans*: 92:8; (b) 15 mL; (c) $Co_2(CO)_8$, 0.54 g, 1.58 mmol; (d) dicobalthexacarbonyl complex **10d**, 0.97 g, 1.51 mmol, **100%**, *cis*:*trans*: 92:8; and (e) red oil.

cis

^{1}H NMR ($CDCl_3$, 400 MHz): δ_H 6.22 (1H, d, *J* = 5.7 Hz), 6.03 (1H, s), 4.44 – 4.39 (1H, m), 3.76 (6H, s), 3.71 (2H, s), 2.06 (2H, s), 2.05 (2H, s), 0.94 (9H, s), 0.16 (6H, s). **^{13}C NMR ($CDCl_3$, 101 MHz)**: δ_C 199.2, 170.4, 139.0, 107.5, 72.9, 57.7, 52.1, 37.1, 31.9, 25.1, 18.3, 17.8, - 5.9.

Signals for the *trans*-isomer were not sufficiently strong to fully assign.

IR (v_{max}/cm^{-1}): 2951, 2091, 2050, 1998, 1971, 1736, 1657. Note that, due to the sensitivity of these dicobalthexacarbonyl complexes, HRMS analysis was not obtained for these compounds; however, following the PKR of these substrates, the resultant cyclopentenone product **11d** was fully characterised.

III Pauson-Khand reactions

Typical Procedure C for Pauson-Khand reactions

Preparation of dimethyl 3*a*-((*tert*-butyldimethylsilyl)oxy)-5-oxo-3,3*a*,4,5-tetrahydropentalene-2,2(1*H*)-dicarboxylate.

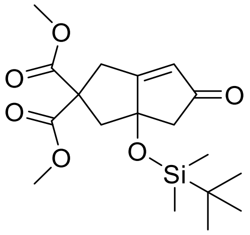

4

Dimethyl 2-(2-((*tert*-butyldimethylsilyl)oxy)allyl)-2-(prop-2-yn-1-yl)malonate dicobalthexacarbonyl complex **3** and distilled DCE (0.1 M) were added to a flame-dried, round-bottom flask equipped with a stirrer bar. The additive was added and the mixture was heated to the decided reaction temperature and for the allotted time. At this point, the reaction mixture was filtered through celite and the solvent was removed *in vacuo* to provide the crude material. The crude material was purified by flash column chromatography (pet. ether:Et_2O, 90:10) and concentrated *in vacuo* to provide dimethyl 3*a*-((*tert*-butyldimethylsilyl)oxy)-5-oxo-3,3*a*,4,5-tetrahydropentalene-2,2(1*H*)-dicarboxylate **4** as a white solid.

The following experiments were carried out according to ***Typical Procedure C.***

Data are reported as: (a) amount of dicobalthexacarbonyl complex; (b) volume of DCE; (c) additive; (d) reaction temperature; (e) reaction time; (f) isolated yield; and (g) product appearance. Individual analysis for each cyclopentenone product is provided.

Table 1

Entry 1
(a) 0.10 g, 0.16 mmol; (b) 2.6 mL; (c) TMANO.2H_2O, 0.12 g, 1.09 mmol; (d) rt; (e) 16 h; (f) 0.018 g, 0.049 mmol, **31%**; and (g) white solid.

Entry 2
(a) 0.10 g, 0.16 mmol; (b) 1.6 mL; (c) DodSMe, 0.2 mL, 0.76 mmol; (d) reflux; (e) 16 h; (f) 0.05 g, 0.125 mmol, **78%**; and (g) white solid.

Entry 3
(a) 0.10 g, 0.16 mmol; (b) 1.6 mL; (c) DodSMe, 0.2 mL, 0.76 mmol; (d) reflux; (e) 2 h; (f) 0.038 g, 0.1 mmol, **63%**; and (g) white solid.

Entry 4
(a) 2.50 g, 3.99 mmol; (b) 40 mL; (c) DodSMe, 5.03 mL, 18.95 mmol; (d) 70 °C; (e) 16 h; (f) 1.29 g, 3.5 mmol, **88%**; and (g) white solid.

Entry 5

(a) 0.96 g, 1.54 mmol; (b) 15.4 mL; (c) $CyNH_2$, 0.62 mL, 5.39 mmol; (d) 70 °C; (e) 16 h; (f) 0.01 g, 0.03 mmol, **2%**; and (g) white solid. Note that starting cobalt complex **3** was returned (0.22 g, 0.35 mmol, **23%**).

Entry 6

(a) 0.97 g, 1.55 mmol; (b) 15.5 mL; (c) TMTU, 0.97 g, 7.36 mmol; (d) 16 h; (e) 0.12 g, 0.32 mmol, **21%**; and (g) white solid. Note that starting cobalt complex **3** was returned (0.28 g, 0.45 mmol, **29%**).

Entry 7

(a) 0.1 g, 0.29 mmol; (b) 2.9 mL; (c) none; (d) 70 °C; (e) 16 h; (f) 0.019 g, 0.05 mmol, **18%**; and (g) white solid.

^{1}H NMR ($CDCl_3$, 400 MHz): δ_H 5.93 (1H, d, ^4J = 1.5 Hz), 3.80 (3H, s), 3.79 (1H, dd, ^2J = 18.0 Hz, ^4J = 2.0 Hz), 3.76 (3H, s), 3.07 (1H, d, ^2J = 18.0 Hz), 2.99 (1H, d, ^2J = 14.0 Hz), 2.62 (1H, d, ^2J = 18.2 Hz), 2.52 (1H, d, ^2J = 18.2 Hz), 2.32 (1H, d, ^2J = 14.0 Hz), 0.83 (9H, s), 0.05 (3H, s), 0.02 (3H, s). **^{13}C NMR ($CDCl_3$, 101 MHz)**: δ_C 206.6, 181.7, 171.5, 170.4, 125.5, 85.0, 60.0, 52.8, 52.7, 47.5, 46.4, 33.5, 25.0, 17.4, -3.6, -4.0. **IR (ν_{max}/cm^{-1})**: 2953, 2931, 2856, 1722, 1650, 1256, 1062. **HRMS m/z (NSI) Calc. for $C_{18}H_{32}NO_6Si$ (M+NH$_4$)**: 386.1993; **found**: 386.1994.

Preparation of dimethyl 5-oxo-3*a*-((triethylsilyl)oxy)-3,3*a*,4,5-tetrahydropentalene-2,2(1*H*)-dicarboxylate.

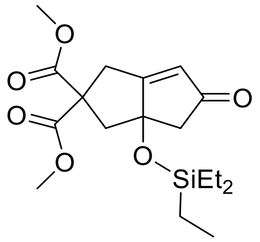

7a

(a) Dimethyl 2-(2-((triethylsilyl)oxy)allyl)-2-(prop-2-yn-1-yl)malonate dicobalthexacarbonyl complex **6a**, 1.28 g, 2.04 mmol; (b) 20 mL; (c) DodSMe, 2.57 mL, 9.69 mmol; (d) 70 °C; (e) 16 h; (f) dimethyl 5-oxo-3*a*-((triethylsilyl)oxy)-3,3*a*,4,5-tetrahydropentalene-2,2(1*H*)-dicarboxylate **7a**, 0.58 g, 1.58 mmol, **77%**, and (g) colourless oil.

^{1}H NMR ($CDCl_3$, 400 MHz): δ_H 5.91 (1H, dd, ^4J = 2.2 Hz, ^4J = 1.0 Hz), 3.81 (1H, dd, ^2J = 18.1 Hz, ^4J = 2.2 Hz), 3.81 (3H, s), 3.75 (3H, s), 3.07 (1H, d, ^2J = 18.2 Hz), 3.02 (1H, d, ^2J = 13.8 Hz), 2.62 (1H, d, ^2J = 18.2 Hz), 2.51 (1H, d, ^2J = 18.2 Hz), 2.24 (1H, d, ^2J = 13.8 Hz), 0.90 (9H, t, J = 7.9 Hz), 0.55 (6H, q, J = 7.8 Hz). **^{13}C NMR ($CDCl_3$, 101 MHz)**: δ_C 206.5, 182.3, 171.4, 170.5, 124.9, 84.7, 60.1, 52.8, 52.5, 46.9, 46.3, 33.4, 6.2, 5.4. **IR (ν_{max}/cm^{-1})**: 2955, 2914, 2877, 1736, 1718, 1650, 1246, 1062. **HRMS m/z (NSI) Calc. for $C_{18}H_{28}NaO_6Si$ (M+Na)**: 391.1547; **found**: 391.1545.

Preparation of dimethyl 5-oxo-3*a*-((tri*iso*propylsilyl)oxy)-3,3*a*,4,5-tetrahydropentalene-2,2(1*H*)-dicarboxylate.

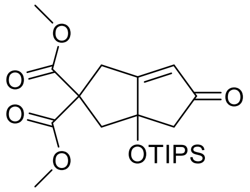

7b

(a) Dimethyl 2-(prop-2-yn-1-yl)-2-(2-((triisopropylsilyl)oxy)allyl)malonate dicobalthexacarbonyl complex **6b**, 0.62 g, 0.92 mmol; (b) 9 mL; (c) DodSMe, 1.2 mL, 4.37 mmol; (d) 70 °C; (e) 16 h; (f) no product obtained; and (g) N/A.

3a-((*tert*-Butyldimethylsilyl)oxy)-2-tosyl-2,3,3a,4-tetrahydrocyclopenta[*c*]pyrrol-5(1*H*)-one.

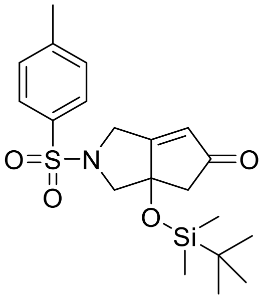

9a

(a) *N*-(2-((*Tert*-butyldimethylsilyl)oxy)allyl)-4-methyl-*N*-(prop-2-yn-1-yl)benzenesulfonamide dicobalthexacarbonyl complex **8a**, 880 mg, 1.40 mmol; (b) 14 mL; (c) DodSMe, 1.77 mL, 6.67 mmol; (d) 70 °C; (e) 2 h; (f) 3a-((*tert*-butyldimethylsilyl)oxy)-2-tosyl-2,3,3a,4-tetrahydrocyclopenta[*c*]pyrrol-5(1*H*)-one **9a**, 0.13 g, 0.32 mmol, **78%**; and (g) off-white solid.

^{1}H NMR ($CDCl_3$, 400 MHz): δ_H 7.76 (2H, d, *J* = 8.3 Hz), 7.36 (2H, d, *J* = 8.4 Hz), 5.96 (1H, dd, ^{4}J = 2.0 Hz, ^{4}J = 1.2 Hz), 4.36 (1H, dd, ^{2}J = 15.6 Hz, ^{4}J = 2.0 Hz), 4.18 (1H, d, ^{2}J = 15.8 Hz), 3.93 (1H, d, ^{2}J = 10.3 Hz), 3.06 (1H, d, ^{2}J = 10.3 Hz), 2.57 (1H, d, ^{2}J = 17.8 Hz), 2.45 (3H, s), 2.47 (1H, d, ^{2}J = 17.8 Hz), 0.81 (9H, s), 0.11 (3H, s), 0.02 (3H, s). **^{13}C NMR ($CDCl_3$, 101 MHz)**: δ_C 204.9, 175.0, 143.4, 133.9, 129.4, 126.9, 125.8, 82.7, 58.5, 46.3, 45.7, 24.9, 21.0, 17.4, -3.8. **IR (ν_{max}/cm^{-1})**: 2928, 1723, 1664, 1335, 1069. **HRMS m/z Calc. for $C_{20}H_{30}NO_4SSi$ (M+H)**: 408.1659; **found** 408.1657. **Melting point**: 112-114 °C.

Preparation of 2-tosyl-3*a*-((triethylsilyl)oxy)-2,3,3*a*,4-tetrahydrocyclopenta[c]pyrrol-5(1*H*)-one

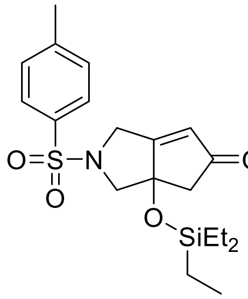

9b

(a) *N*-(Prop-2-yn-1-yl)-*N*-(2-((triethylsilyl)oxy)allyl)toluenesulfonamide dicobalthexacarbonyl complex **8b**, 0.67 g, 1.01 mmol; (b) 10 mL; (c) DodSMe,1.27 mL, 4.78 mmol; (d) 70 °C; (e) 16 h; (f) 2-tosyl-3*a*-((triethylsilyl)oxy)-2,3,3*a*,4-tetrahydrocyclopenta[c]pyrrol-5(1*H*)-one **9b**, 0.35 g, 0.86 mmol, **85%**; and (g) a white oil.

^{1}H NMR (CDCl$_3$, 400 MHz): δ_H 7.74 (2H, d, *J* = 8.2 Hz), 7.34 (2H, d, *J* = 8.2 Hz), 5.95 – 5.92 (1H, m), 4.33 (1H, dd, ^2J = 15.6 Hz, ^4J = 1.8 Hz), 4.17 (1H, d, ^2J = 15.6 Hz), 3.91 (1H, d, *J* = 10.5 Hz), 3.05 (1H, d, ^2J = 10.5 Hz), 2.55 (1H, d, ^2J = 17.9 Hz), 2.44 (3H, s), 2.42 (1H, d, *J* = 17.9 Hz), 0.87 (9H, t, *J* = 7.9 Hz), 0.59 – 0.42 (6H, m). **^{13}C NMR (CDCl$_3$, 101 MHz)**: δ_C 205.0, 175.3, 143.4, 133.7, 129.4, 126.9, 125.6, 82.5, 58.7, 46.3, 45.7, 21.0, 6.3, 5.3. **IR (ν_{max}/cm^{-1})**: 2955, 2912, 2875, 1722, 1600, 1347, 1164. **HRMS m/z (NSI) Calc. for $C_{20}H_{30}NO_4SSi$ (M+H)**: 408.1659; **found** 408.1656.

Preparation of dimethyl 6-methyl-5-oxo-3*a*-((*tert*-butyldimethylsilyl)oxy)-3,3*a*,4,5-tetrahydropentalene-2,2(1*H*)-dicarboxylate.

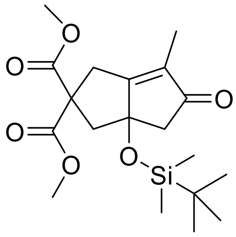

9c

(a) Dimethyl 2-(but-2-yn-1-yl)-2-(2-((*tert*-butyldimethylsilyl)oxy)allyl)malonate dicobalthexacarbonyl complex **8c**, 0.42 g, 0.65 mmol; (b) 6.5 mL; (c) DodSMe, 0.88 mL, 3.12 mmol; (d) 70 °C; (e) 16 h; (e) 6-methyl-5-oxo-3*a*-((*tert*-butyldimethylsilyl)oxy)-3,3*a*,4,5-tetrahydropentalene-2,2(1*H*)-dicarboxylate **9c**, 0.23 g, 0.60 mmol, **93%**; and (g) yellow oil.

^{1}H NMR (CDCl$_3$, 400 MHz): δ_H 3.80 (3H, s), 3.75 (3H, s), 3.68 (1H, dq, ^2J = 18.1 Hz, ^5J = 1.8 Hz), 3.00 (1H, d, *J* = 13.8 Hz), 2.97 (1H, d, *J* = 18.1 Hz), 2.63 (1H, d, ^2J = 18.3 Hz), 2.50 (1H, d, *J* = 18.3 Hz), 2.24 (1H, d, *J* = 13.8 Hz), 1.75 (3H, d, ^5J = 1.5 Hz), 0.81 (9H, s), 0.00 (3H, s), -0.03 (3H, s). **^{13}C NMR (CDCl$_3$, 101 MHz)**: δ_C 206.8, 174.3, 171.8, 170.6, 133.4, 83.3, 60.1, 52.8, 52.7, 47.2, 46.6, 32.5, 25.0, 17.4, 8.0, -3.6, -3.9. **IR (ν_{max}/cm^{-1})**: 2953, 2877, 1738, 1718, 1683, 1255, 1074. **HRMS m/z (NSI) Calc. for $C_{19}H_{30}NaO_6Si$ (M+Na)**: 405.1704; **found**: 405.1704.

Preparation of dimethyl 6-methyl-5-oxo-3*a*-((triethylsilyl)oxy)-3,3*a*,4,5-tetrahydropentalene-2,2(1*H*)-dicarboxylate.

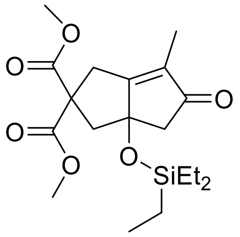

9d

(a) Dimethyl 2-(but-2-yn-1-yl)-2-(2-((triethylsilyl)oxy)allyl)malonate dicobalthexacarbonyl complex **8d**, 1.60 g, 2.50 mmol; (b) 25 mL; (c) DodSMe, 3.15 mL, 11.87 mmol; (d) 70 °C; (e) 16 h; (f) dimethyl 6-methyl-5-oxo-3*a*-((triethylsilyl)oxy)-3,3*a*,4,5-tetrahydropentalene-2,2(1*H*)-dicarboxylate **9d**, 0.82 g, 2.14 mmol, **89%**; and (g) yellow oil.

^{1}H NMR ($CDCl_3$, 400 MHz): δ_H 3.76 (3H, s), 3.71 (3H, s), 3.69 (1H, dd, ^2J = 18.2 Hz, ^5J = 1.7 Hz), 2.97 (1H, d, ^2J = 13.4 Hz), 2.94 (1H, d, ^2J = 18.1 Hz), 2.58 (1H, d, ^2J = 18.1 Hz), 2.45 (1H, d, ^2J = 18.2 Hz), 2.13 (1H, d, ^2J = 13.8 Hz), 1.69 (3H, d, ^5J = 1.3 Hz), 0.84 (9H, t, J = 7.9 Hz), 0.47 (6H, q, J = 7.8 Hz). **^{13}C NMR ($CDCl_3$, 101 MHz)**: δ_C 206.5, 174.9, 171.6, 170.6, 132.8, 83.0, 60.2, 52.7, 52.4, 46.6, 46.5, 32.4, 7.9, 6.2, 5.3. **IR (ν_{max}/cm^{-1})**: 2953, 2877, 1738, 1718, 1683, 1255, 1074. **HRMS m/z (NSI) Calc. for $C_{19}H_{31}O_6Si$ (M+H)**: 383.1884; **found**: 383.1886.

Preparation of 6-methyl-2-tosyl-3*a*-((triethylsilyl)oxy)-2,3,3*a*,4-tetrahydrocyclopenta[c]pyrrol-5(1*H*)-one.

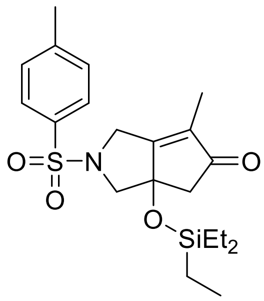

9e

(a) *N*-(But-2-yn-1-yl)-4-methyl-*N*-(2-((triethylsilyl)oxy)allyl)benzenesulfonamide dicobalthexacarbonyl complex **8e**, 1.00 g, 1.48 mmol; (b) 15 mL; (c) DodSMe, 1.86 mL, 7.02 mmol; (d) 70 °C; (e) 16 h; (f) 6-methyl-2-tosyl-3a-((triethylsilyl)oxy)-2,3,3a,4-tetrahydrocyclopenta[*c*]pyrrol-5(1*H*)-one **9e**, 0.56 g, 1.33 mmol, **89%**; and (g) white solid.

^{1}H NMR ($CDCl_3$, 400 MHz): δ_H 7.77 – 7.74 (2H, m), 7.35 – 7.32 (2H, m), 4.24 (1H, dd, ^2J = 15.1 Hz, ^5J = 1.2 Hz), 4.13 (1H, d, ^2J = 15.0 Hz), 3.89 (1H, d, ^2J = 10.4 Hz), 3.03 (1H, d, ^2J = 10.4 Hz), 2.58 (1H, d, ^2J = 18.0 Hz), 2.45 (3H, s), 2.43 (1H, d, ^2J = 18.0 Hz), 1.70 (3H, d, ^5J = 0.9 Hz), 0.85 (9H, t, J = 7.9 Hz), 0.55 – 0.39 (6H, m). **^{13}C NMR ($CDCl_3$, 101 MHz)**: δ_C 205.3, 167.9, 143.3, 134.1, 133.9, 129.3, 126.9, 80.8, 59.1, 46.0, 45.0, 21.0, 8.2, 6.3, 5.3. **IR (ν_{max}/cm^{-1})**: 2956, 1727, 1696, 1349, 1167. **HRMS m/z (NSI) Calc. for $C_{21}H_{32}NO_4SSi$ (M+H)**: 422.1816; **found** 422.1813. **Melting point**: 60 – 62 °C.

Preparation of 3*a*-((*tert*-butyldimethylsilyl)oxy)-6-methyl-3*a*,4-dihydro-1*H*-cyclopenta[c]furan-5(3*H*)-one.

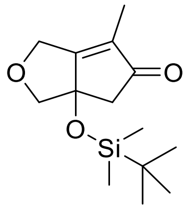

9f

(a) ((3-(But-2-yn-1-yloxy)prop-1-en-2-yl)oxy)(*tert*-butyl)dimethylsilane dicobalthexacarbonyl complex **8f**, 1.011 g, 1.92 mmol; (b) 12 mL; (c) DodSMe 2.42 mL, 9.12 mmol; (d) 70 °C; (e) 48 h; (f) 3*a*-((*tert*-butyldimethylsilyl)oxy)-6-methyl-3*a*,4-dihydro-1*H*-cyclopenta[c]furan-5(3*H*)-one **9f**, 0.305 g, 1.14 mmol, **59%**; and (g) white solid.

^{1}H NMR (CDCl$_3$, 400 MHz): δ_H 4.61 (1H, dd, ^2J = 15.0 Hz, ^5J = 1.3 Hz), 4.47 (1H, d, ^2J = 15.0 Hz), 4.15 (1H, d, ^2J = 9.4 Hz), 3.44 (1H, d, ^2J = 9.4 Hz), 2.55 (1H, d, ^2J = 17.8 Hz), 2.43 (1H, d, ^2J = 17.8 Hz), 1.73 (3H, t, ^5J = 1.3 Hz), 0.82 (9H, s), 0.03 (3H, s), 0.00 (3H, s). **^{13}C NMR (CDCl$_3$, 101 MHz)**: δ_C 206.8, 172.3, 133.1, 81.9, 76.8, 63.4, 45.6, 25.0, 17.5, 8.5, -3.7, -3.8. **IR (v_{max}/cm^{-1})**: 2955, 2926, 2855, 1713, 1682, 1082, 1001, 833. **HRMS m/z (NSI) Calc. for $C_{14}H_{25}O_3Si$ (M+H)**: 269.1573; **found** 269.1580. **Melting point**: 62 – 64 °C.

Preparation of dimethyl 3a-((*tert*-butyldimethylsilyl)oxy)-5-oxo-6-phenyl-3,3a,4,5-tetrahydropentalene-2,2(1*H*)-dicarboxylate.

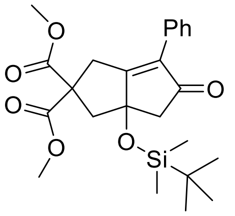

9g

(a) Dimethyl 2-(2-((*tert*-butyldimethylsilyl)oxy)allyl)-2-(3-phenylprop-2-yn-1-yl)malonate dicobalthexacarbonyl complex **8g**, 0.98 g, 1.4 mmol; (b) 14 mL; (c) DodSMe, 1.76 mL, 6.65 mmol; (d) 70 °C; (e) 16 h; (c) dimethyl 3a-((*tert*-butyldimethylsilyl)oxy)-5-oxo-6-phenyl-3,3a,4,5-tetrahydropentalene-2,2(1*H*)-dicarboxylate, **9g**, 0.145 g, 0.33 mmol, **24%**; and (g) colourless oil.

^{1}H NMR (CDCl$_3$, 400 MHz): δ_H 7.40 – 7.22 (5H, m), 4.09 (1H, d, ^2J = 18.8 Hz), 3.82 (3H, s), 3.70 (3H, s), 3.10 (1H, d, ^2J = 18.8 Hz), 3.07 (1H, d, ^2J = 14.2 Hz), 2.76 (2H, ABq, ^2J = 18.2 Hz), 2.36 (1H, d, ^2J = 14.2 Hz), 0.85 (9H, s), 0.05 (3H, s), 0.02 (3H, s). **^{13}C NMR (CDCl$_3$, 101 MHz):** δ_C 204.8, 174.8, 171.4, 170.5, 135.1, 129.7, 128.4, 128.3, 128.1, 83.3, 60.7, 52.8, 52.7, 48.4, 46.4, 34.3, 25.1, 17.4, -3.5, -3.8. **IR (v_{max}/cm^{-1}):** 2951, 2928, 2855, 1734, 1713. **HRMS m/z (NSI/ion trap) Calc. for $C_{24}H_{33}O_6Si$ (M+H):** 445.2038; **found** 445.2041.

Preparation of dimethyl 3a-((*tert*-butyldimethylsilyl)oxy)-6-(((*tert*-butyldimethylsilyl)oxy)methyl)-5-oxo-3,3a,4,5-tetrahydropentalene-2,2(1*H*)-dicarboxylate.

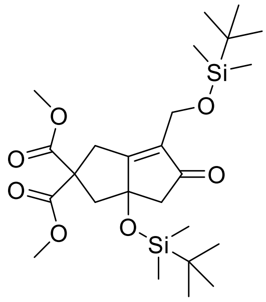

9h

(a) Dimethyl 2-(2-((*tert*-butyldimethylsilyl)oxy)allyl)-2-(4-((*tert*-butyldimethylsilyl)oxy)but-2-yn-1-yl)malonate dicobalthexacarbonyl complex **8h**, 0.72 g, 0.93 mmol; (b) 9.3 mL; (c) DodSMe, 1.17 mL, 4.41 mmol; (d) 70 °C; (e) 16 h; (f) dimethyl 3a-((*tert*-butyldimethylsilyl)oxy)-6-(((*tert*-butyldimethylsilyl)oxy)methyl)-5-oxo-3,3a,4,5-tetrahydropentalene-2,2(1*H*)-dicarboxylate, **9h**, 0.42 g, 0.82 mmol, **88%**; and (g) colourless oil.

^{1}H NMR ($CDCl_3$, 400 MHz): δ_H 4.43 (2H, ABqd, ^2J = 15.1 Hz, ^5J = 2.2 Hz), 3.79 (1H, dt, ^2J = 18.4 Hz, ^5J = 2.2 Hz), 3.79 (3H, s), 3.74 (3H, s), 3.33 (1H, d, ^2J = 18.7 Hz), 2.95 (1H, d, ^2J = 13.9 Hz), 2.56 (2H, ABq, ^2J = 18.3 Hz), 2.30 (1H, d, ^2J = 13.9 Hz), 0.92 (9H, s), 0.82 (9H, s), 0.11 (3H, s), 0.10 (3H, s), 0.01 (3H, s), 0.00 (3H, s). **^{13}C NMR ($CDCl_3$, 101 MHz):** δ_C 205.4, 175.7, 171.7, 170.6, 136.1, 83.9, 60.5, 57.1, 52.7, 52.6, 47.5, 46.3, 33.1, 25.4, 25.0, 17.8, 17.4, -3.6, -3.9, -6.0, -6.1. **IR (ν_{max}/cm^{-1}):** 2953, 2930, 2857, 1738, 1717, 1682. **HRMS m/z (NSI/ion trap) Calc. for $C_{25}H_{45}O_7Si_2$ (M+H):** 513.2684**; found** 513.2698.

Preparation of dimethyl (2a^1*S,4a*R**,6a*R**)-4*a*-((*tert*-butyldimethylsilyl)oxy)-4-oxo-2*a*^{1},4,4*a*,5,6,6*a*-hexahydrocyclopenta[*cd*]pentalene-1,1(2*H*)-dicarboxylate.**

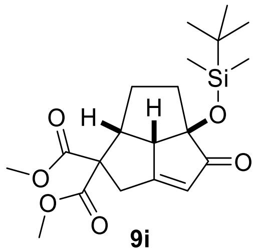

9i

(a) Dimethyl 2-(3-((*tert*-butyldimethylsilyl)oxy)cyclopent-2-en-1-yl)-2-(prop-2-yn-1-yl)malonate dicobalthexacarbonyl complex **8i**, 0.35 g, 0.53 mmol; (b) 5 mL; (c) DodSMe, 0.67 mL, 2.53 mmol; (d) 70 °C; (e) 16 h; (f) dimethyl (2a^1*S**,4a*R**,6a*R**)-4*a*-((*tert*-butyldimethylsilyl)oxy)-4-oxo-2*a*^{1},4,4*a*,5,6,6*a*-hexahydrocyclopenta[*cd*]pentalene-1,1(2*H*)-dicarboxylate, **9i**, 0.18 g, 0.47 mmol, **89%**; and (g) white solid.

^{1}H NMR ($CDCl_3$, 400 MHz): δ_H 6.08 (1H, t, ^4J = 1.9 Hz), 3.81 (3H, s), 3.75 (3H, s), 3.63 (1H, d, ^2J = 18.0 Hz), 3.41 (1H, d, J = 7.3 Hz) 3.32 (1H, dt, J = 11.1 Hz, J = 7.3 Hz), 2.93 (1H, dt, ^2J = 18.0 Hz, ^4J = 1.9 Hz), 2.11 (1H, dd J = 7.4 Hz, J = 6.8 Hz), 1.96 – 1.85 (1H, m), 1.74 (1H, dt, ^2J = 12.9 Hz, J = 7.4 Hz), 0.98 – 0.92 (1H, m), 0.90 (9H, s), 0.18 (3H, s), 0.08 (3H, s). **^{13}C NMR ($CDCl_3$, 101 MHz):** δ_C 208.6, 178.7, 171.6, 169.2, 127.3, 89.4, 63.4, 62.0, 52.7, 52.2, 44.7, 39.9, 34.6, 25.7, 25.3, 17.7, -3.7, -4.0. **IR (ν_{max}/cm^{-1})** 2955, 2936, 2859, 1755, 1730, 1711, 1628. **HRMS m/z (NSI/ion trap) Calc. for $C_{20}H_{31}O_6Si$ (M+H):** 395.1890; **found:** 395.1888. **Melting point**: 79 – 81 °C.

Preparation of dimethyl (2a[1]*S,4a*R**,7a*R**)-4a-((*tert*-butyldimethylsilyl)oxy)-4-oxo-2,2a[1],4,4a,5,6,7,7a-octahydro-1*H*-cyclopenta[*cd*]indene-1,1-dicarboxylate.**

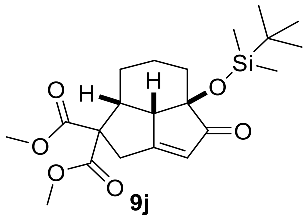

9j

(a) Dimethyl 2-(3-((*tert*-butyldimethylsilyl)oxy)cyclohex-2-en-1-yl)-2-(prop-2-yn-1-yl)malonate dicobalthexacarbonyl complex **8j**, 0.33 g, 0.5 mmol; (b) 5 mL; (c) DodSMe, 0.63 mL, 2.4 mmol; (d) 70 °C; (e) 16 h; (f) dimethyl (2a[1]*S**,4a*R**,7a*R**)-4*a*-((*tert*-butyldimethylsilyl)oxy)-4-oxo-2,2*a*1,4,4*a*,5,6,7,7*a*-octahydro-1*H*-cyclopenta[*cd*]indene-1,1-dicarboxylate, **9j**, 0.07 g, 0.16 mmol, **31%**; and (g) colourless oil.

^{1}H NMR ($CDCl_3$, 400 MHz): δ_H 5.89 – 5.90 (1H, m), 3.79 (3H, s), 3.78 (3H, s), 3.60 (1H, dt, ^{2}J = 20.7 Hz, ^{4}J = 1.9 Hz), 3.38 (1H, d, J = 7.4 Hz), 3.14 (1H, dd, ^{2}J = 20.7 Hz, ^{4}J = 2.2 Hz), 3.10 – 3.01 (1H, m), 1.80 (1H, d, ^{2}J = 14.4 Hz), 1.62 – 1.43 (4H, m), 1.32 (1H, td, ^{2}J = 14.0 Hz, J = 3.4 Hz), 0.93 (9H, s), 0.17 (3H, s), 0.07 (3H, s). **^{13}C NMR ($CDCl_3$, 101 MHz):** δ_C 209.9, 177.7, 171.2, 168.8, 121.6, 82.0, 64.9, 56.6, 52.6, 40.1, 34.0, 25.3, 22.9, 19.8, 17.8, -3.6, -4.0. **IR (ν_{max}/cm^{-1})**: 2951, 2928, 2857, 2012, 1732, 1719, 1632. **HRMS m/z (NSI/ion trap) Calc. for $C_{21}H_{33}O_6Si$ (M+H):** 409.2041; **found:** 409.2047.

Preparation of dimethyl 4-((*tert*-butyldimethylsilyl)oxy)-5-oxo-3,3*a*,4,5-tetrahydropentalene-2,2(1*H*)-dicarboxylate.

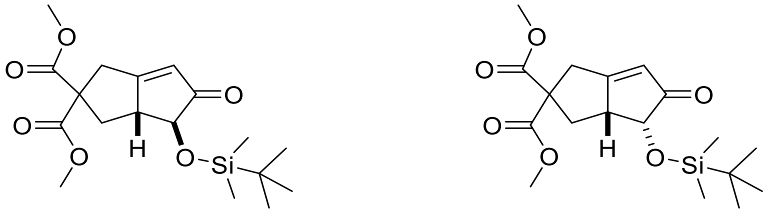

***anti*-11a** ***syn*-11a**

(a) Dimethyl 2-(3-((*tert*-butyldimethylsilyl)oxy)allyl)-2-(prop-2-yn-1-yl)malonate dicobalthexacarbonyl complexes **10a**, 0.27 g, 0.43 mmol, *trans*:*cis,* 71:29; (b) 4.5 mL; (c) DodSMe, 0.54 mL, 2.05 mmol; (d) 70 °C; (e) 16 h; (f) dimethyl 4-((*tert*-butyldimethylsilyl)oxy)-5-oxo-3,3*a*,4,5-tetrahydropentalene-2,2(1*H*)-dicarboxylate, **11a**, 0.14 g, 0.38 mmol, **88%**, *anti*:*syn,* 72:28; and (g) pale yellow oil.

***anti*-11a**

^{1}H NMR ($CDCl_3$, 400 MHz): δ_H 5.95 (1H, dd, ^{4}J = 2.0 Hz, ^{4}J = 1.7 Hz), 4.00 (1H, d, J = 3.5 Hz), 3.81 (3H, s), 3.77 (3H, s), 3.38 – 3.19 (2H, m), 3.07 – 3.00 (1H, m), 2.87 (1H, dd, ^{2}J = 13.2 Hz, J = 8.0 Hz), 1.94 (1H, d, ^{2}J = 13.0 Hz), 0.93 (9H, s), 0.17 (3H, s), 0.14 (3H, s). **^{13}C NMR ($CDCl_3$, 101 MHz)**: δ_C 206.3, 178.0, 171.1, 170.7, 123.0, 81.3, 59.8, 52.8, 52.7, 52.5, 37.4, 35.2, 25.3, 17.9, -5.0, -5.6.

***syn*-11a**

^{1}H NMR ($CDCl_3$, 400 MHz): δ_H 5.89 – 5.87 (1H, m), 4.16 (1H, d, *J* = 5.6 Hz), 3.80 (3H, s), 3.76 (3H, s), 3.31 – 3.30 (2H, m), 3.17 – 3.09 (1H, m), 2.47 (1H, dd, ^2J = 13.2 Hz, *J* = 8.0 Hz), 2.24 (1H, t, ^2J = 12.8 Hz), 0.89 (9H, s), 0.16 (3H, s), 0.13 (6H, s). **^{13}C NMR ($CDCl_3$, 101 MHz)**: δ_C 207.1, 183.4, 171.7, 170.7, 122.3, 72.3, 59.7, 52.7, 52.6, 49.3, 35.3, 31.9, 25.2, 17.9, -5.0, -5.7.

The following data refers to the mixture of isomers.

IR (v_{max}/cm^{-1}): 2953, 1731, 1637, 1438, 1159. **HRMS m/z (NSI) Calc. for $C_{18}H_{29}O_6Si$ (M+H)**: 369.1728; **found** 369.1730.

Preparation of dimethyl 4-((triethylsilyl)oxy)-5-oxo-3,3*a*,4,5-tetrahydropentalene-2,2(1*H*)-dicarboxylate.

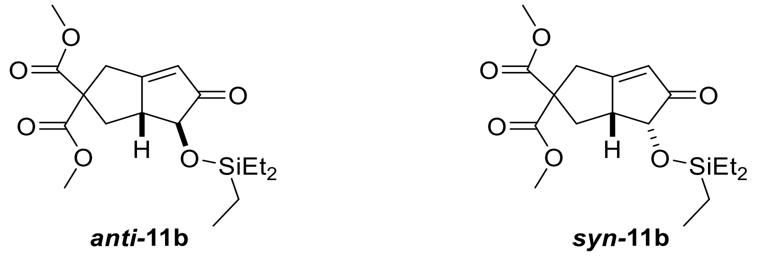

***anti*-11b** ***syn*-11b**

(a) Dimethyl 2-(3-((triethylsilyl)oxy)allyl)-2-(prop-2-yn-1-yl)malonate dicobalthexacarbonyl complexes **10b**, 0.38 g, 0.60 mmol; *trans*:*cis*, 71:29; (b) 6 mL; (c) DodSMe, 0.75 mL, 2.84 mmol; (d) 70 °C; (e) 16 h; (f) dimethyl 4-((triethylsilyl)oxy)-5-oxo-3,3*a*,4,5-tetrahydropentalene-2,2(1*H*)-dicarboxylate, **11b**, 0.19 g, 0.52 mmol, **86%**, *anti*:*syn*, 70:30; and (g) pale yellow oil.

anti-11b

^{1}H NMR ($CDCl_3$, 400 MHz): δ_H 5.95 – 5.93 (1H, m), 3.97 (1H, d, *J* = 3.2 Hz), 3.79 (3H, s), 3.75 (3H, s), 3.37 – 3.32 (1H, m), 3.22 – 3.21 (1H, m), 3.17 – 3.02, (1H, m), 2.89 – 2.84 (1H, m), 1.93 (1H, dd, ^2J = 13.2 Hz, *J* = 11.6 Hz), 0.98 (9H, t, *J* = 8.0 Hz), 0.70 – 0.63 (6H, m). **^{13}C NMR ($CDCl_3$, 101 MHz)**: δ_C 207.1, 183.4, 171.8, 170.7, 122.3, 72.2, 59.7, 52.8, 52.6, 49.4, 35.4, 31.7, 6.2, 4.3.

syn-11b

^{1}H NMR ($CDCl_3$, 400 MHz): δ_H 5.88 – 5.87 (1H, m) 4.14 (1H, d, *J* = 5.6 Hz), 3.79 (3H, s), 3.75 (3H, s), 3.31 – 3.29 (2H, m), 3.15 – 3.08 (1H, m), 2.45 (1H, dd, ^2J = 13.2 Hz, *J* = 8.0 Hz), 2.23 (1H, dd, ^2J = 12.9 Hz, *J* = 12.4 Hz), 0.96 (9H, t, *J* = 8.0 Hz), 0.69 – 0.62 (6H, m). **^{13}C NMR ($CDCl_3$, 101 MHz)**: δ_C 206.2, 178.0, 171.0, 170.7, 123.1, 80.9, 59.8, 52.8, 52.7, 52.6, 37.3, 35.2, 6.2, 4.2.

The following data refers to the mixture of isomers.

IR (v_{max}/cm^{-1}): 2931, 2856, 1733, 1637, 1438, 1251. **HRMS m/z (NSI) Calc. for $C_{18}H_{29}O_6Si$ (M+H)**: 369.1728; **found**: 369.1730.

Preparation of 2-tosyl-4-((triethylsilyl)oxy)-2,3,3*a*,4-tetrahydrocyclopenta[c]pyrrol-5(1*H*)-one.

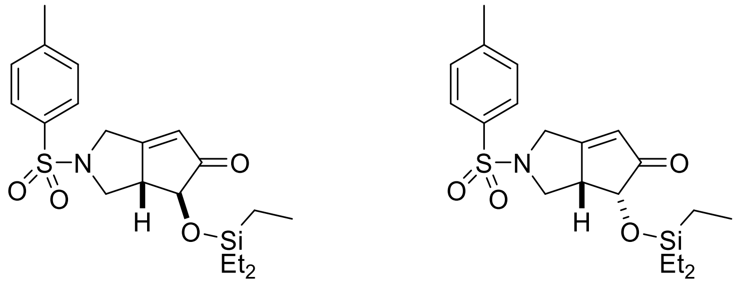

***anti*-11c** ***syn*-11c**

(a) *N*-(Prop-2-yn-1-yl)-*N*-(3-((triethylsilyl)oxy)allyl)toluenesulfonamide dicobalthexacarbonyl complexes **10c**, 0.41 g, 0.62 mmol, *trans*:*cis*, 70:30; (b) 6 mL; (c) DodSMe, 0.79 mL, 2.95 mmol; (d) 70 °C; (e) 3 h; (f) 2-tosyl-4-((triethylsilyl)oxy)-2,3,3*a*,4-tetrahydrocyclopenta[c]pyrrol-5(1*H*)-one, **11c**, 0.17 g, 0.41 mmol, **66%**, *anti*:*syn*, 50:50; and (g) white solid.

anti-11c

^{1}H NMR ($CDCl_3$, 400 MHz): δ_H 7.72 – 7.68 (2H, m), 7.34 – 7.30 (2H, m), 5.97 (1H, dd, ^{4}J = 3.7 Hz, ^{4}J = 1.8 Hz), 4.29 (1H, dt, ^{2}J = 16.8 Hz, ^{4}J = 1.6 Hz), 4.06 (1H, dd, ^{2}J = 9.5 Hz, *J* = 8.5 Hz), 4.00 (1H, dt, ^{2}J = 17.0 Hz, ^{4}J = 1.4 Hz), 3.88 (1H, d, *J* = 3.6 Hz), 3.05 – 2.95 (1H, m), 2.78 (1H, dd, *J* = 10.6 Hz, ^{2}J = 9.5 Hz), 2.40 (3H, s), 0.94 (9H, t, *J* = 7.9 Hz), 0.65 – 0.54 (6H, m). **^{13}C NMR ($CDCl_3$, 101 MHz)**: δ_C 205.6, 172.3, 143.7, 132.9, 129.6, 126.9, 123.1, 78.7, 51.9, 51.4, 47.9, 21.0, 6.1, 4.2.

syn-11c

^{1}H NMR ($CDCl_3$, 400 MHz): δ_H 7.72 – 7.68 (2H, m), 7.34 – 7.30 (2H, m), 5.88 – 5.86 (1H, m), 4.26 (1H, dt, ^{2}J = 17.1 Hz, ^{4}J = 2.0 Hz), 4.12 (1H, d, *J* = 5.4 Hz), 4.04 (1H, d, ^{2}J = 17.0 Hz) 3.70 (1H, apparent t, ^{2}J = 8.0 Hz, *J* = 8.0 Hz) 3.20 – 3.11 (1H, m), 3.08 (1H, dd, *J* = 10.6 Hz, ^{2}J = 8.3 Hz), 2.46 (3H, s), 0.88 (9H, t, *J* = 7.9 Hz), 0.65 – 0.54 (6H, m). **^{13}C NMR ($CDCl_3$, 101 MHz)**: δ_C 204.6, 177.1, 143.6, 132.7, 129.4, 122.6, 71.3, 50.9, 48.1, 47.4, 46.0, 21.0, 6.1, 4.2.

The following data refers to the mixture of isomers.

IR (ν_{max}/cm^{-1}): 2953, 2937, 2911, 2877, 1715, 1645. **HRMS M/Z (NSI) Calc. for $C_{20}H_{30}NO_4SSi$ (M+H)**: 408.1659; **found**: 408.1659. **Melting point**: Decomposes over 60 – 68 °C.

Preparation of dimethyl 1-((*tert*-butyldimethylsilyl)oxy)-2-oxo-1,2,4,6,7,7a-hexahydro-5*H*-indene-5,5-dicarboxylate.

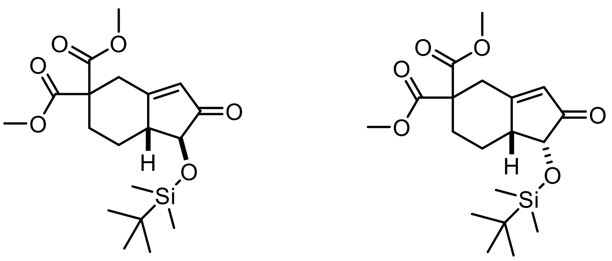

***anti*-11d** ***syn*-11d**

(a) Dimethyl 2-(4-((*tert*-butyldimethylsilyl)oxy)but-3-en-1-yl)-2-(prop-2-yn-1-yl)malonate dicobalthexacarbonyl complexes **10d**, 0.94 g, 1.47 mmol, *cis*:*trans*, 92:8; (b) 15 mL; (c) DodSMe, 1.85 mL, 6.97 mmol; (d) 70 °C; (e) 24 h; (f) dimethyl 1-((*tert*-butyldimethylsilyl)oxy)-2-oxo-1,2,4,6,7,7*a*-hexahydro-5*H*-indene-5,5-dicarboxylate, **11d**, 0.31 g, 0.81 mmol, **55%**, *anti*:*syn*, 73:27; and (g) white solid.

***anti*-11d**

^{1}H NMR (CDCl$_3$, 400 MHz): δ_H 5.97 (1H, t, ^4J = 2.0 Hz), 3.81 (1H, d, J = 2.8 Hz), 3.79 (3H, s), 3.75 (3H, s), 3.47 (1H, dd, ^2J = 14.4 Hz, ^4J = 2.0 Hz), 2.67-2.62 (1H, m), 2.58 – 2.52 (2H, m), 2.34 – 2.25 (1H, m), 1.99 (1H, td, ^2J = 13.7 Hz, J = 3.4 Hz), 1.39 – 1.28 (1H, m), 0.93 (9H, s), 0.18 (3H, s). 0.14 (3H, s). **^{13}C NMR (CDCl$_3$, 101 MHz)**: δ_C 205.0, 172.5, 170.7, 169.7, 126.8, 79.2, 55.2, 52.6, 52.4, 48.2, 34.9, 29.8, 27.2, 25.3, 17.8, -4.8, -5.6. **IR (ν_{max}/cm^{-1})**: 2928, 2855, 1722, 1705, 1618. **HRMS m/z (NSI) Calc. for $C_{19}H_{31}O_6Si$ (M+H)**: 383.1884; **found** 383.1885. **Melting point**: 122 – 124 °C.

^{1}H and ^{13}C NMR signals of relating to the *syn*-isomer could not be fully assigned.

One Pot Complexation/Pauson-Khand Reaction

Preparation of dimethyl 3*a*-((*tert*-butyldimethylsilyl)oxy)-5-oxo-3,3*a*,4,5-tetrahydropentalene-2,2(1*H*)-dicarboxylate.

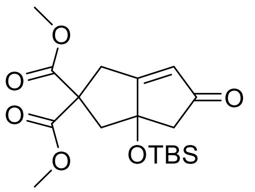

4

Dimethyl 2-(2-((*tert*-butyldimethylsilyl)oxy)allyl)-2-(prop-2-yn-1-yl)malonate **2** (0.058 g, 0.170 mmol) and distilled DCE (1.7 mL) were added to a flame-dried, round-bottom flask equipped with a stirrer bar. $Co_2(CO)_8$ (0.059 g, 0.172 mmol) was added and the solution was stirred for 1 h at room temperature. After this time, DodSMe (0.21 ml, 0.81 mmol) was added and the mixture was heated to the 70 °C for 16 h. At this point, the reaction mixture was filtered through celite and the solvent was removed *in vacuo* to provide the crude material. The crude material was purified by flash column chromatography (pet. ether:Et_2O, 90:10) and concentrated *in vacuo* to provide dimethyl 3-((*tert*-butyldimethylsilyl)oxy)-5-oxo-3,3,4,5-tetrahydropentalene-2,2(1*H*)-dicarboxylate **4** as a white solid (0.058 g, 0.157 mmol, **93%**).

Data for this compound can be found on page S39.

IV Deprotection of silyl-protected cyclopentenones

Typical Procedure D for the deprotection of silyl-protected cyclopentenones

Preparation of dimethyl 3*a*-hydroxy-5-oxo-3,3*a*,4,5-tetrahydropentalene-2,2(1*H*)-dicarboxylate.

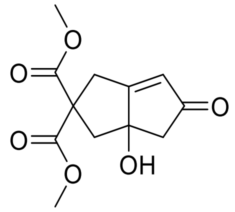

12a

Dimethyl 5-oxo-3a-((triethylsilyl)oxy)-3,3a,4,5-tetrahydropentalene-2,2(1*H*)-dicarboxylate **7a** (0.15 g, 0.41 mmol) and THF:H_2O (10:1, 2.42 mL) were added to a flame-dried, round-bottom flask under an argon atmosphere. 3 M aqueous HCl (0.04 mL) was added and the resulting solution was stirred for 16 h at ambient temperature before being quenched by the addition of a saturated aqueous solution of $NaHCO_3$ (10 mL). Following this, Et_2O (10 mL) was added to create a biphasic mixture, which was separated, and the aqueous layer was washed with Et_2O (3 × 10 mL). The combined organic extracts were washed with brine (5 mL), dried over Na_2SO_4, filtered, and concentrated *in vacuo* to provide the crude product as a yellow oil. The crude material was purified by flash column chromatography (pet. ether 40-60:Et_2O, 100:0 – 0:100) and concentrated *in vacuo* to give dimethyl 3a-hydroxy-5-oxo-3,3a,4,5-tetrahydropentalene-2,2(1*H*)-dicarboxylate **12a** (0.93 g, **88**%) as a white gum.

^{1}H NMR ($CDCl_3$, 400 MHz): δ_H 5.91 – 5.80 (1H, m), 3.82 (3H, s), 3.76 (3H, s), 3.62 (1H, dd, ^2J = 18.4 Hz, ^4J = 2.0 Hz), 3.28 (1H, d, ^2J = 18.4 Hz), 3.06 (1H, s), 2.97 (1H, d, ^2J = 14.3 Hz), 2.59 (1H, d, ^2J = 18.0 Hz), 2.53 (1H, d, ^2J = 18.4 Hz), 2.23 (1H, d, ^2J = 14.4 Hz). **^{13}C NMR ($CDCl_3$, 101 MHz)**: δ_C 206.9, 181.0, 172.3, 170.8, 125.5, 83.4, 60.0, 53.1, 52.9, 47.7, 44.2, 33.3. **IR (v_{max}/cm^{-1})**: 3472, 2955, 1710, 1641, 1249, 1058. **HRMS m/z (NSI) Calc. for $C_{12}H_{18}NO_6$ (M+NH_4)**: 272.1129; **found** 272.1130.

The following experiments were carried out according to ***Typical Procedure D.***

Data are reported as: (a) amount of cyclopentenone substrate; (b) volume of 3 M aqueous HCl solution; (c) reaction temperature; (d) reaction time; (e) isolated yield; and (f) product appearance. Individual analysis for each product is provided.

Preparation of dimethyl 3*a*-hydroxy-6-methyl-5-oxo-3,3*a*,4,5-tetrahydropentalene-2,2(1*H*)-dicarboxylate.

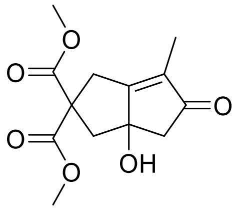

12b

(a) Dimethyl 6-methyl-5-oxo-3*a*-((triethylsilyl)oxy)-3,3*a*,4,5-tetrahydropentalene-2,2(1*H*)-dicarboxylate **9d**, 0.4 g, 1.09 mmol; (b) 0.12 mL; (c) rt; (d) 16 h; (e) dimethyl 3*a*-hydroxy-6-methyl-5-oxo-3,3*a*,4,5-tetrahydropentalene-2,2(1*H*)-dicarboxylate, **12b**, 0.26 g, 0.97 mmol, **94%**; and (f) colourless oil.

^{1}H NMR ($CDCl_3$, 400 MHz): δ_H 3.82 (3H, s), 3.76 (3H, s), 3.53 – 3.48 (1H, m), 3.19 (1H, d, ^2J = 18.4 Hz), 2.97 (1H, d, ^2J = 14.0 Hz), 2.85 (1H, s), 2.60 (1H, d, ^2J = 18.0 Hz), 2.51 (1H, d, ^2J = 18.0 Hz), 2.17 (1H, d, ^2J = 14.4 Hz), 1.72 (3H, d, ^5J = 0.8 Hz). **^{13}C NMR ($CDCl_3$, 101 MHz)**: δ_C 206.8, 173.5, 172.5, 171.0, 133.6, 81.6, 60.1, 53.0, 52.8, 47.3, 44.6, 32.3, 8.0. **IR (ν_{max}/cm^{-1})**: 3453, 2955, 1731, 1714, 1673, 1253, 1069. **HRMS m/z (NSI) Calc. for $C_{13}H_{16}O_6Na$ (M+Na)**: 291.0839; **found** 291.0837.

Preparation of 3a-hydroxy-2-tosyl-2,3,3a,4-tetrahydrocyclopenta[c]pyrrol-5(1*H*)-one.

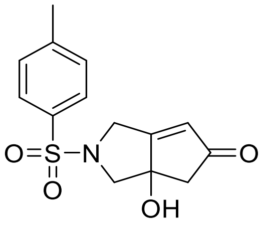

12c

(a) 2-Tosyl-3*a*-((triethylsilyl)oxy)-2,3,3*a*,4-tetrahydrocyclopenta[c]pyrrol-5(1*H*)-one **9b**, 0.15 g, 0.37 mmol; (b) 0.04 mL; (c) rt; (d) 5 h; (e) 3*a*-hydroxy-6-methyl-2-tosyl-2,3,3*a*,4-tetrahydrocyclopenta[c]pyrrol-5(1*H*)-one, **12c**, 0.089 g, 0.3 mmol, **82%**; and (f) white solid.

^{1}H NMR ($CDCl_3$, 400 MHz): δ_H = 7.77 (2H, d, *J* = 8.4 Hz), 7.37 (2H, d, *J* = 8.0 Hz), 5.99 – 5.98 (1H, m), 4.45 (1H, dd, ^2J = 16.4 Hz, ^4J = 2.0 Hz), 4.13 (1H, d, ^2J = 16.6 Hz), 3.98 (1H, d, ^2J = 10.8 Hz), 3.07 (1H, d, ^2J = 11.2 Hz), 2.60 (1H, d, ^2J = 17.8 Hz), 2.47 (1H, d, ^2J = 18.0 Hz), 2.46 (3H, s), 2.27 (1H, s). **^{13}C NMR ($CDCl_3$, 101 MHz)**: δ_C 204.9, 174.5, 143.9, 132.9, 129.5, 127.1, 126.1, 81.1, 57.5, 45.6, 45.3, 21.1. **IR (ν_{max}/cm^{-1})**: 3380 (br), 3258, 1731, 1701, 1647, 1158. **HRMS m/z (ESI) Calc. for $C_{14}H_{16}NO_4S$ (M+H)**: 294.0795; **found** 294.0797. **Melting Point**: 108 – 109 °C.

Preparation of 3*a*-hydroxy-6-methyl-2-tosyl-2,3,3*a*,4-tetrahydrocyclopenta[c]pyrrol-5(1*H*)-one.

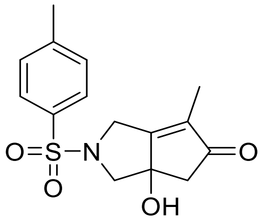

12d

(a) 6-Methyl-2-tosyl-3*a*-((triethylsilyl)oxy)-2,3,3*a*,4-tetrahydrocyclopenta[c]pyrrol-5(1*H*)-one **9e**, 0.2 g, 0.47 mmol; (b) 0.06 mL; (c) rt; (d) 16 h; (e) 3*a*-hydroxy-6-methyl-2-tosyl-2,3,3*a*,4-tetrahydrocyclopenta[c]pyrrol-5(1*H*)-one, **12d**, 0.13 g, 0.42 mmol, **90%**; and (f) white solid.

^{1}H NMR ($CDCl_3$, 400 MHz): δ_H 7.76 – 7.74 (2H, m), 7.36 – 7.34 (2H, m), 4.35 (1H, dd, ^2J = 16.0 Hz, ^5J = 1.6 Hz), 4.05 (1H, d, ^2J = 16.0 Hz), 3.95 (1H, d, ^2J = 10.8 Hz), 3.02 (1H, d, ^2J = 10.8 Hz), 2.93 (1H, s), 2.61 (1H, d, ^2J = 18.0 Hz), 2.44 (1H, d, ^2J = 18.0 Hz), 2.42 (3H, s), 1.70 (3H, s). **^{13}C NMR ($CDCl_3$, 101 MHz)**: δ_C 205.7, 167.4, 143.7, 134.6, 133.1, 129.5, 127.0, 79.2, 57.9, 45.3, 45.0, 21.1, 8.3. **IR (v_{max}/cm^{-1})**: 3440, 2922, 1721, 1687, 1600, 1158. **HRMS (NSI) Calc. for $C_{15}H_{18}NO_4S$ (M+H)**: 308.0951; **found** 308.0952. **Melting Point**: 143 – 145 °C.

Preparation of 3*a*-hydroxy-6-methyl-3*a*,4-dihydro-1*H*-cyclopenta[c]furan-5(3*H*)-one.

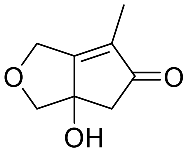

12e

(a) 3*a*-((*Tert*-butyldimethylsilyl)oxy)-6-methyl-3*a*,4-dihydro-1*H*-cyclopenta[c]furan-5(3*H*)-one **9f**, 0.21 g, 0.79 mmol; (b) 0.043 mL; (c) 40 °C; (d) 100 h; (e) 3*a*-hydroxy-6-methyl-3*a*,4-dihydro-1*H*-cyclopenta[c]furan-5(3*H*)-one, **12e**, 0.075g, 0.49 mmol, **62%**; and (f) pale yellow oil.

^{1}H NMR ($CDCl_3$, 400 MHz): δ_H 4.74 (1H, d, ^2J = 15.6 Hz), 4.54 (1H, d, ^2J = 16.0 Hz), 4.20 (1H, d, ^2J = 9.6 Hz), 3.55 (1H, d, ^2J = 9.6 Hz), 3.22 (1H, s), 2.65 (1H, d, ^2J = 18.0 Hz), 2.51 (1H, d, ^2J = 18.0 Hz), 1.76 (3H, s). **^{13}C NMR ($CDCl_3$, 101 MHz)**: δ_C 207.1, 171.9, 133.2, 80.4, 75.8, 63.2, 44.6, 8.5. **IR (v_{max}/cm^{-1})**: 3387, 1707, 1686, 1030, 995. **HRMS m/z (NSI) Calc. for $C_8H_{11}O_3$ (M+H)**: 155.0708; **found** 155.0707.

Preparation of dimethyl (3*aR,4*S**)-4-hydroxy-5-oxo-3,3*a*,4,5-tetrahydropentalene-2,2(1*H*)-dicarboxylate.**

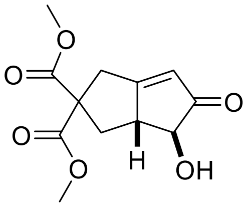

13a

(a) Dimethyl 4-((*tert*-butyldimethylsilyl)oxy)-5-oxo-3,3*a*,4,5-tetrahydropentalene-2,2(1*H*)-dicarboxylate **11a**, 0.24 g, 0.64 mmol, *anti*:*syn,* 72:28; (b) 0.07 mL; (c) rt; (d) 18 h; (e) dimethyl (3*aR**,4*S**)-4-hydroxy-5-oxo-3,3*a*,4,5-tetrahydropentalene-2,2(1*H*)-dicarboxylate, **13a**, 0.06g, 0.22 mmol, **34%**; and (f) white solid.

^{1}H NMR ($CDCl_3$, 400 MHz): δ_H 6.00 (1H, dd, ^4J = 3.6 Hz, ^4J = 2.0 Hz), 4.00 (1H, d, *J* = 3.2 Hz), 3.79 (3H, s), 3.76 (3H, s), 3.41 (1H, d, ^2J = 19.2 Hz), 3.21 (1H, d, ^2J = 19.2 Hz), 3.09– 3.03 (1H, m), 2.96 (1H, s), 2.94 (1H, dd, ^2J = 12.8 Hz, *J* = 7.6 Hz), 1.98 (1H, dd, ^2J = 13.2 Hz, *J* = 12.0 Hz). **^{13}C NMR ($CDCl_3$, 101 MHz)**: δ_C 207.8, 180.6, 171.0, 170.6, 122.0, 80.4, 60.0, 52.9, 52.7, 52.0, 37.0, 35.2. **IR (v_{max}/cm^{-1})**: 3516, 2957, 1710, 1635, 1252. **HRMS m/z (NSI) Calc. for $C_{12}H_{15}O_6$ (M+H)**: 255.0869; **found** 255.0863. **Melting point**: 108 – 110 °C. Also see the X-ray crystallography data in Section V.

Preparation of (3*aS,4*S**)-4-hydroxy-2-tosyl-2,3,3*a*,4-tetrahydrocyclopenta[c]pyrrol-5(1*H*)-one.**

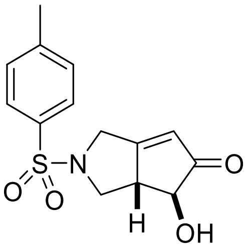

13b

(a) 2-Tosyl-4-((triethylsilyl)oxy)-2,3,3*a*,4-tetrahydrocyclopenta[c]pyrrol-5(1*H*)-one **11c**, 0.54 g, 0.82 mmol, *anti*:*syn*, 50:50; (b) 0.81 mL; (c) rt; (d) 5 h; (e) (3*aS**,4*S**)-4-hydroxy-2-tosyl-2,3,3*a*,4-tetrahydrocyclopenta[c]pyrrol-5(1*H*)-one, **13b**, 0.05g, 0.17 mmol, **20%**; and (f) white solid.

^{1}H NMR ($CDCl_3$, 400 MHz): δ_H 7.78 – 7.75 (2H, m), 7.39 – 7.37 (2H, m), 6.09 (1H, dd, ^4J = 4.0 Hz, ^4J = 2.4 Hz), 4.35 (1H, dt, ^2J = 17.6 Hz, ^4J = 1.6 Hz), 4.18 (1H, dd, ^2J = 9.5 Hz, *J* = 8.6 Hz), 4.09 (1H, d, ^2J = 17.2 Hz), 3.95 (1H, d, *J* = 3.6 Hz), 3.14 – 3.07 (1H, m), 2.92 (1H, s), 2.86 (1H, dd, *J* = 10.6 Hz, ^2J = 9.5 Hz), 2.47 (3H, s). **^{13}C NMR ($CDCl_3$, 101 MHz)**: δ_C 206.5, 174.8, 144.3, 133.3, 130.0, 127.4, 122.6, 78.9, 51.13, 51.09, 47.7, 21.5. **IR (v_{max}/cm^{-1})**: 3445, 3063, 2916, 1711, 1659, 1344, 1159. **HRMS m/z (NSI) Calc. for $C_{14}H_{16}NO_4S$ (M+H)**: 294.0795; **found** 294.0795. **Melting Point**: Decomposes over 153 – 158 °C. Also see the X-ray crystallography data in Section V.

V X-Ray crystallography data for 13a and 13b.

Experimental Technique, Single Crystal Diffraction

Crystal growth was achieved using a slow evaporation procedure between acetone and hexane. More specifically, a nearly saturated solution of the compound in acetone was prepared in a small vial, which was then placed inside a larger vial, which was half filled with hexane. The larger vial was closed tightly with a screw top lid and, over time, the slow evaporation process facilitated crystal growth.

Single crystal x-ray diffraction data for **13a** were measured with an Oxford Diffraction Gemini S instrument while data for **13b** were measured by the National Crystallography Service, University of Southampton, with a Rigaku Saturn724 rotating anode instrument.[15] Data collection and processing used CrysalisPro software.[16] The structures were refined to convergence on F^2 using all independent reflections and the program SHELXL-2018 as implemented within WinGX.[17,18] The structure of **13b** was modelled as disordered such that both mirror image forms sit on the same crystallographic site. Restraints were placed on the disordered heterocyclic group to ensure displacement ellipsoids approximated to normal behaviour. Selected crystallographic data and refinement parameters for **13a** and **13b** are presented in **Table S1**. Views of the molecular structures are given in **Figures S1** and **S2**. CCDC deposition numbers CCDC 2105307 and 2105308 contain the full supplementary crystallographic data for this paper in cif format. These data are provided free of charge by the joint Cambridge Crystallographic Data Centre and Fachinformationszentrum Karlsruhe Access Structures service www.ccdc.cam.ac.uk/structures.

Table S1. Selected crystallographic data and refinement parameters.

**Compound**	**13a**	**13b**
**CCDC**	2105307	2105308
**Formula**	$C_{12}H_{14}O_6$	$C_{14}H_{15}NO_4S$
**Form. Wt.**	254.23	293.33
**Space Group**	$P2_1/n$	$P2_1/c$
**Crystal system**	Monoclinic	Monoclinic
**Temp. (K)**	123(2)	100(2)
**a (Å)**	11.5838(4)	19.602(2)
**b (Å)**	7.3459(3)	5.5072(6)
**c (Å)**	13.1599(4)	12.139(2)
**β (°)**	92.831(3)	97.055(13)
**Volume (Å^3)**	1118.45(7)	1300.5(3)
**Z**	4	4
**λ (Å)**	1.54184	0.71075
**Measured Reflections**	3964	13607
**Unique Reflections**	2177	2300
**2θmax (°)**	146.03	50.06
**R_{int}**	0.0204	0.1199
**Observed Reflections [I>2σI]**	2047	1481
**No. Parameters**	169	186
**S**	1.054	1.006
**R [on *F*, obs refls only]**	0.0399	0.0690
**ωR [on F^2, all data]**	0.1100	0.1899
**Largest diff. peak /hole (eÅ^{-3})**	0.386/-0.205	0.379/-0.486

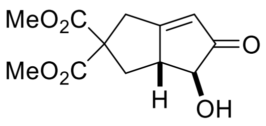

13a

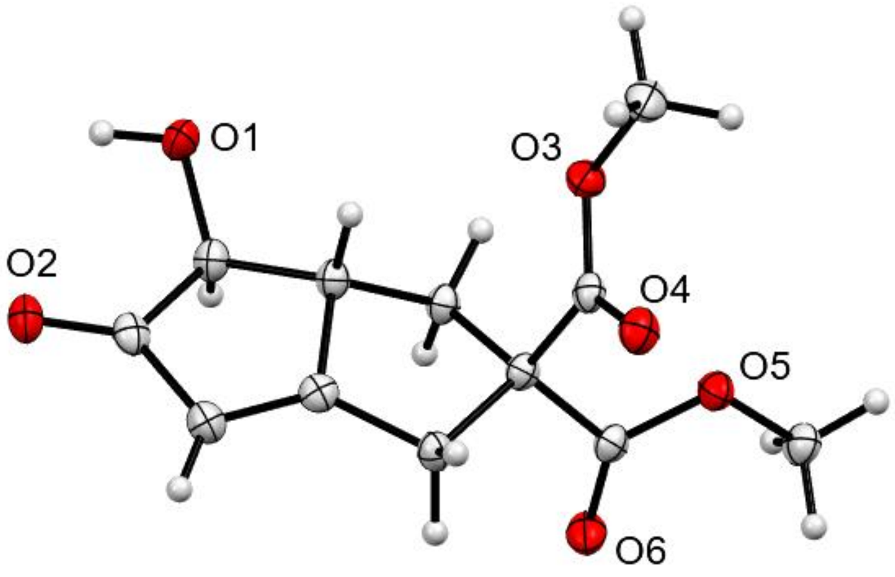

Figure **S1**. ORTEP view of the molecular structure of **13a**. Non-H atoms are drawn as 50% probability ellipsoids and H atoms are drawn as spheres of arbitrary size.

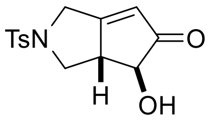

13b

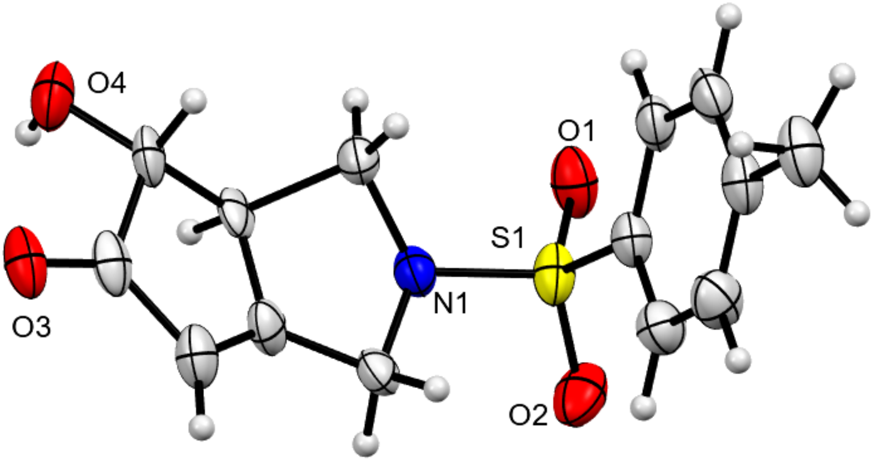

Figure **S2**. ORTEP view of the molecular structure of **13b**. Only one of the two disordered conformations is shown. Non-H atoms are drawn as 50% probability ellipsoids and H atoms are drawn as spheres of arbitrary size.

VI ^{1}H and ^{13}C NMR spectra for all novel compounds

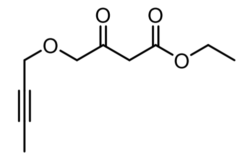

S8

Compound **S8**.
400.13 MHz ^{1}H NMR spectrum
$CDCl_3$

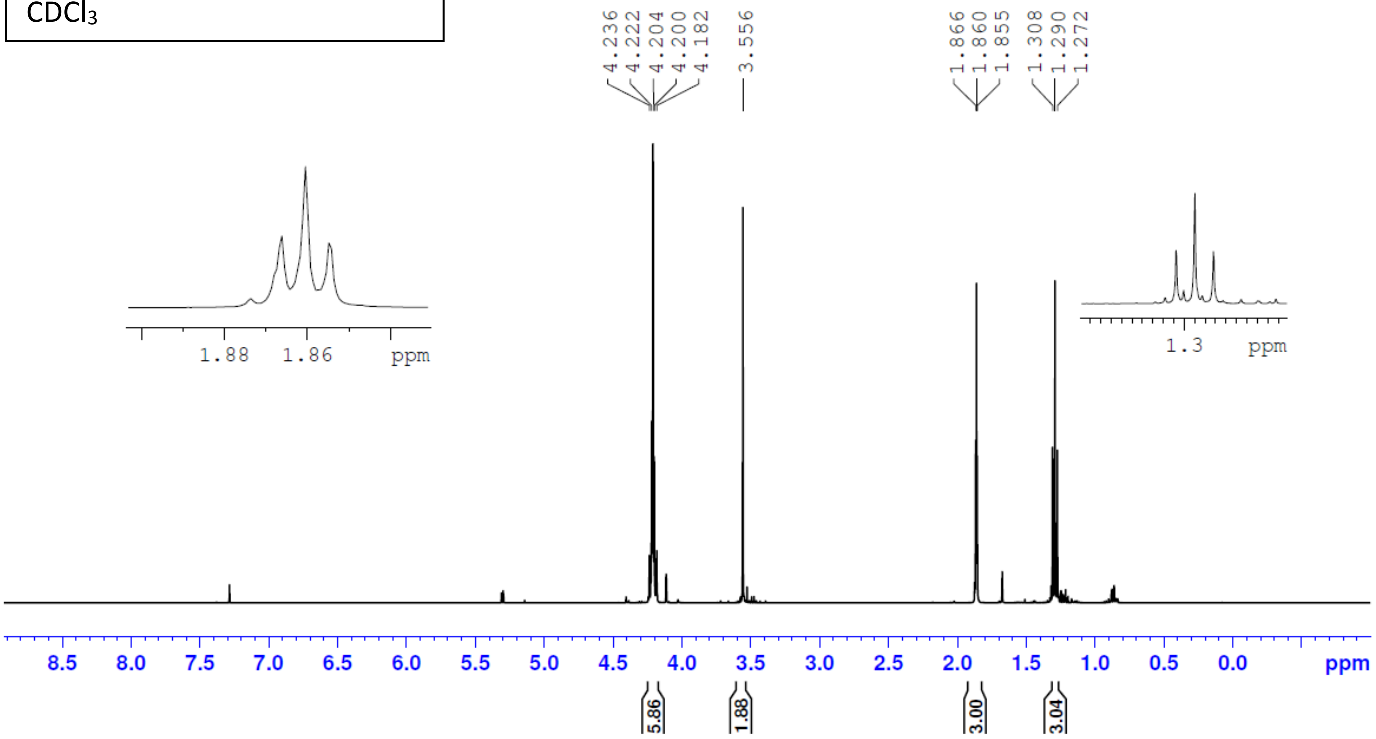

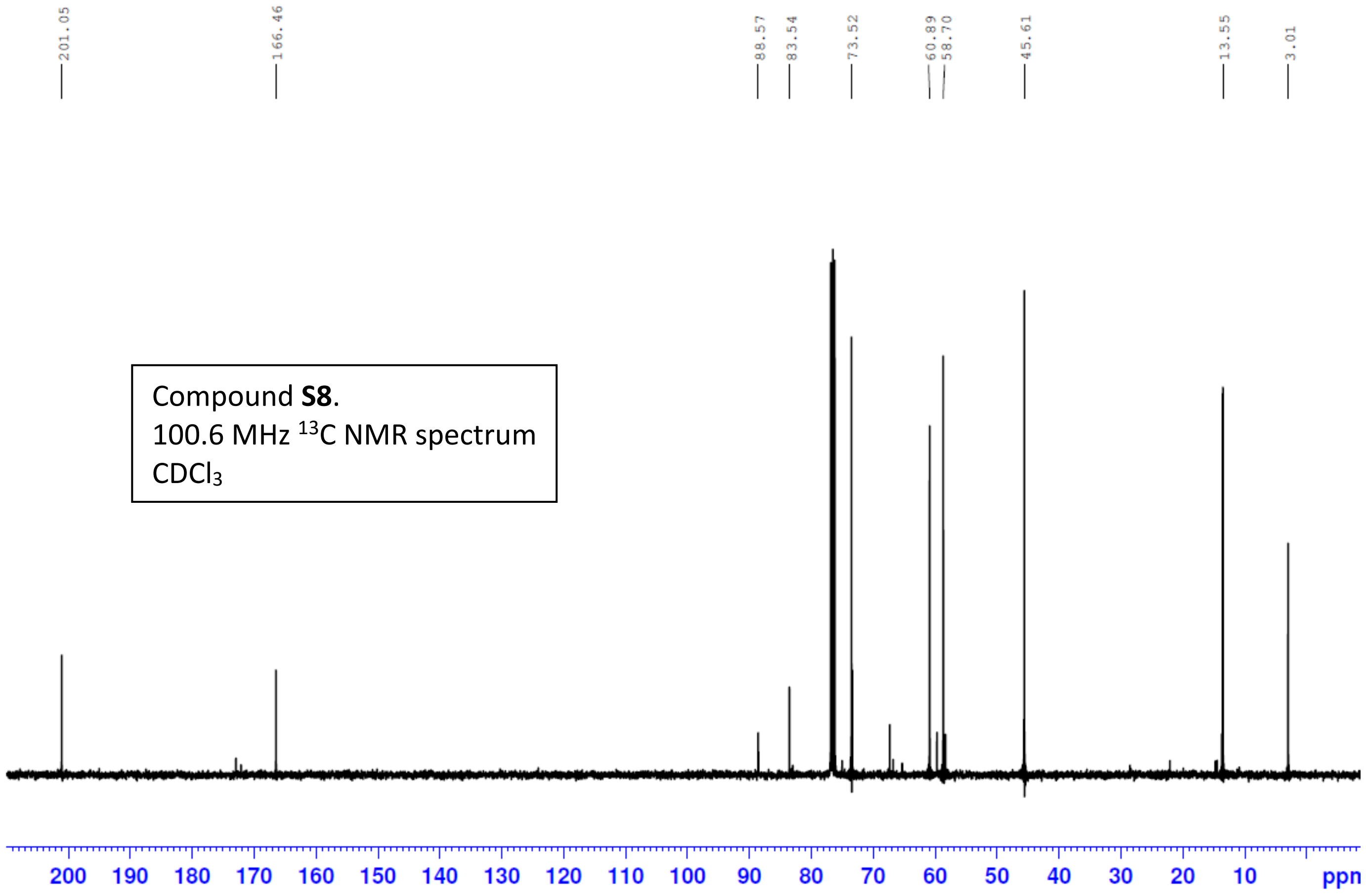

Compound **S8**.
100.6 MHz ^{13}C NMR spectrum
$CDCl_3$

Compound **S9**.
400.13 MHz ^{1}H NMR spectrum
$CDCl_3$

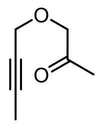

S9

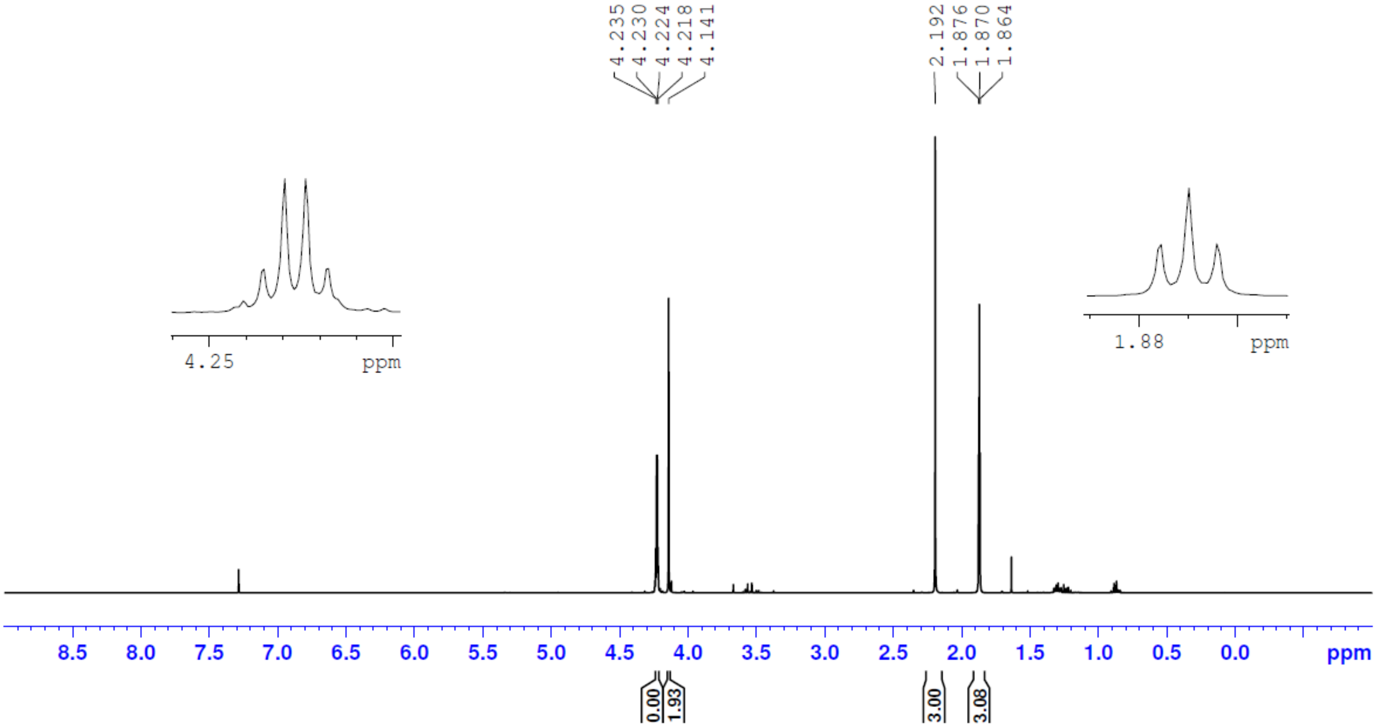

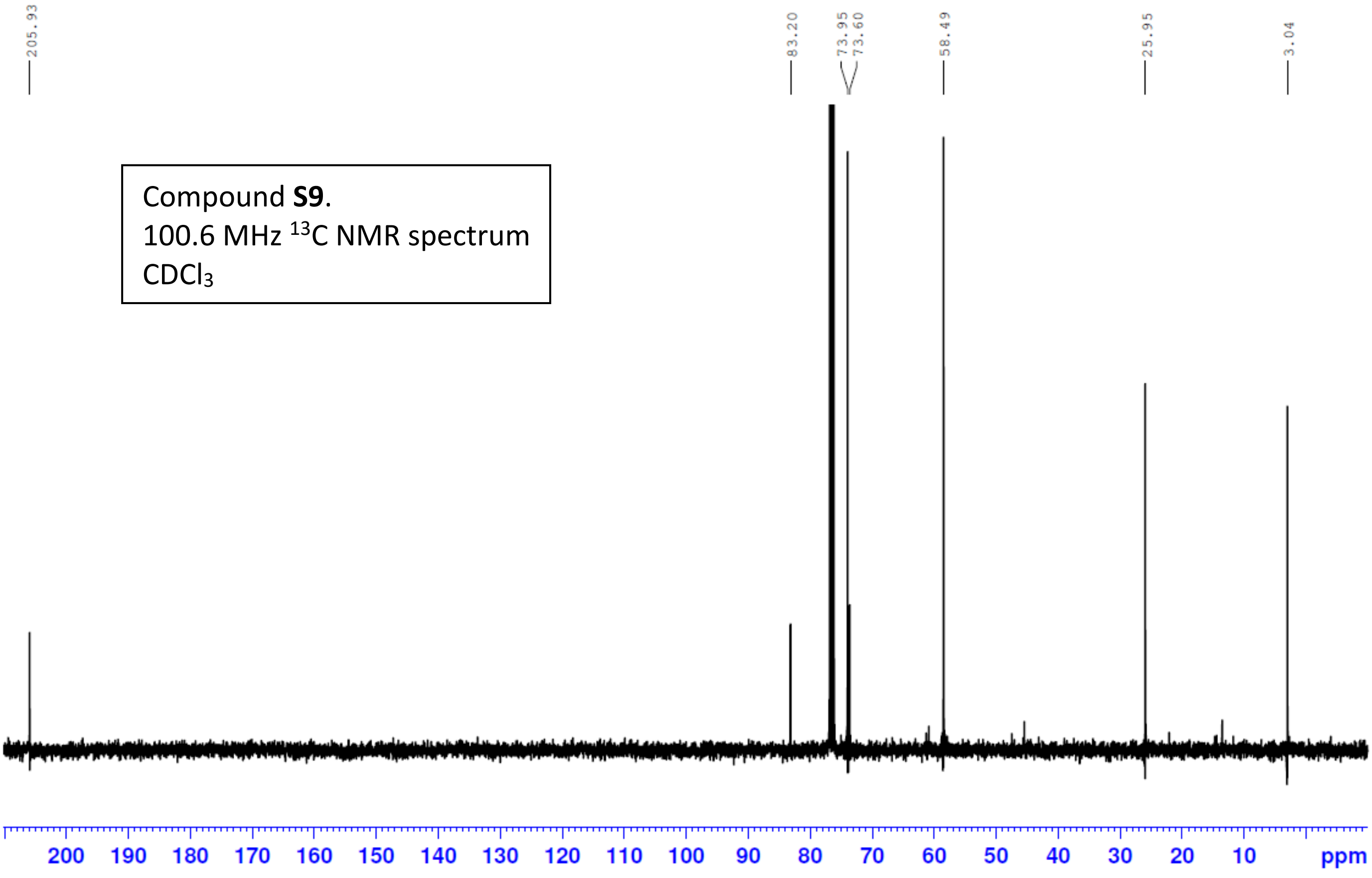

Compound **S9**.
100.6 MHz ^{13}C NMR spectrum
$CDCl_3$

Compound **S10**.
400.13 MHz ^{1}H NMR spectrum
$CDCl_3$

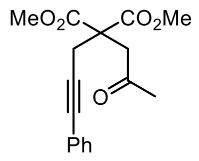

S10

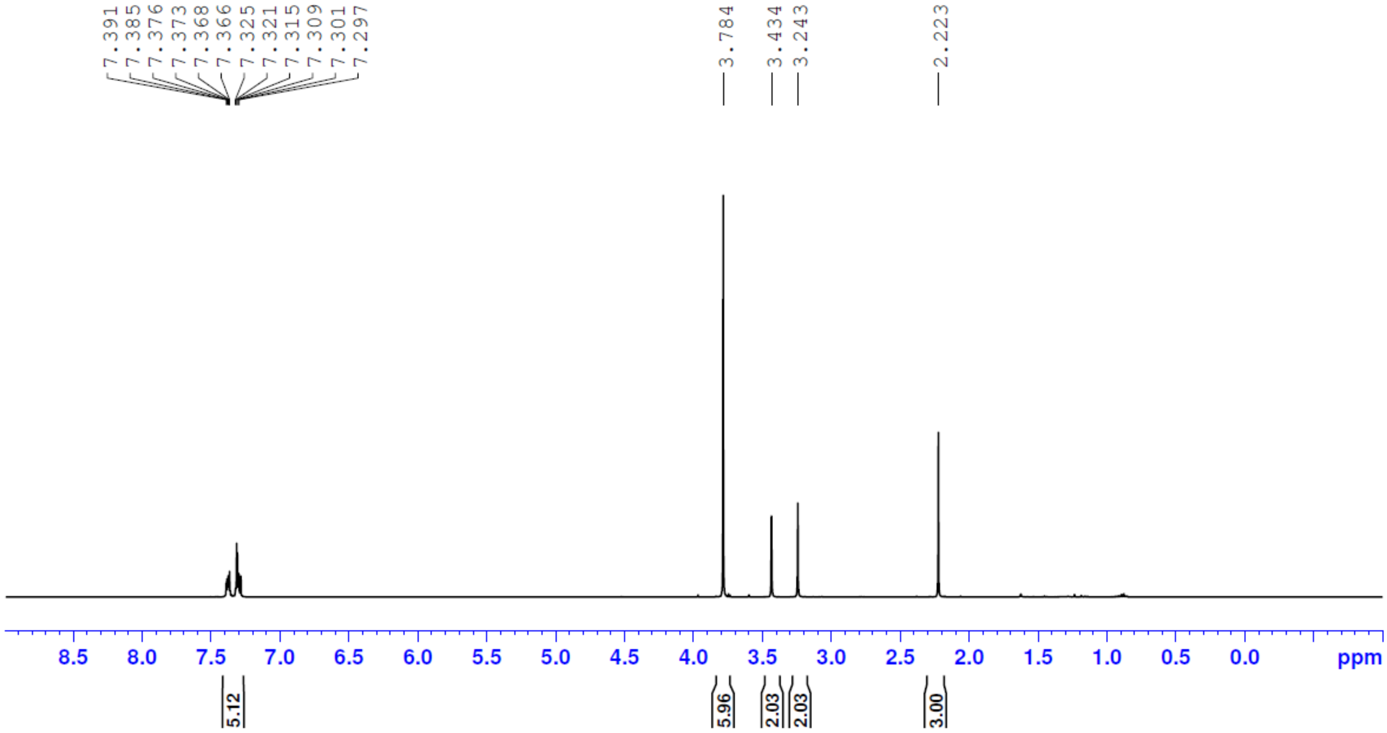

Compound **S10**.
100.6 MHz ^{13}C NMR spectrum
$CDCl_3$

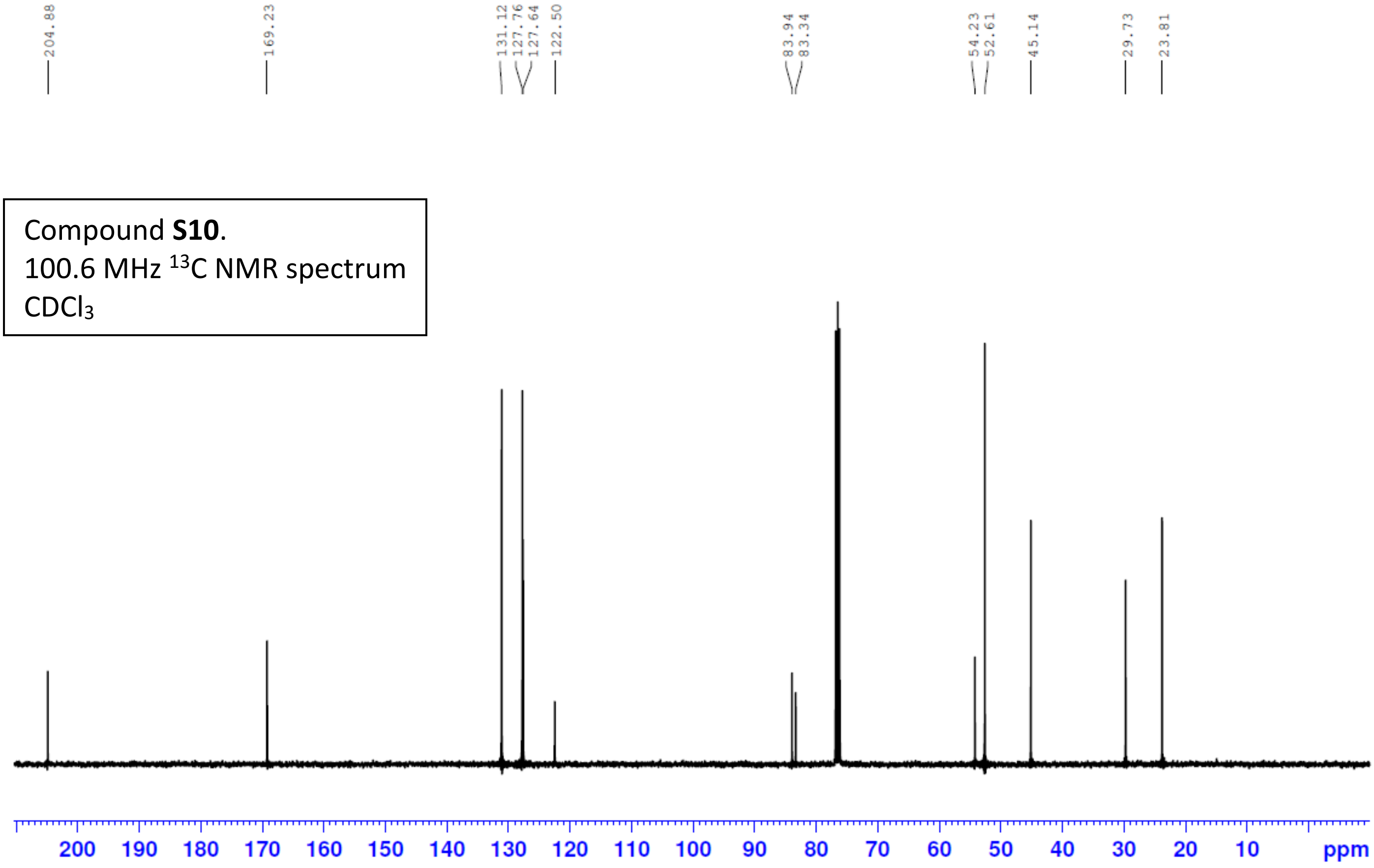

Compound **S11**.
400.13 MHz ^{1}H NMR spectrum
$CDCl_3$

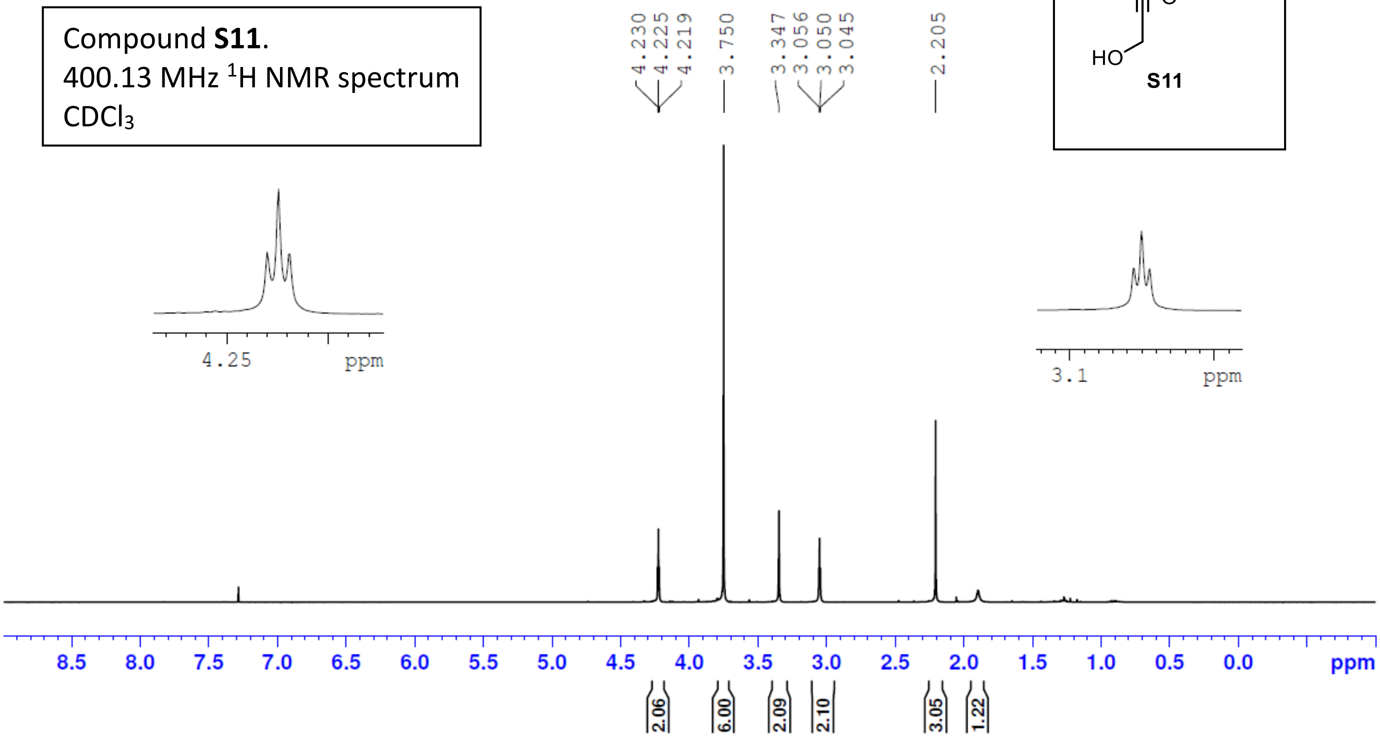

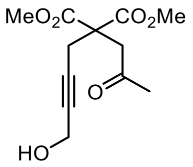

S11

Compound **S11**.
100.6 MHz ^{13}C NMR spectrum
$CDCl_3$

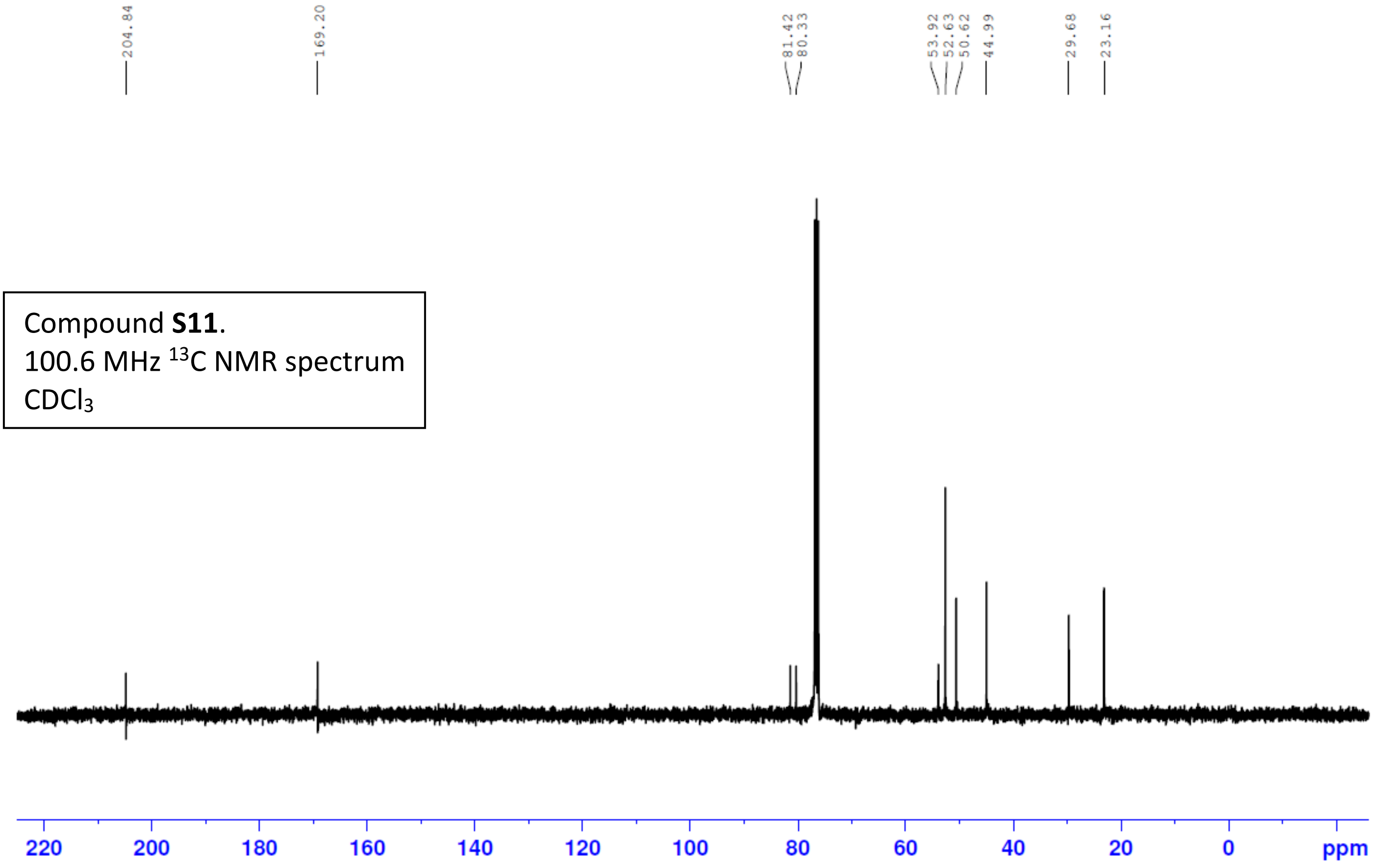

Compound **S12**.
400.13 MHz ^{1}H NMR spectrum
$CDCl_3$

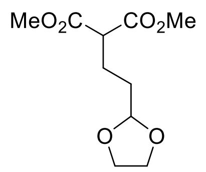

S12

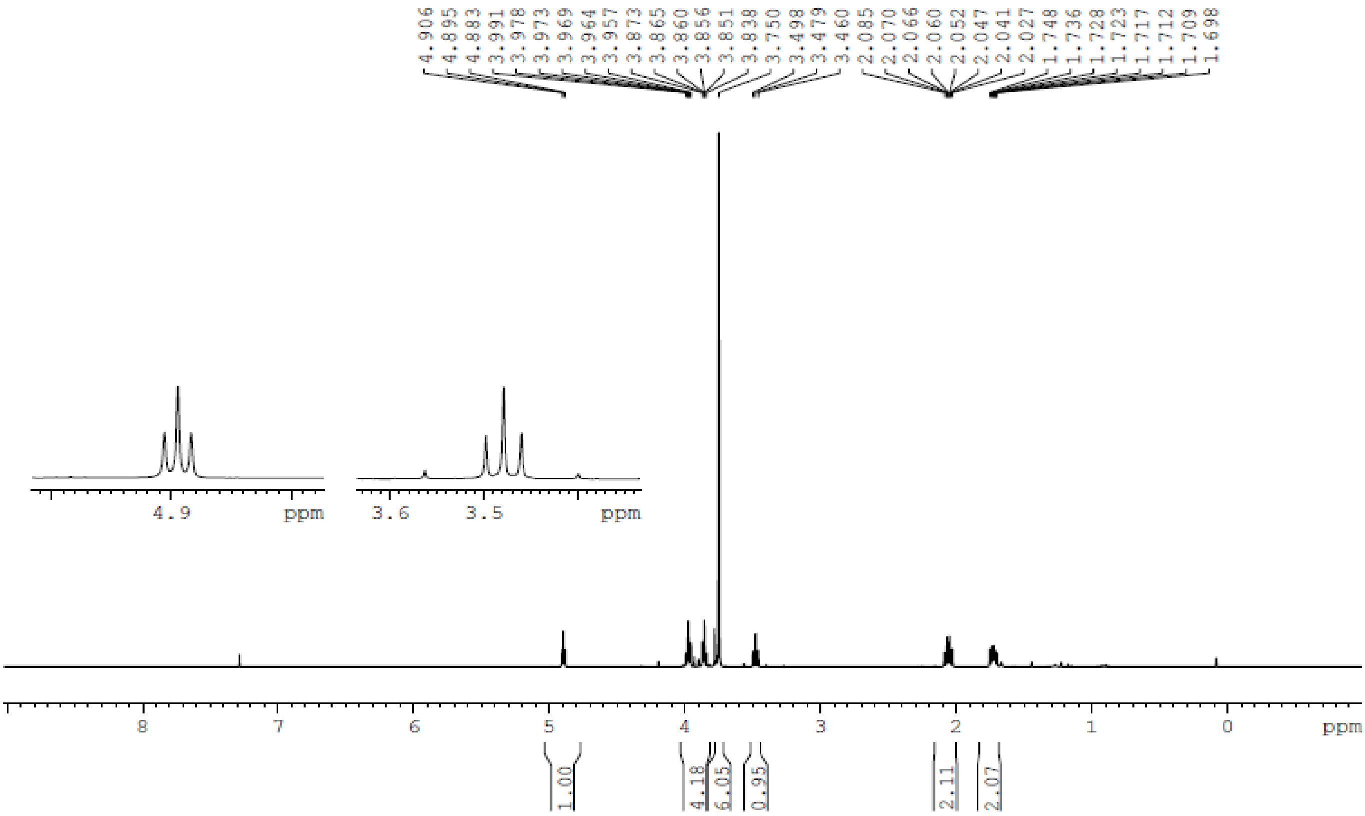

Compound **S12**.
100.6 MHz ^{13}C NMR spectrum
$CDCl_3$

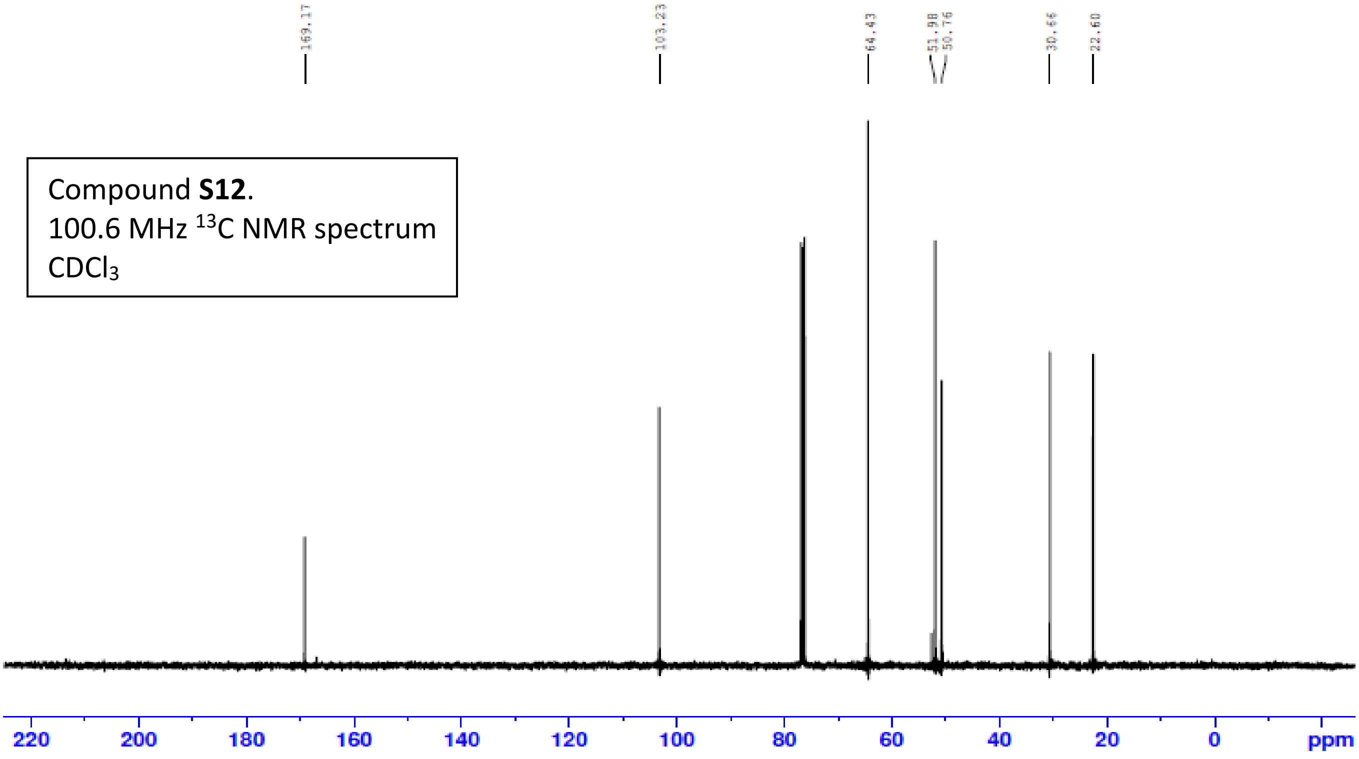

Compound **S13**.
400.13 MHz ^{1}H NMR spectrum
$CDCl_3$

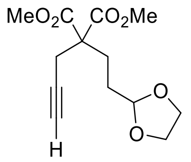

S13

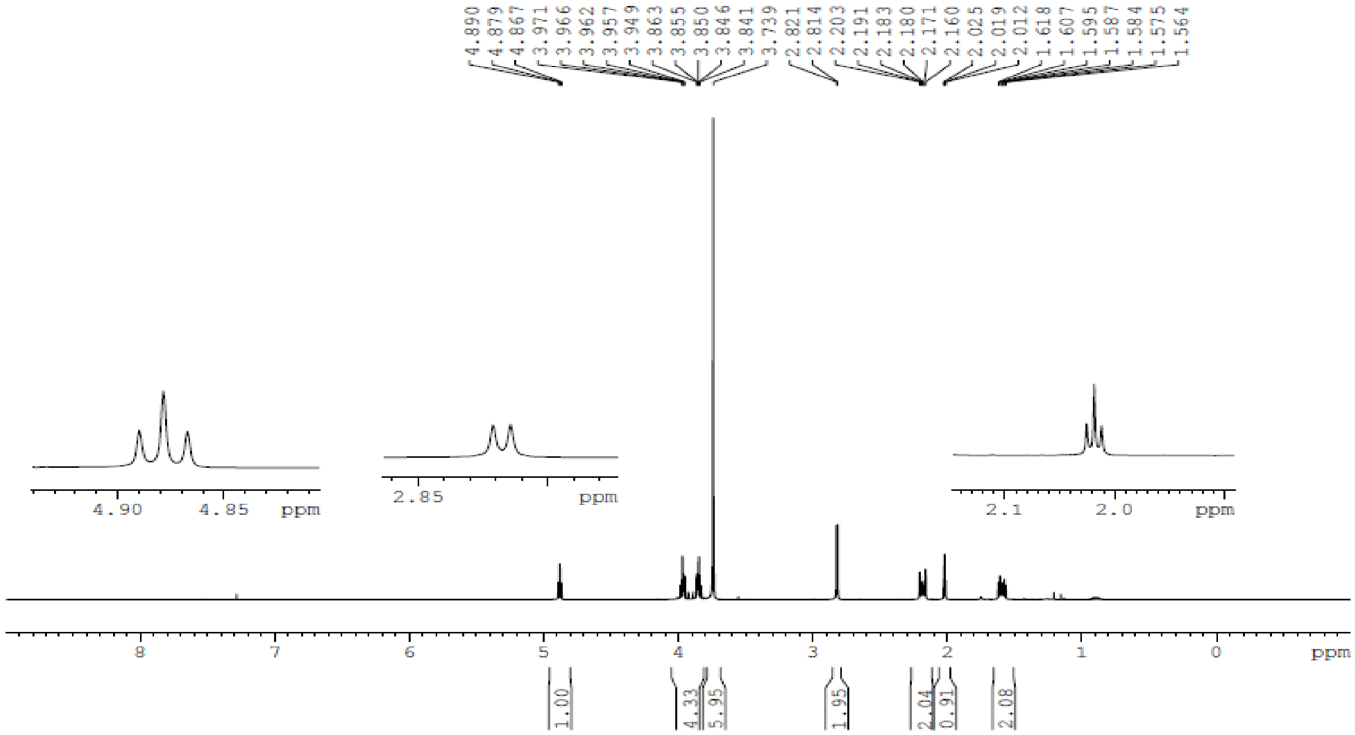

Compound **S13**.
100.6 MHz ^{13}C NMR spectrum
$CDCl_3$

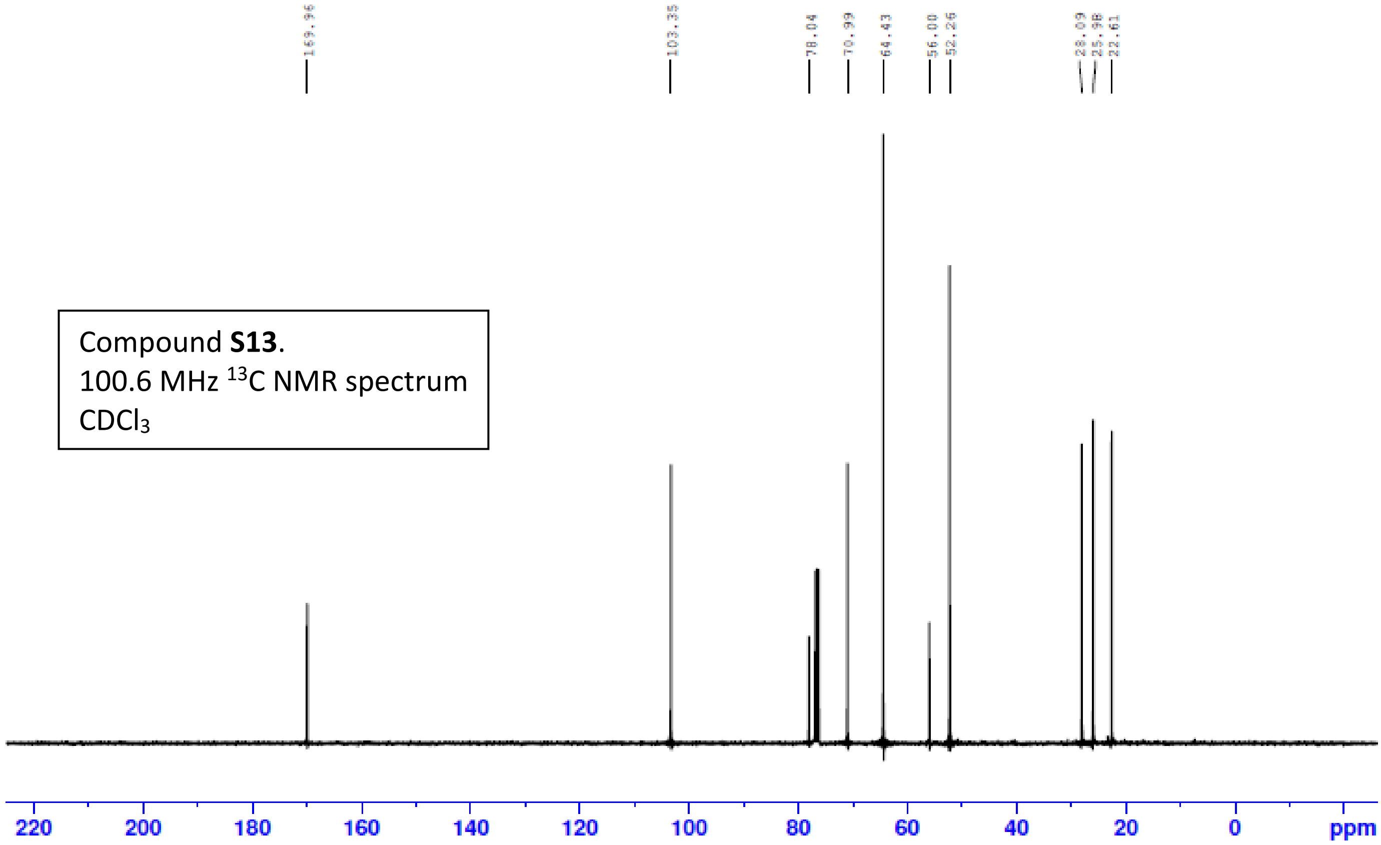

Compound **S17**.
400.13 MHz ^{1}H NMR spectrum
$CDCl_3$

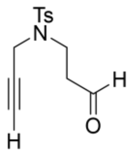

S17

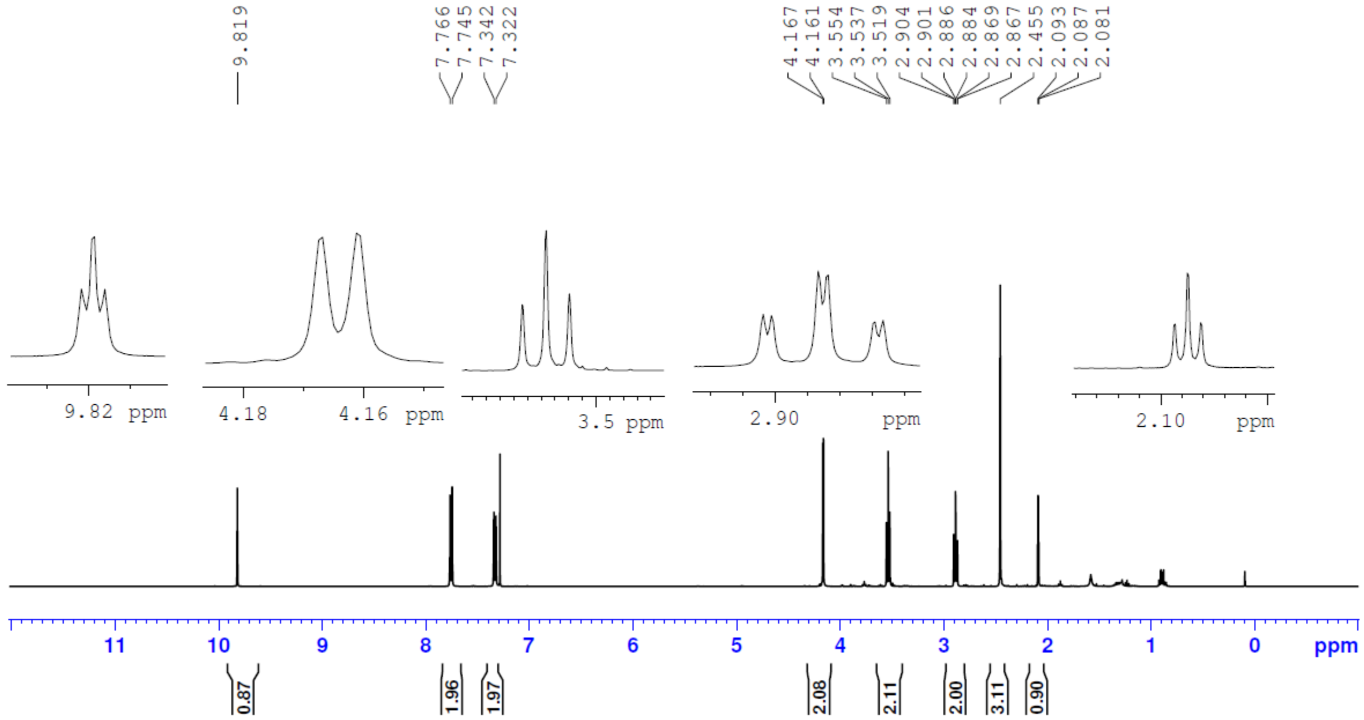

Compound **S17**.
100.6 MHz ^{13}C NMR spectrum
$CDCl_3$

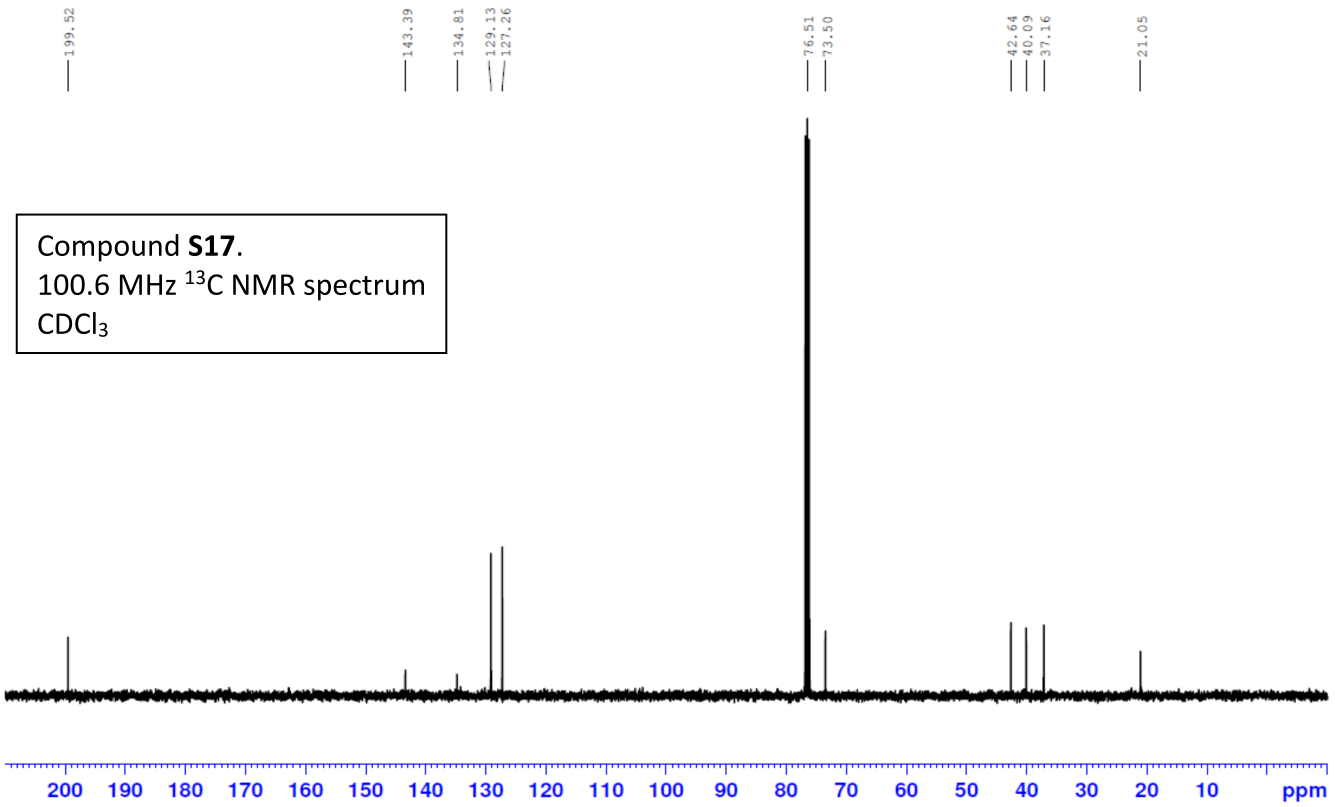

Compound **S18**.
400.13 MHz ^{1}H NMR spectrum
$CDCl_3$

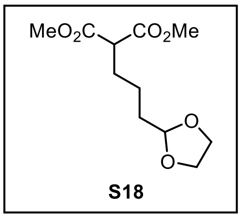

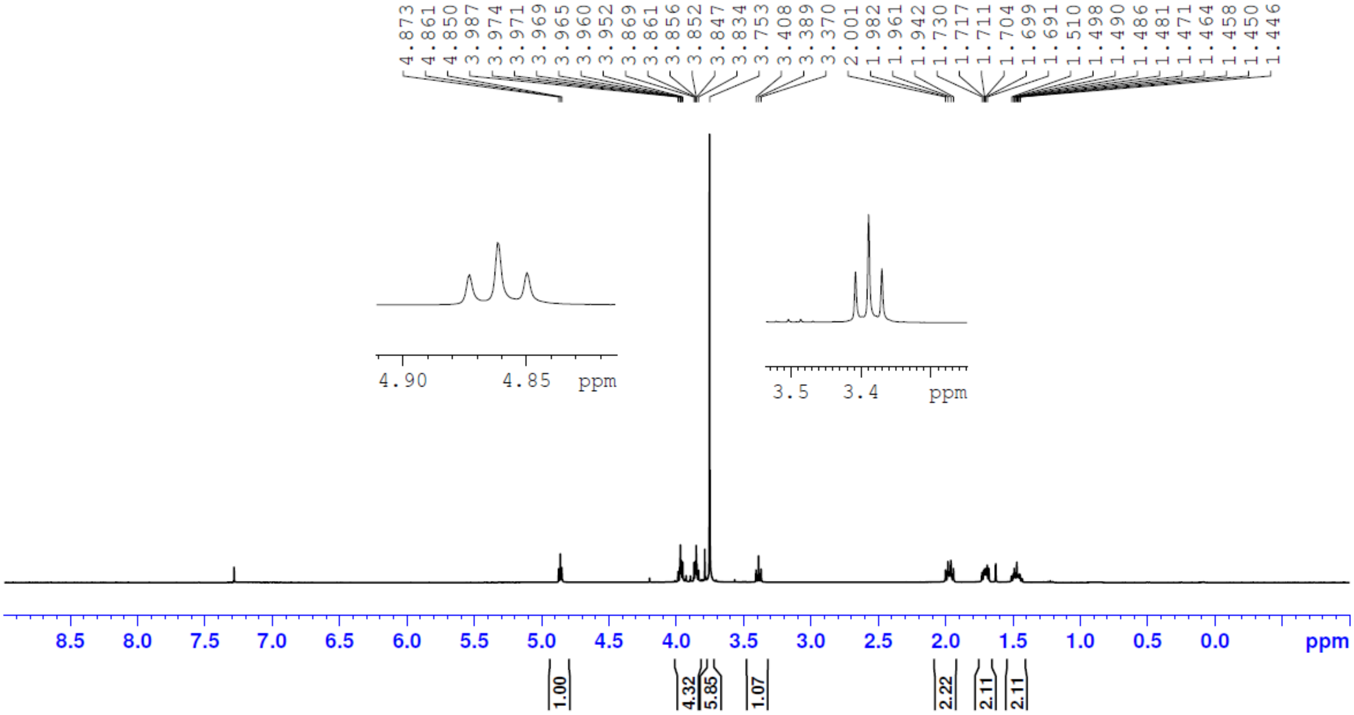

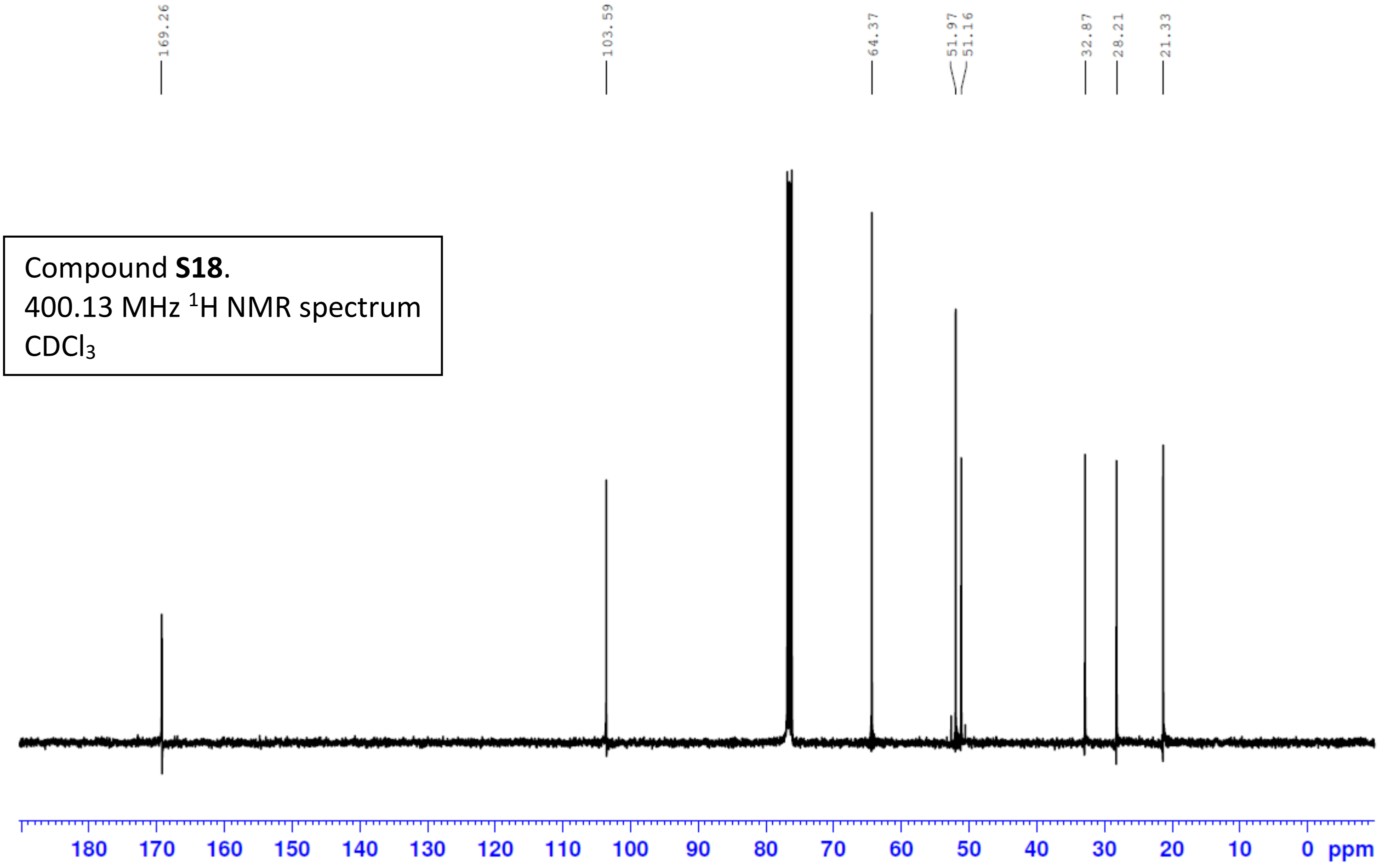

Compound **S18**.
400.13 MHz ^{1}H NMR spectrum
$CDCl_3$

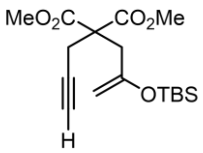

2

Compound **2**.
400.13 MHz ^{1}H NMR spectrum
$CDCl_3$

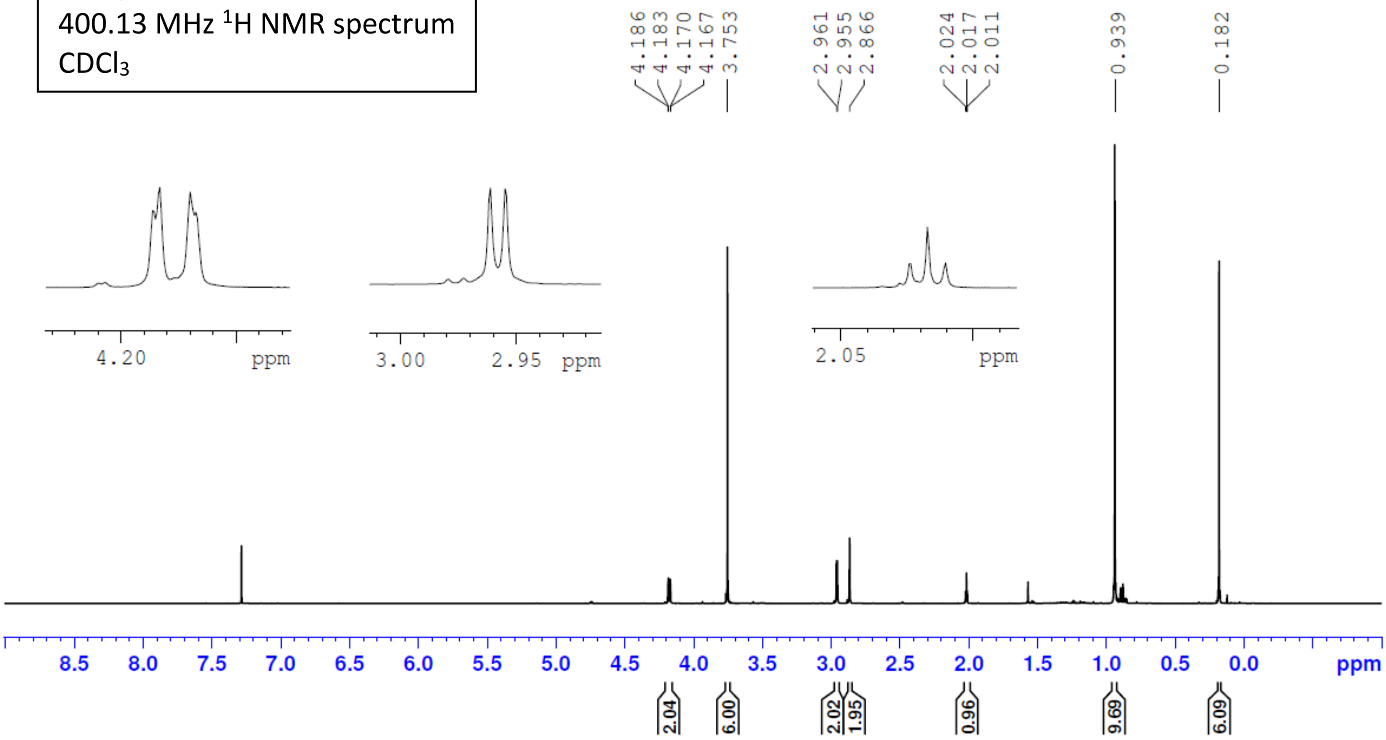

Compound **2**.
100.6 MHz ^{13}C NMR spectrum
$CDCl_3$

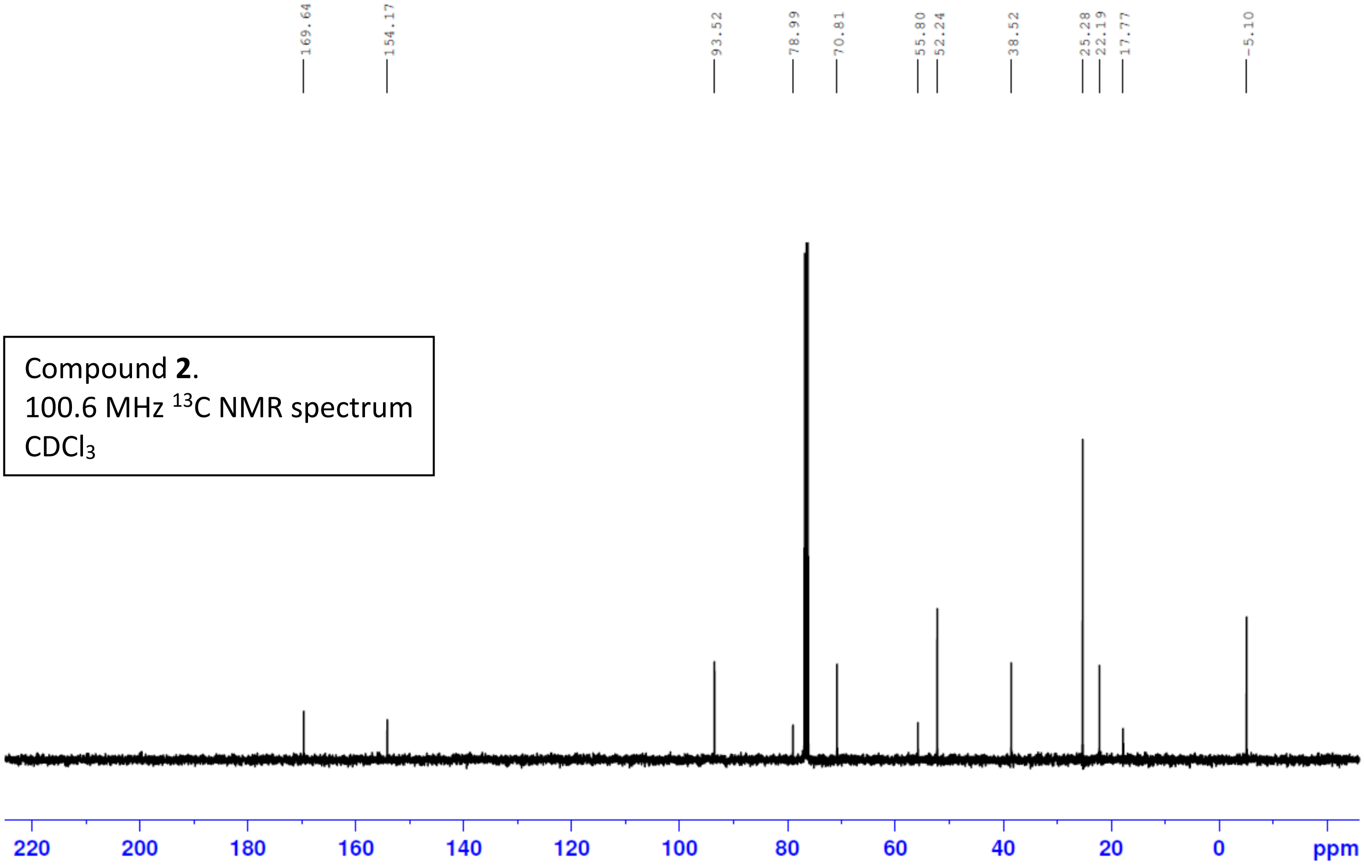

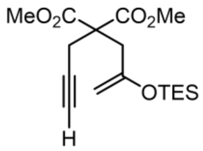

5a

Compound **5a**.
400.13 MHz ^{1}H NMR spectrum
$CDCl_3$

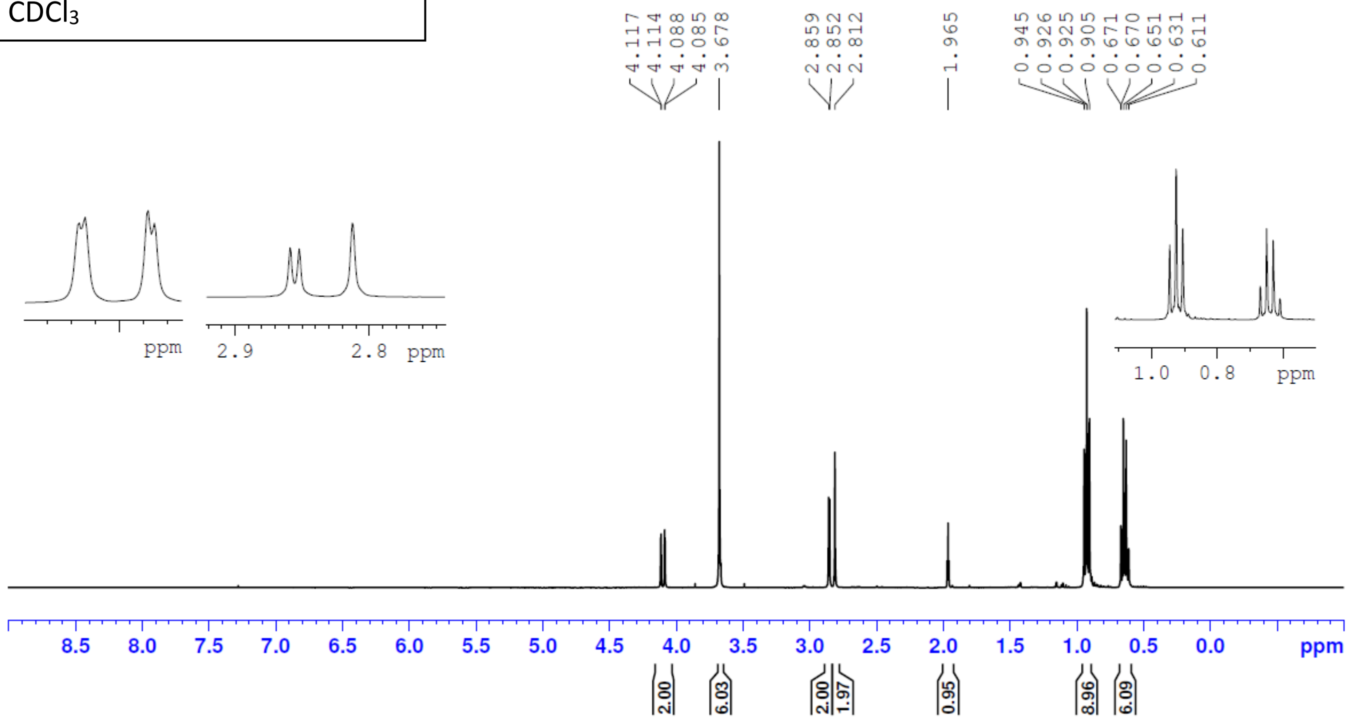

Compound **5a**.
100.6 MHz ^{13}C NMR spectrum
$CDCl_3$

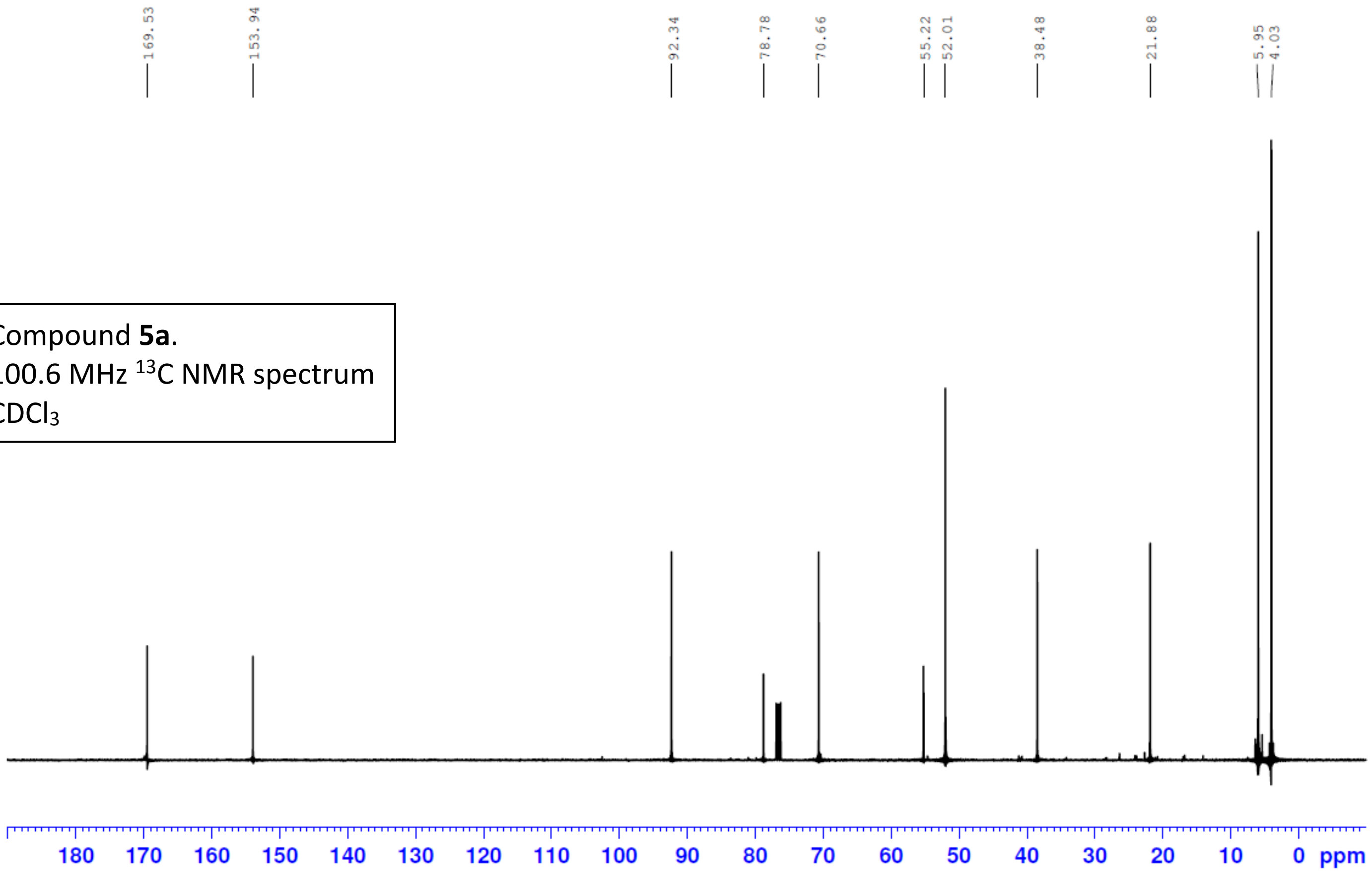

Compound **5b**.
400.13 MHz ^{1}H NMR spectrum
$CDCl_3$

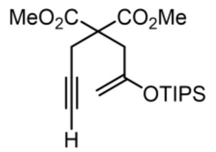

5b

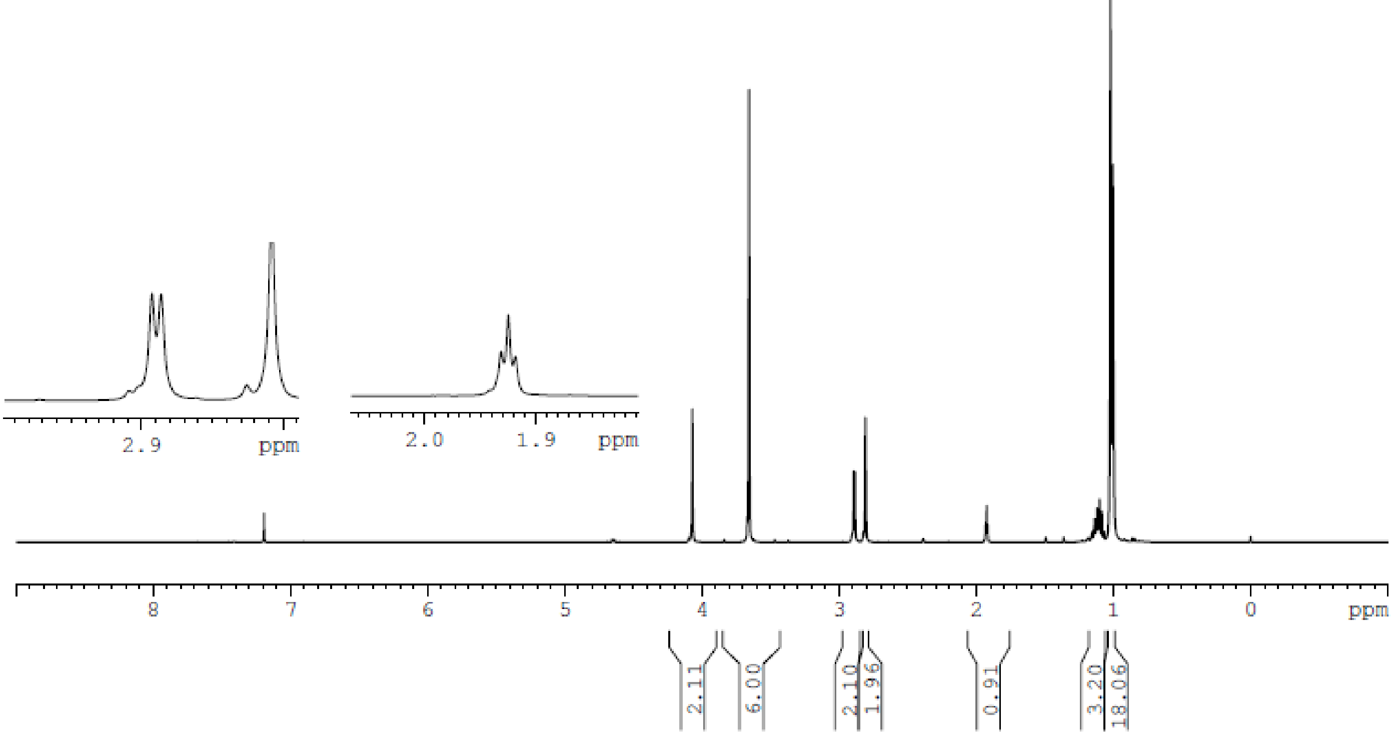

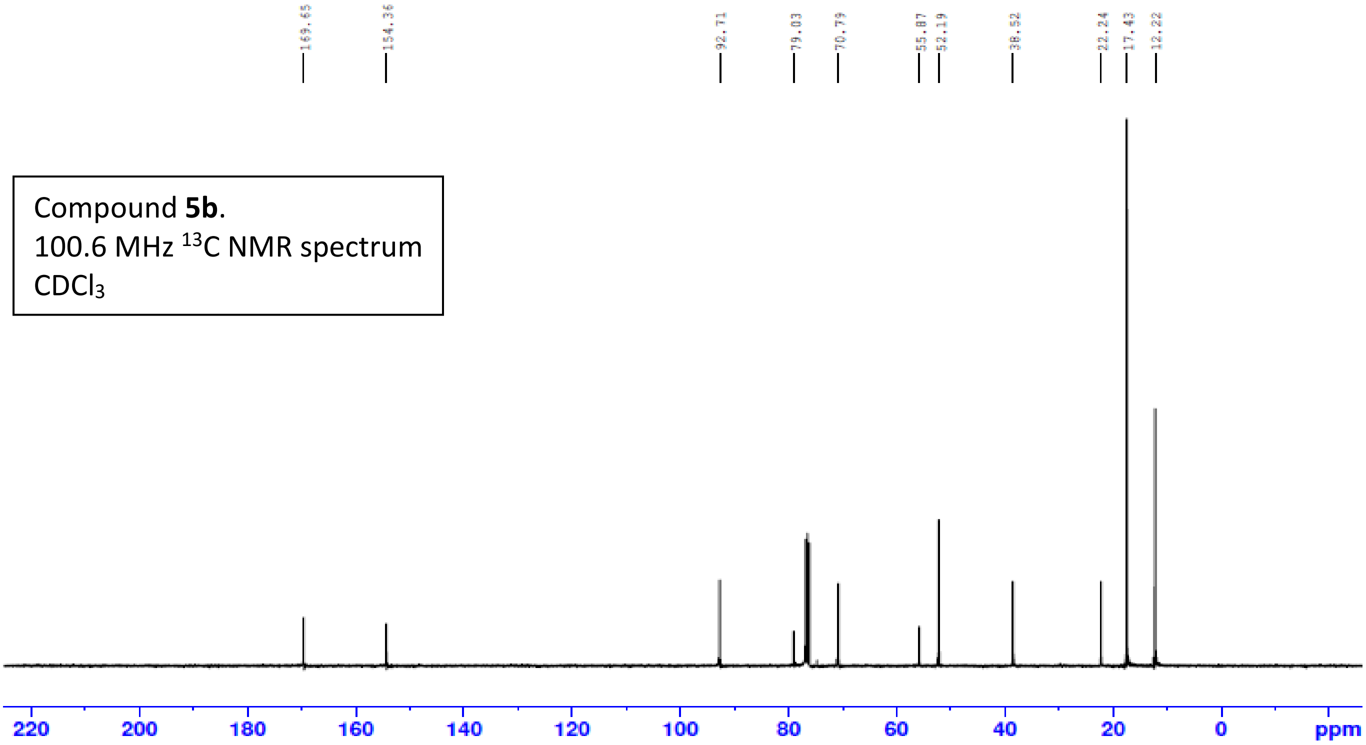

Compound **5b**.
100.6 MHz ^{13}C NMR spectrum
$CDCl_3$

Compound **S21**.
400.13 MHz ^{1}H NMR spectrum
$CDCl_3$

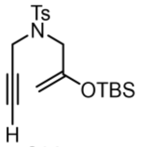

S21

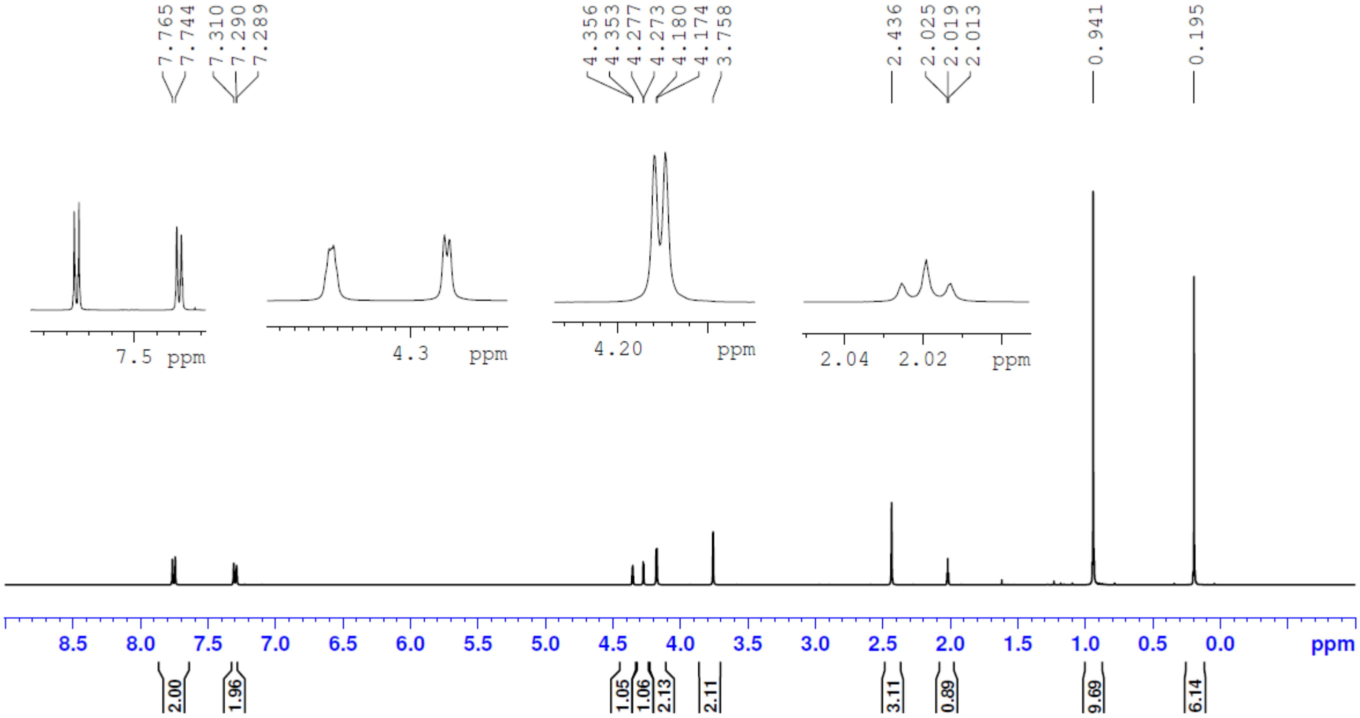

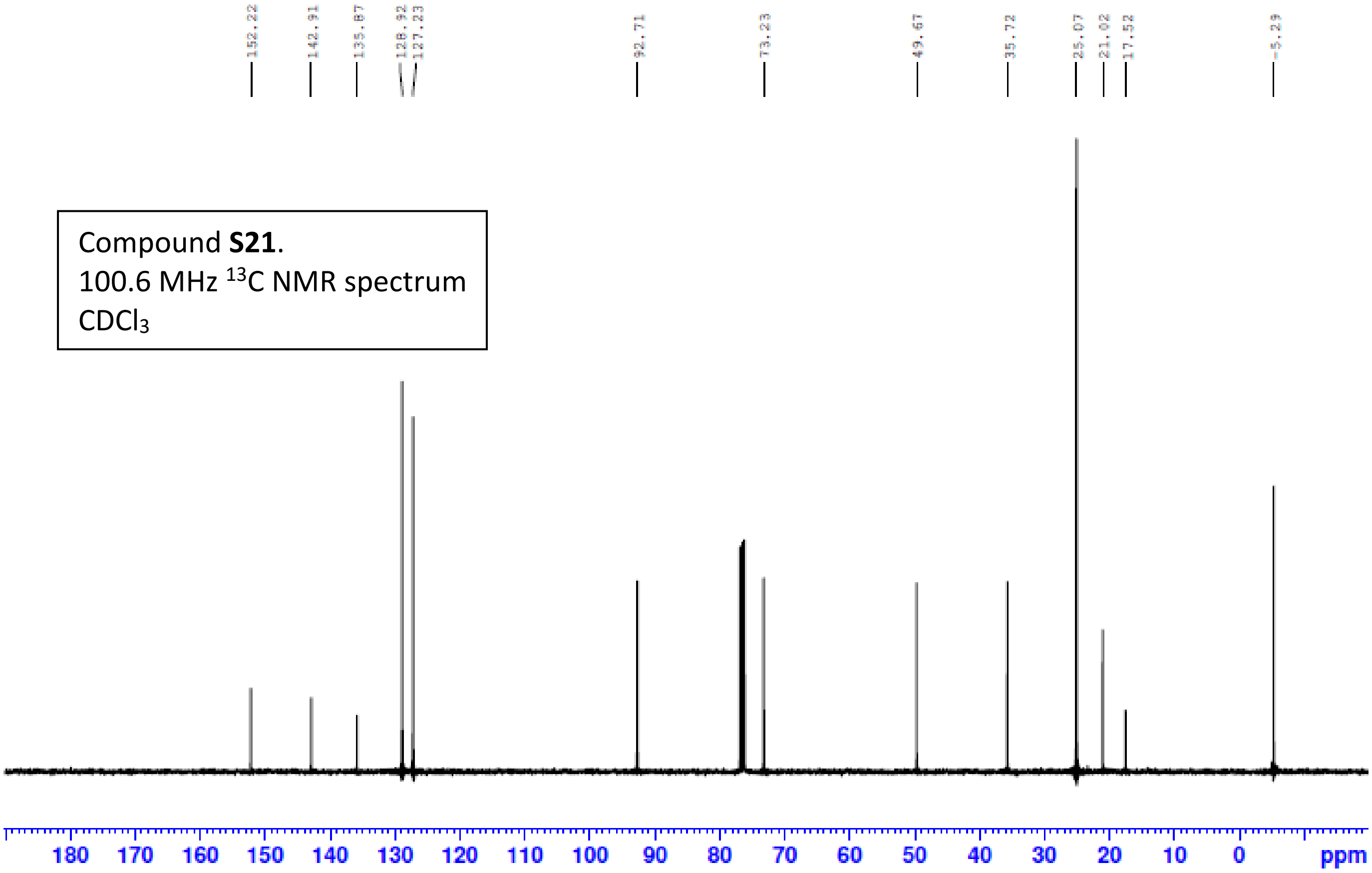

Compound **S21**.
100.6 MHz ^{13}C NMR spectrum
$CDCl_3$

Compound **S22**.
400.13 MHz ^{1}H NMR spectrum
$CDCl_3$

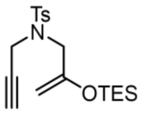

S22

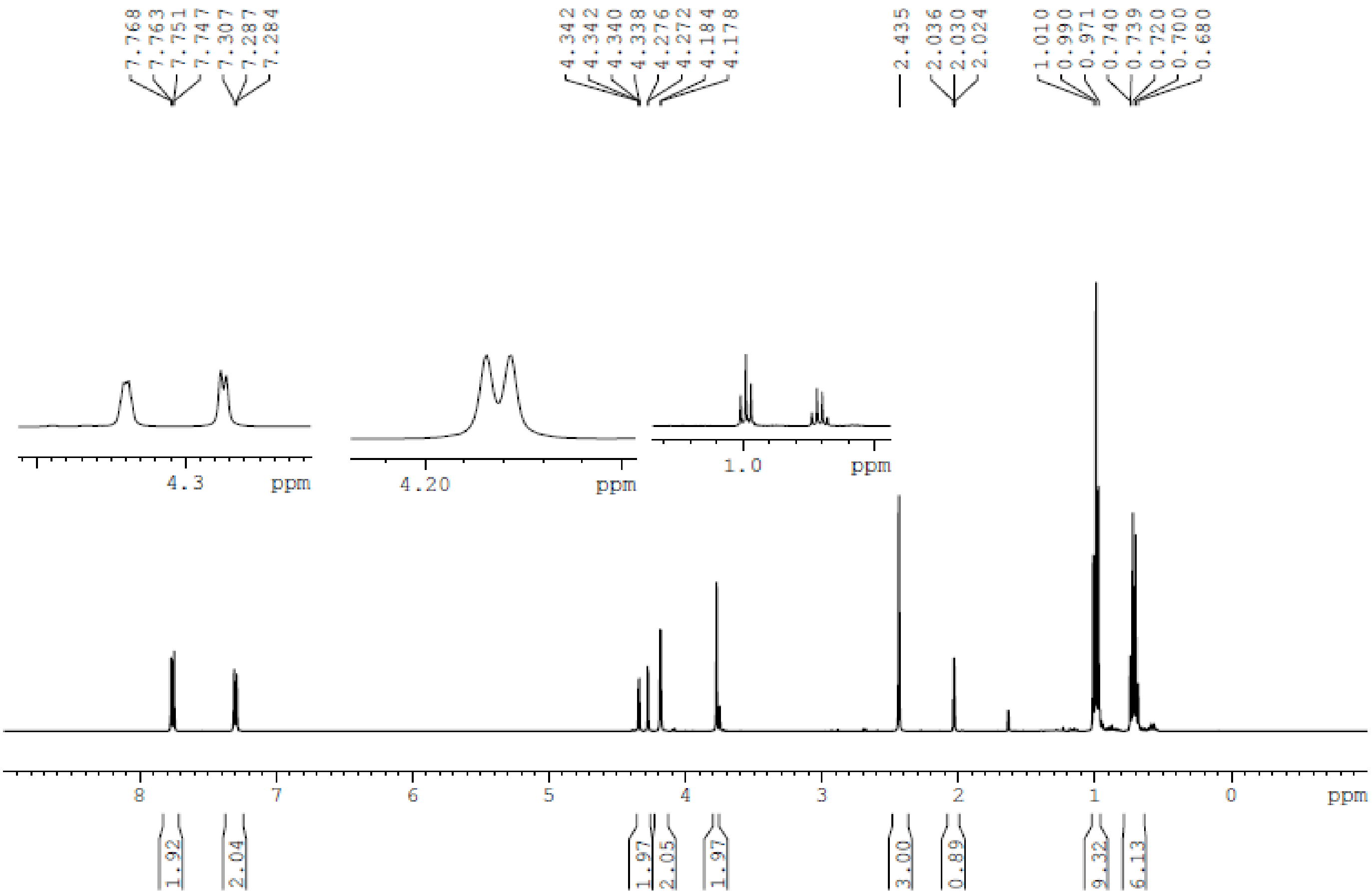

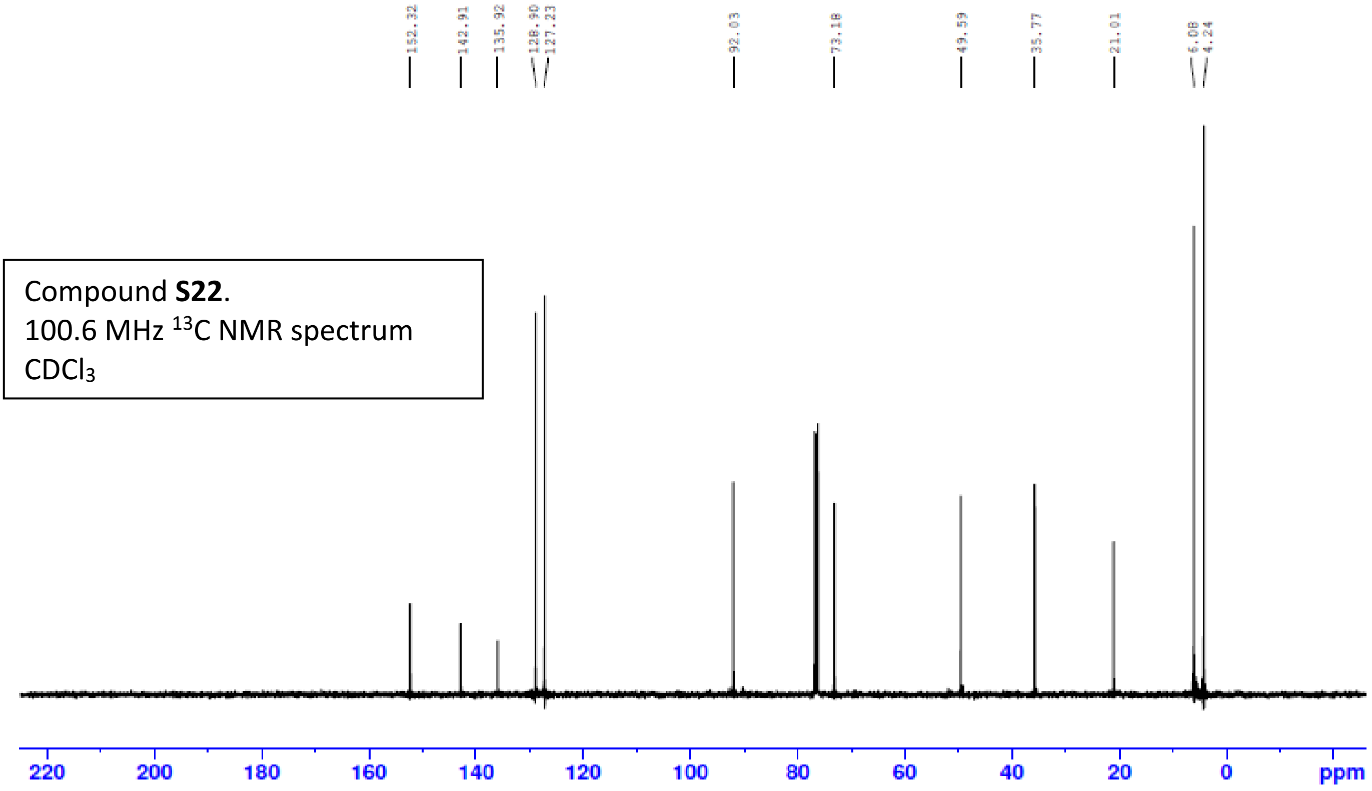

Compound **S22**.
100.6 MHz ^{13}C NMR spectrum
$CDCl_3$

Compound **S23**.
400.13 MHz ^{1}H NMR spectrum
$CDCl_3$

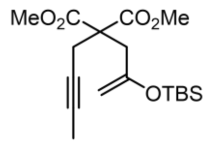

S23

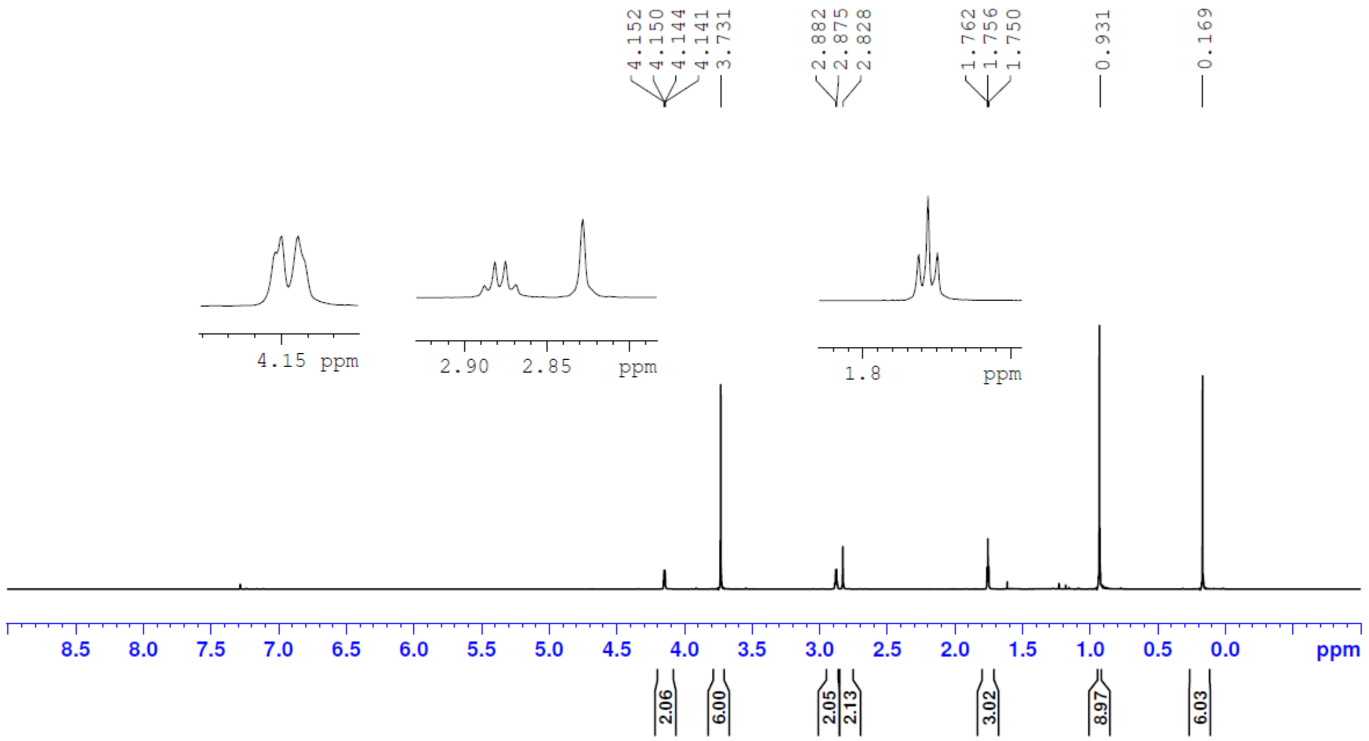

Compound **S23**.
100.6 MHz ^{13}C NMR spectrum
$CDCl_3$

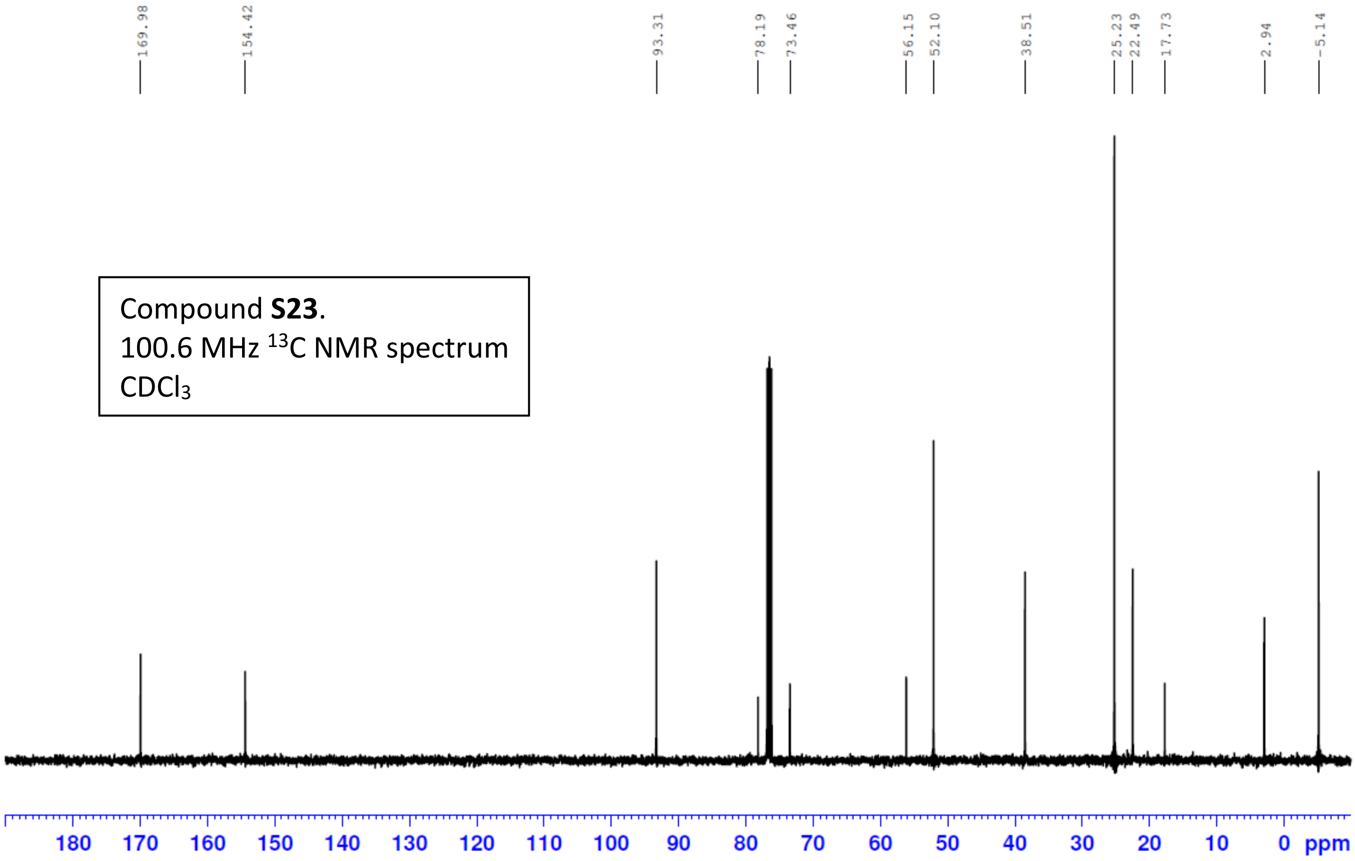

Compound **S24**.
400.13 MHz ^{1}H NMR spectrum
$CDCl_3$

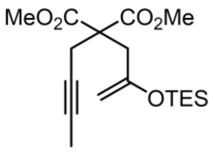

S24

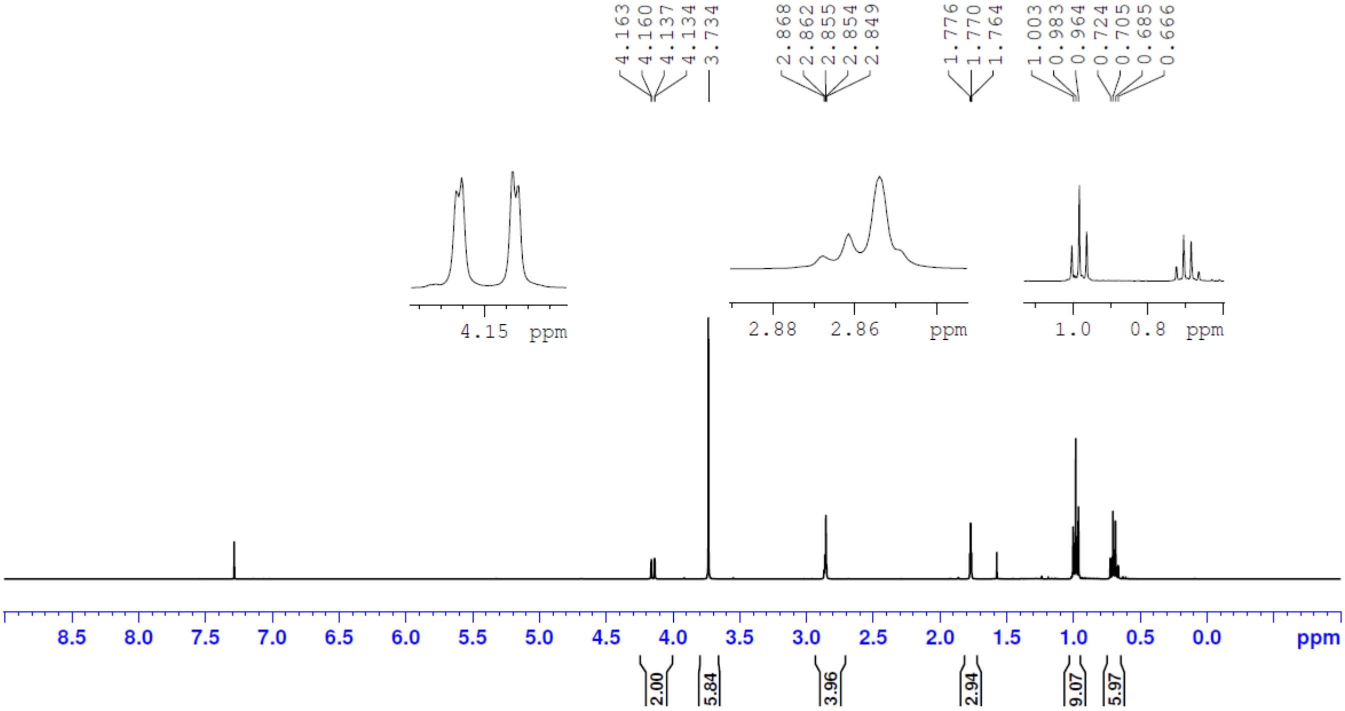

Compound **S24**.
100.6 MHz ^{13}C NMR spectrum
$CDCl_3$

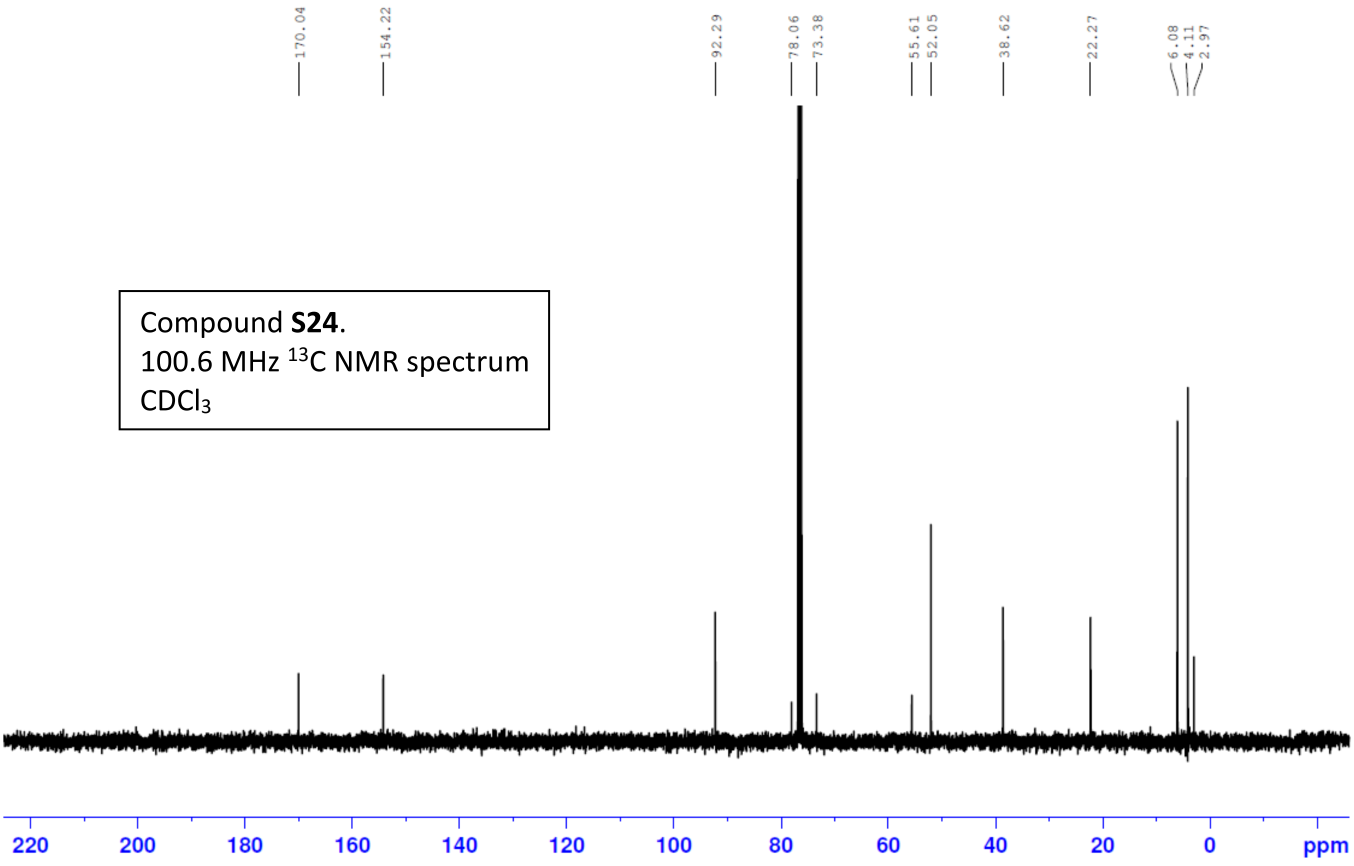

Compound **S25**.
400.13 MHz ^{1}H NMR spectrum
$CDCl_3$

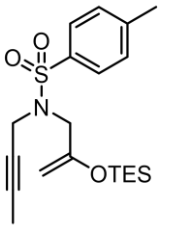

S25

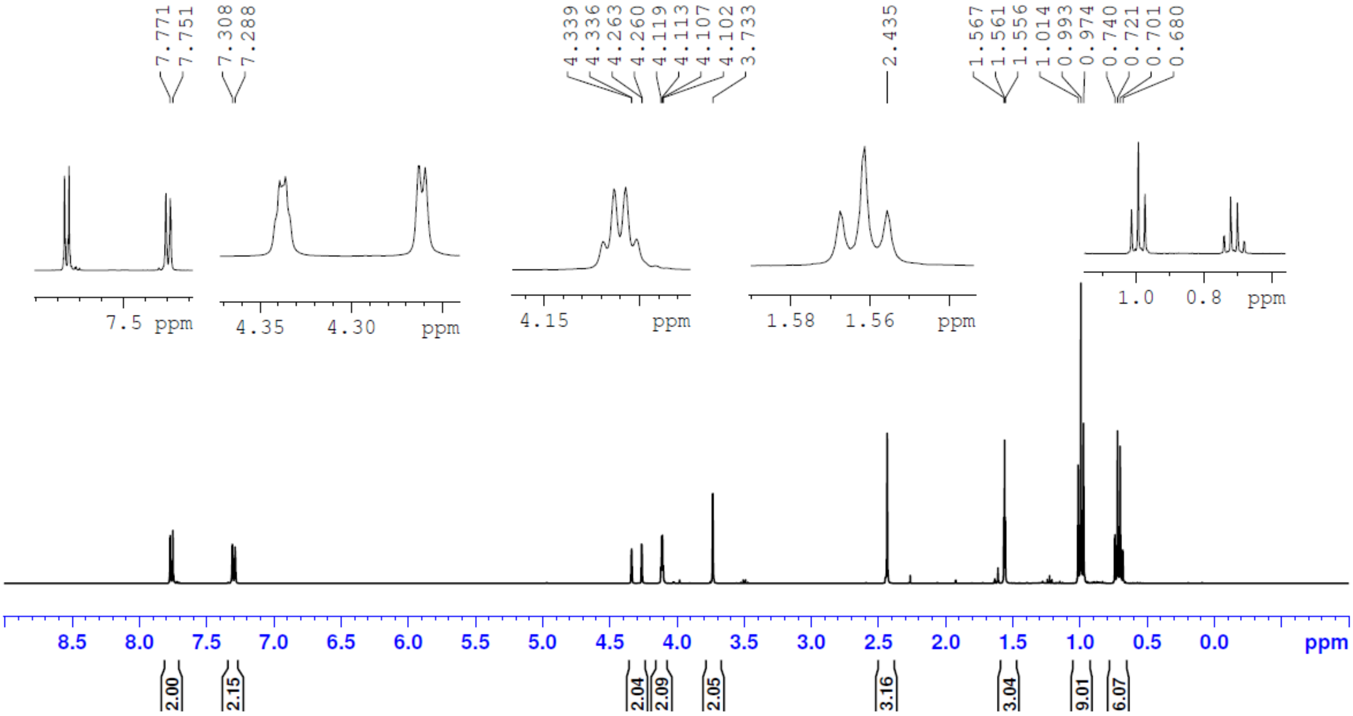

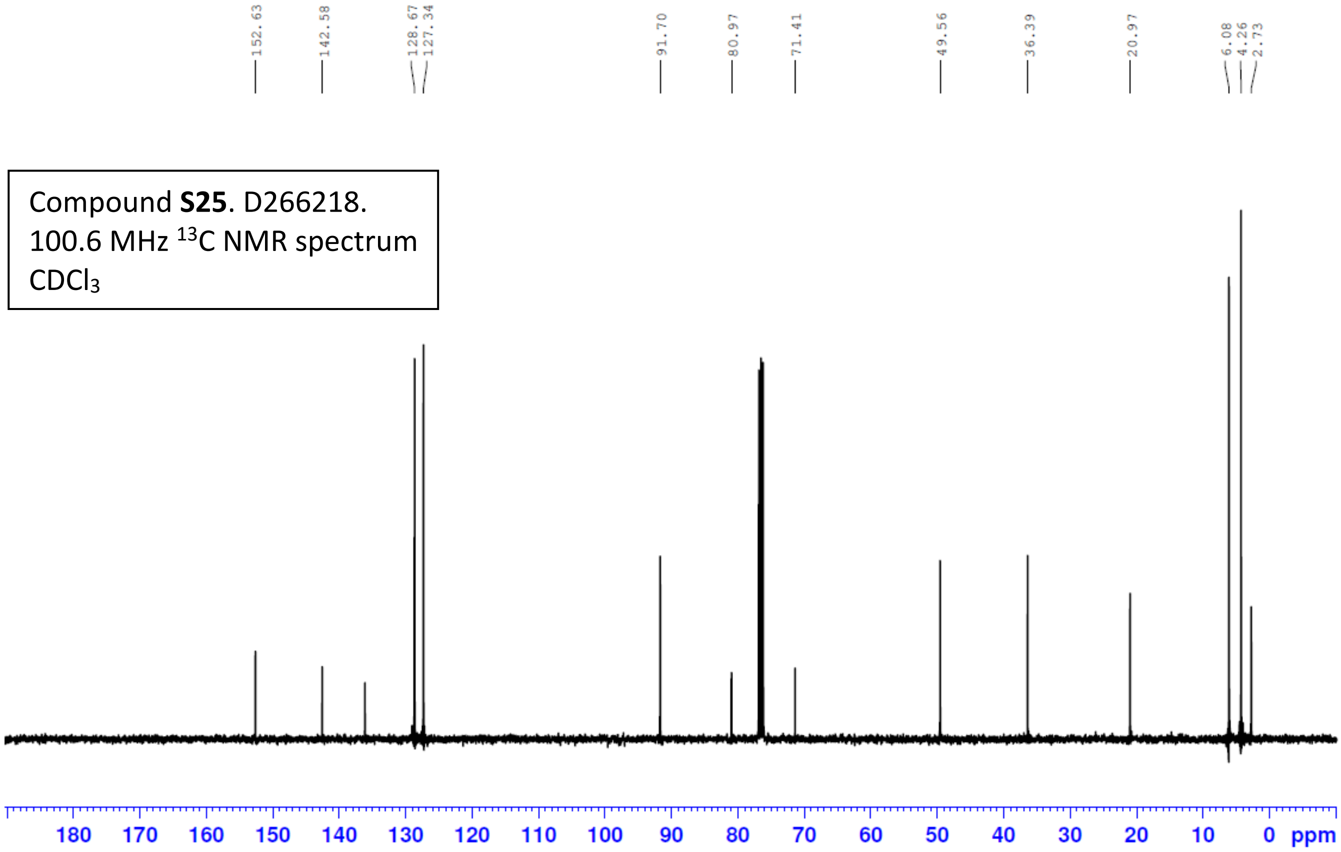

Compound **S25**. D266218.
100.6 MHz ^{13}C NMR spectrum
$CDCl_3$

Compound **S26**.
400.13 MHz ^{1}H NMR spectrum
$CDCl_3$

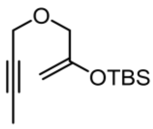

S26

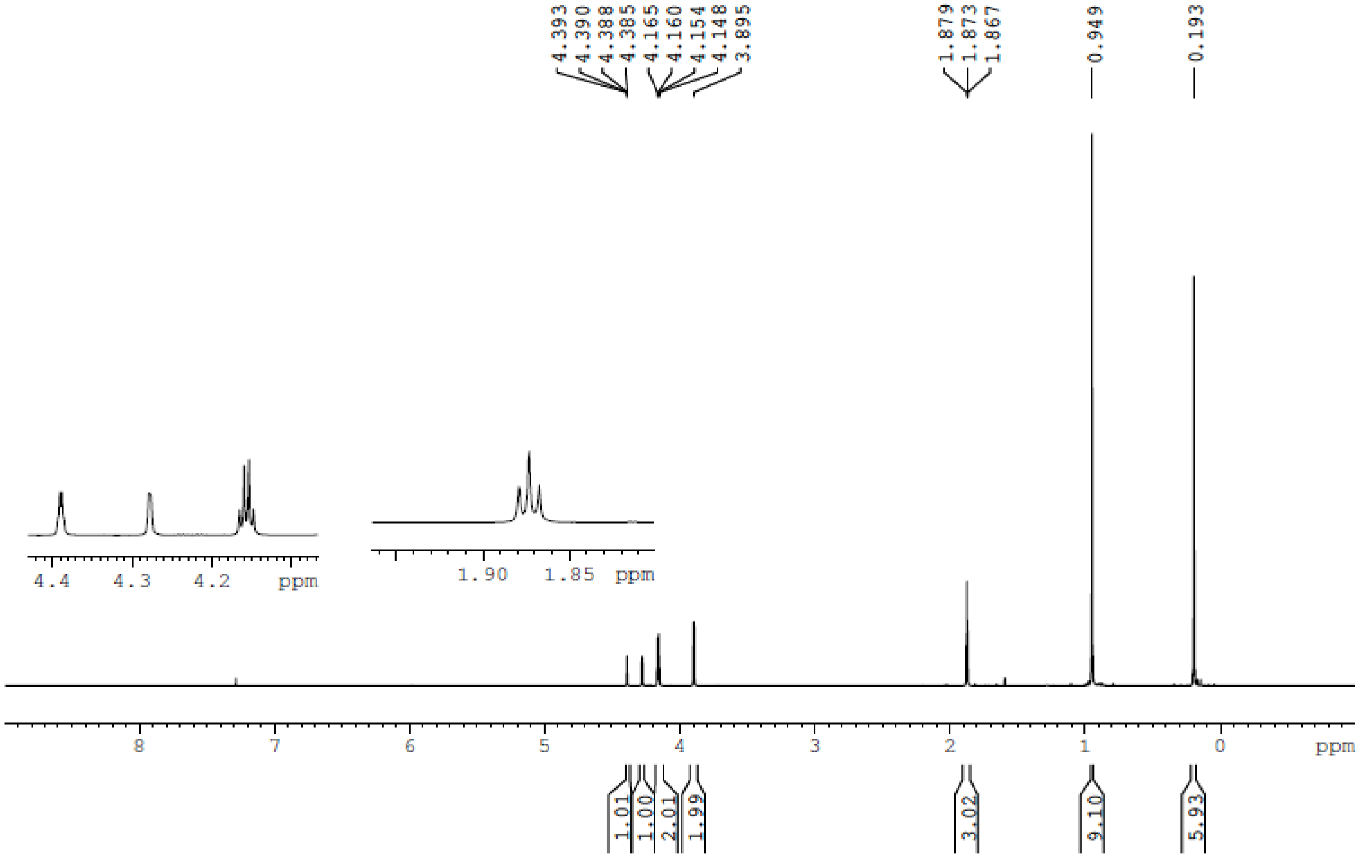

Compound **S26**.
100.6 MHz ^{13}C NMR spectrum
$CDCl_3$

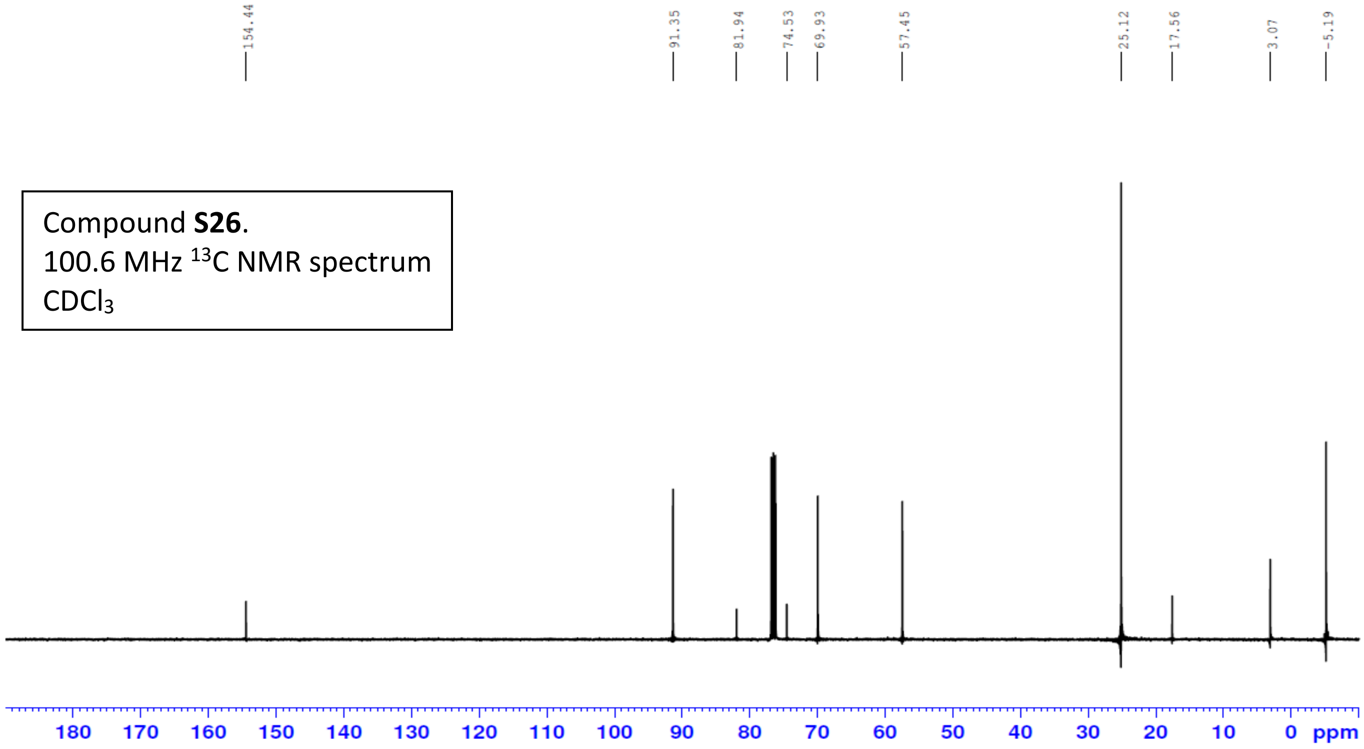

Compound **S27**.
400.13 MHz ^{1}H NMR spectrum
$CDCl_3$

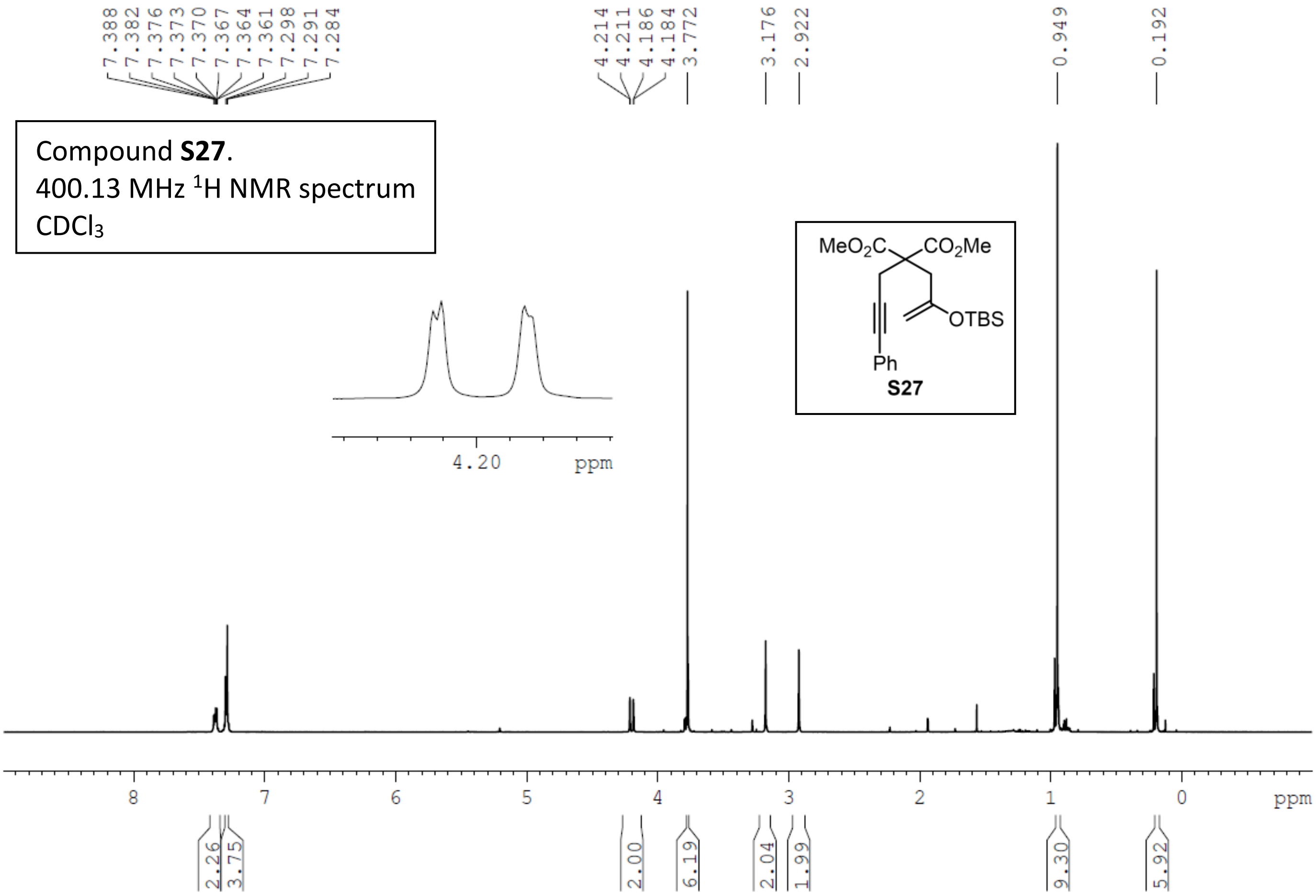

Compound **S27**.
100.6 MHz ^{13}C NMR spectrum
$CDCl_3$

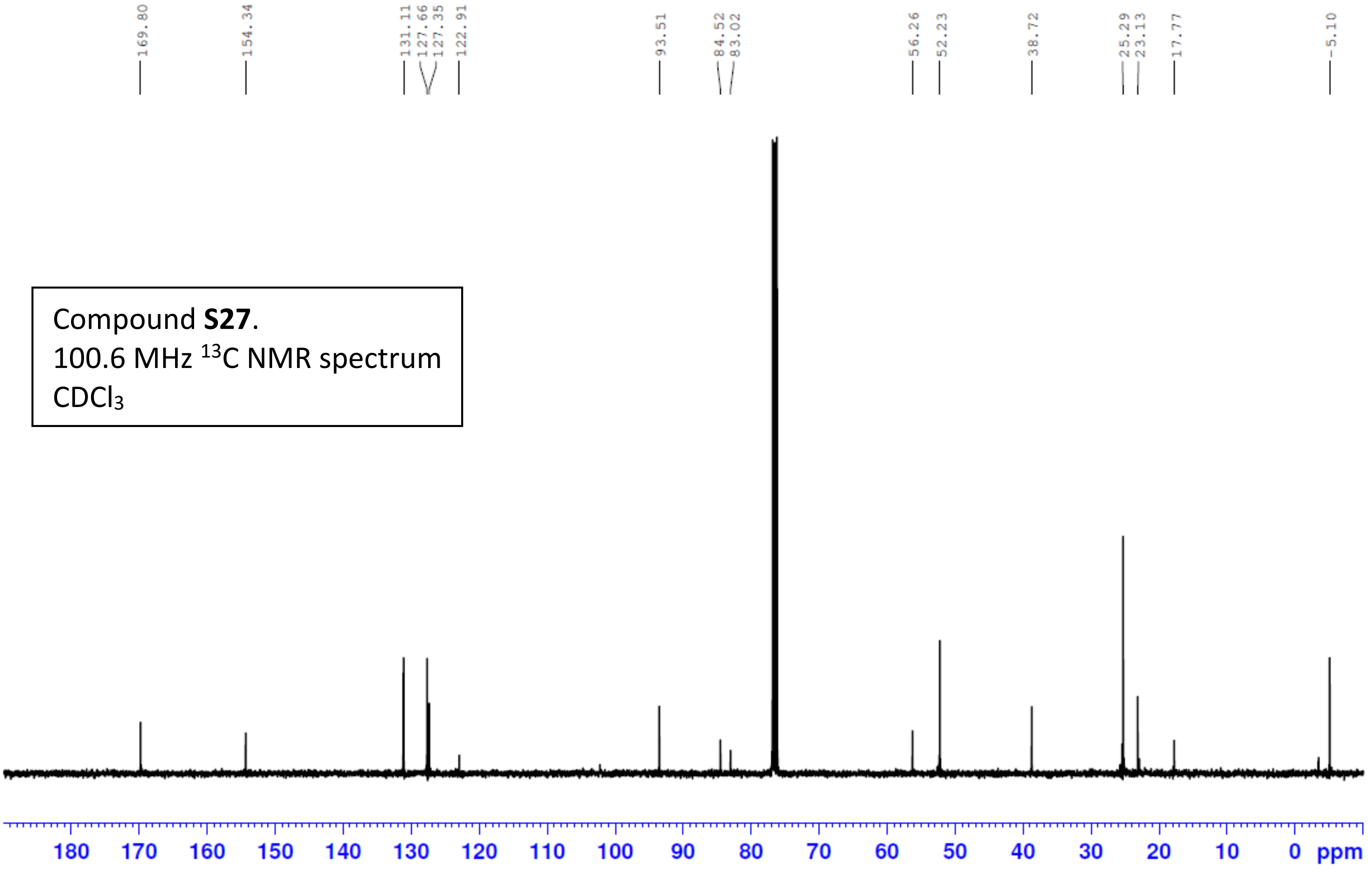

Compound **S28**.
400.13 MHz ^{1}H NMR spectrum
$CDCl_3$

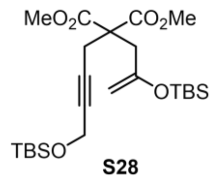

S28

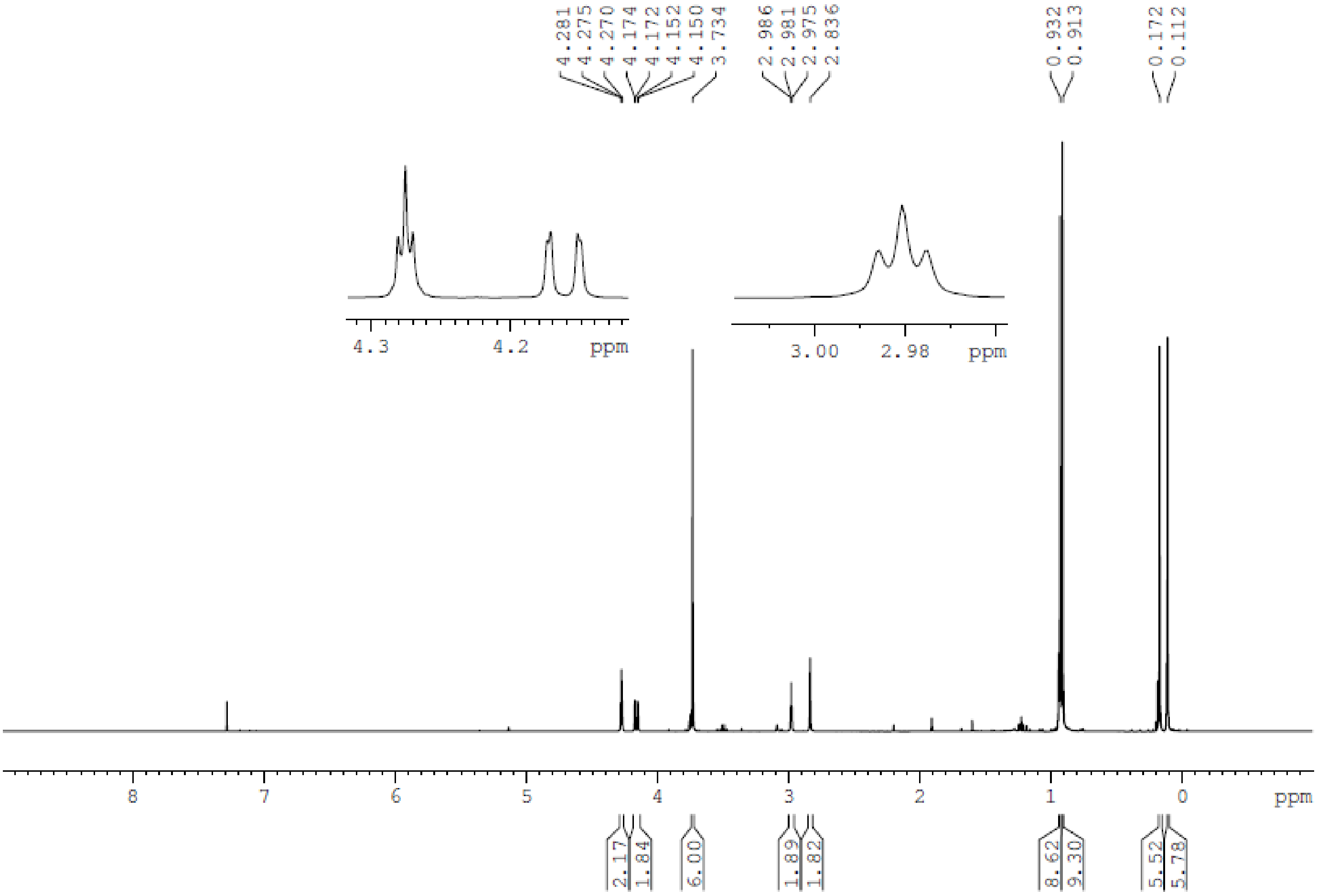

Compound **S28**.
100.6 MHz ^{13}C NMR spectrum
$CDCl_3$

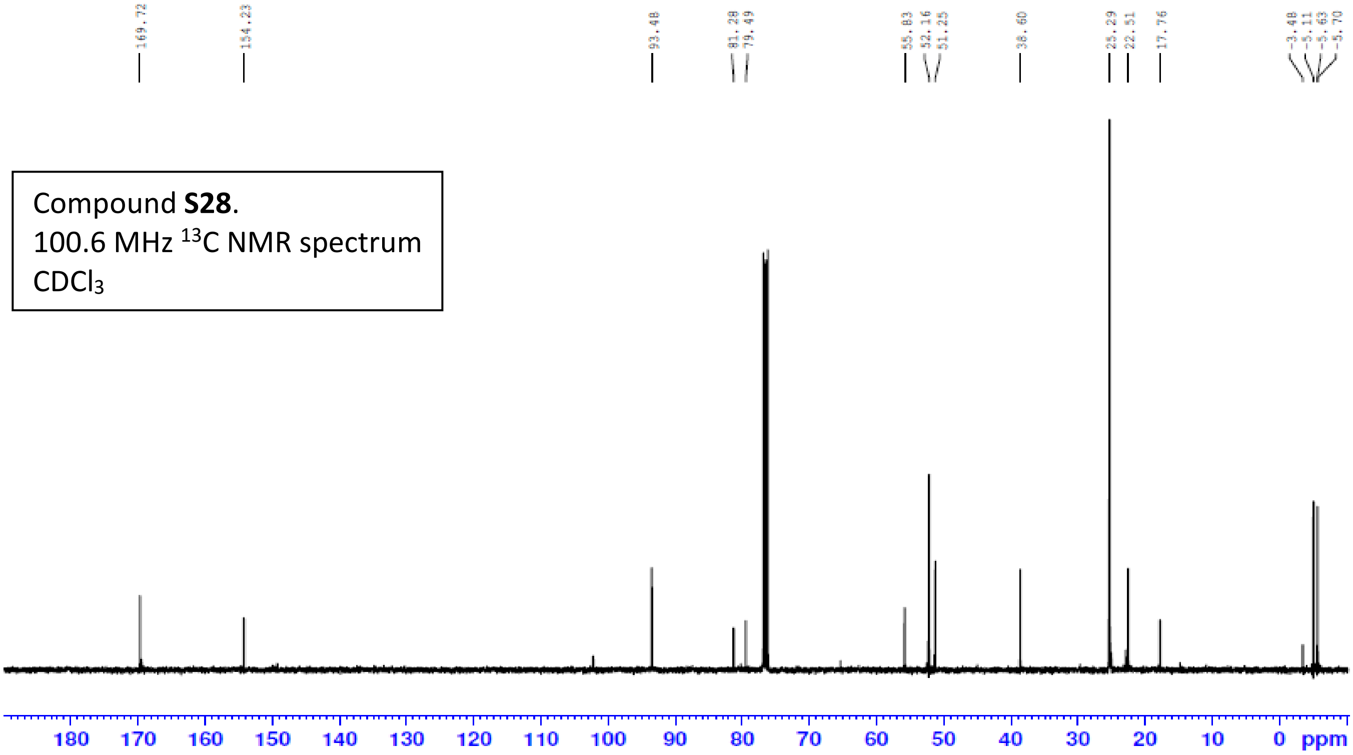

Compound **S30**.
400.13 MHz ^{1}H NMR spectrum
$CDCl_3$

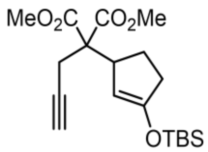

S30

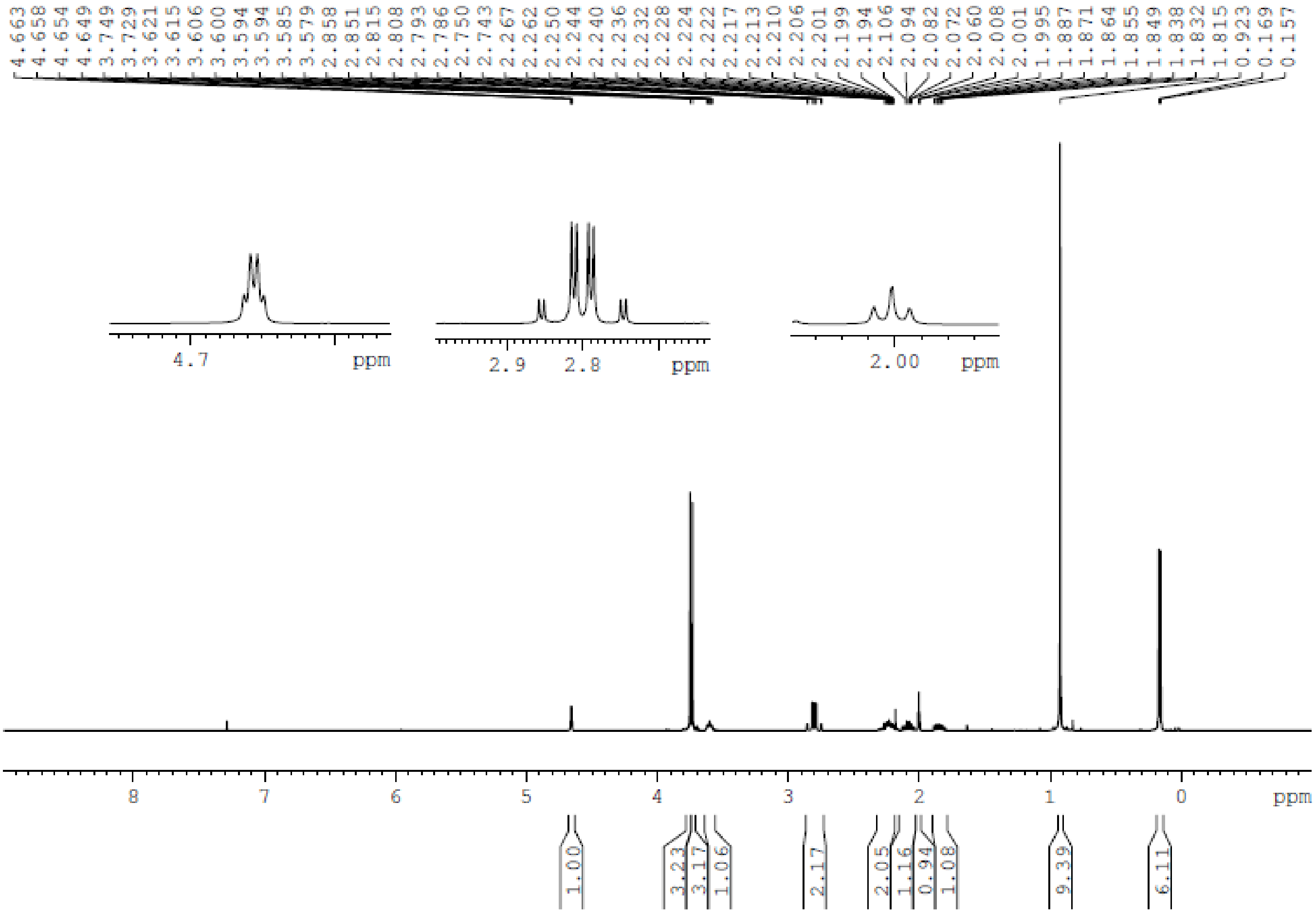

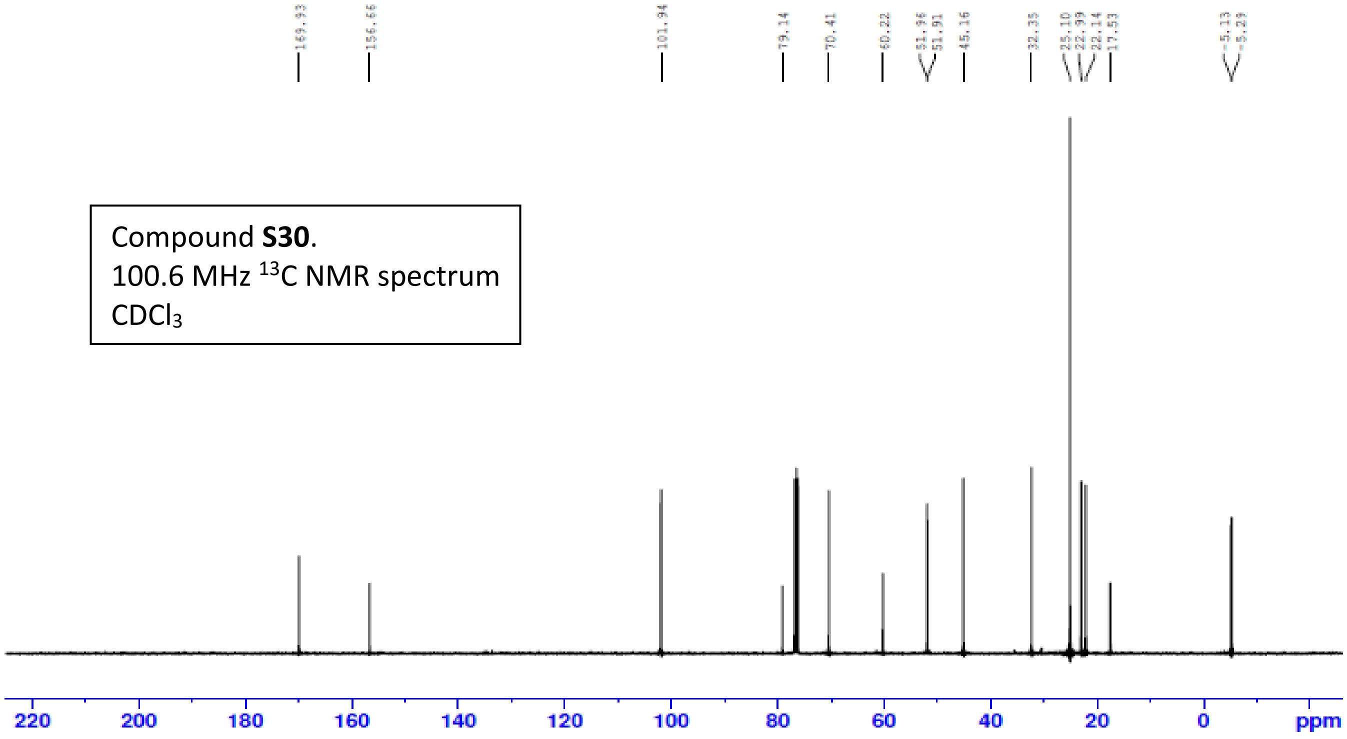

Compound **S30**.
100.6 MHz ^{13}C NMR spectrum
$CDCl_3$

Compound **S31**.
400.13 MHz ^{1}H NMR spectrum
$CDCl_3$

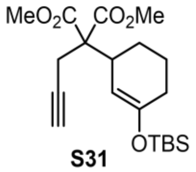

S31

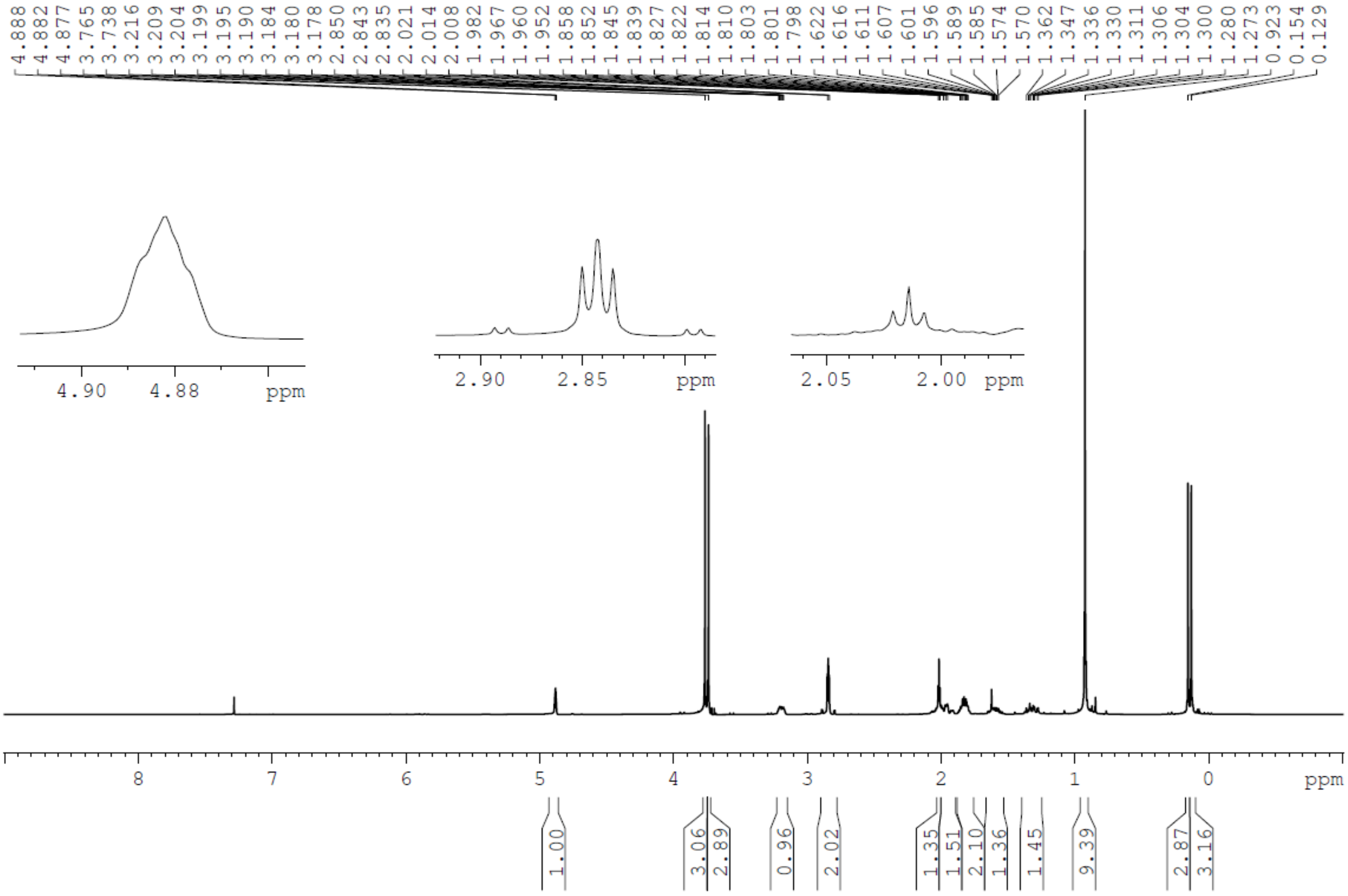

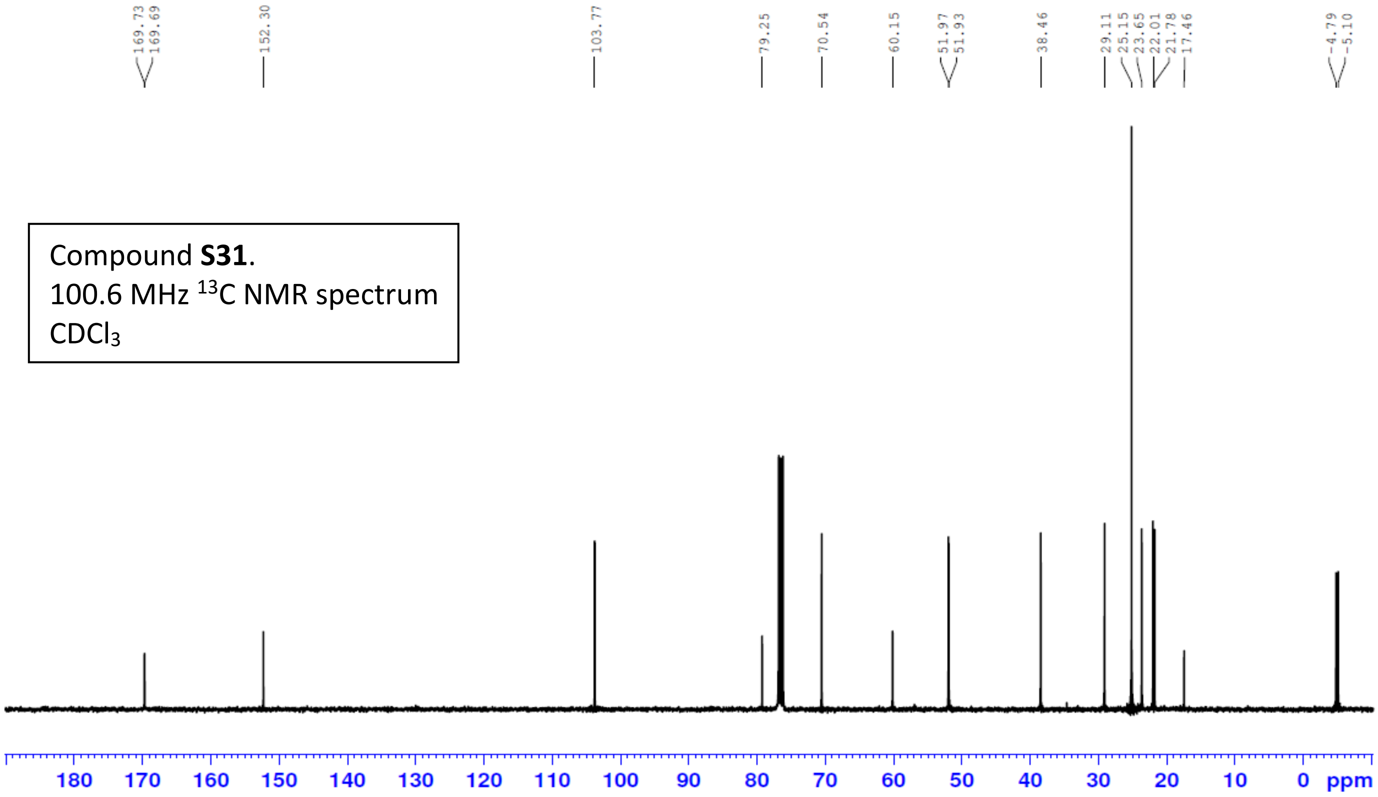

Compound **S31**.
100.6 MHz ^{13}C NMR spectrum
$CDCl_3$

Compound **S32**.
400.13 MHz ^{1}H NMR spectrum
$CDCl_3$

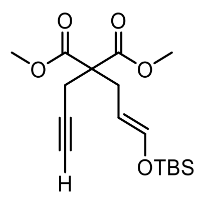

***trans*-S32**

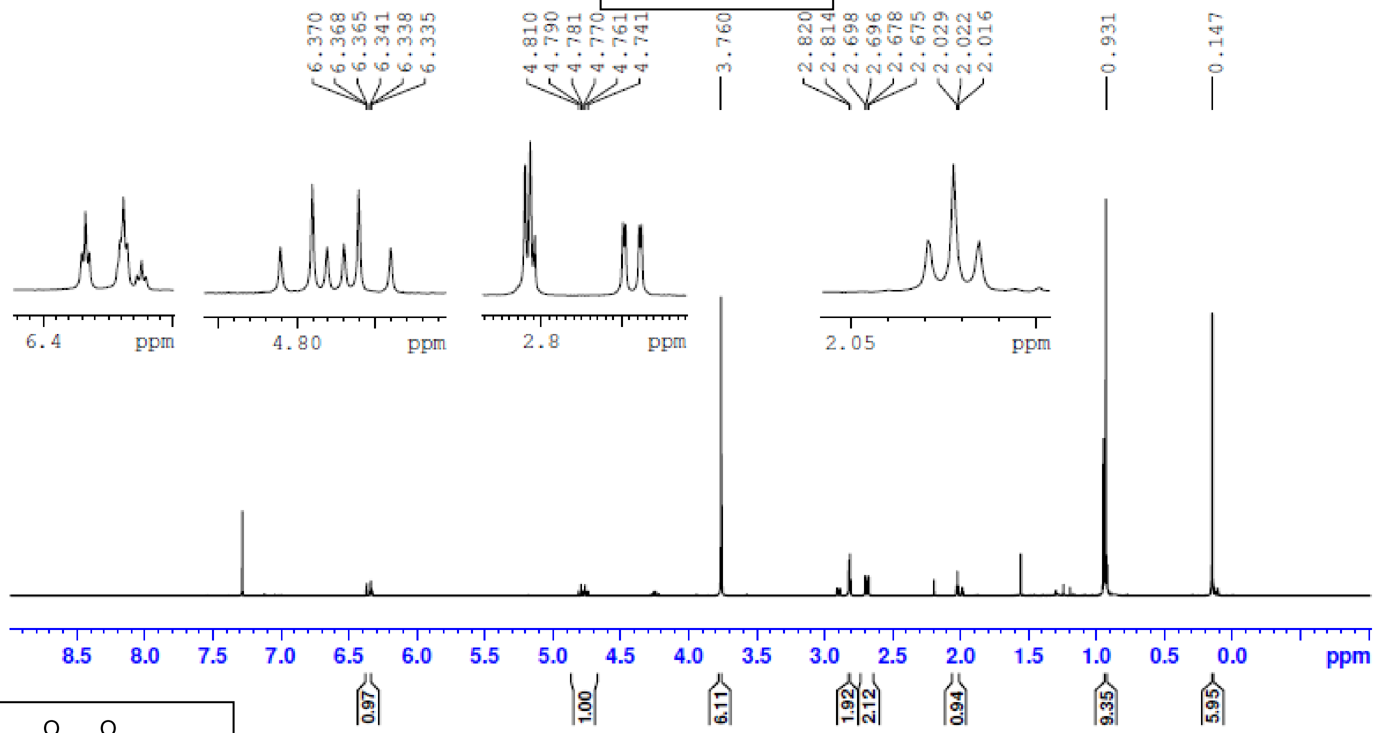

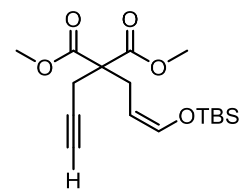

***cis*-S32**

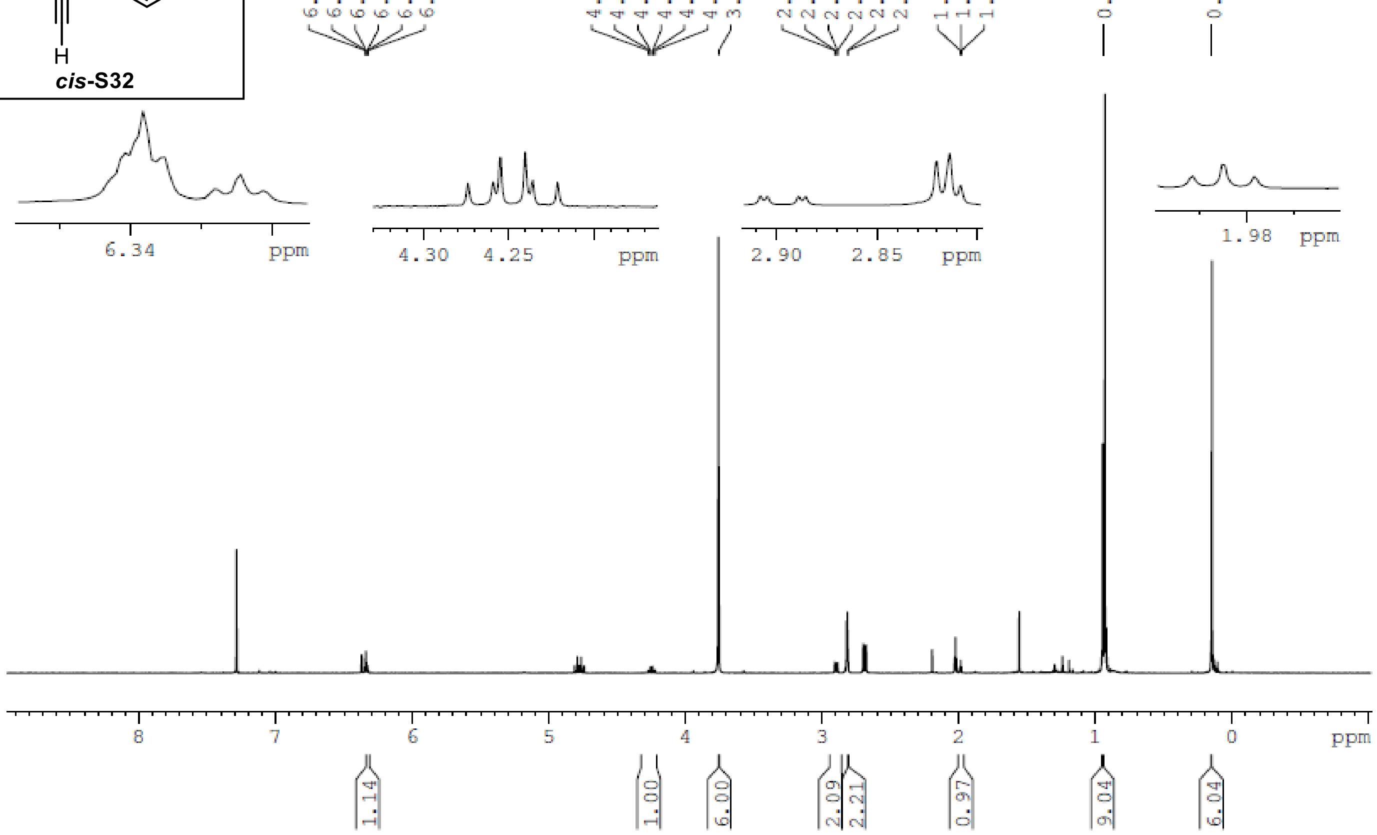

Compound **S32**.
100.6 MHz ^{13}C NMR spectrum
$CDCl_3$

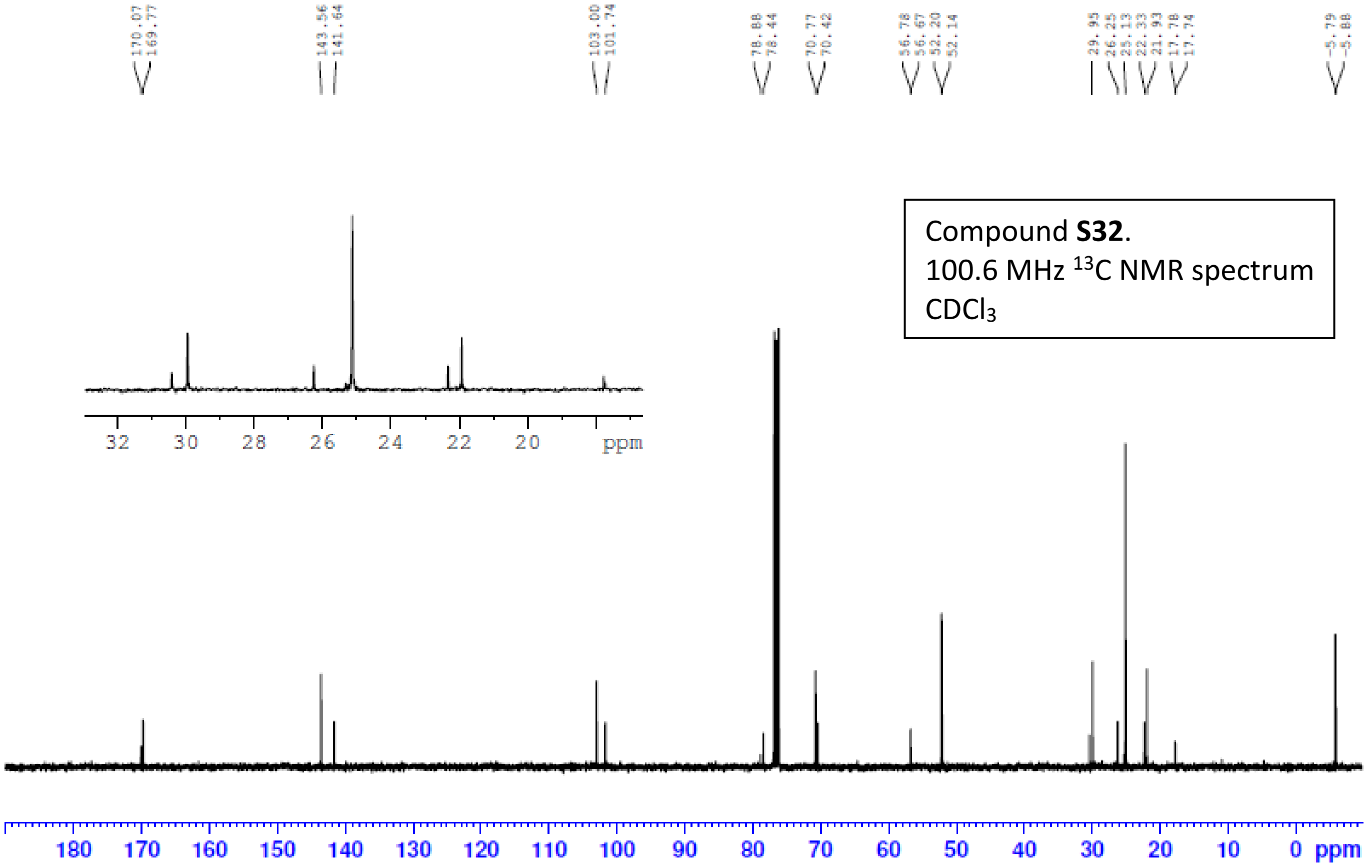

Compound **S33**.
400.13 MHz ^{1}H NMR spectrum
$CDCl_3$

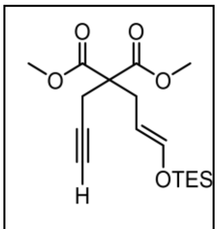

trans-**S33**

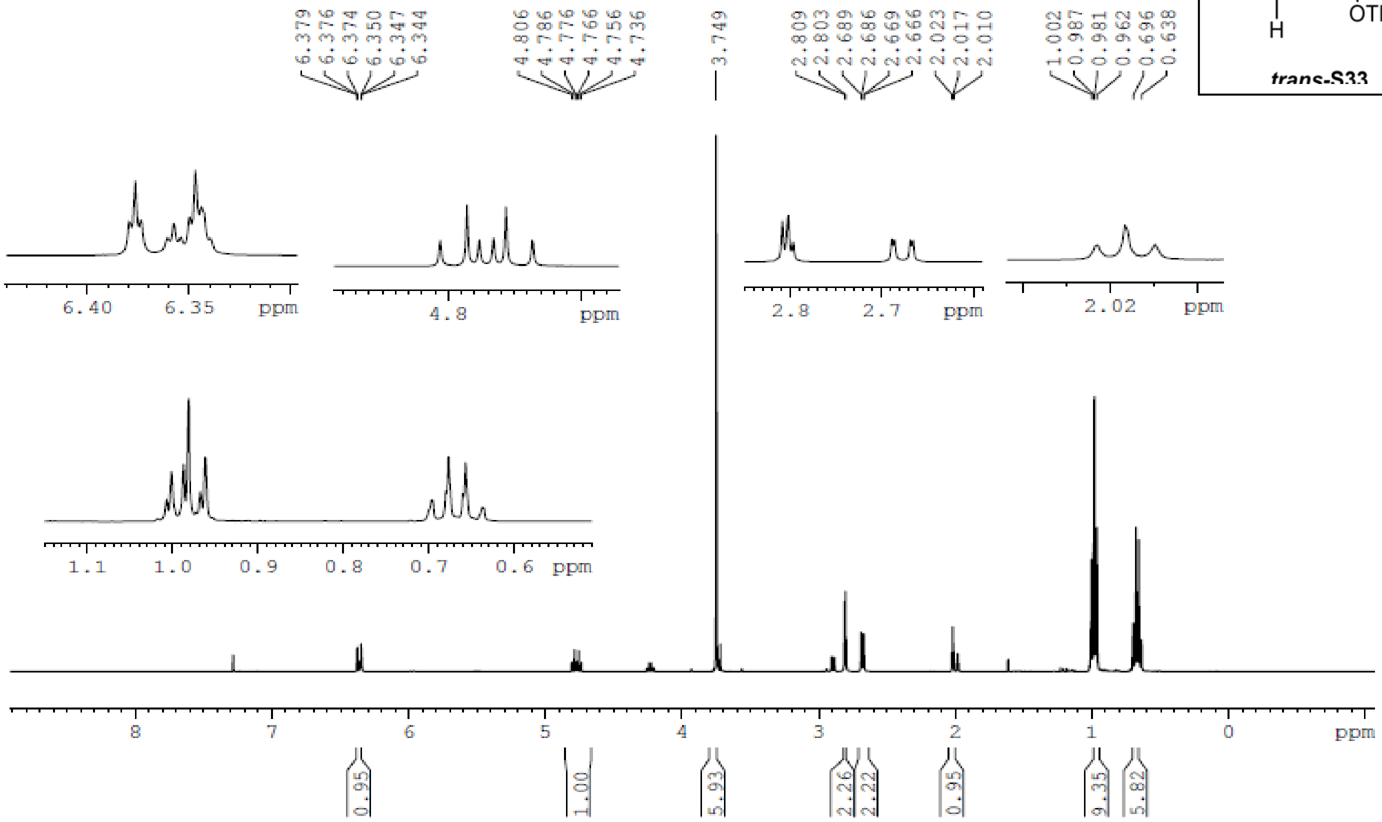

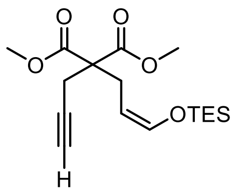

cis-**S33**

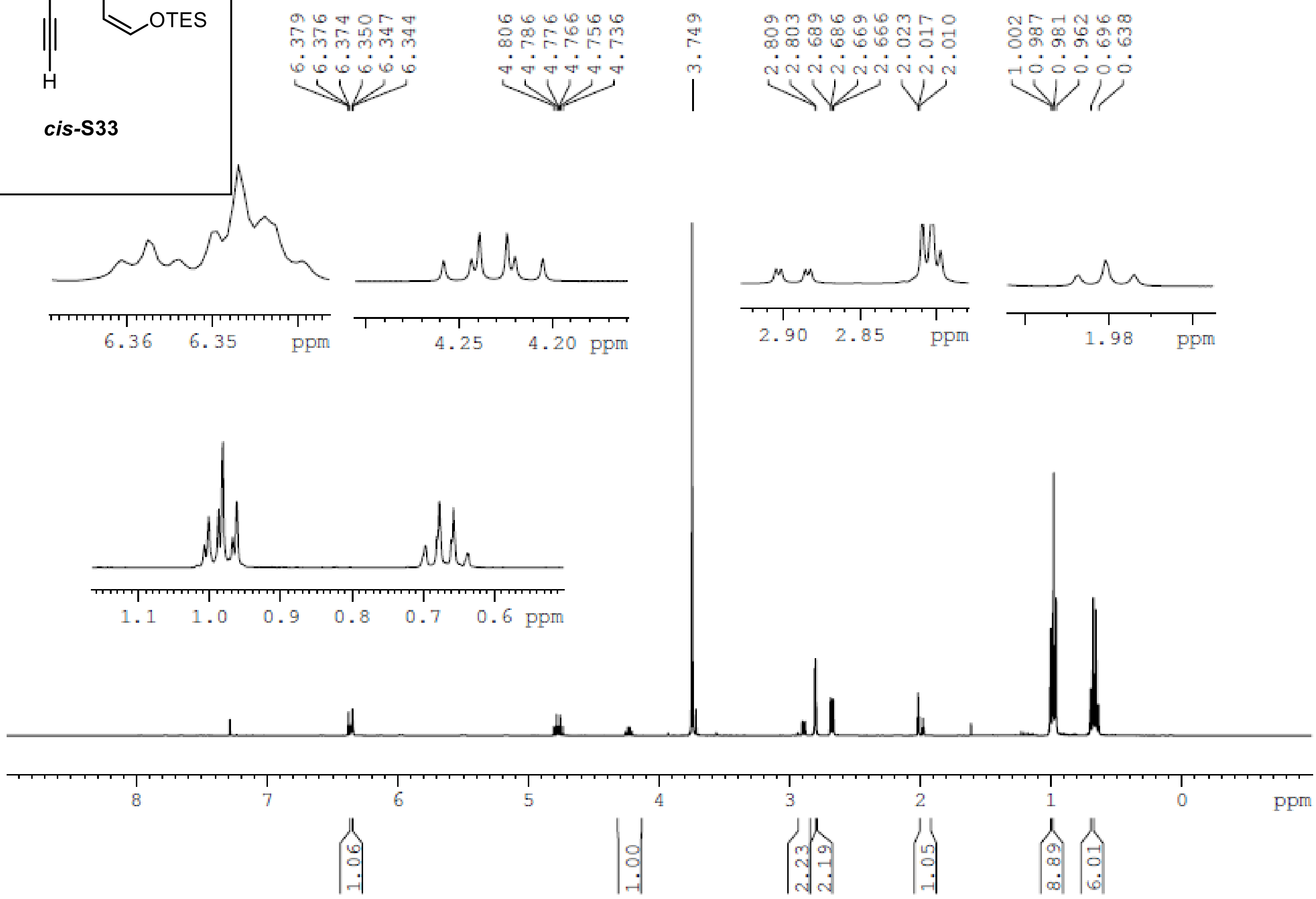

Compound **S33**.
100.6 MHz ^{13}C NMR spectrum
$CDCl_3$

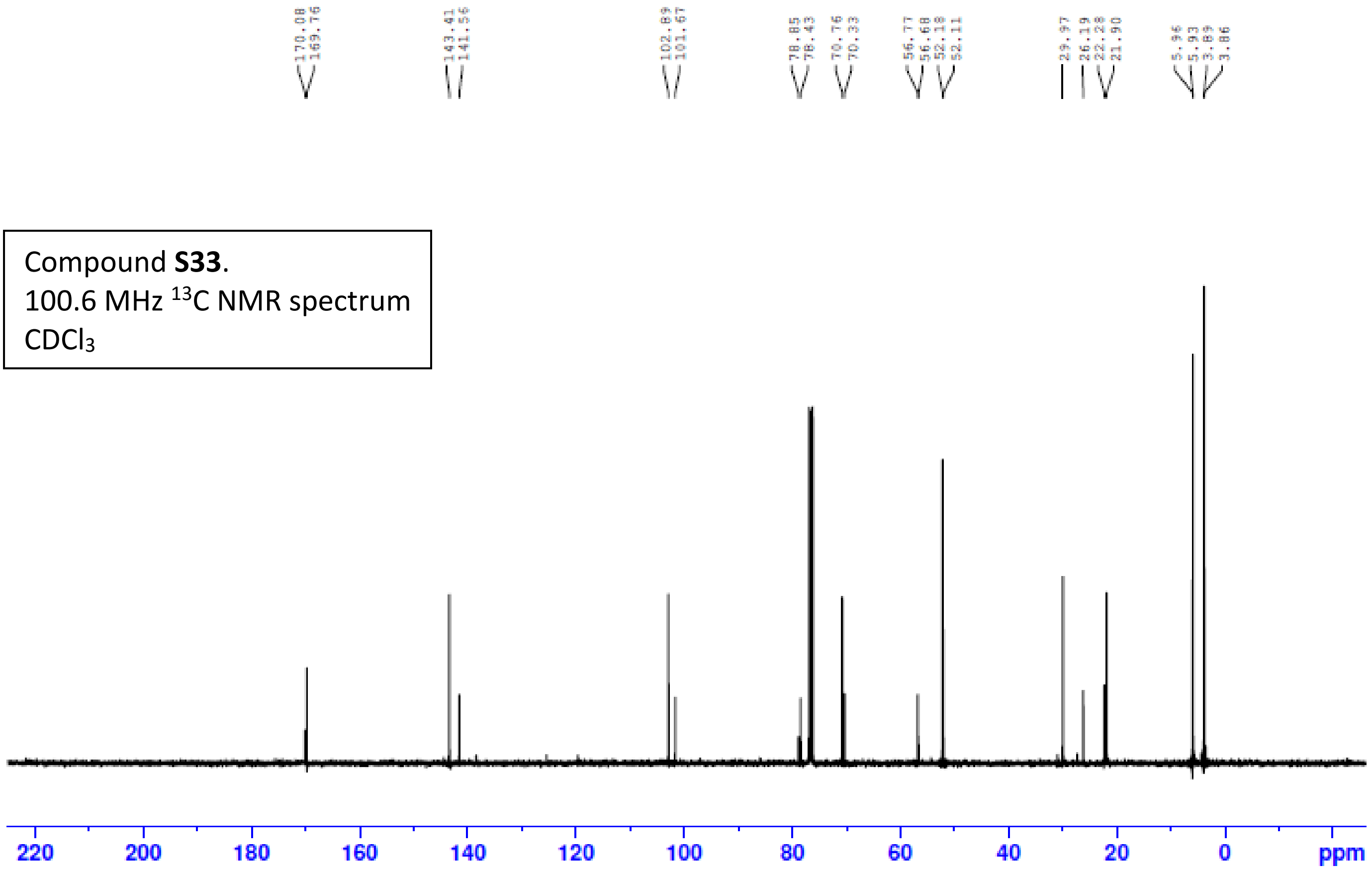

Compound **S34**.
400.13 MHz ^{1}H NMR spectrum
$CDCl_3$

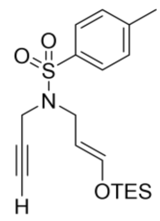

***trans*-S34**

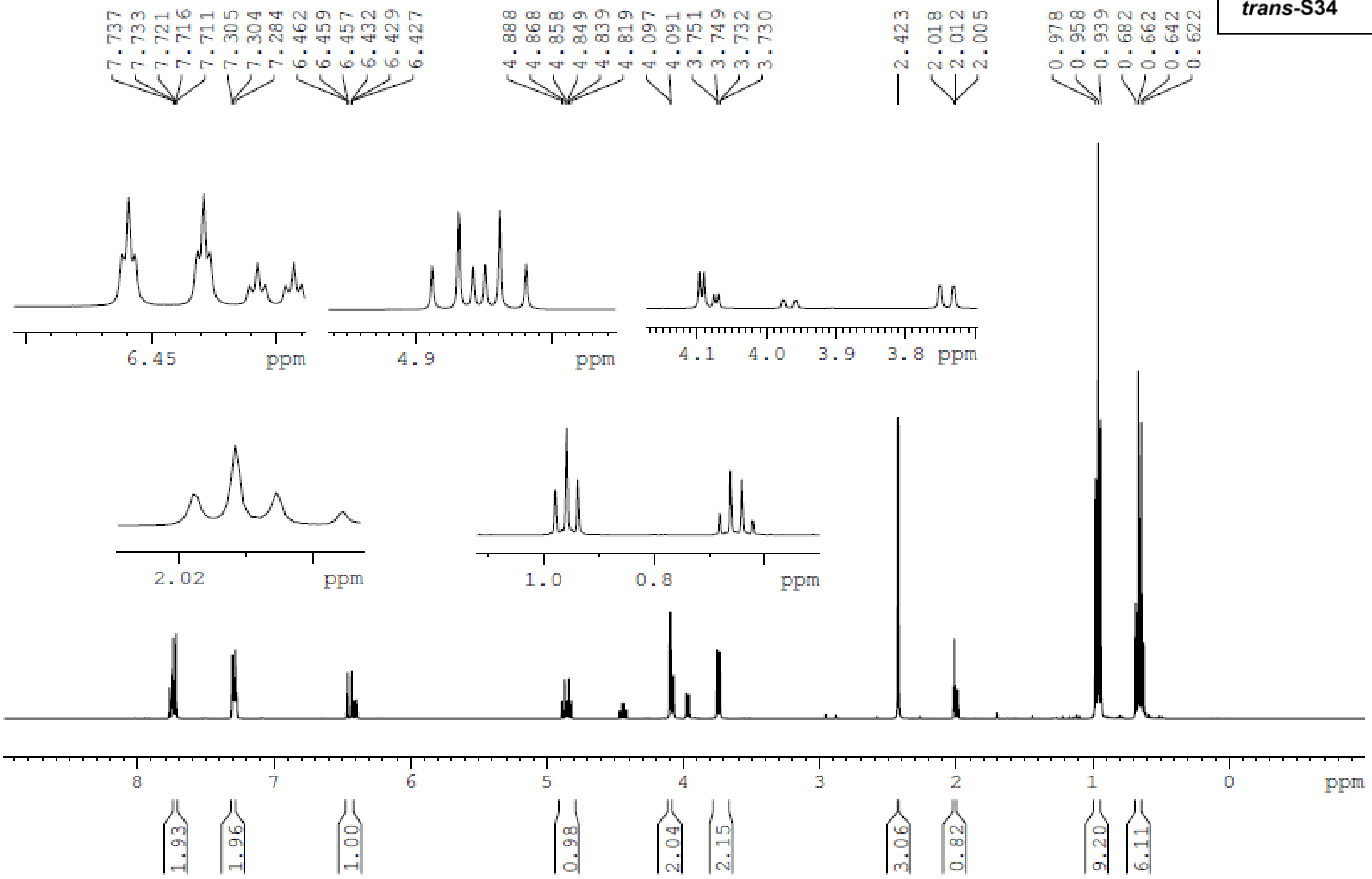

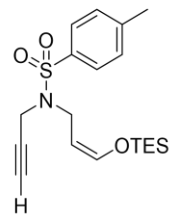

***cis*-S34**

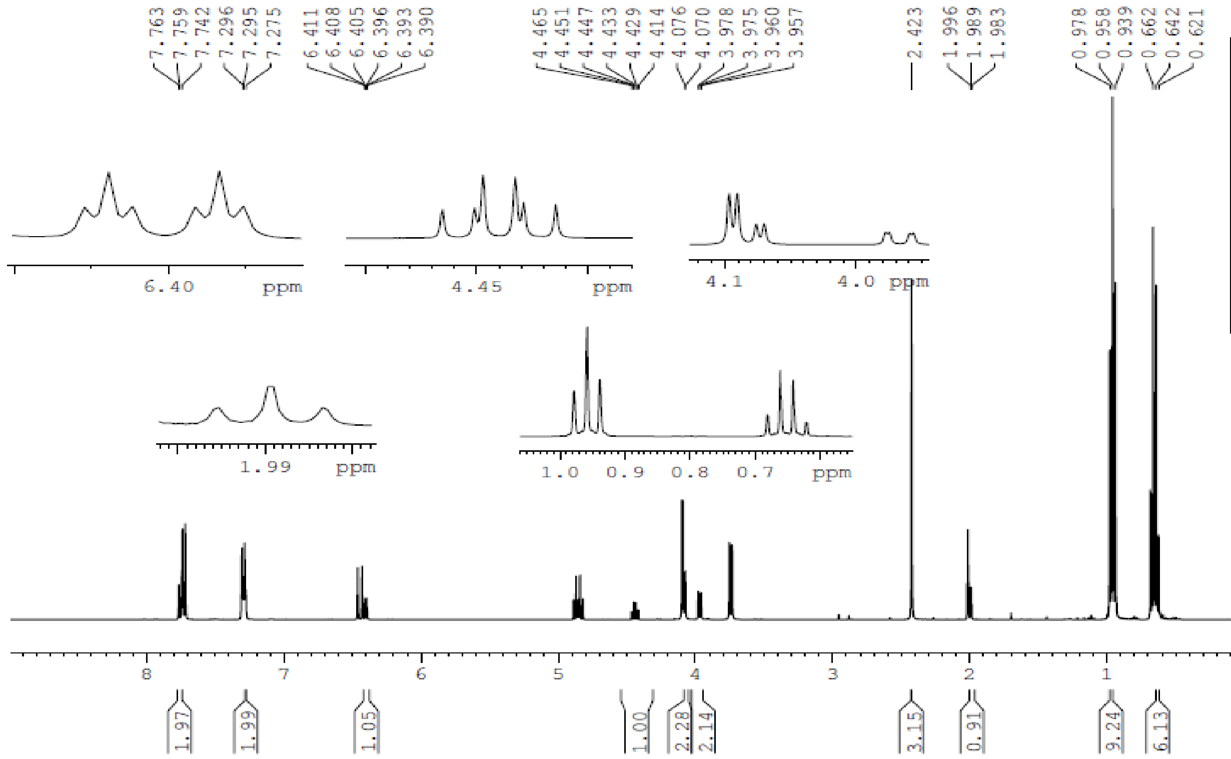

Compound **S34**.
100.6 MHz ^{13}C NMR spectrum
$CDCl_3$

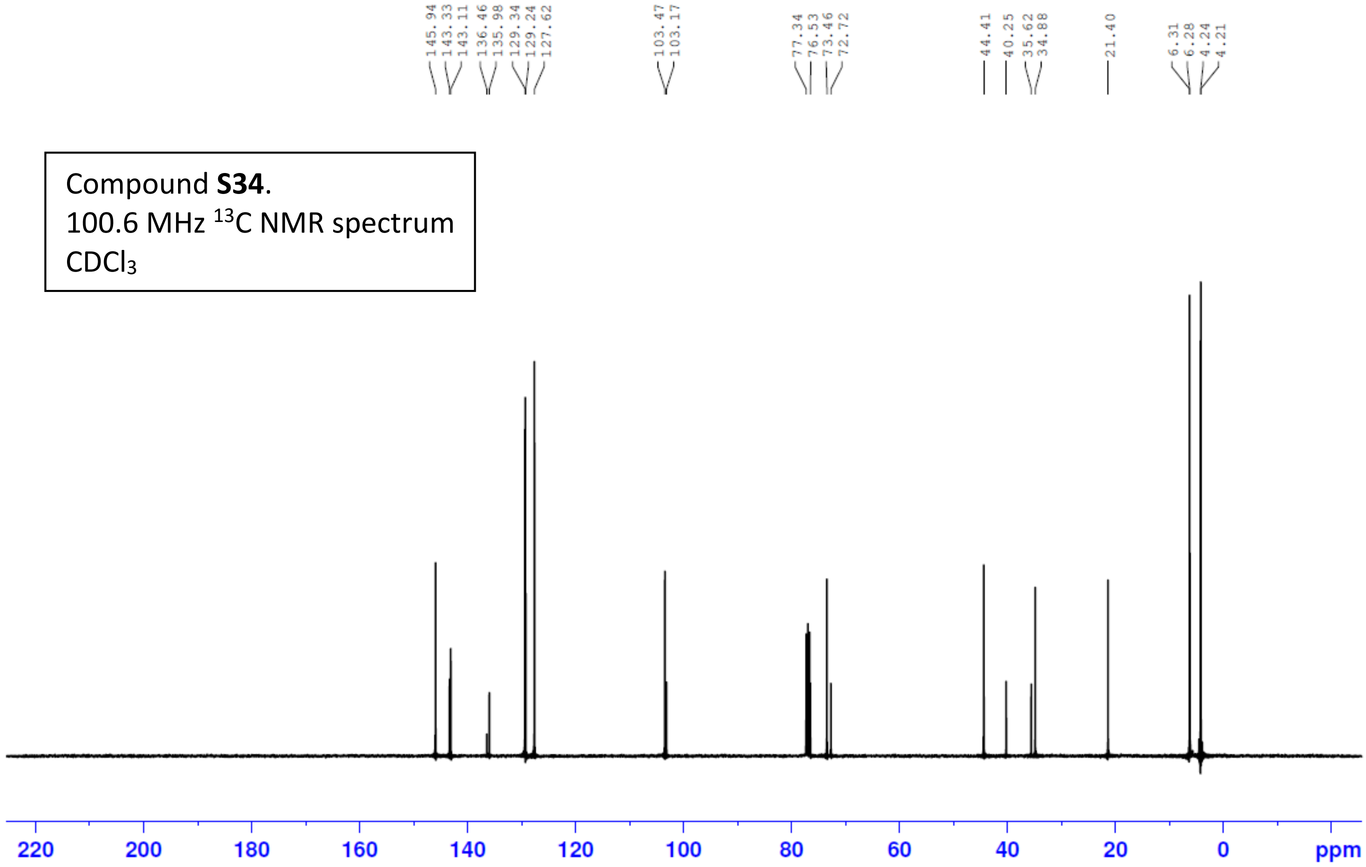

Compound **S35**.
400.13 MHz ^{1}H NMR spectrum
$CDCl_3$

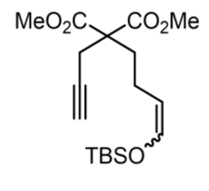

S35

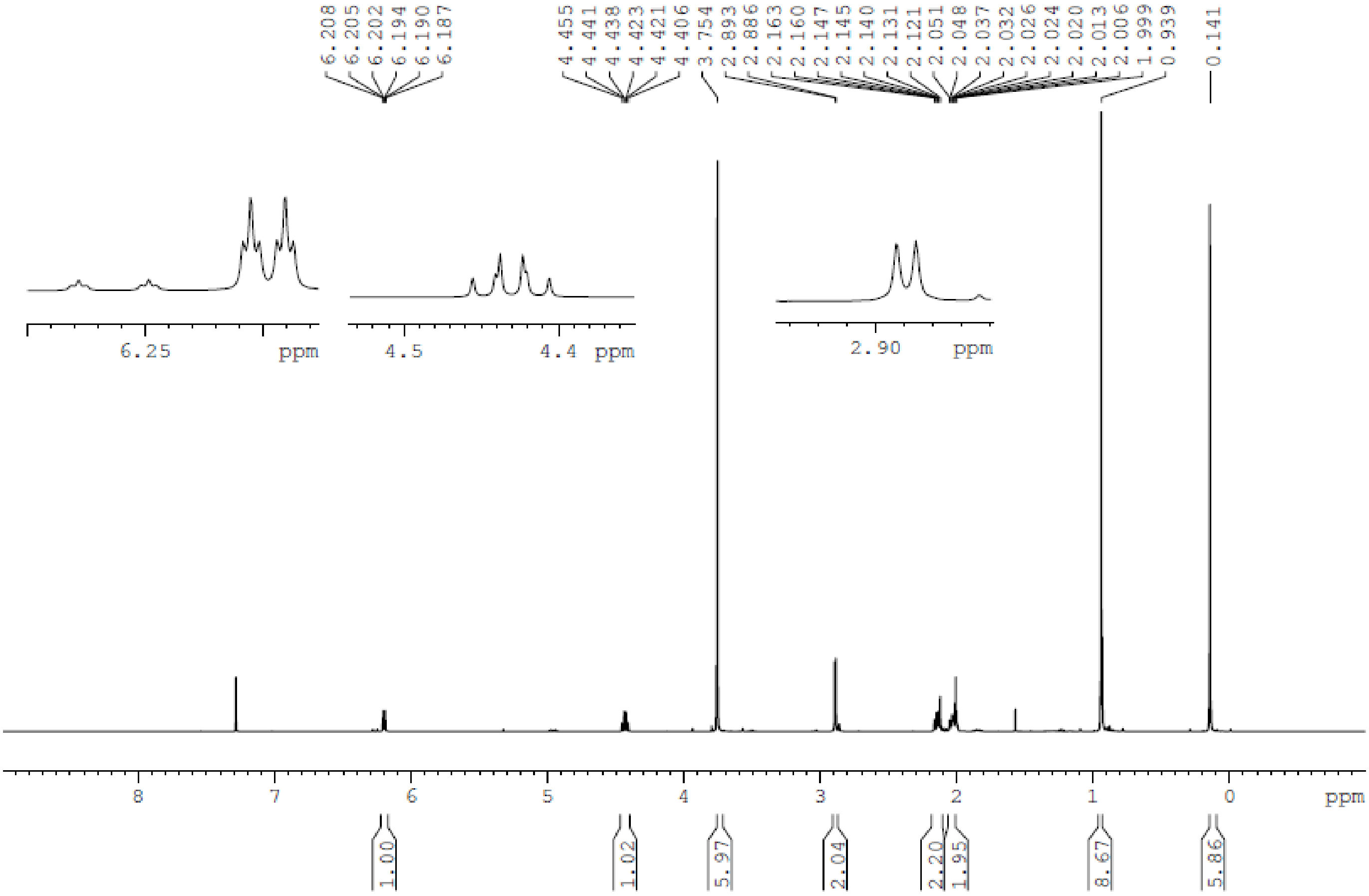

Compound **S35**.
100.6 MHz ^{13}C NMR spectrum
$CDCl_3$

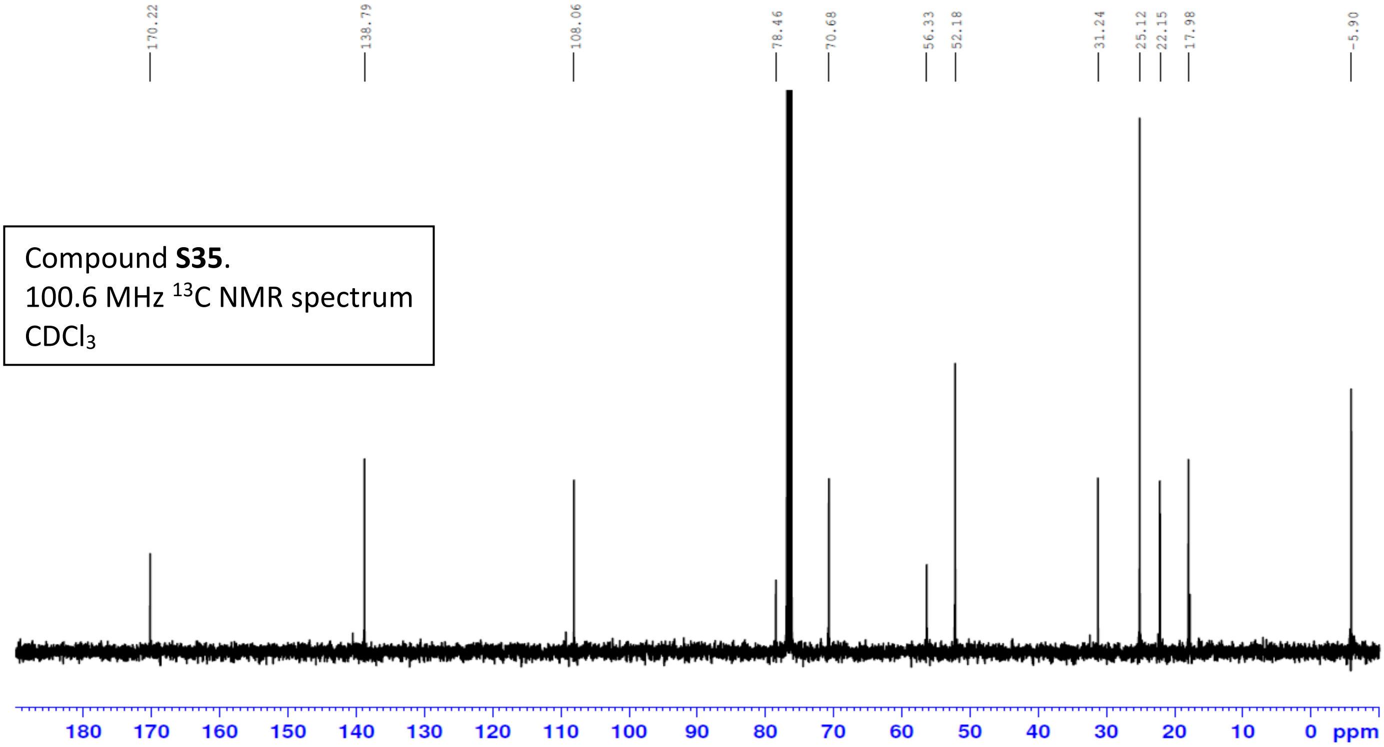

Compound **3**.
400.13 MHz ^{1}H NMR spectrum
$CDCl_3$

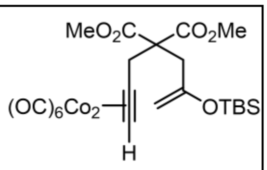

3

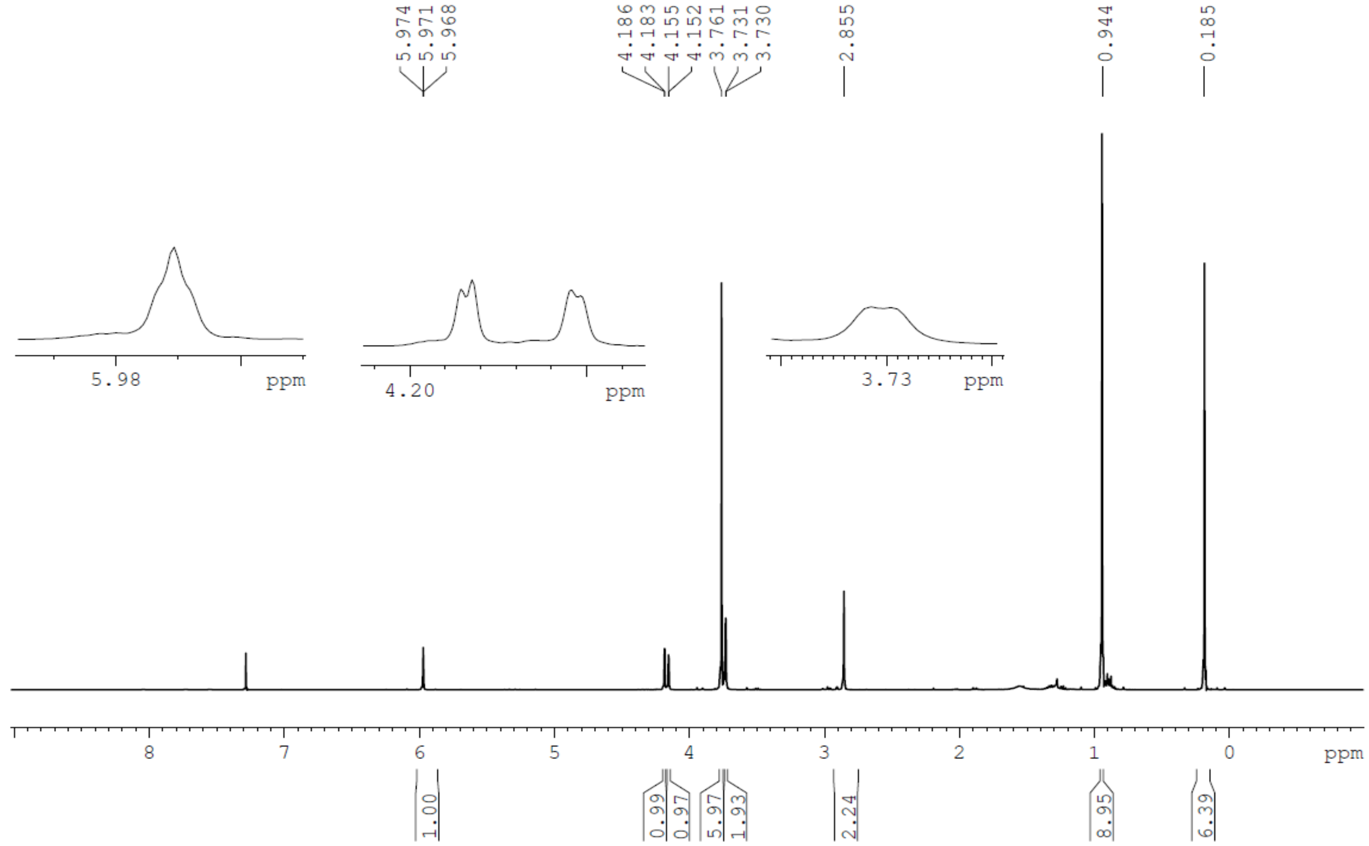

Compound **3**.
100.6 MHz ^{13}C NMR spectrum
$CDCl_3$

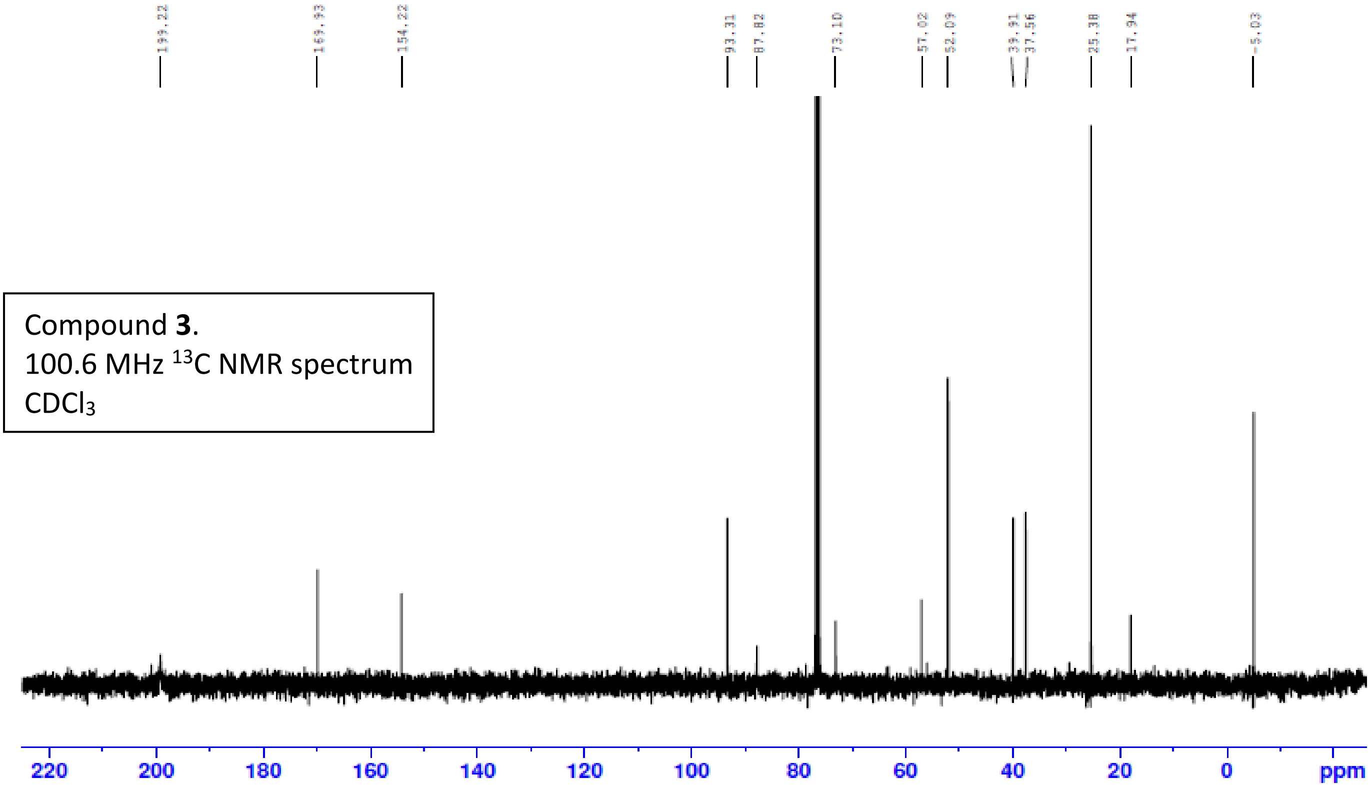

Compound **6a**.
400.13 MHz ^{1}H NMR spectrum
$CDCl_3$

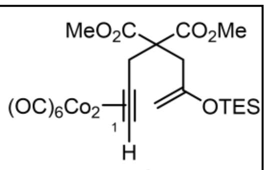

6a

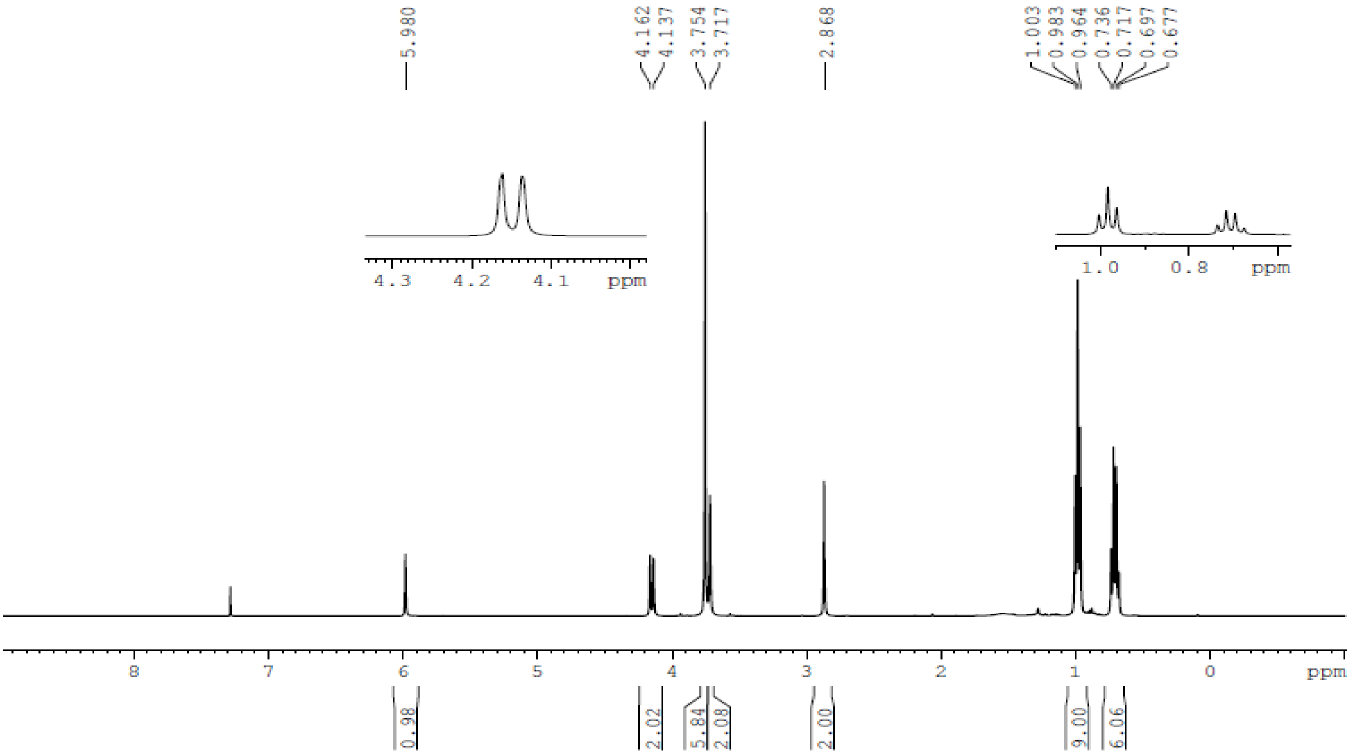

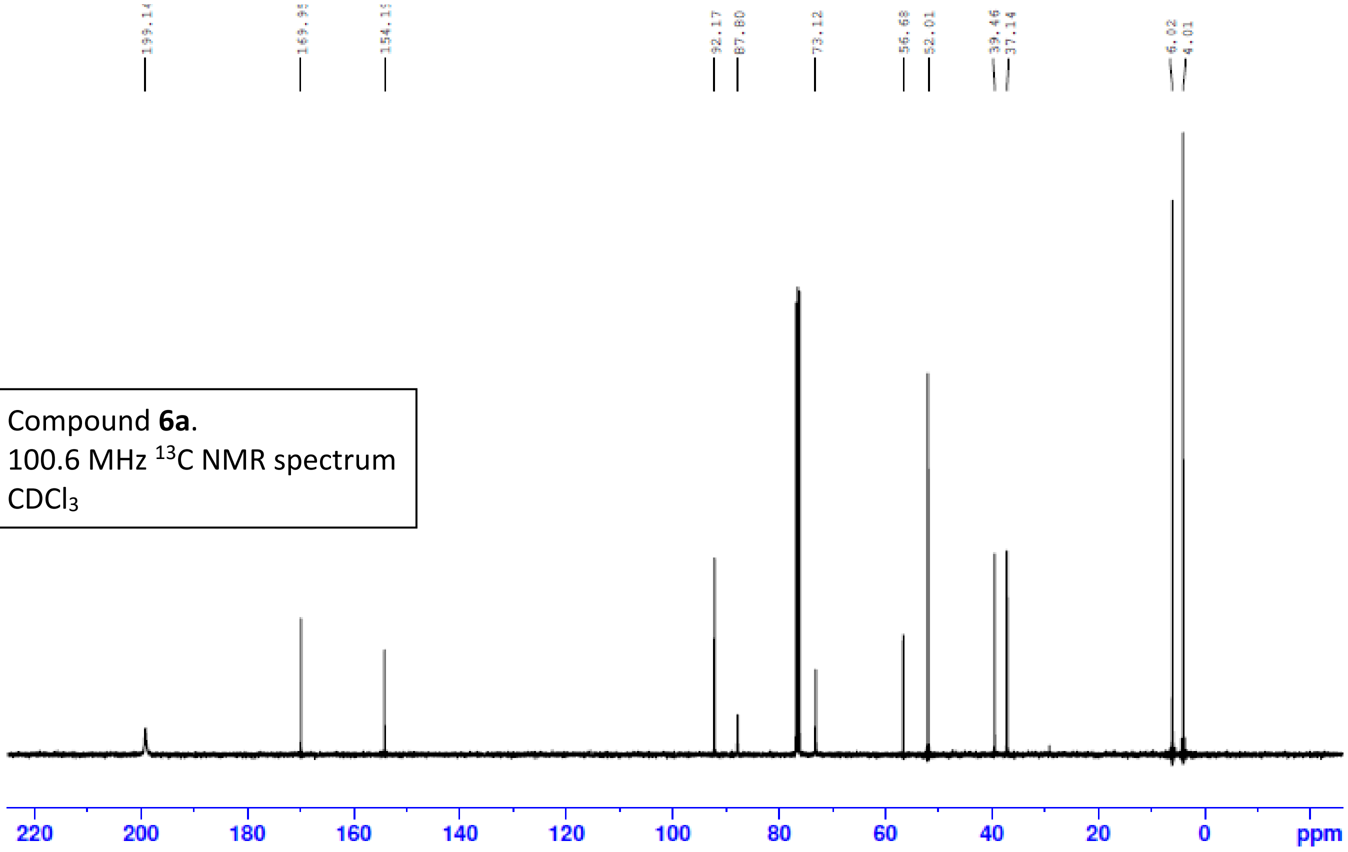

Compound **6a**.
100.6 MHz ^{13}C NMR spectrum
$CDCl_3$

Compound **6b**.
400.13 MHz ^{1}H NMR spectrum
$CDCl_3$

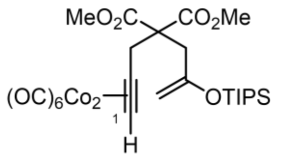

6b

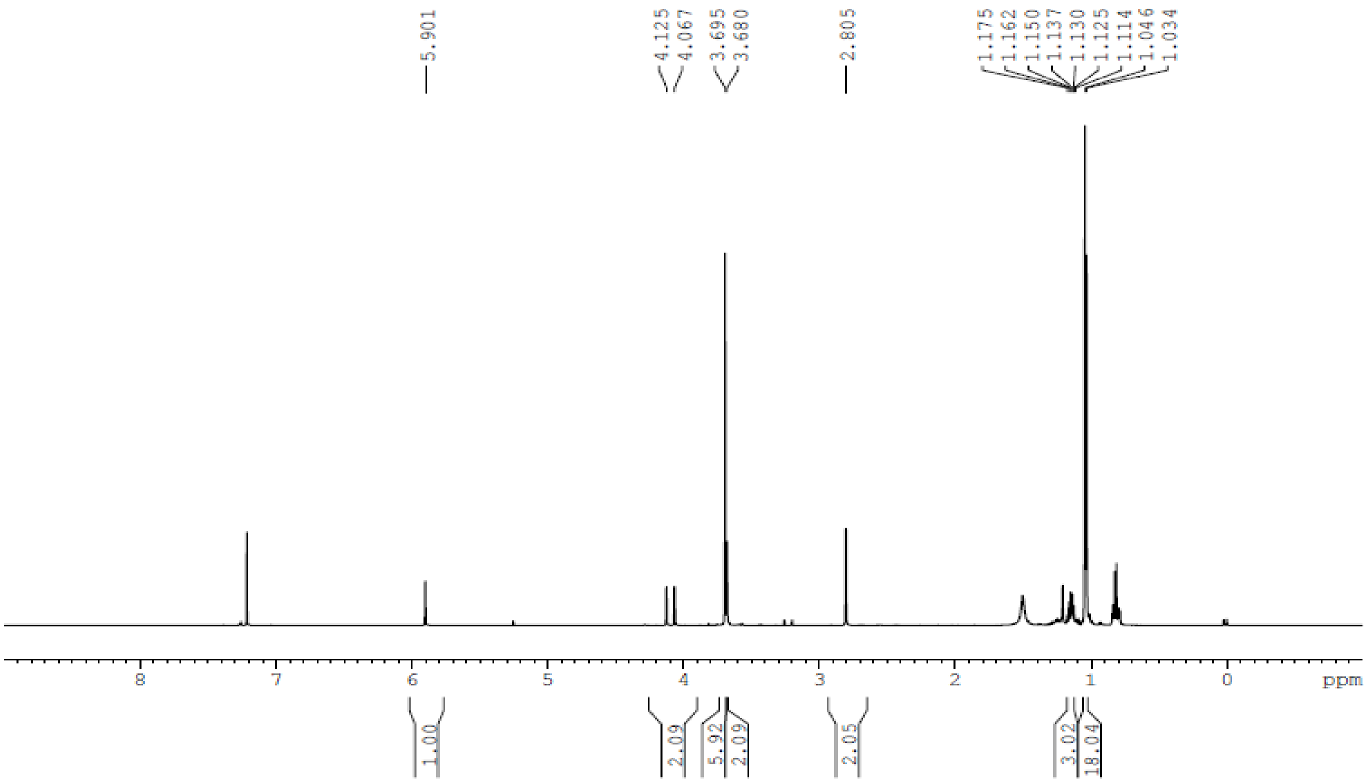

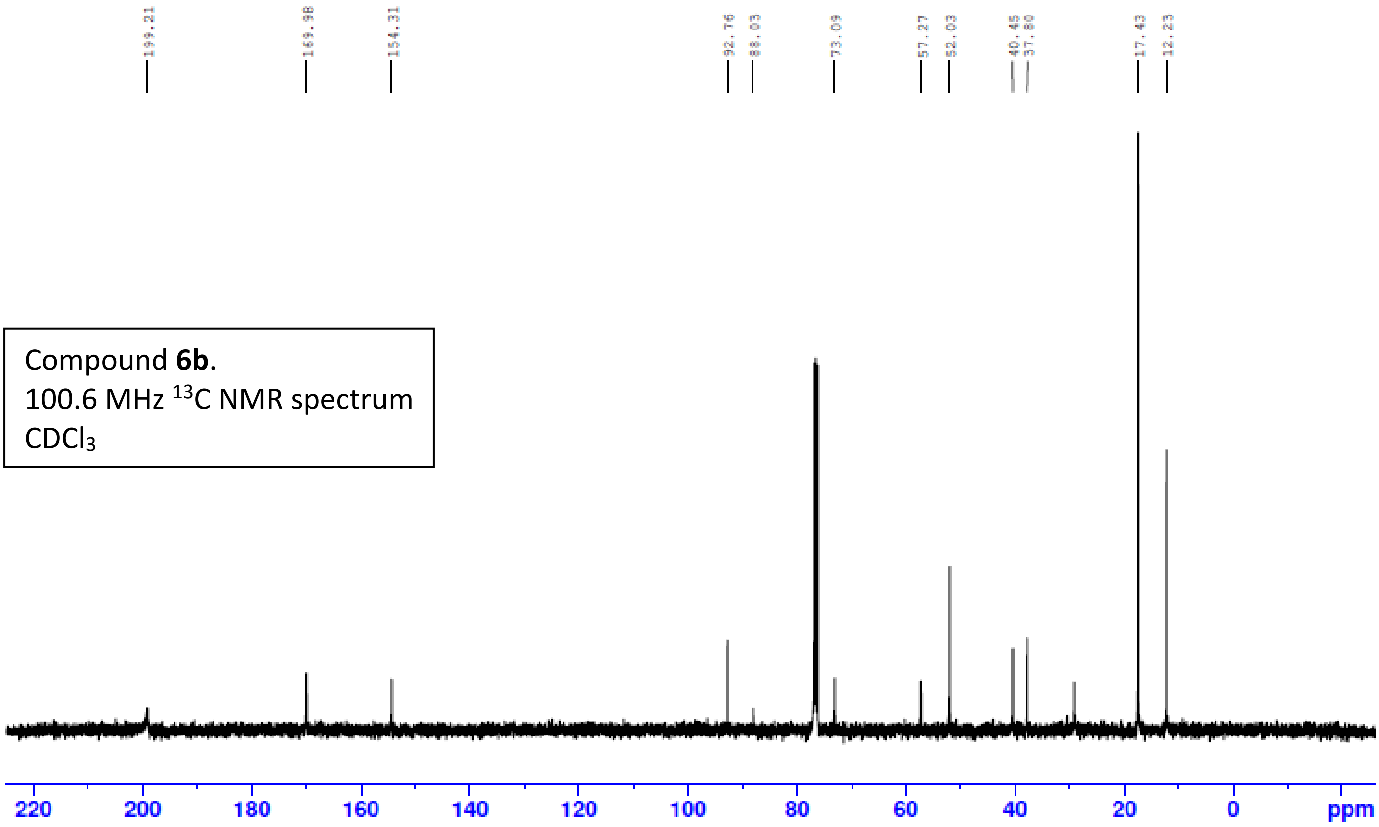

Compound **6b**.
100.6 MHz ^{13}C NMR spectrum
$CDCl_3$

Compound **8a**.
400.13 MHz ^{1}H NMR spectrum
$CDCl_3$

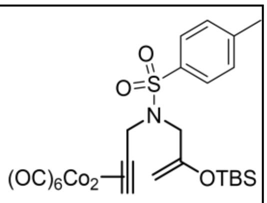

8a

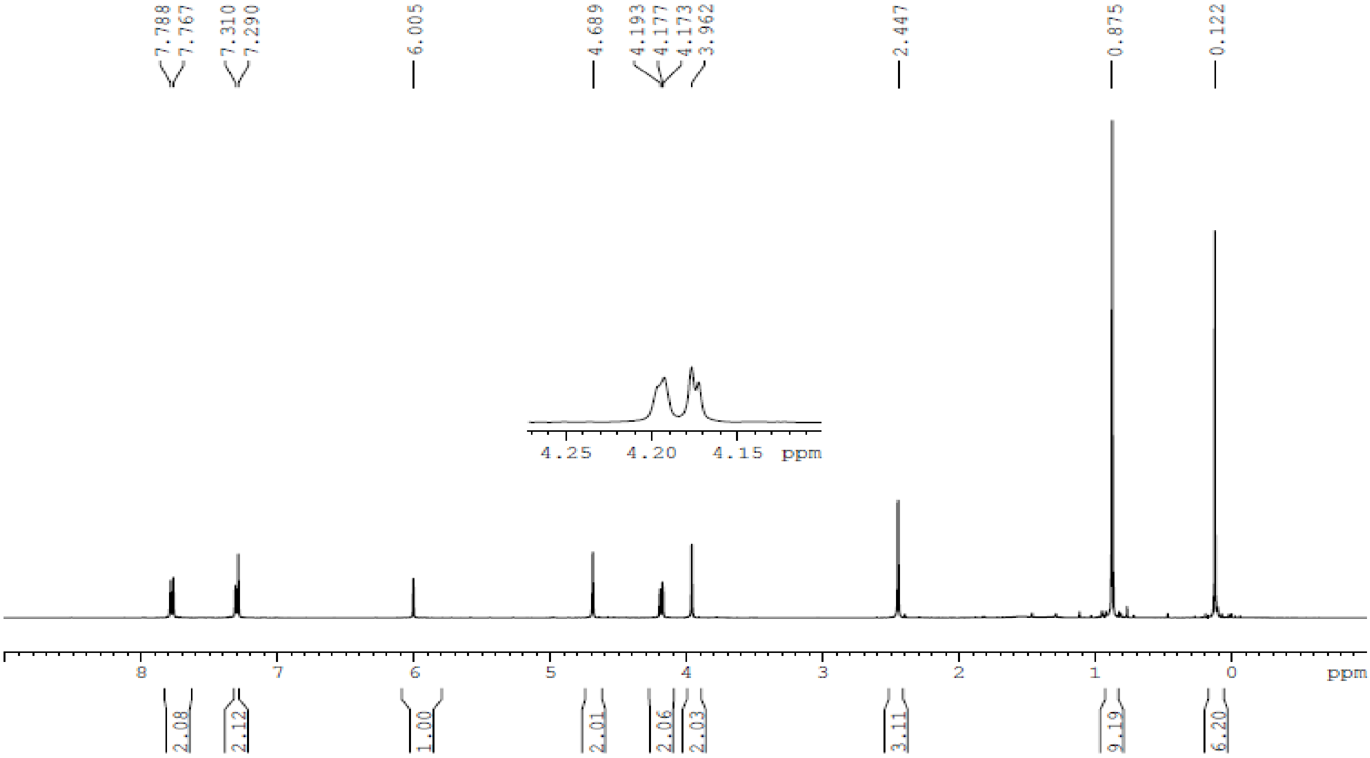

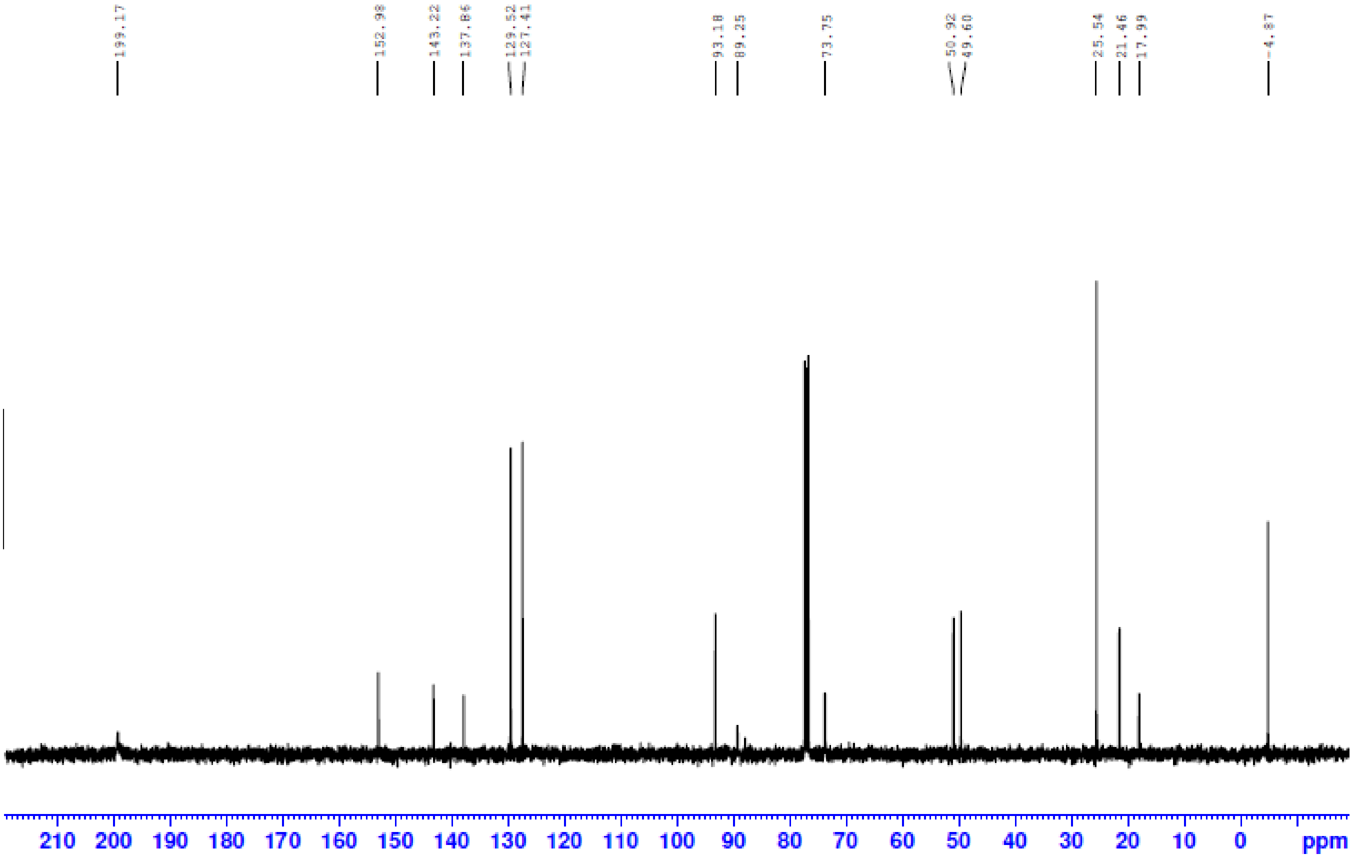

Compound **8b**.
400.13 MHz ^{1}H NMR spectrum
$CDCl_3$

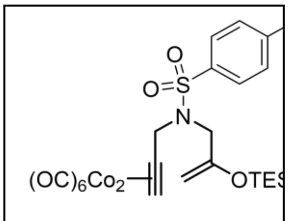

8b

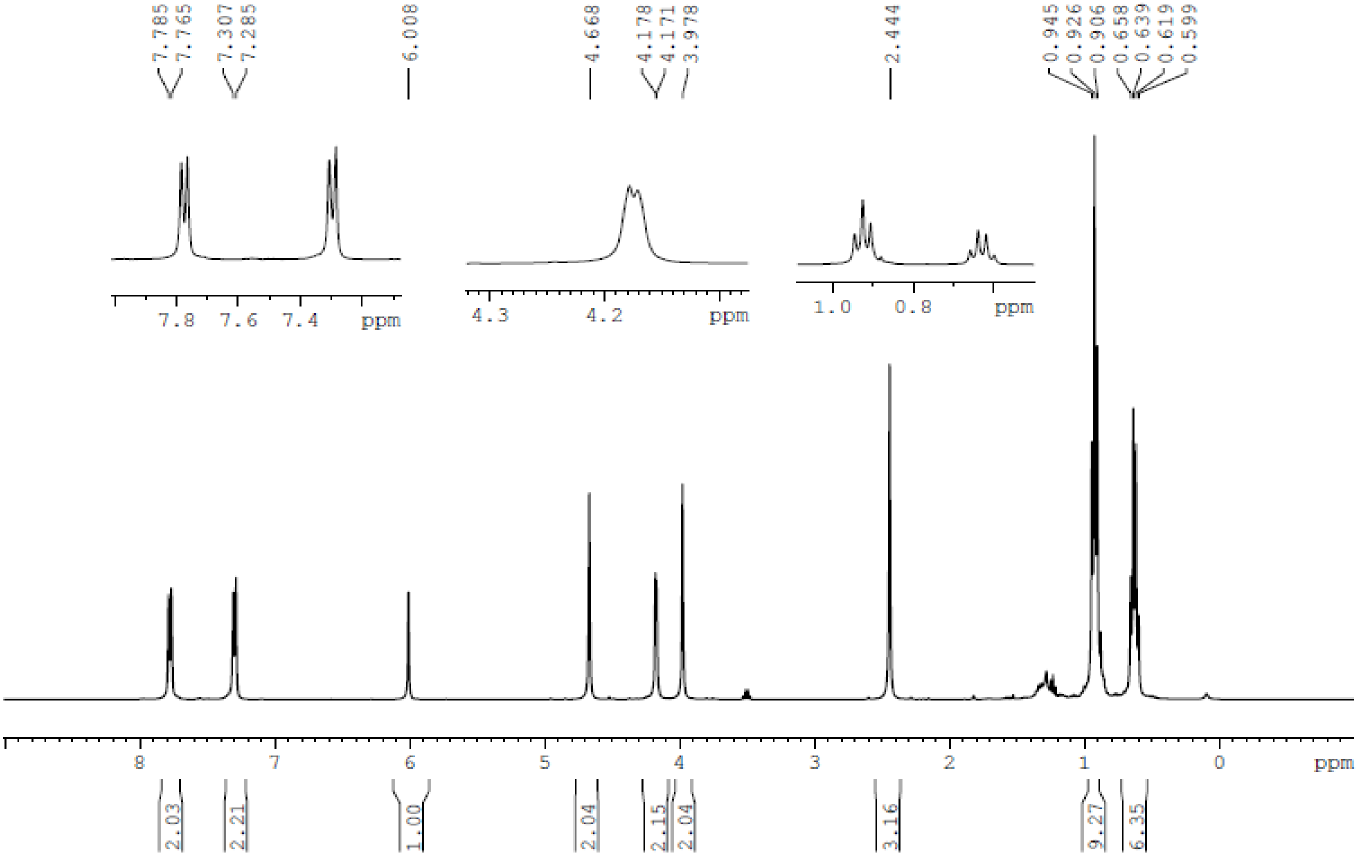

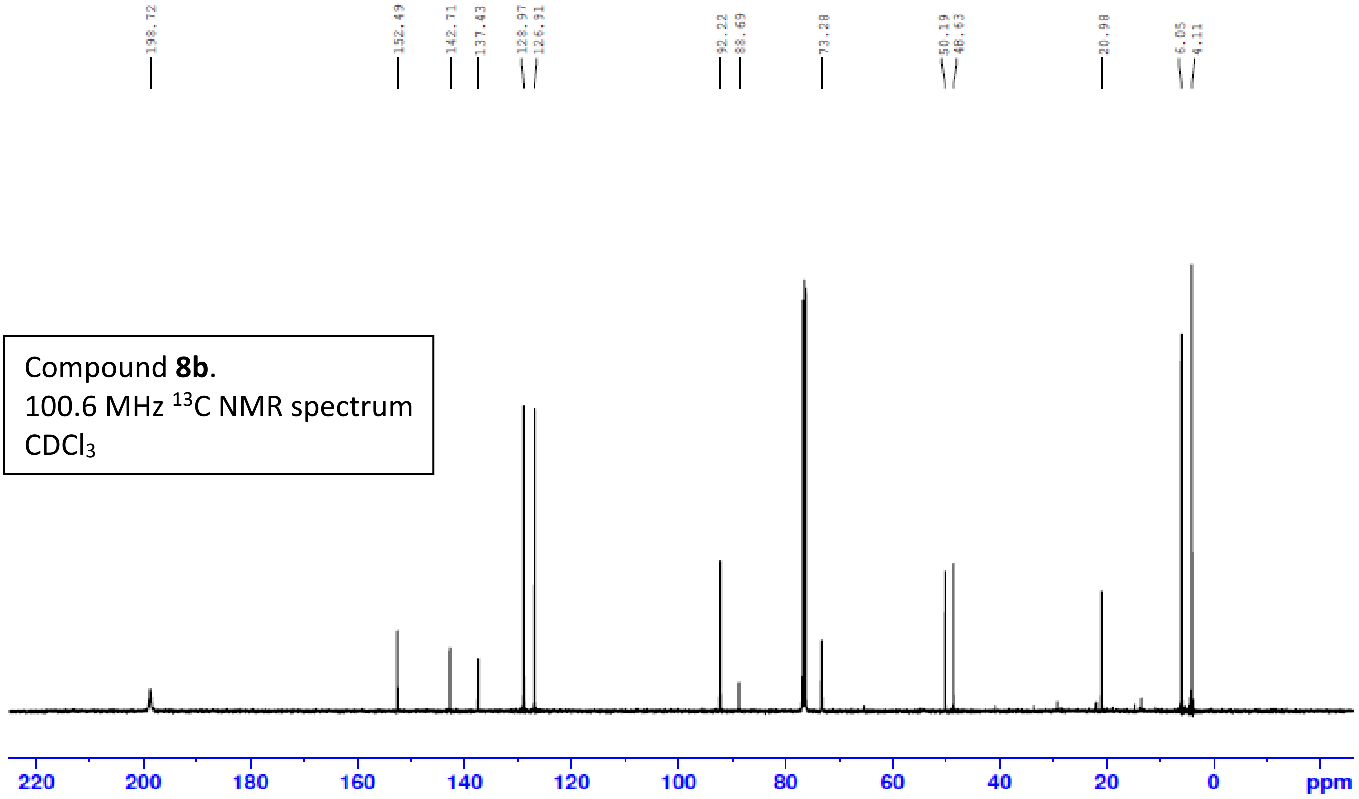

Compound **8b**.
100.6 MHz ^{13}C NMR spectrum
$CDCl_3$

Compound **8c**.
400.13 MHz ^{1}H NMR spectrum
$CDCl_3$

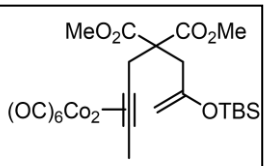

8c

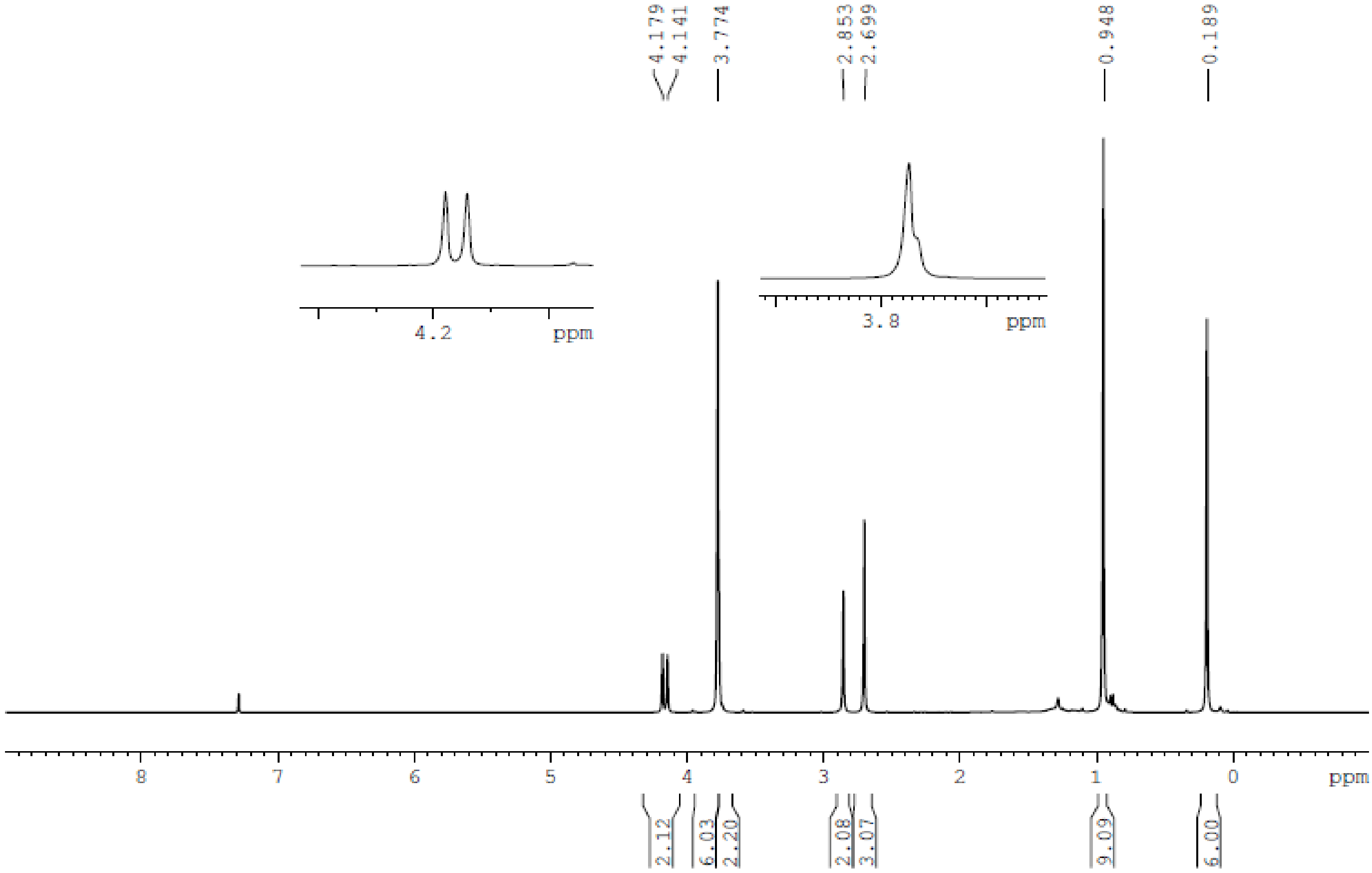

Compound **8c**.
100.6 MHz ^{13}C NMR spectrum
$CDCl_3$

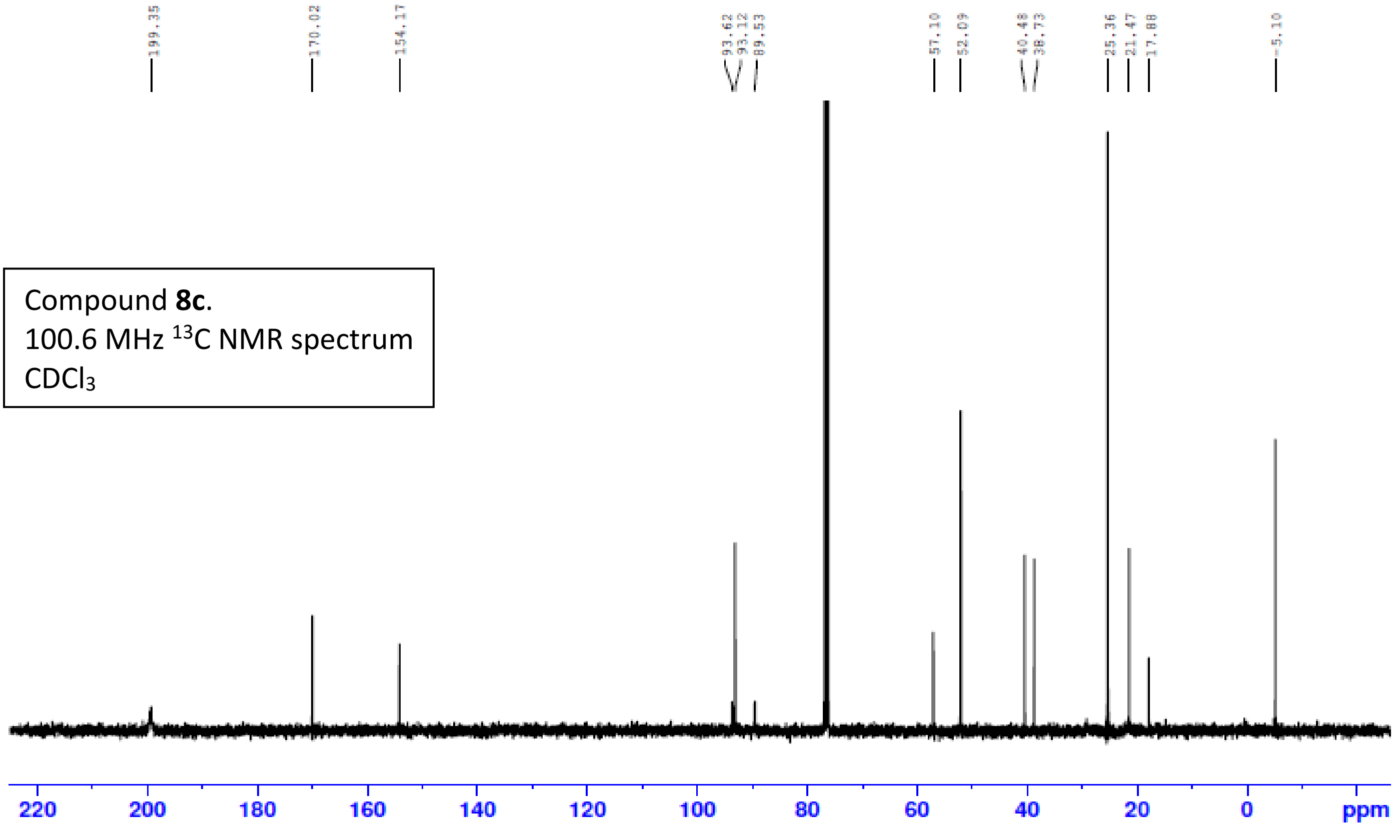

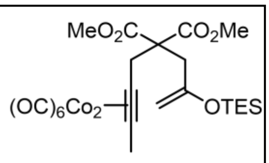

8d

Compound **8d**.
400.13 MHz ^{1}H NMR spectrum
$CDCl_3$

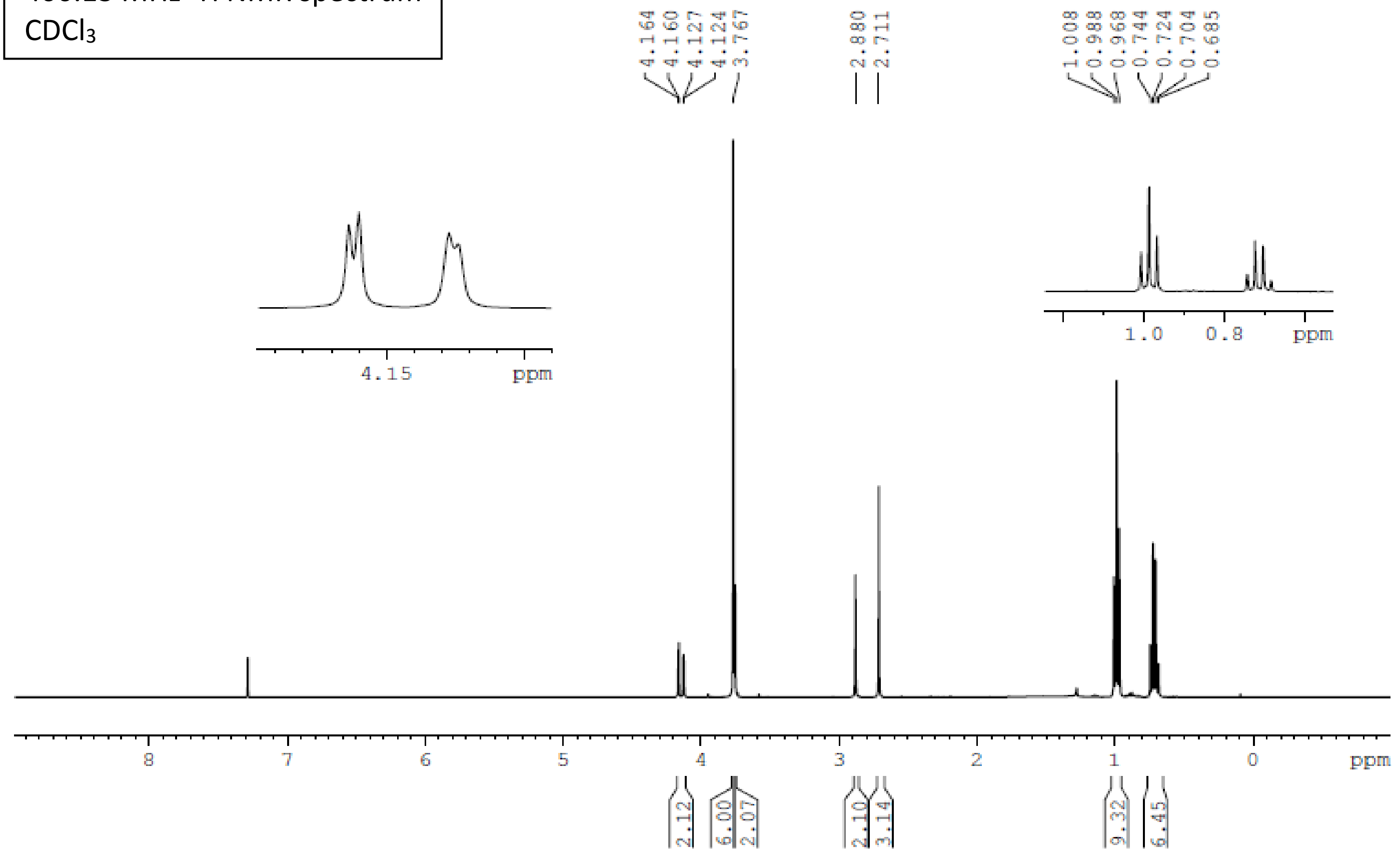

Compound **8d**.
100.6 MHz ^{13}C NMR spectrum
$CDCl_3$

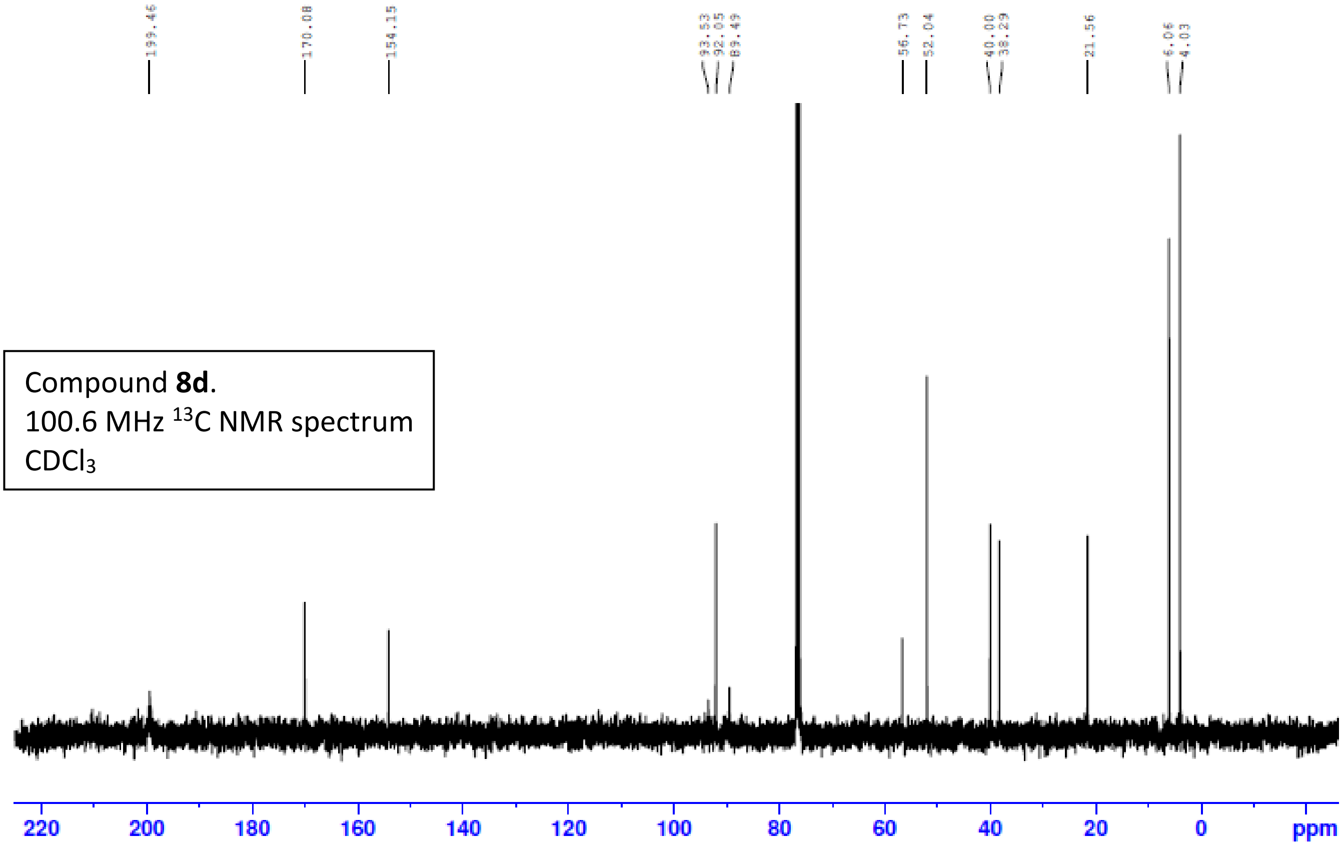

Compound **8e**.
400.13 MHz ^{1}H NMR spectrum
$CDCl_3$

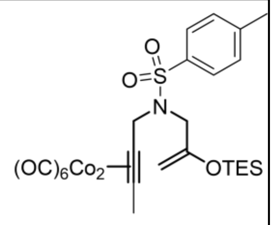

8e

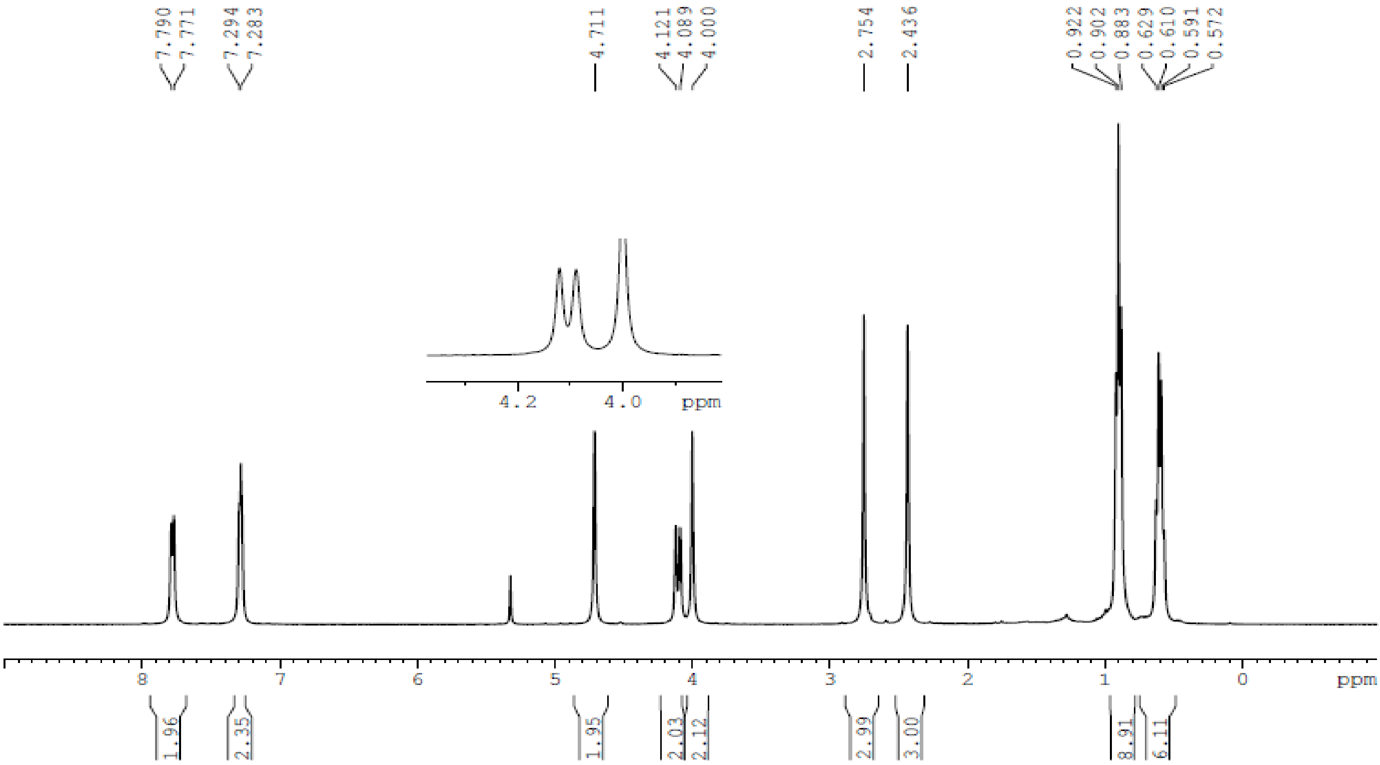

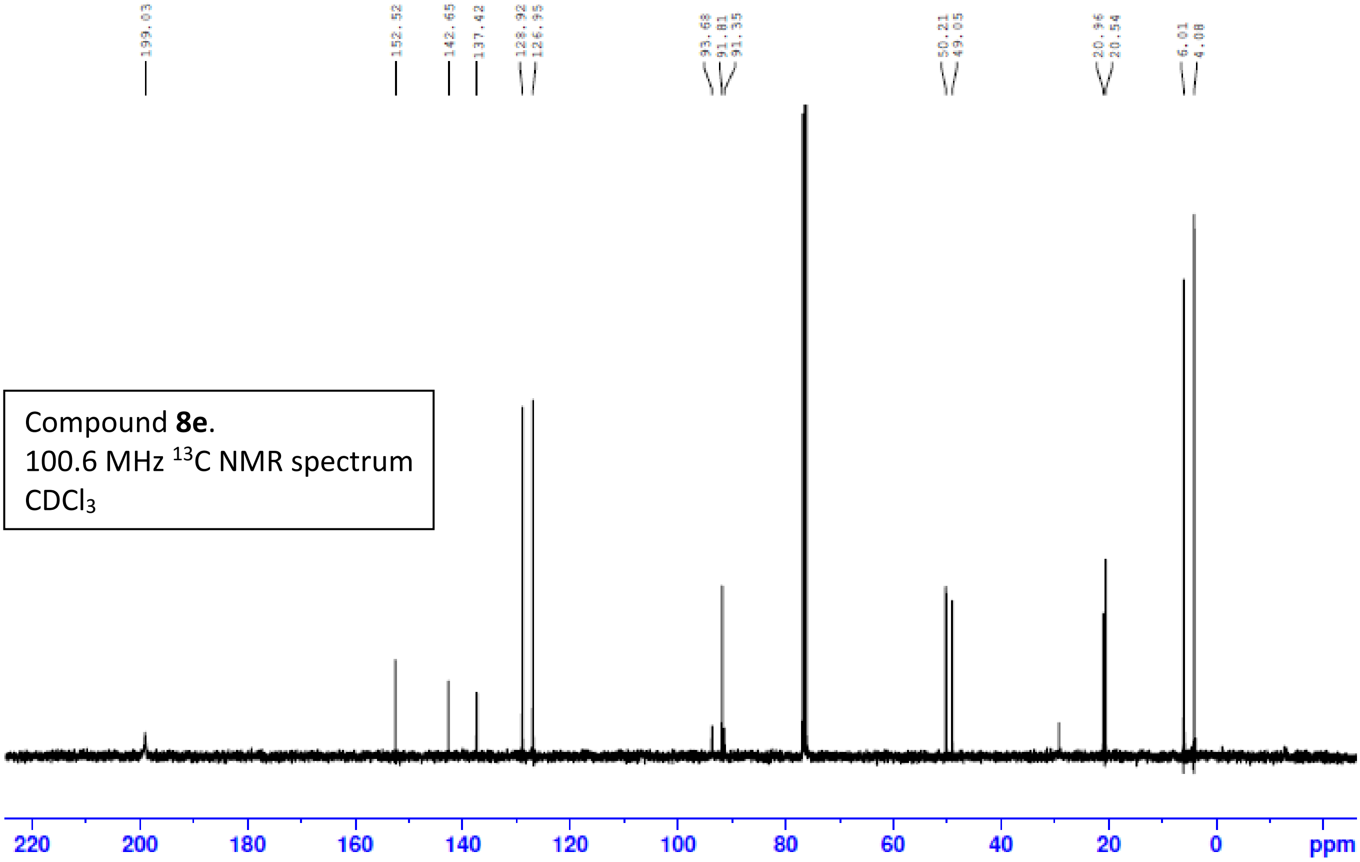

Compound **8e**.
100.6 MHz ^{13}C NMR spectrum
$CDCl_3$

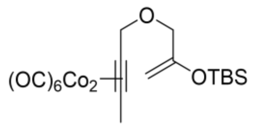

8f

Compound **8f**.
400.13 MHz ^{1}H NMR spectrum

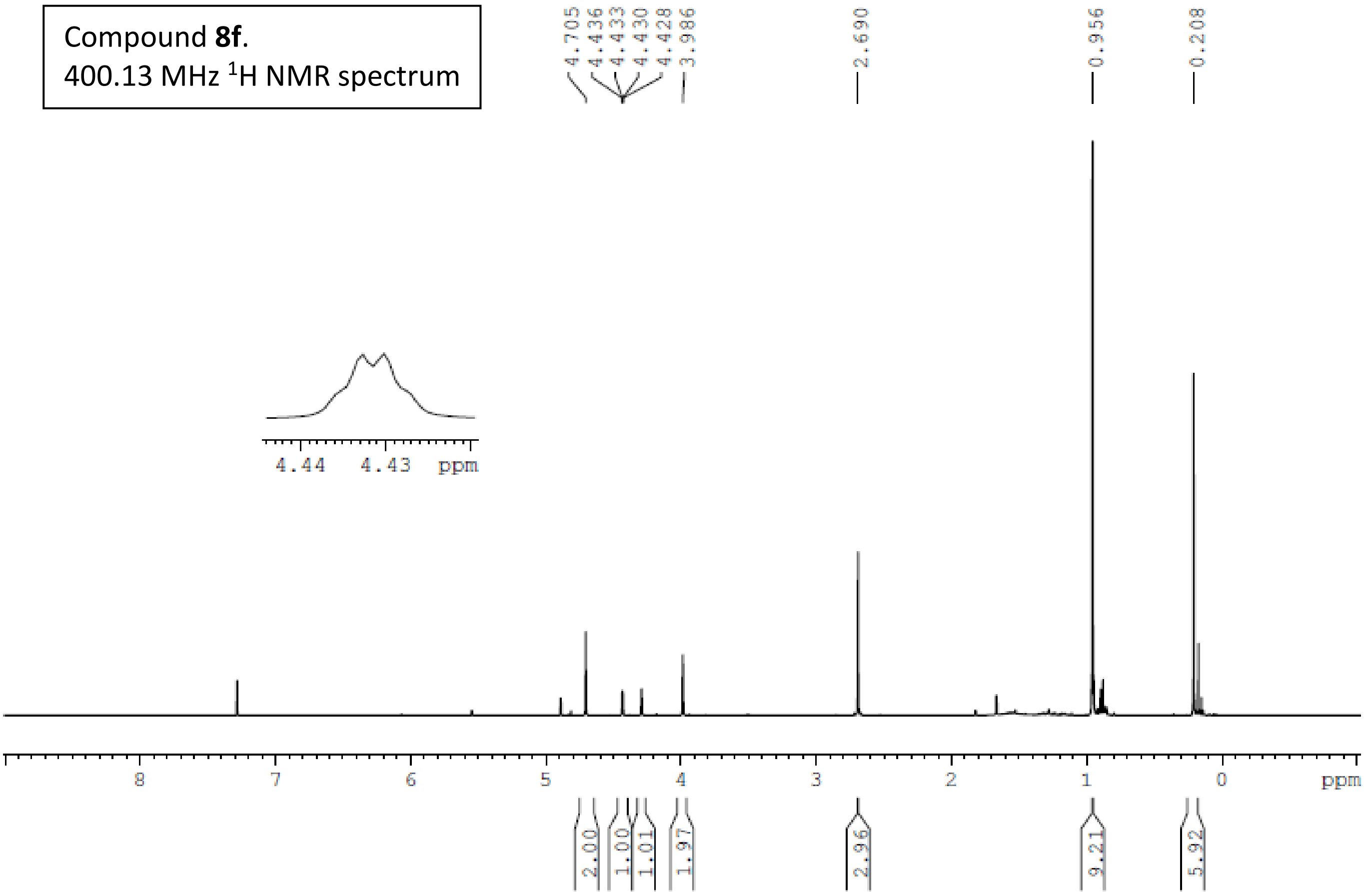

Compound **8f**.
100.6 MHz ^{13}C NMR spectrum

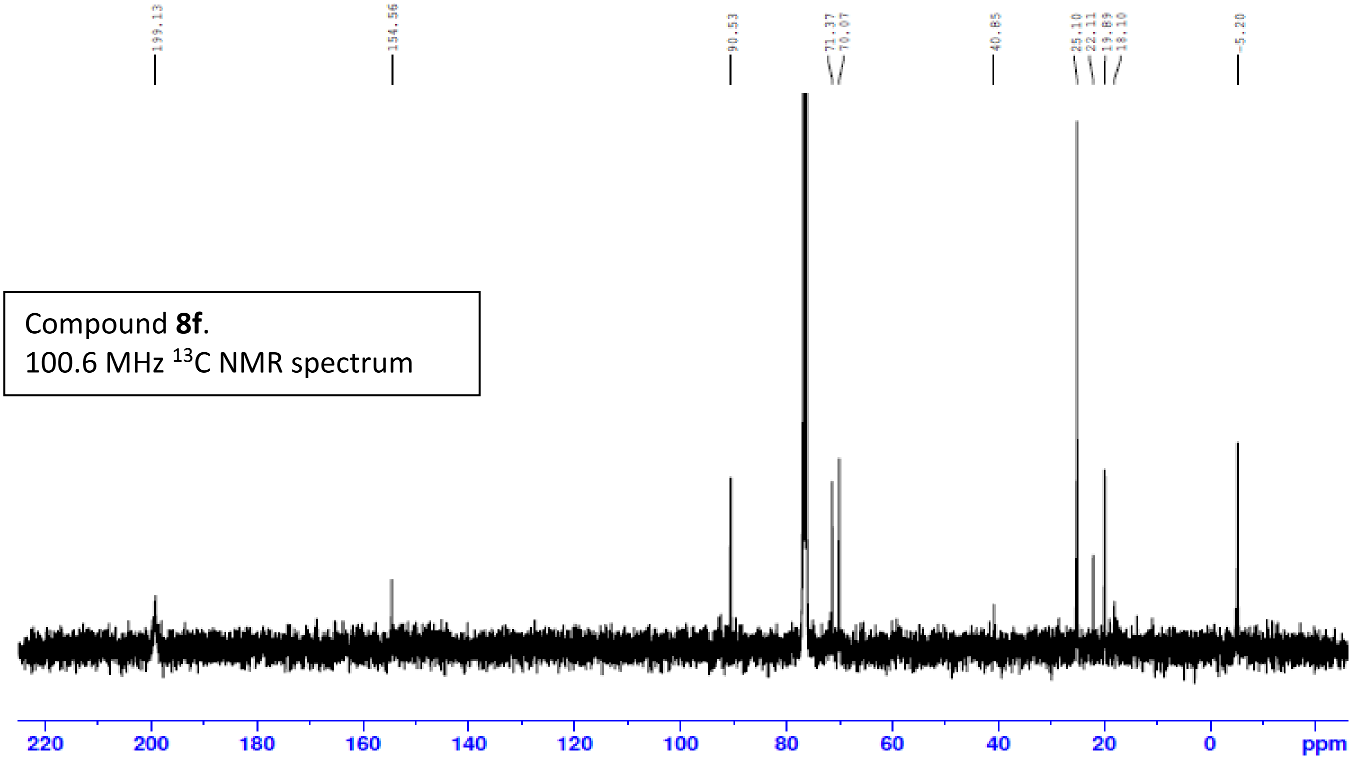

Compound **8g**.
400.13 MHz ^{1}H NMR spectrum

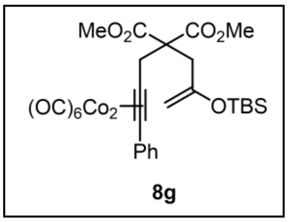

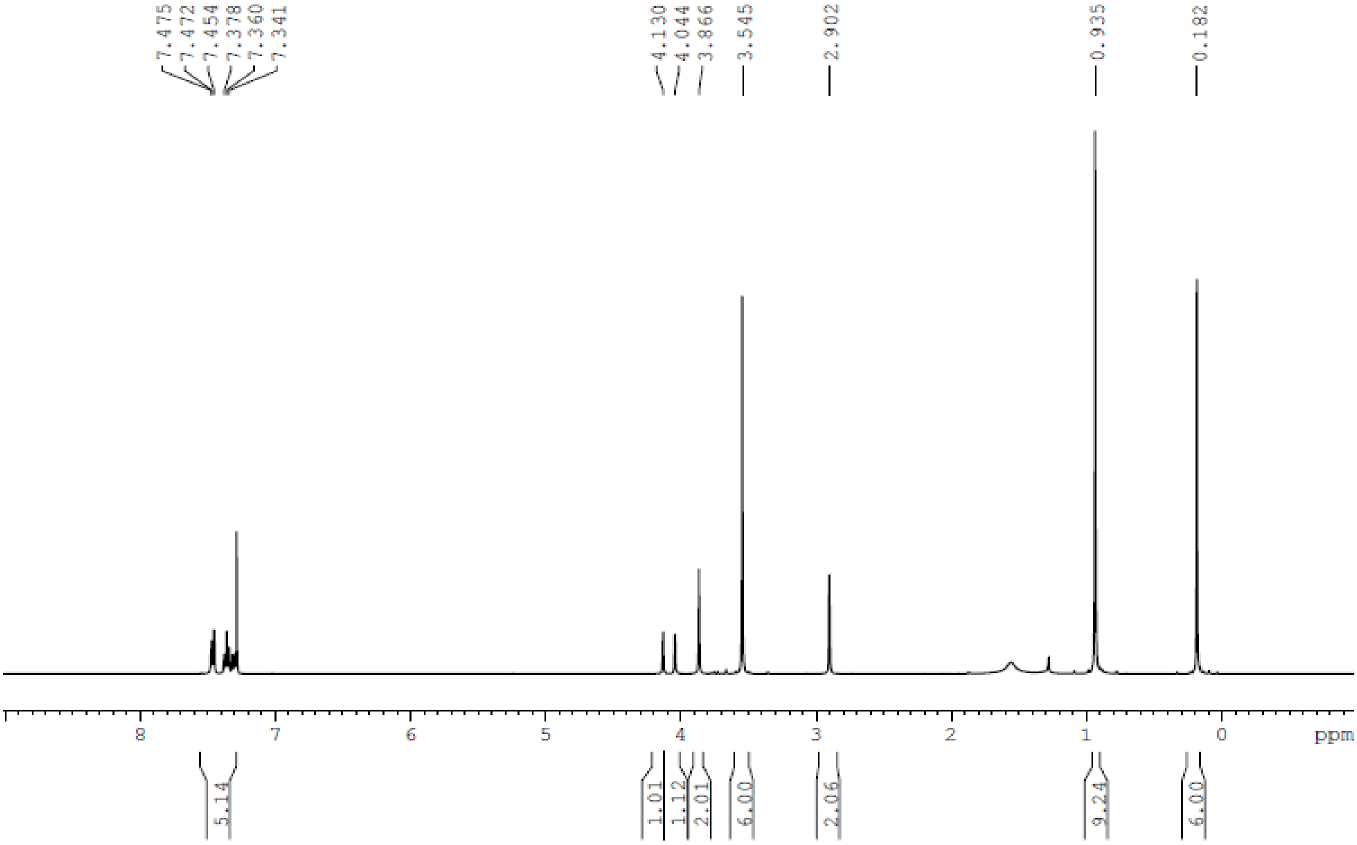

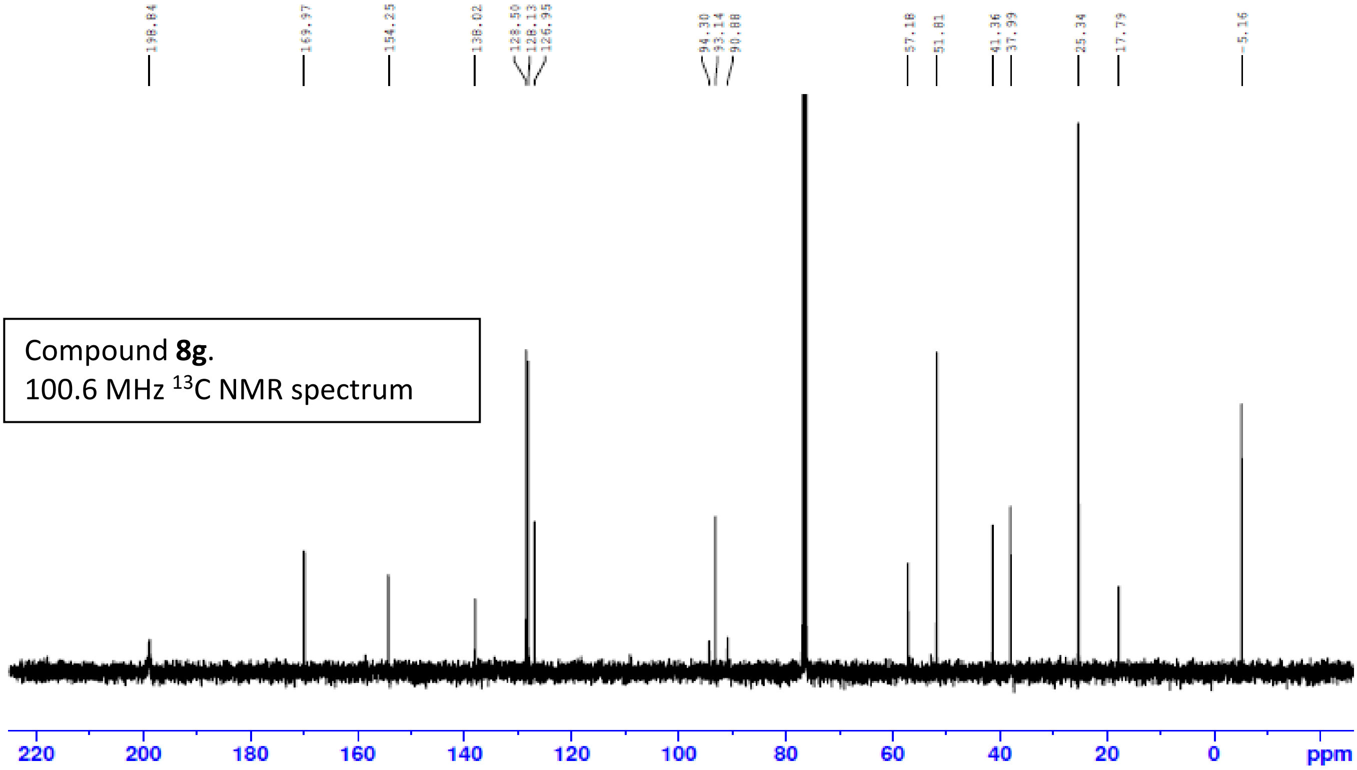

Compound **8g**.
100.6 MHz ^{13}C NMR spectrum

Compound **8h**.
400.13 MHz ^{1}H NMR spectrum

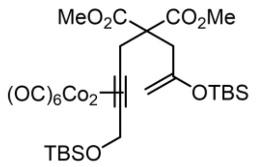

8h

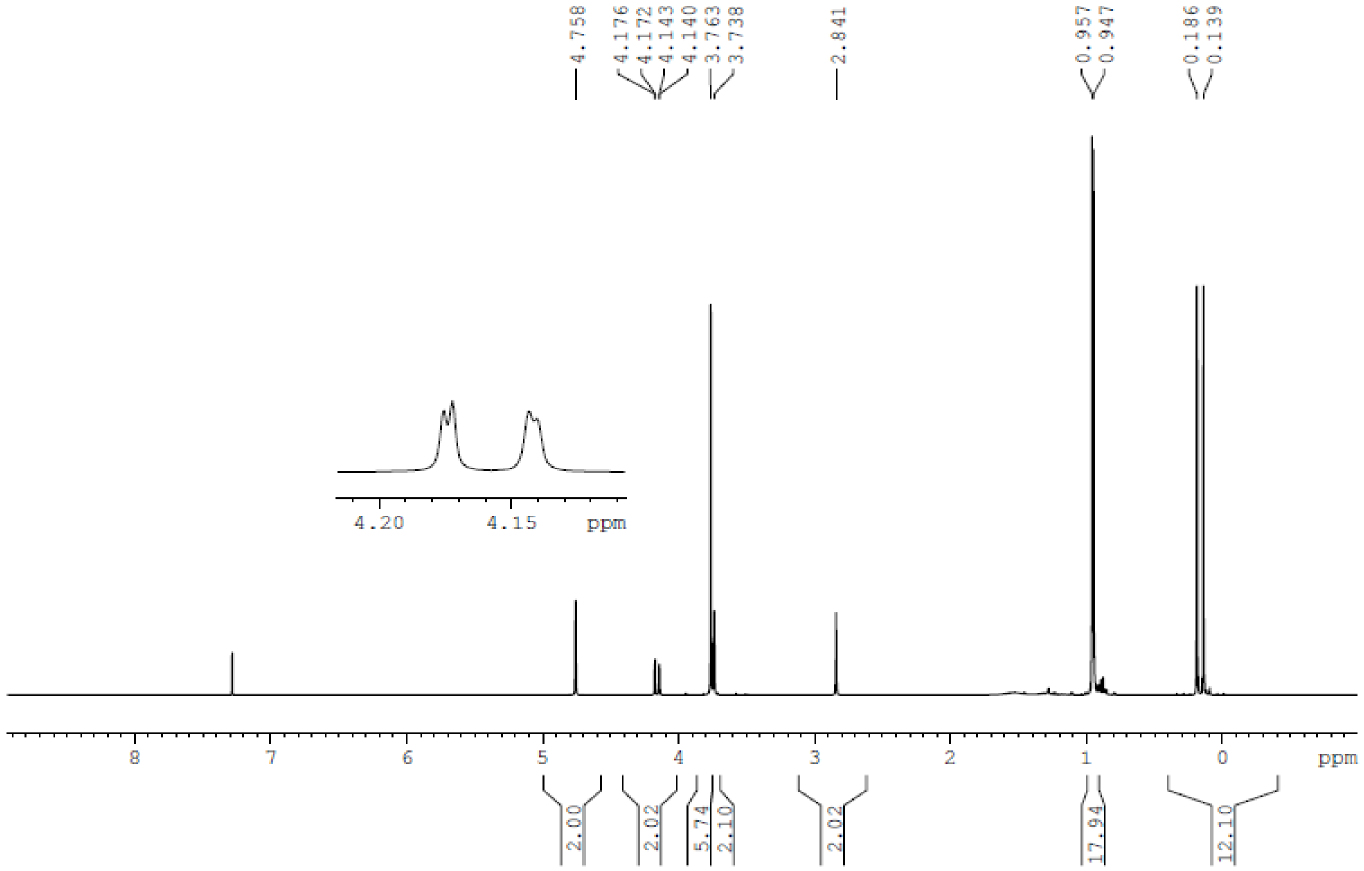

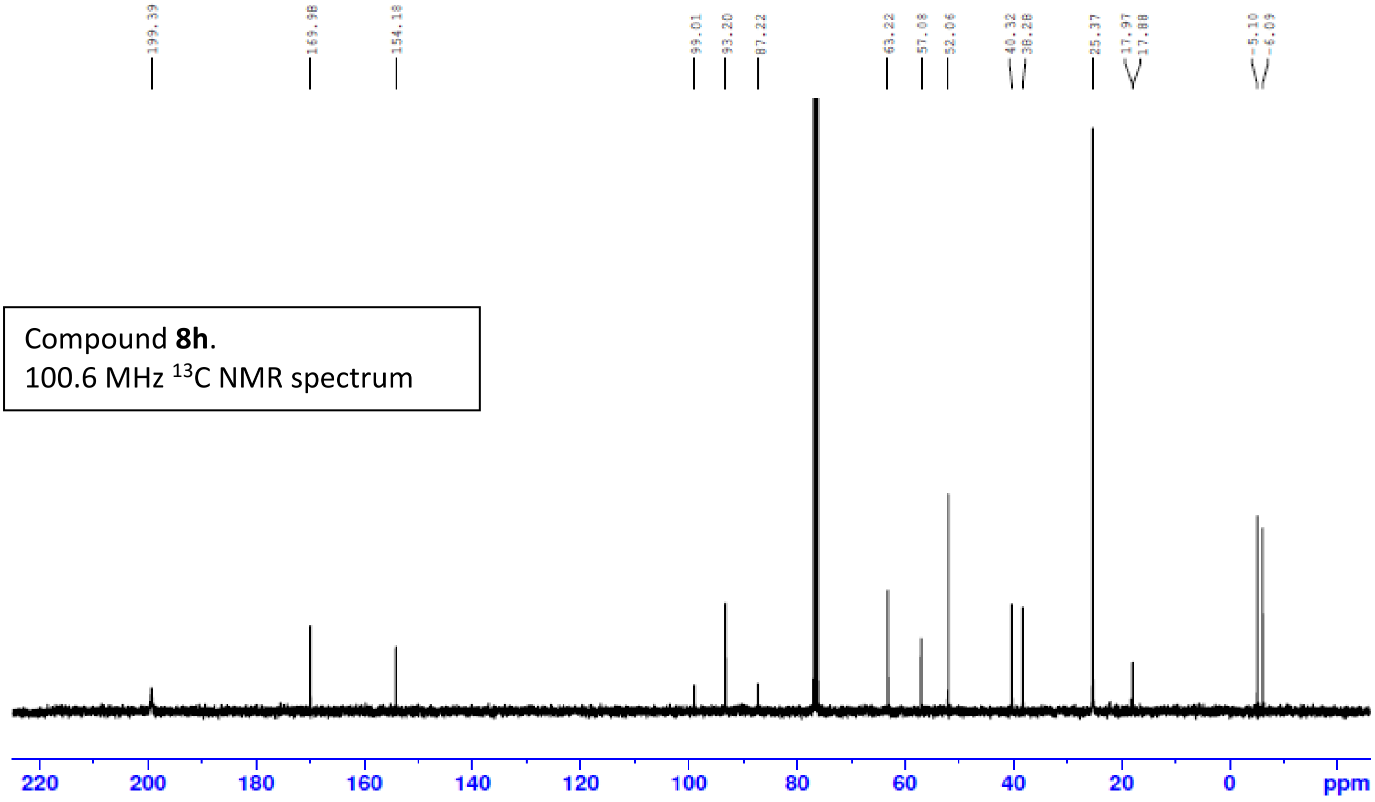

Compound **8h**.
100.6 MHz ^{13}C NMR spectrum

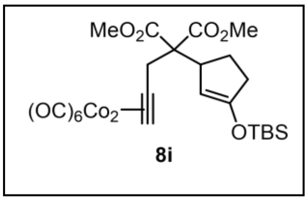

Compound **8i**.
400.13 MHz ^{1}H NMR spectrum

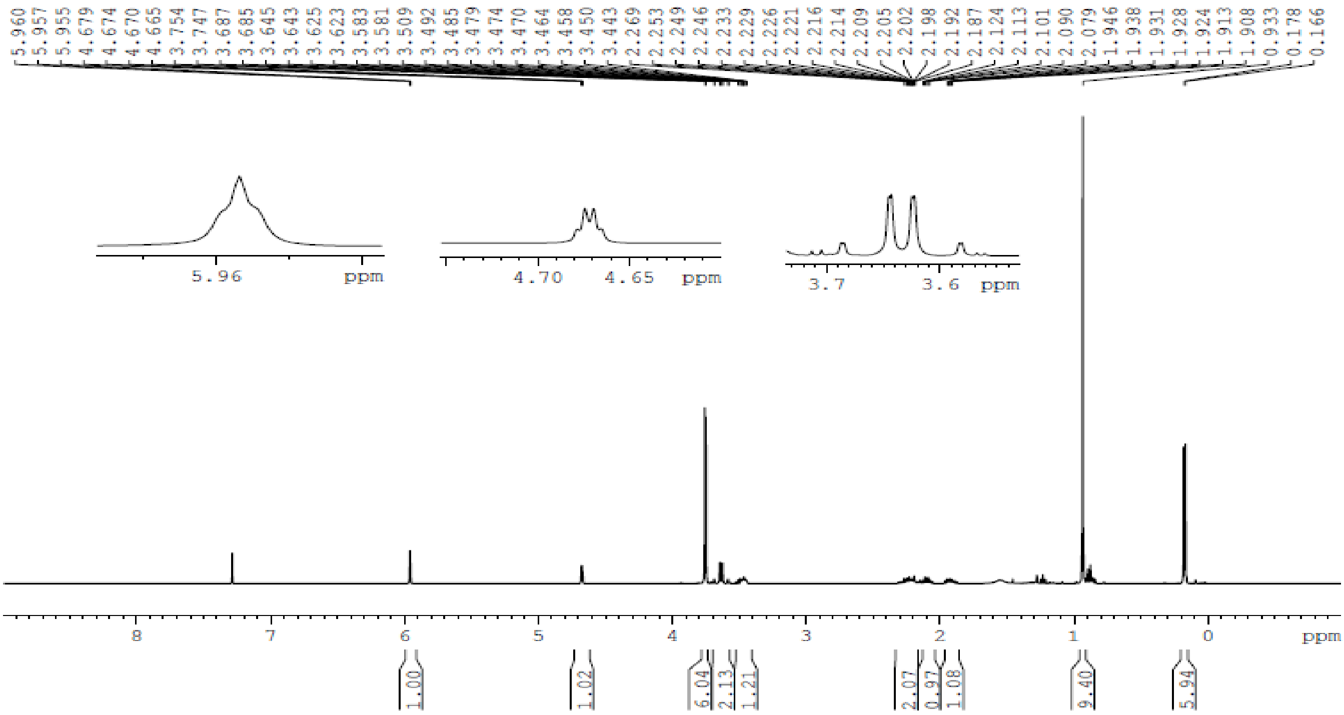

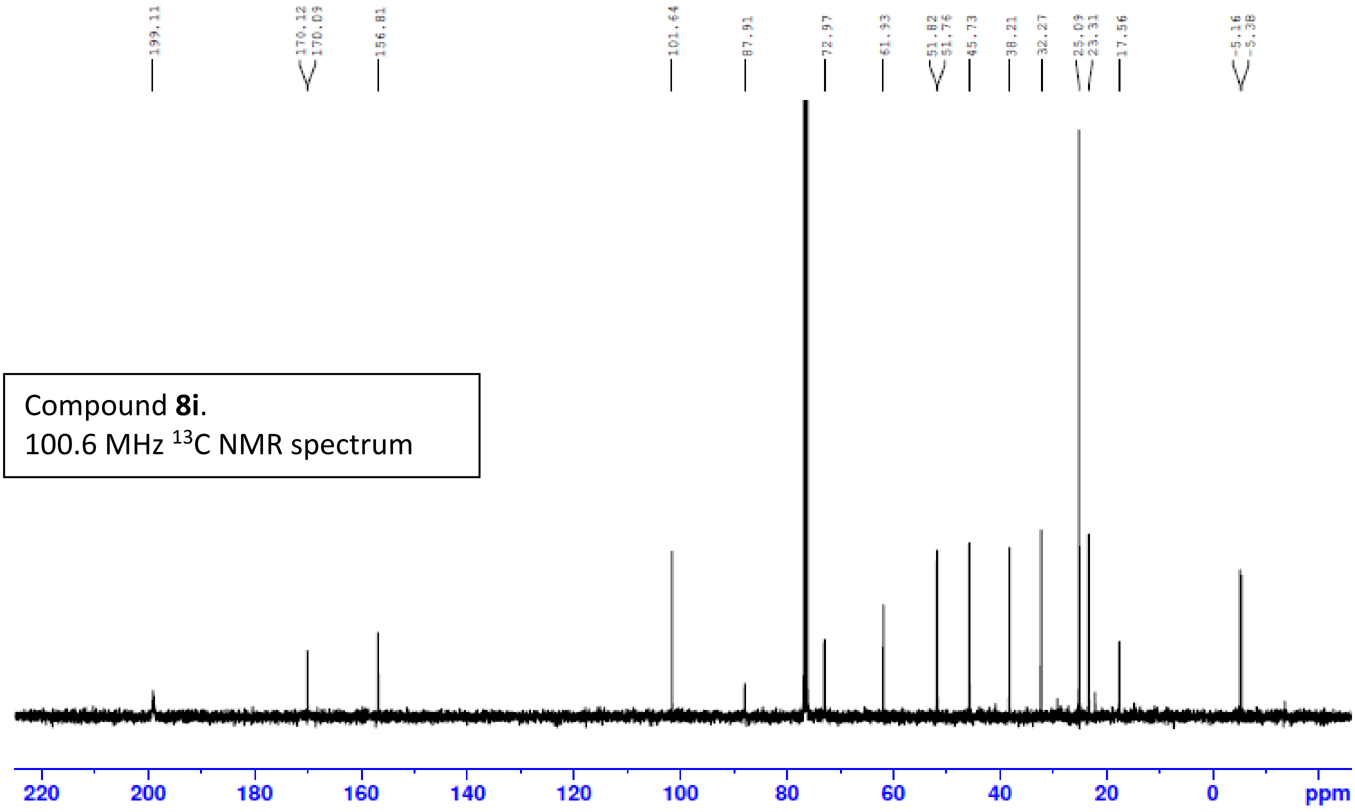

Compound **8i**.
100.6 MHz ^{13}C NMR spectrum

Compound **8j**.
400.13 MHz ^{1}H NMR spectrum

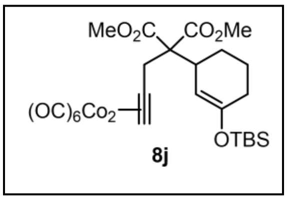

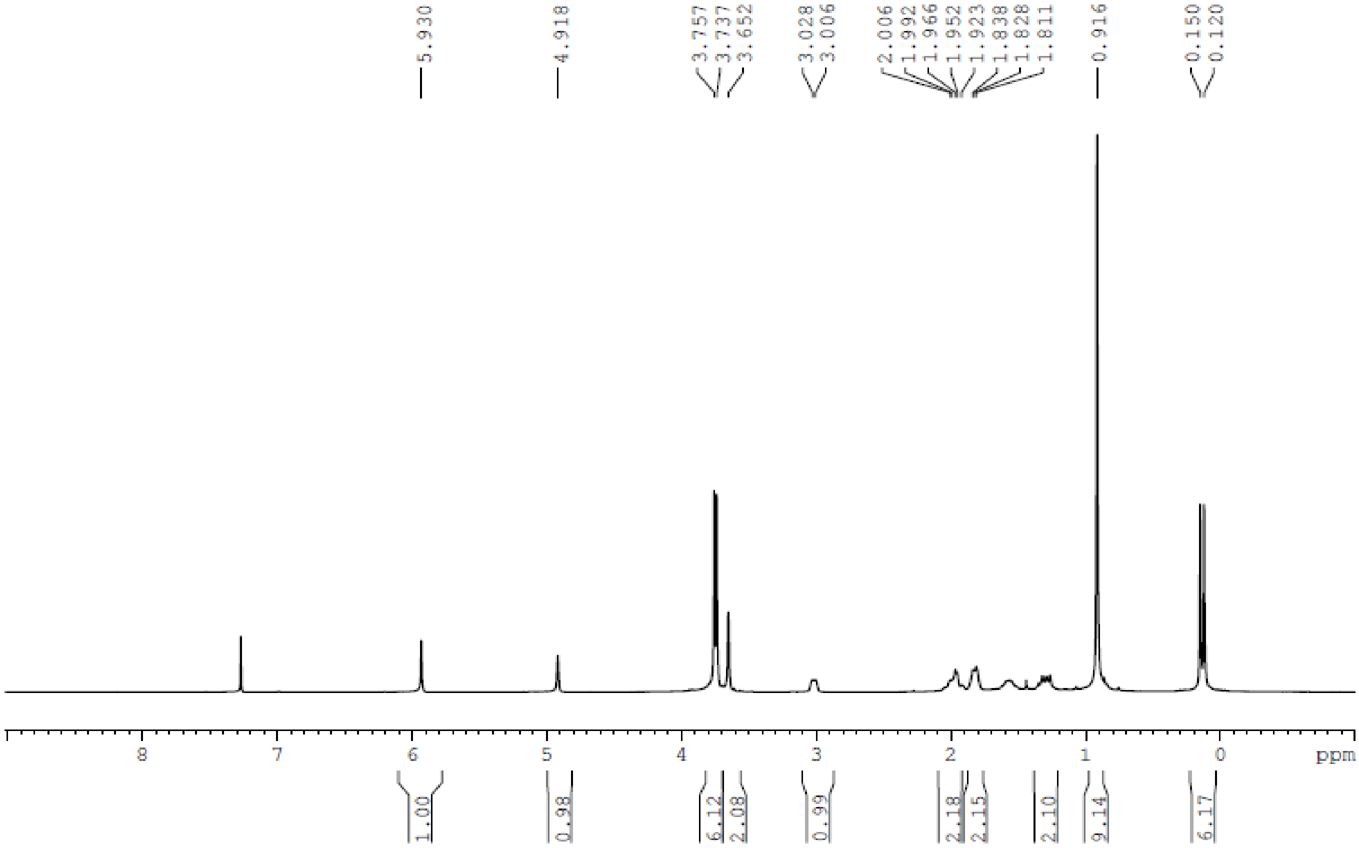

Compound **8j**.
100.6 MHz ^{13}C NMR spectrum

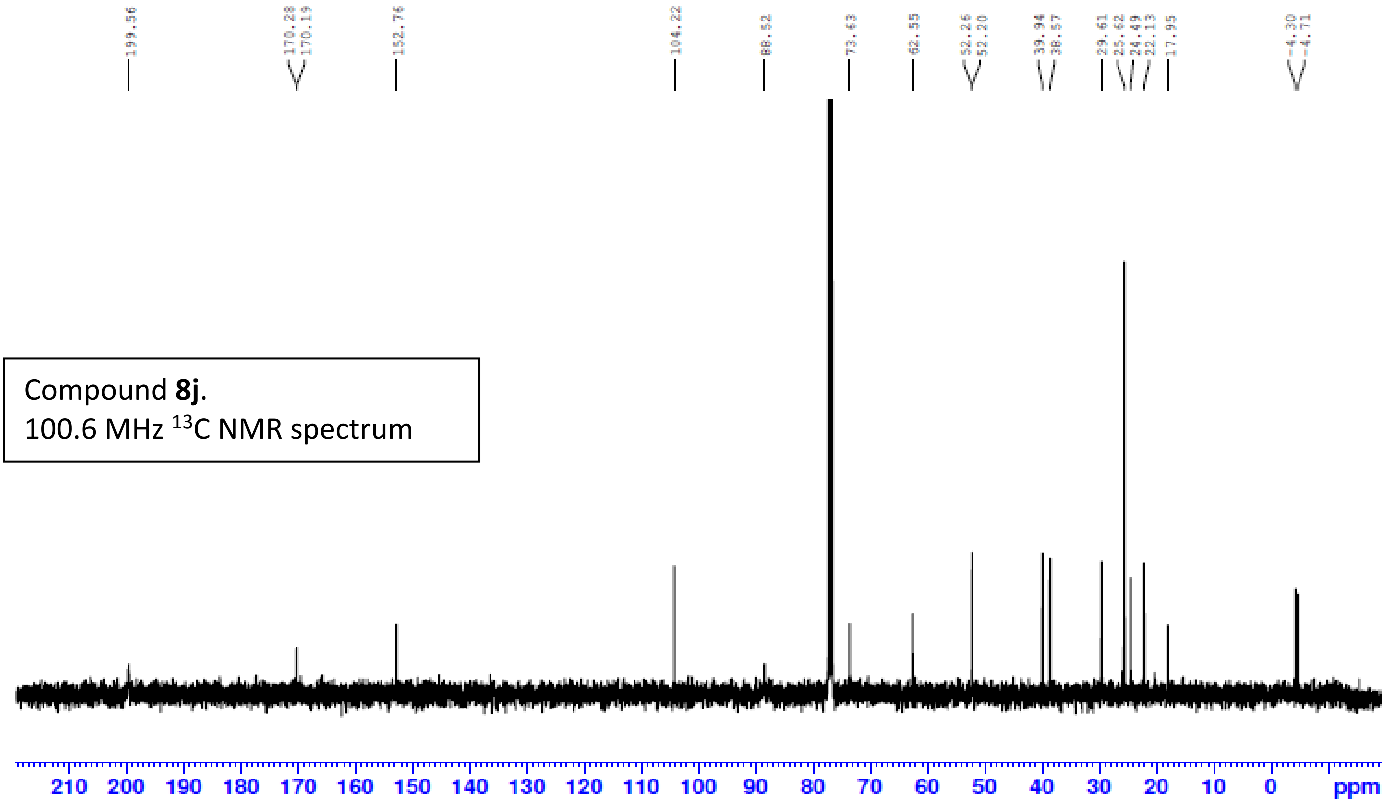

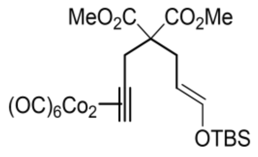

***trans*-10a**

Compound ***trans*-10a**.
400.13 MHz ^{1}H NMR spectrum
$CDCl_3$

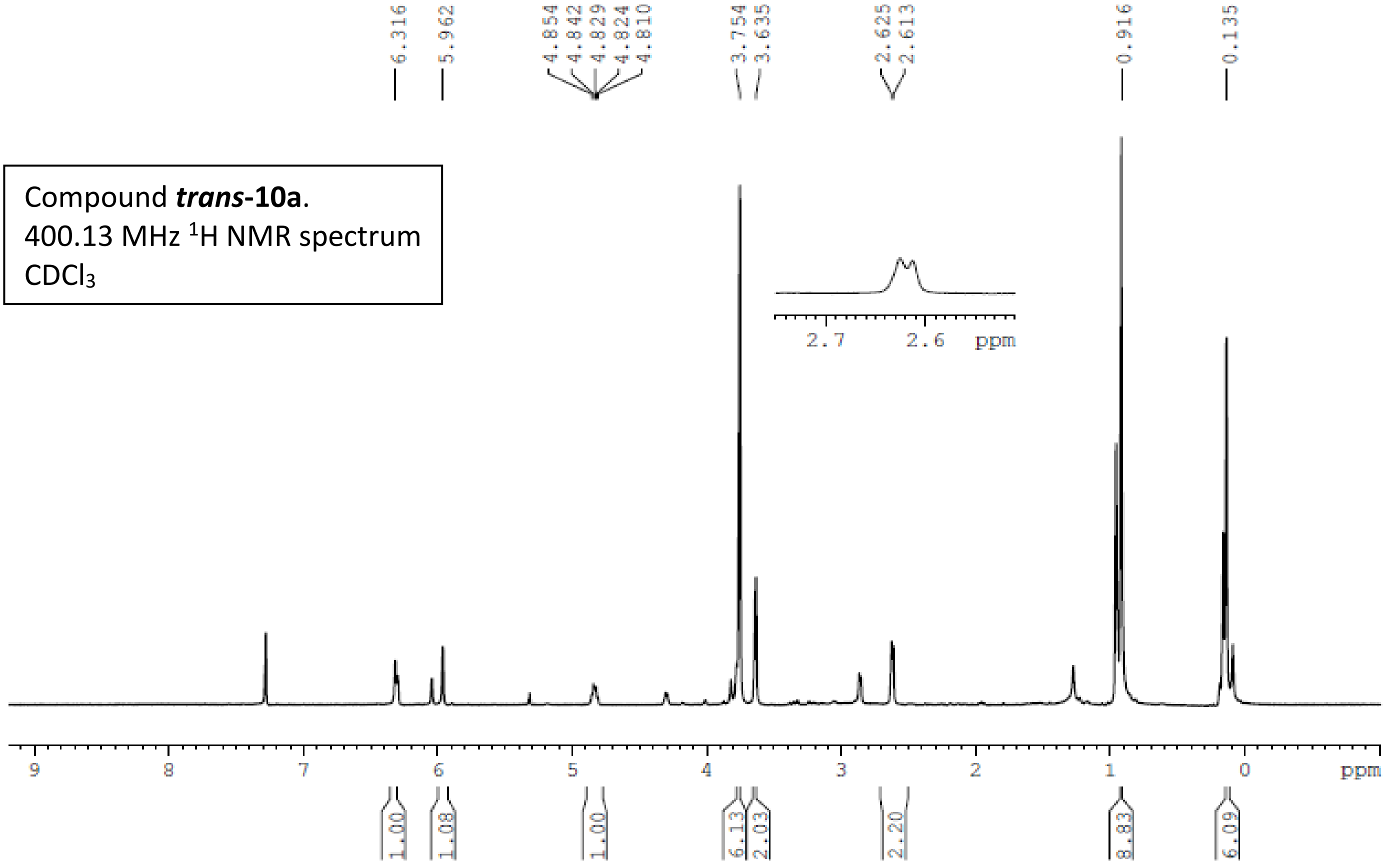

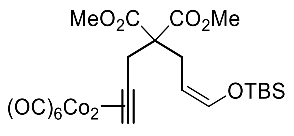

***cis*-10a**

Compound ***cis*-10a**.
400.13 MHz ^{1}H NMR spectrum
$CDCl_3$

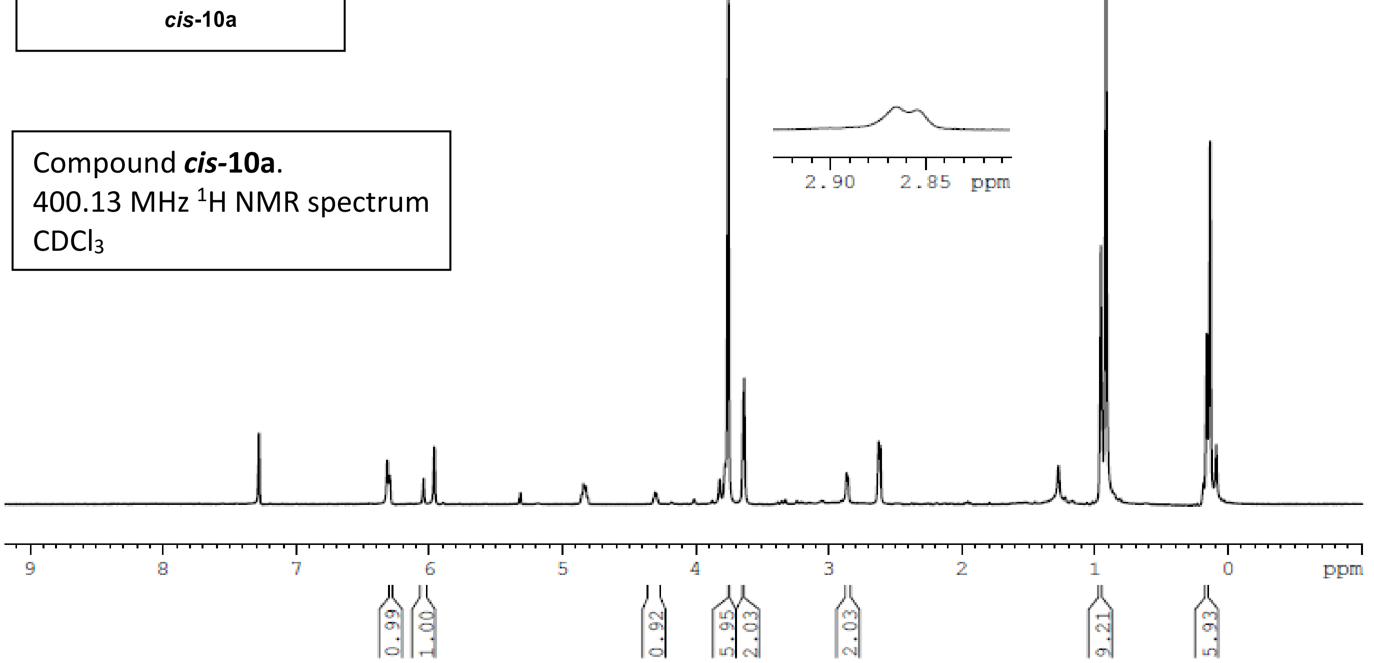

Compound **10a**.
400.13 MHz ^{1}H NMR spectrum
$CDCl_3$

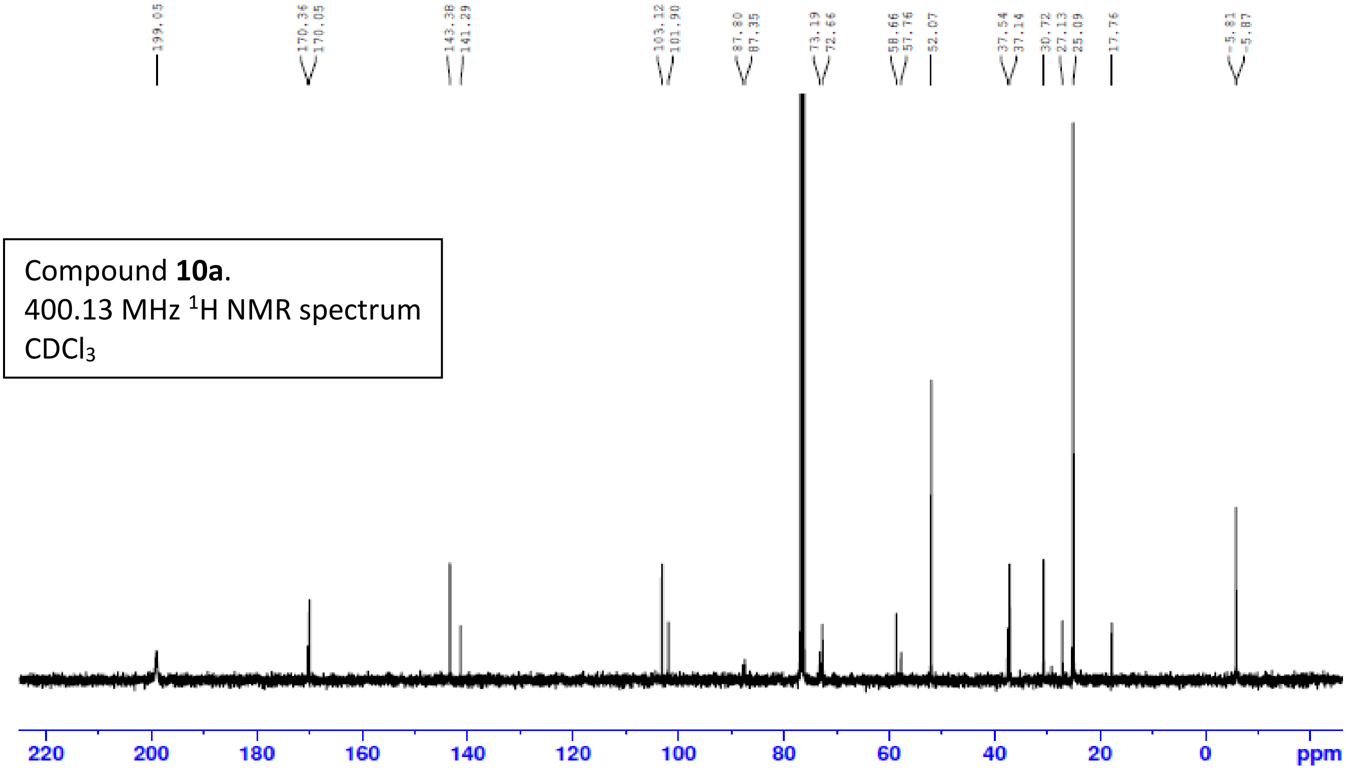

Compound ***trans*-10b**.
400.13 MHz ^{1}H NMR spectrum

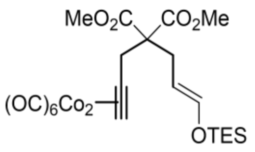

***trans*-10b**

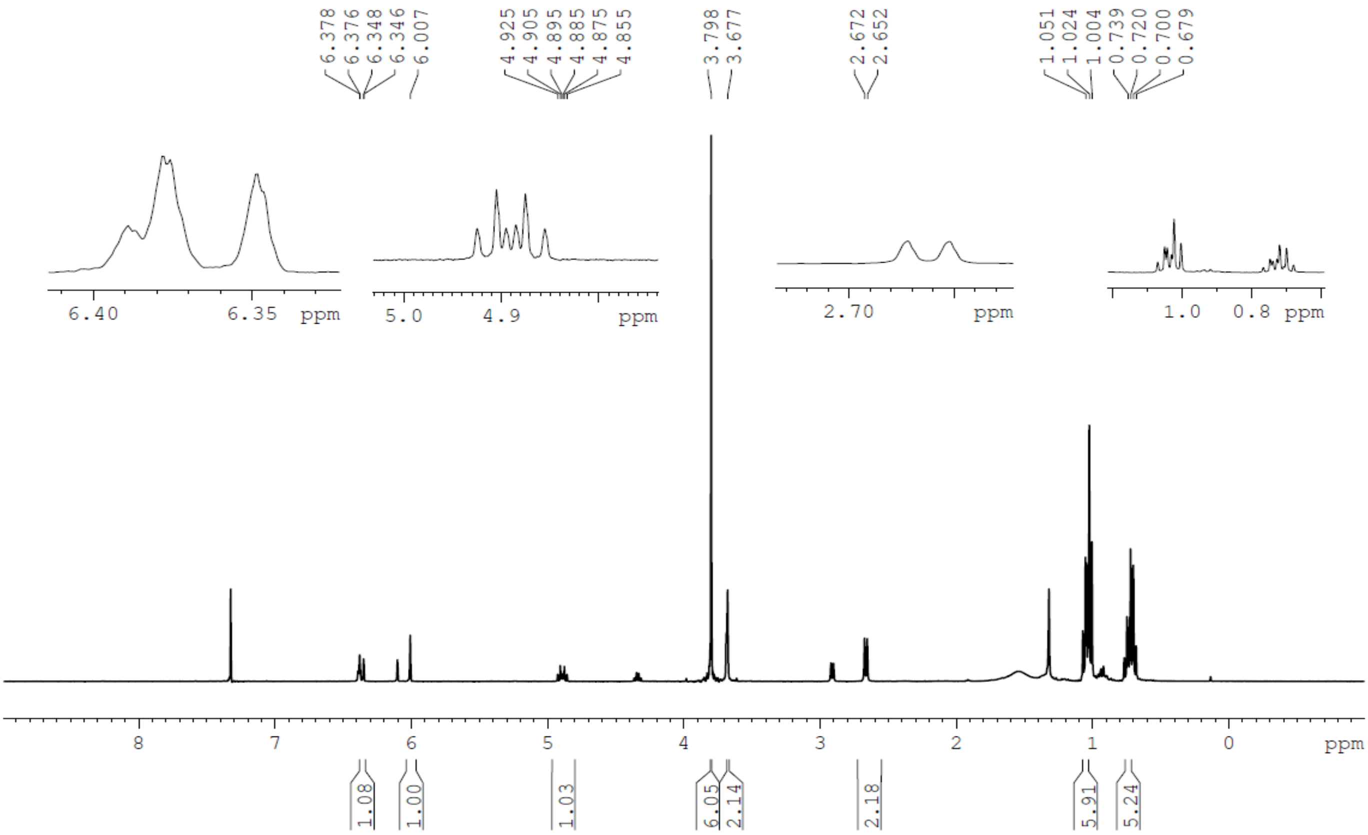

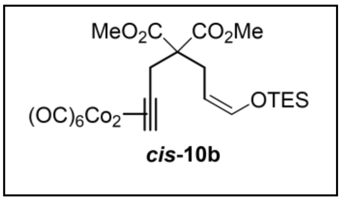

Compound ***cis*-10b**.
400.13 MHz ^{1}H NMR spectrum

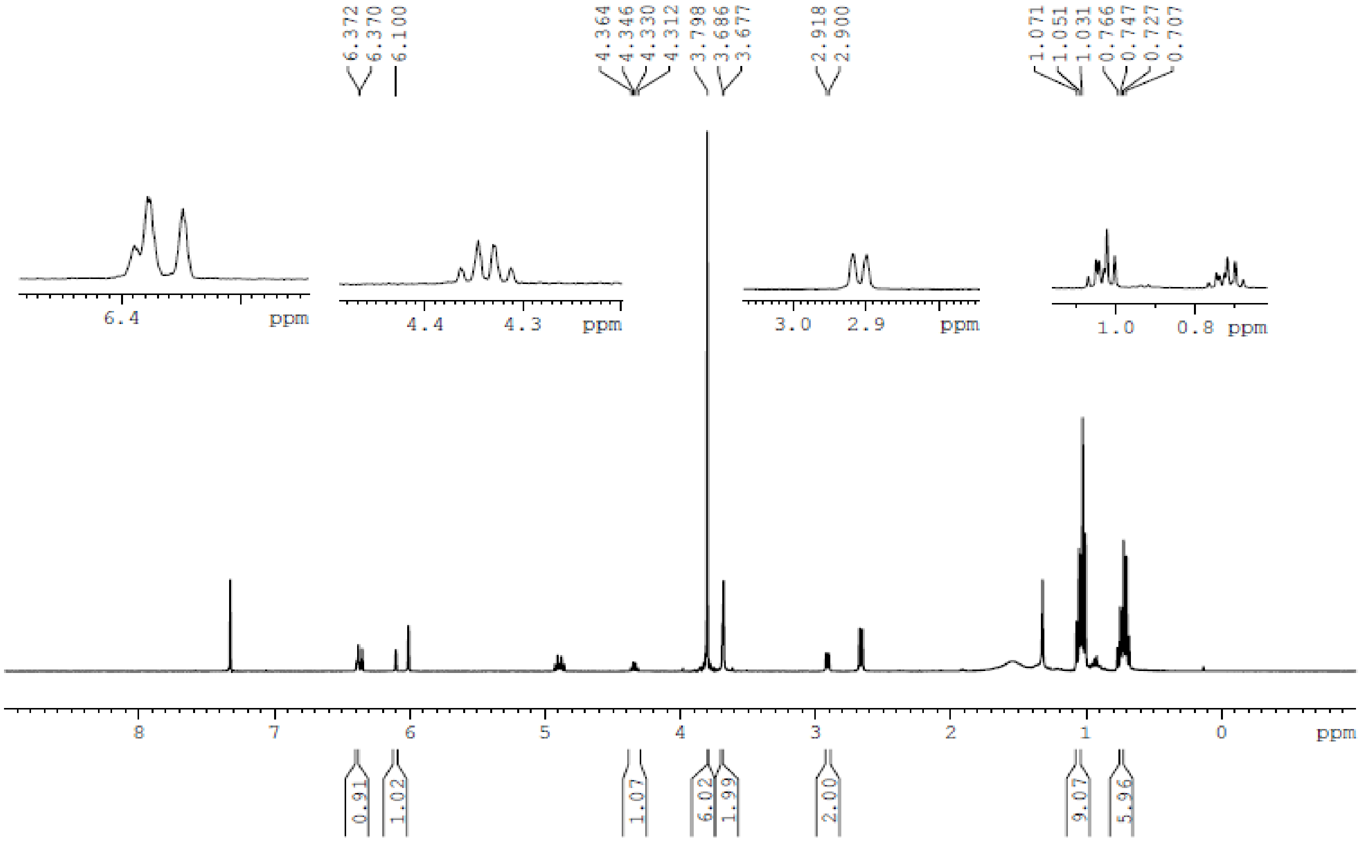

Compound **10b**.
100.6 MHz ^{13}C NMR spectrum

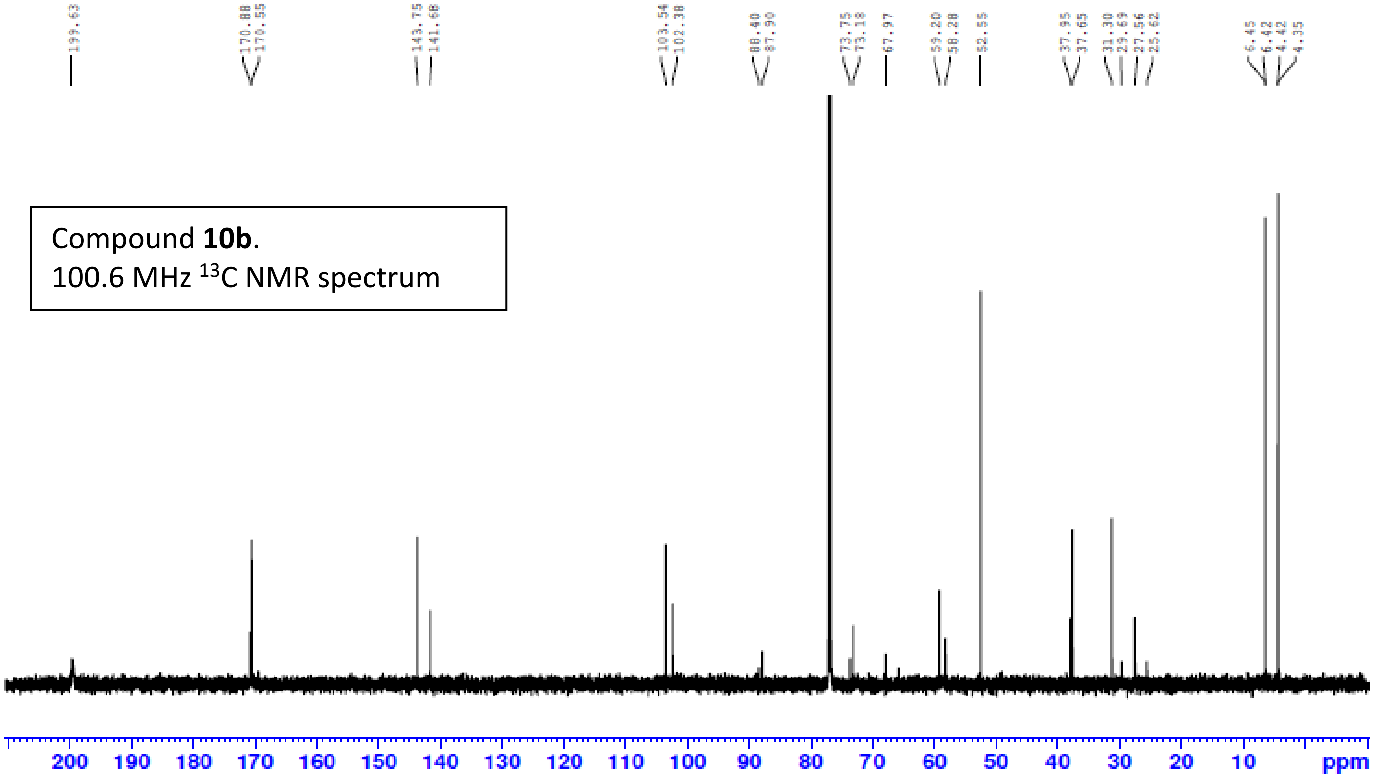

Compound **10c**.
400.13 MHz ^{1}H NMR spectrum

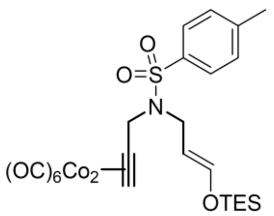

***trans*-10c**

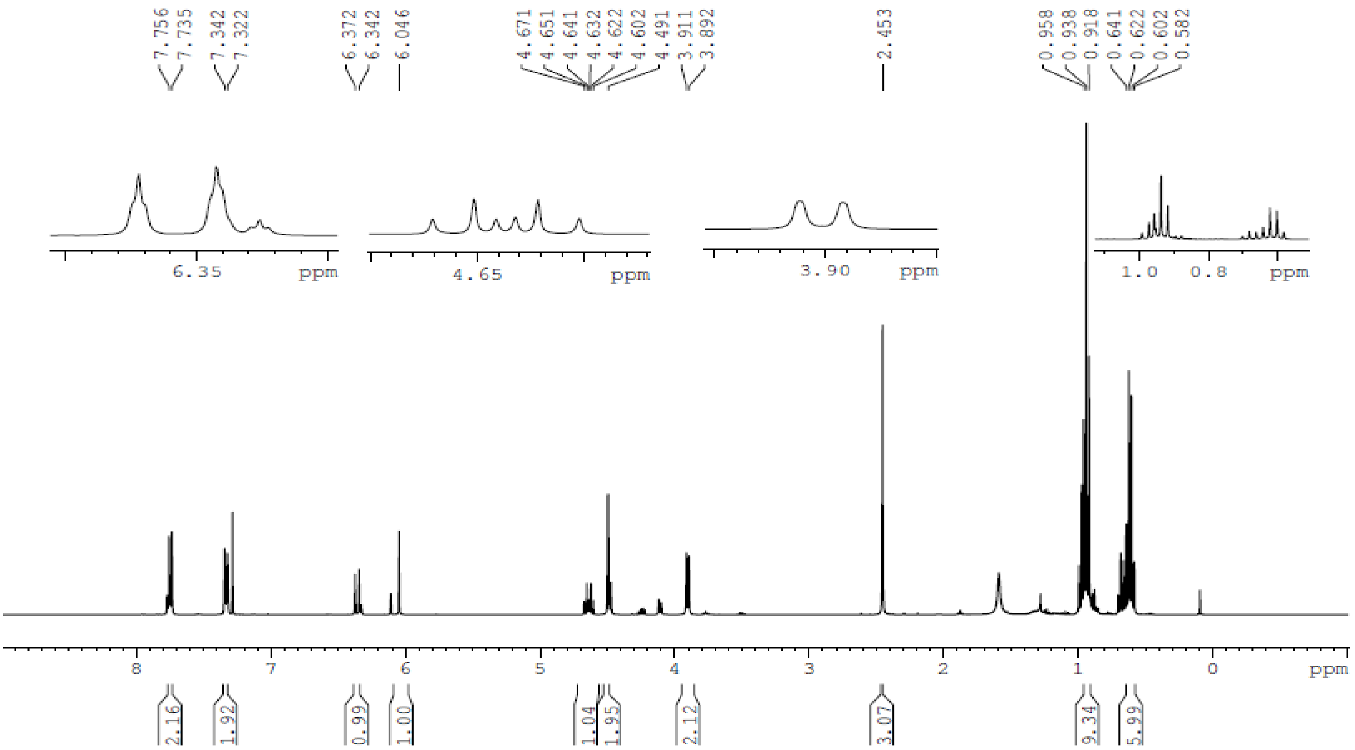

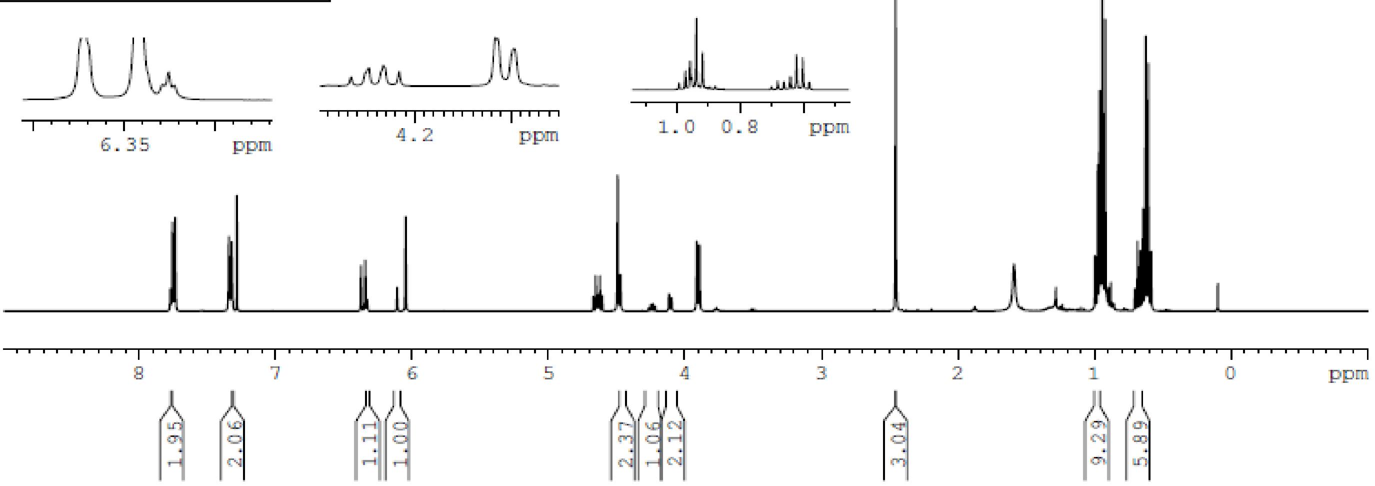

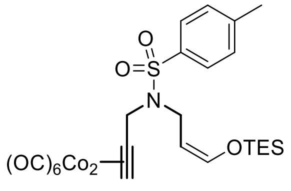

***cis*-10c**

Compound **10c**.
100.6 MHz ^{13}C NMR spectrum

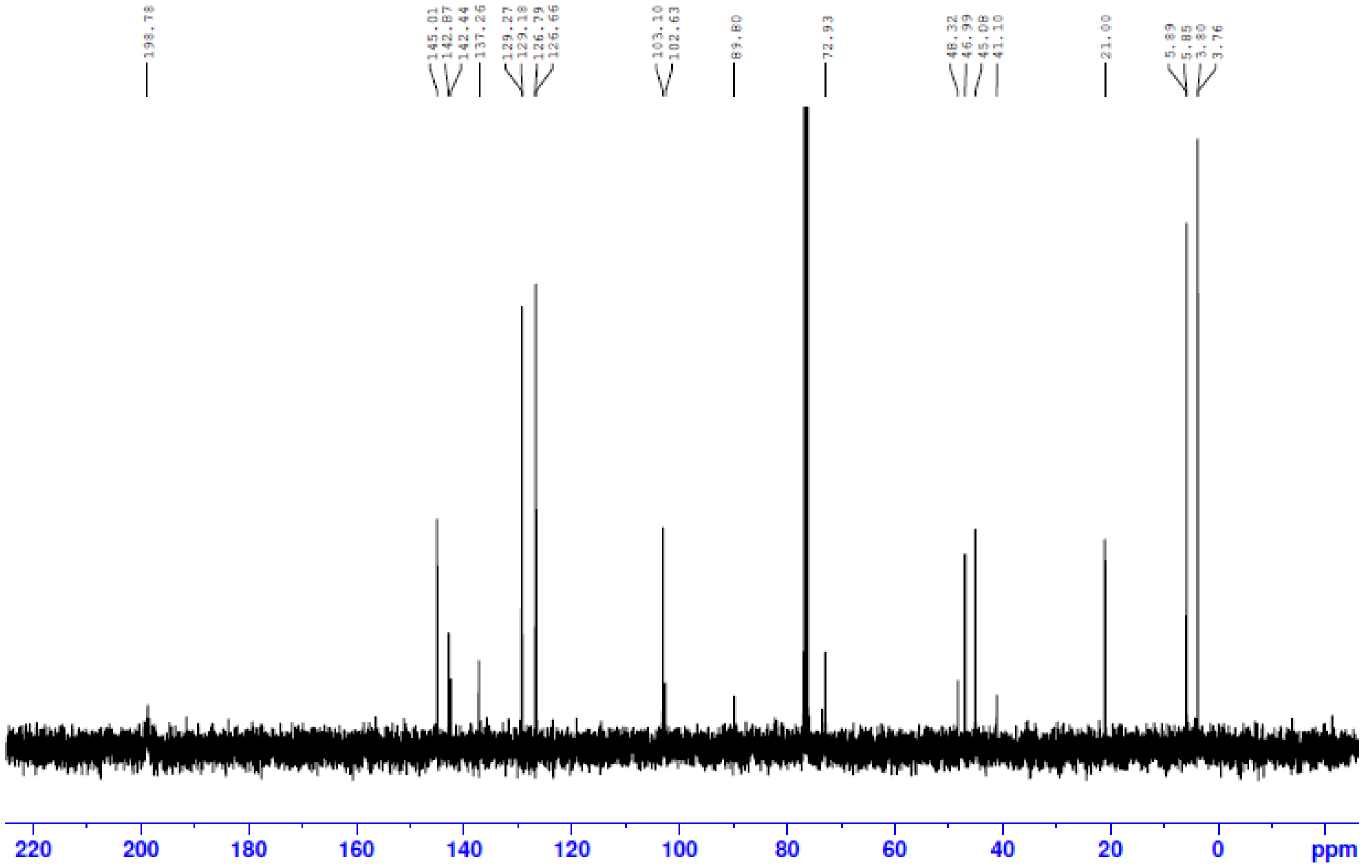

Compound **10d**.
400.13 MHz ^{1}H NMR spectrum
$CDCl_3$

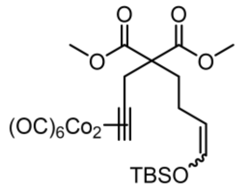

10d

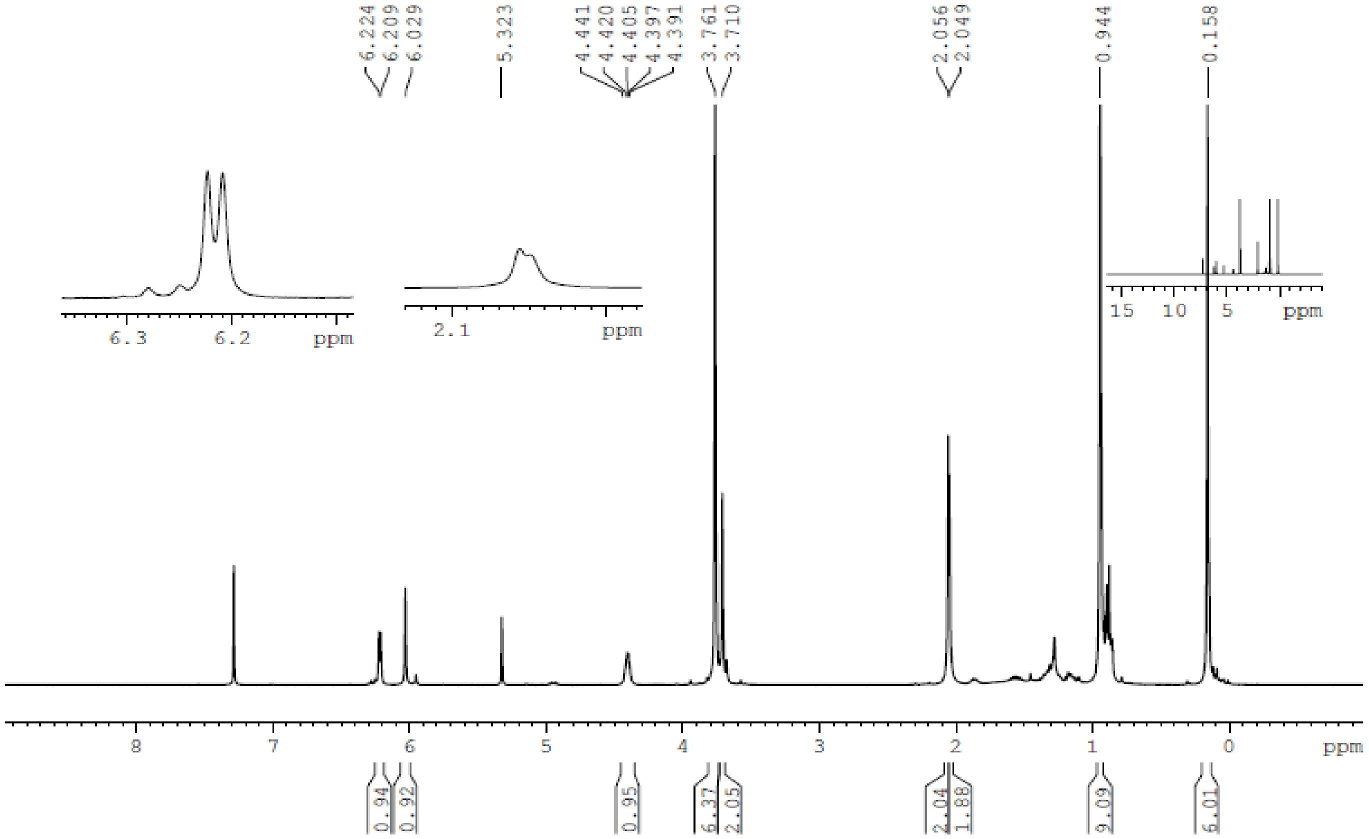

Compound **10d**.
100.6 MHz ^{13}C NMR spectrum
$CDCl_3$

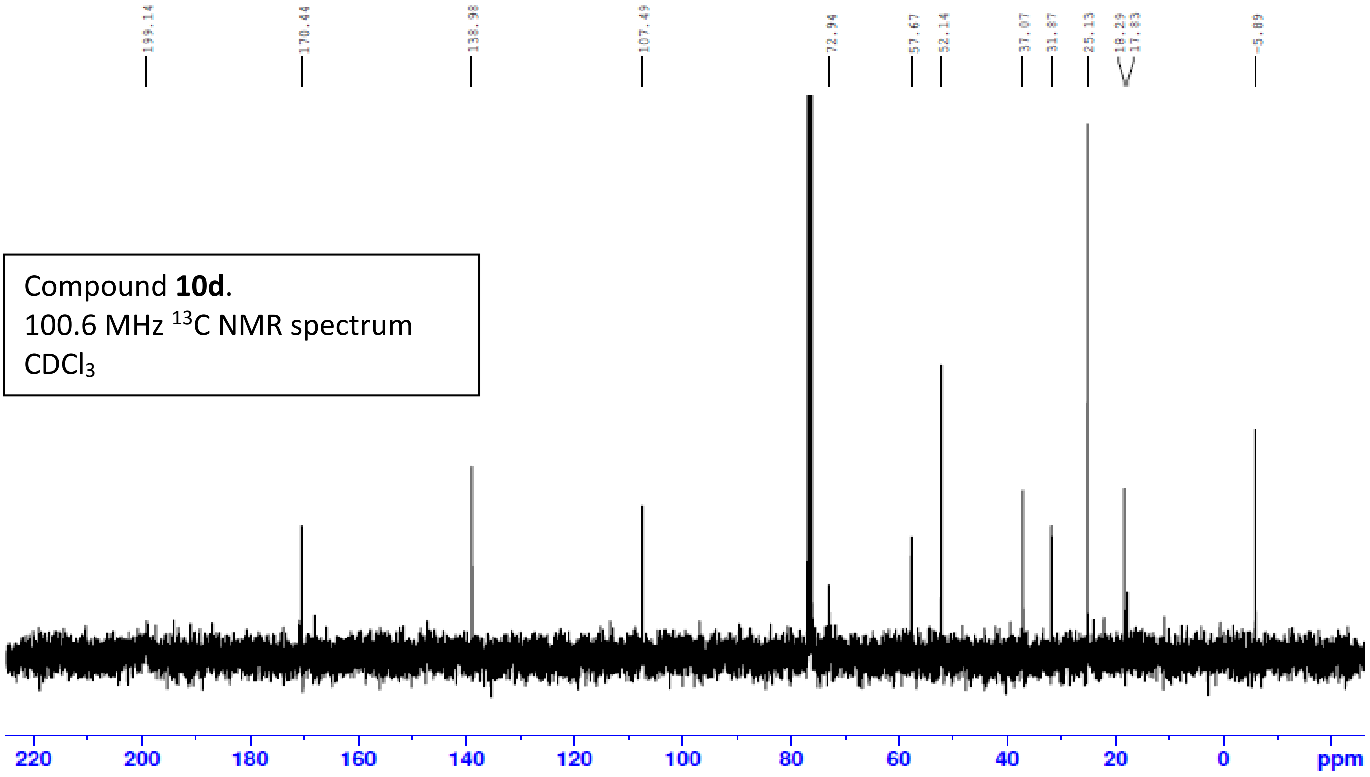

Compound **4**.
400.13 MHz ^{1}H NMR spectrum
$CDCl_3$

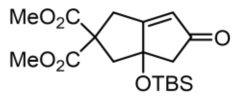

4

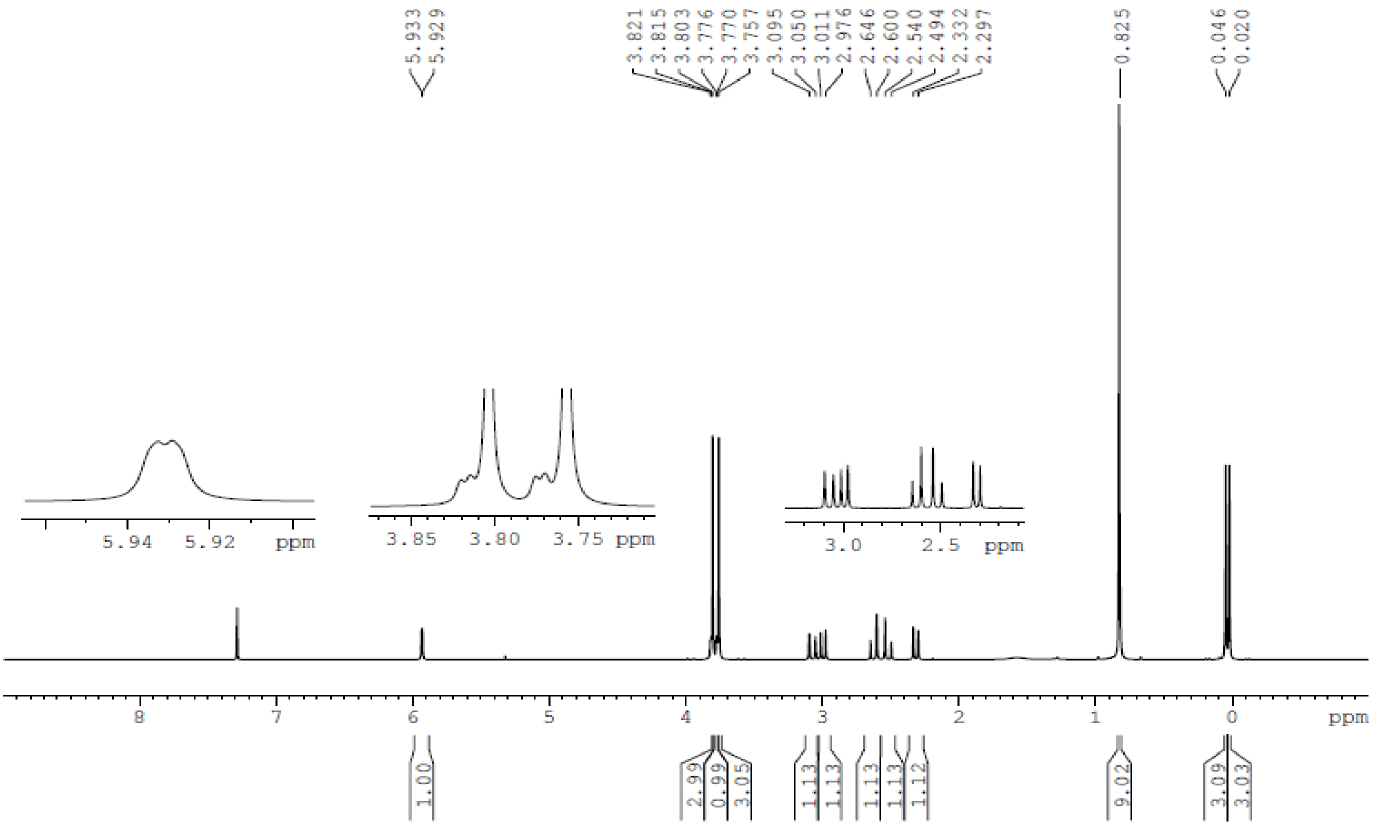

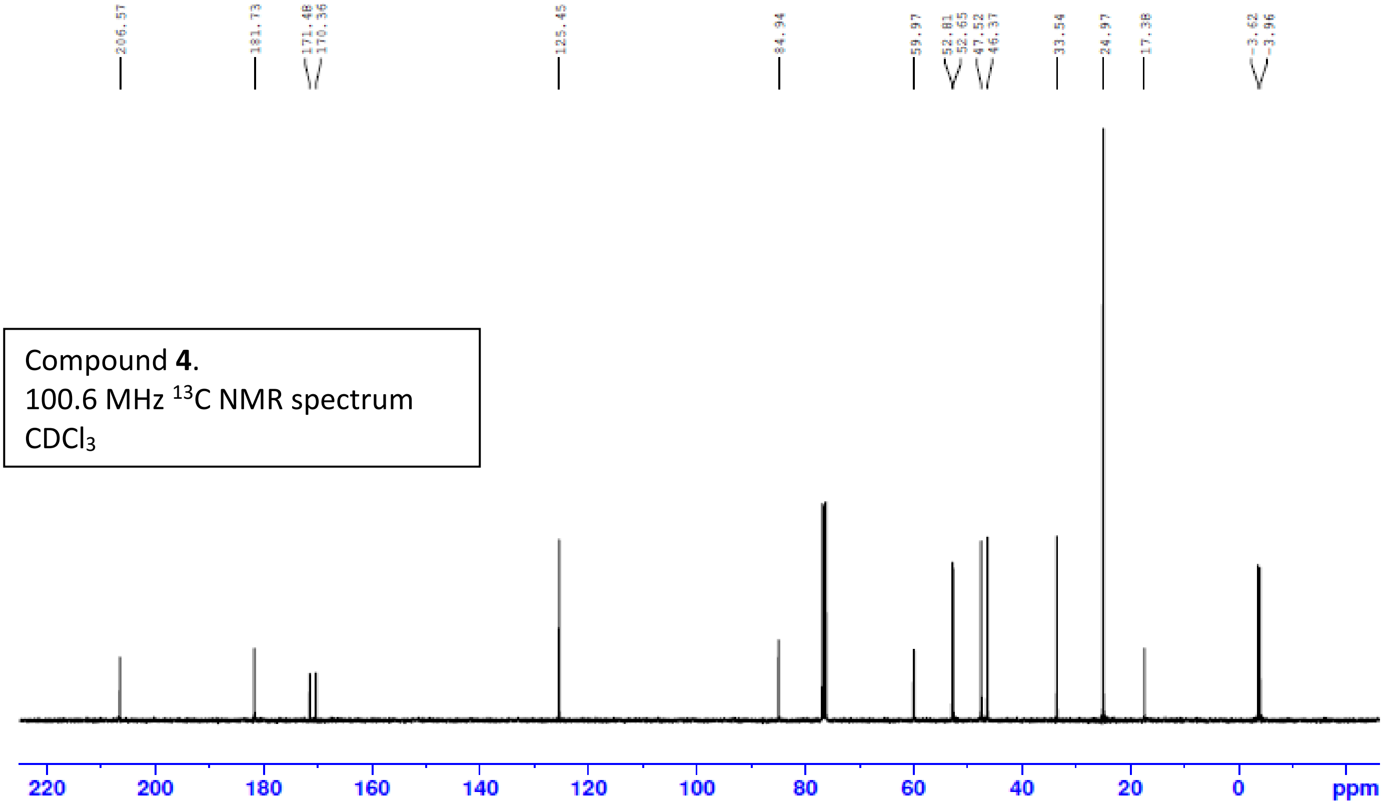

Compound **4**.
100.6 MHz ^{13}C NMR spectrum
$CDCl_3$

Compound **7a**.
400.13 MHz ^{1}H NMR spectrum
$CDCl_3$

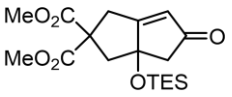

7a

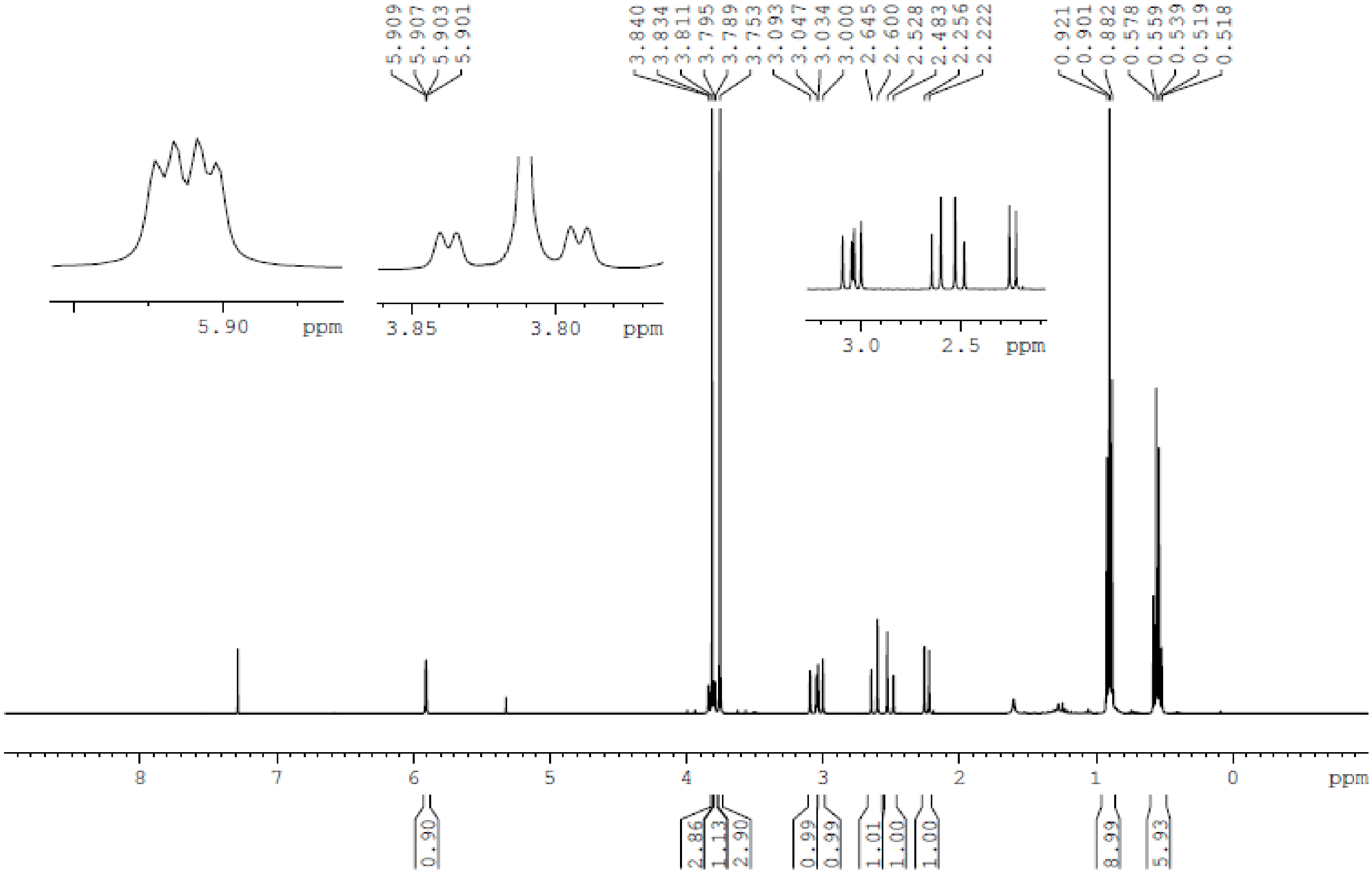

Compound **7a**.
100.6 MHz ^{13}C NMR spectrum
$CDCl_3$

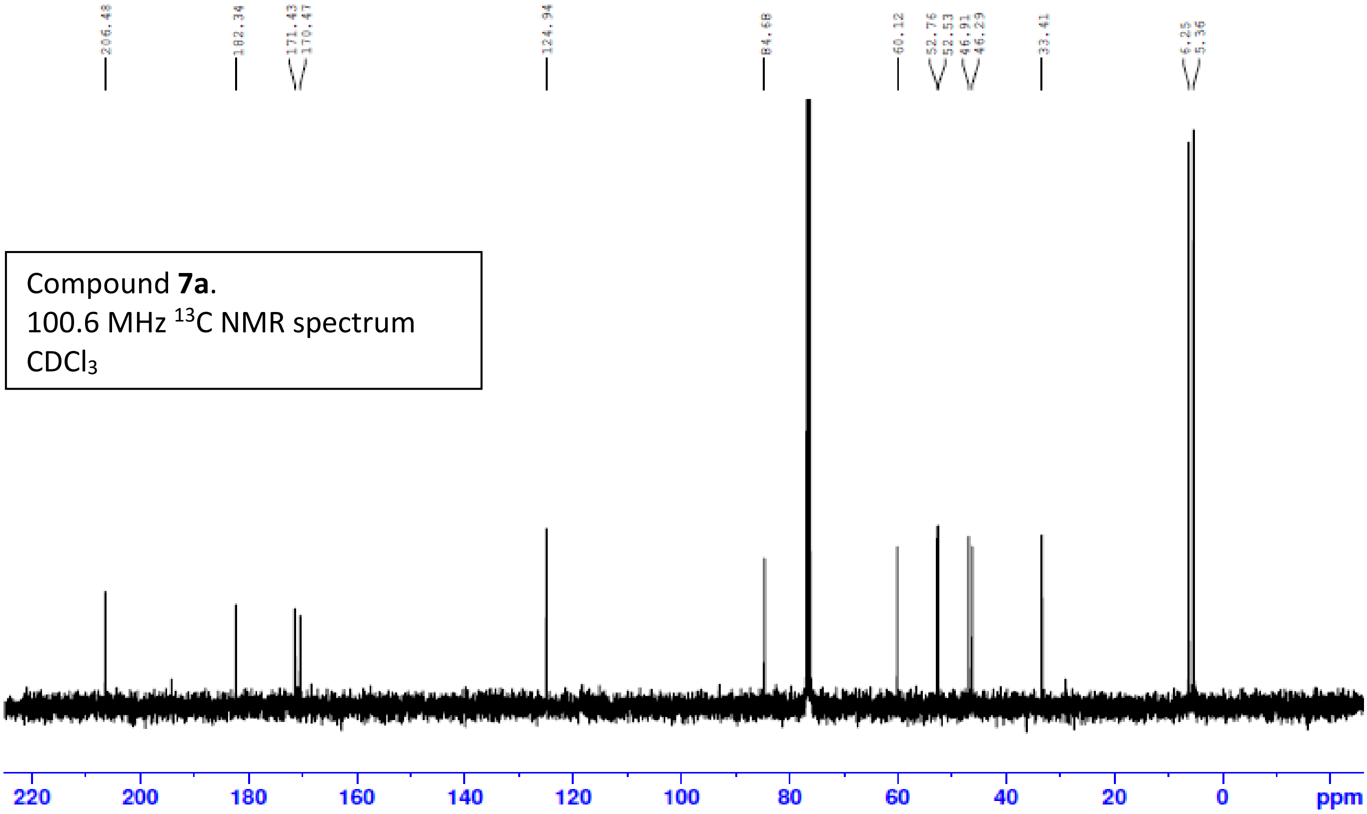

Compound **9a**.
400.13 MHz ^{1}H NMR spectrum
$CDCl_3$

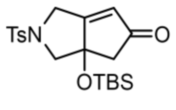

9a

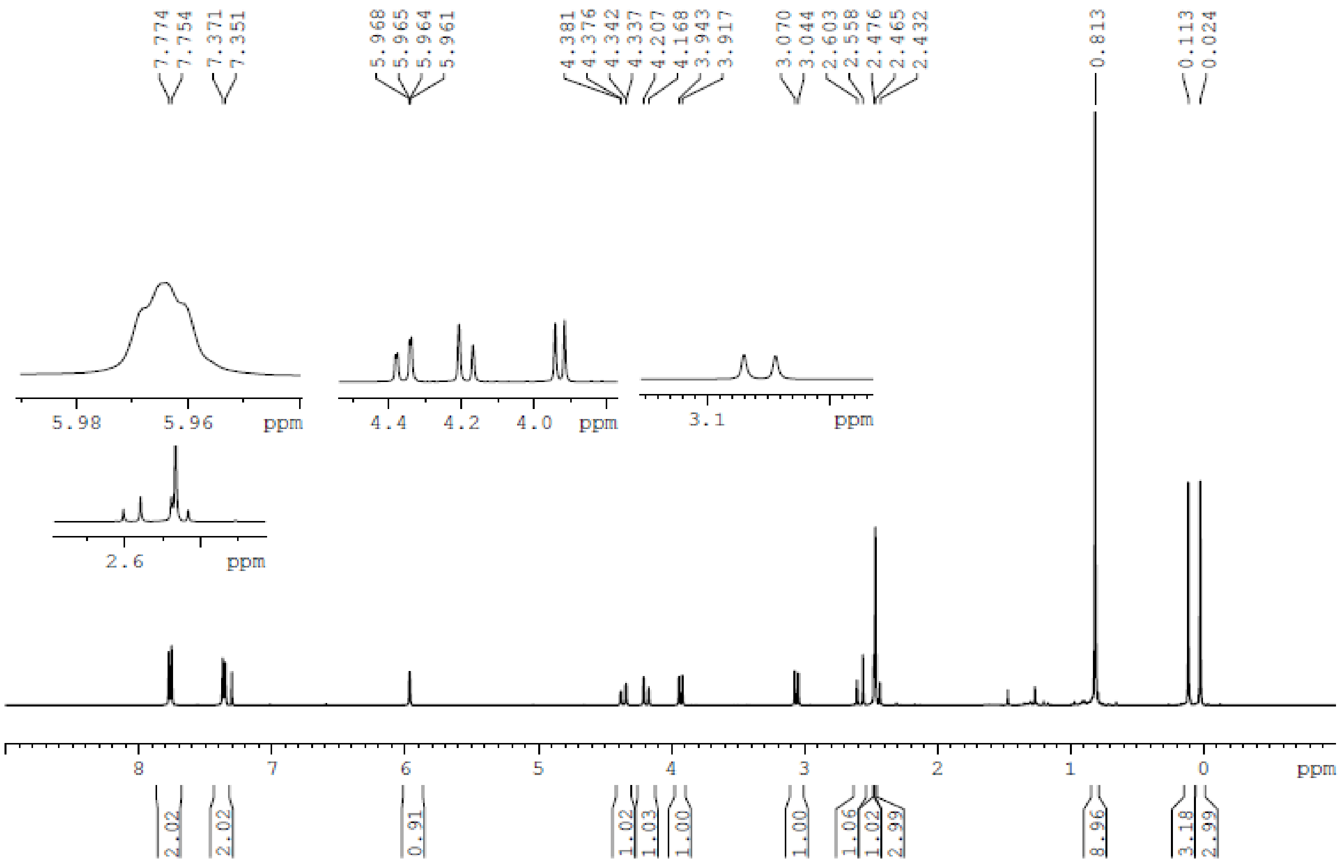

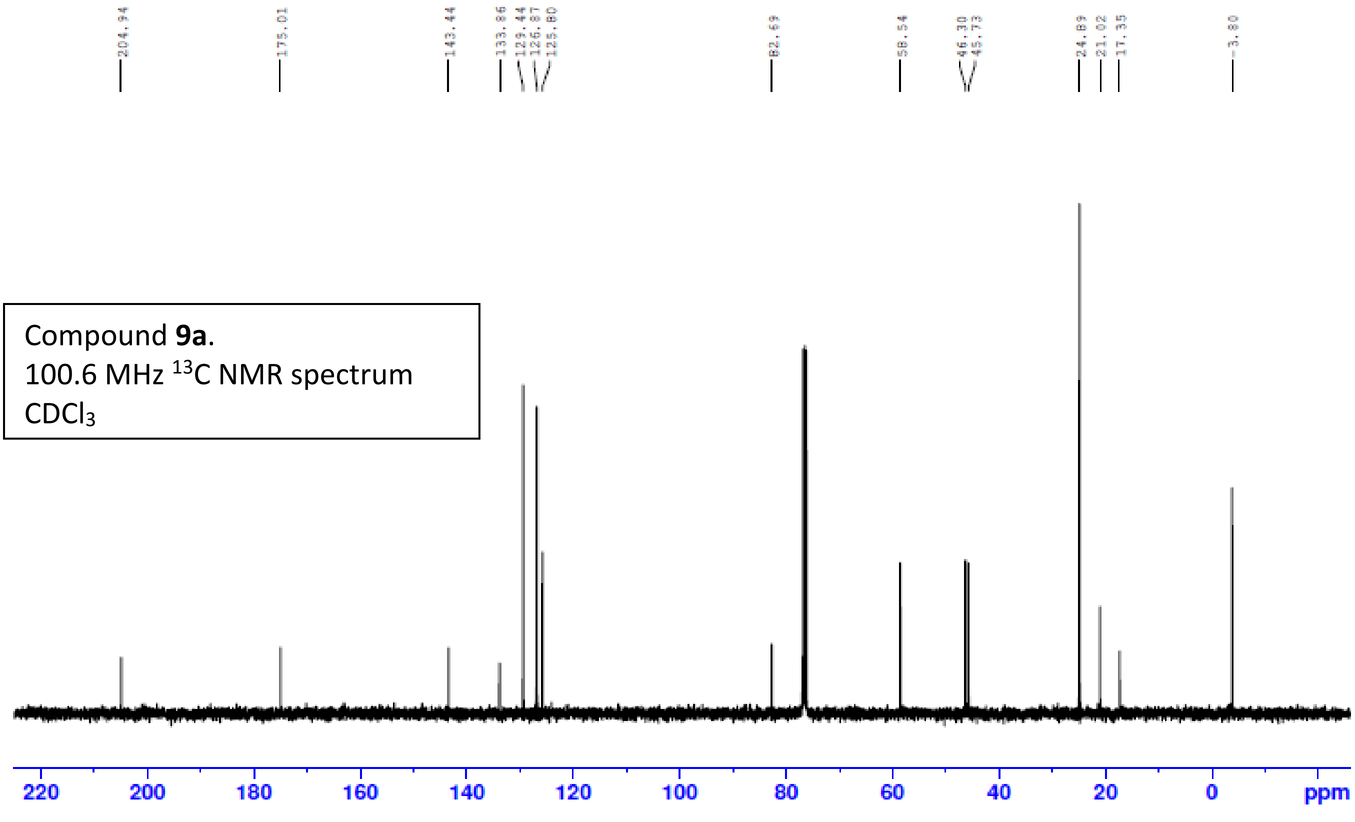

Compound **9a**.
100.6 MHz ^{13}C NMR spectrum
$CDCl_3$

Compound **9b**.
400.13 MHz ^{1}H NMR spectrum
$CDCl_3$

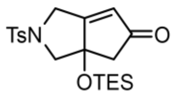

9b

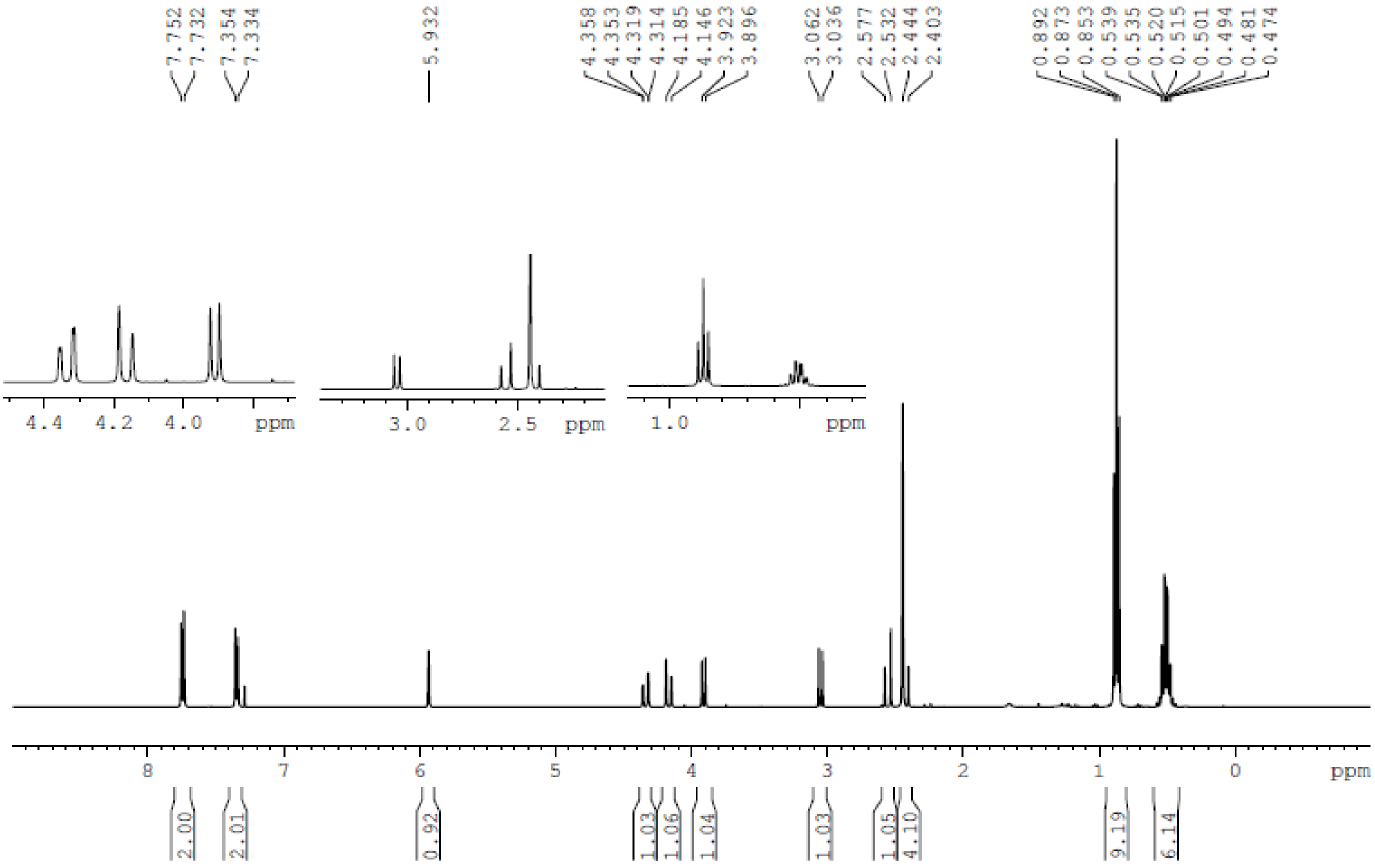

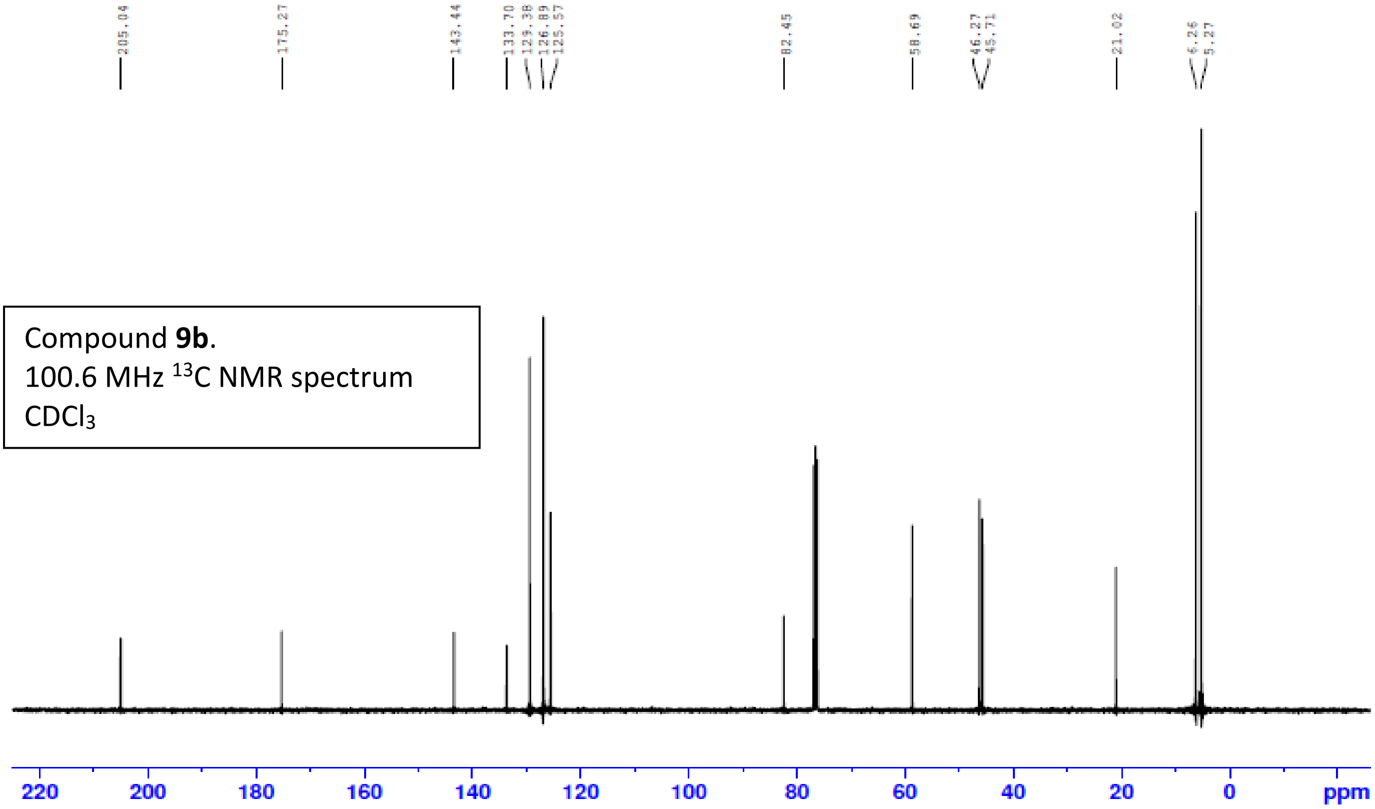

Compound **9b**.
100.6 MHz ^{13}C NMR spectrum
$CDCl_3$

Compound **9c**.
400.13 MHz ^{1}H NMR spectrum
$CDCl_3$

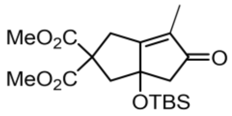

9c

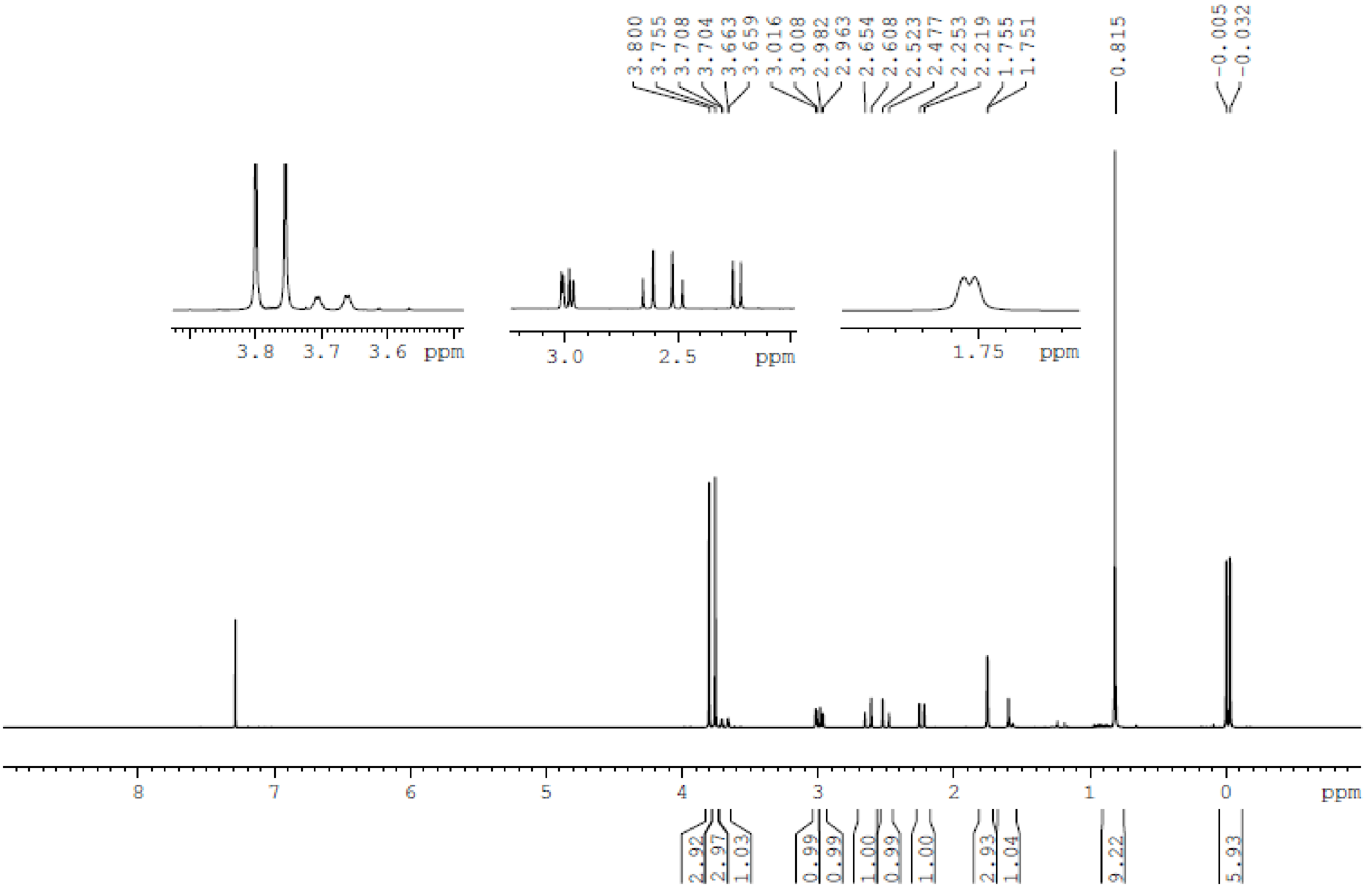

Compound **9c**.
100.6 MHz ^{13}C NMR spectrum
$CDCl_3$

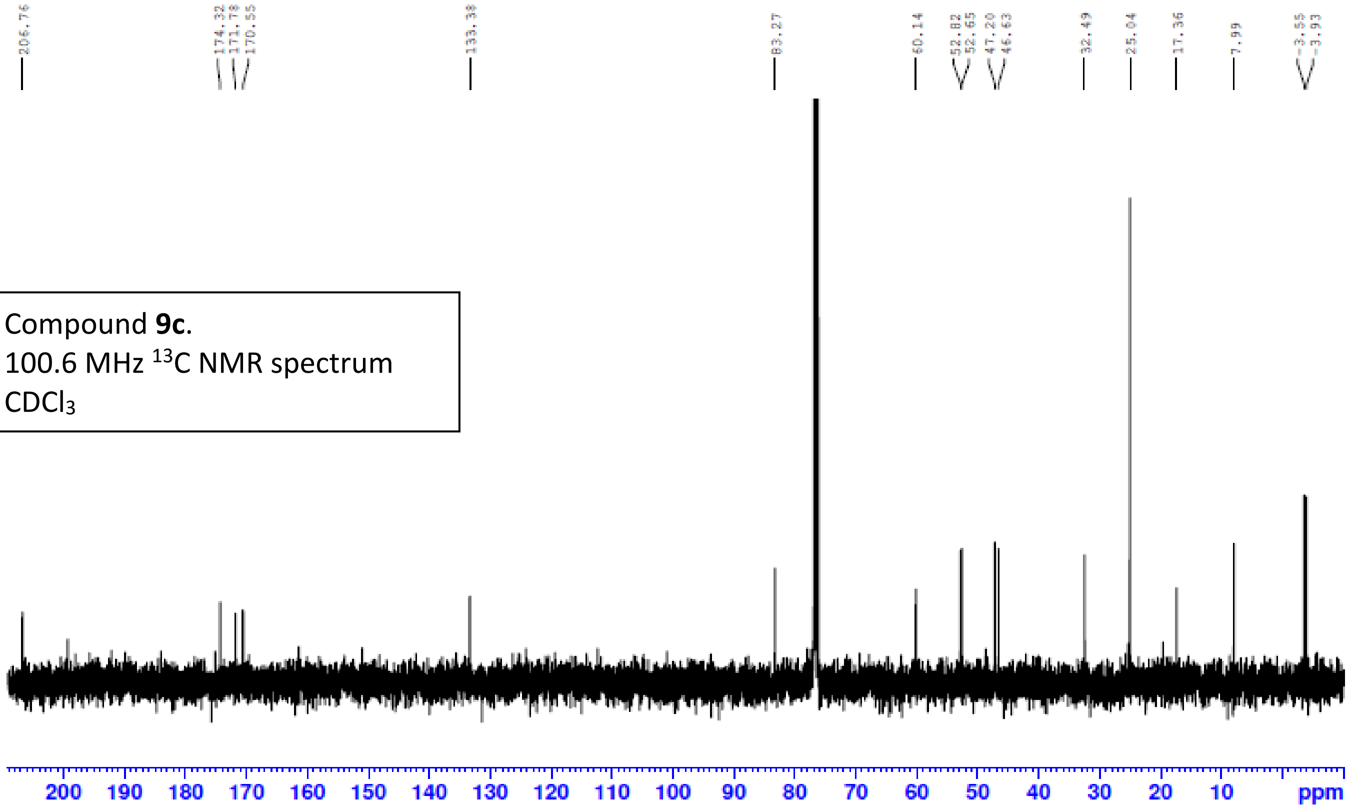

Compound **9d**.
400.13 MHz ^{1}H NMR spectrum
$CDCl_3$

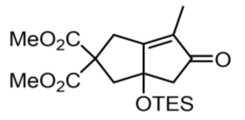

9d

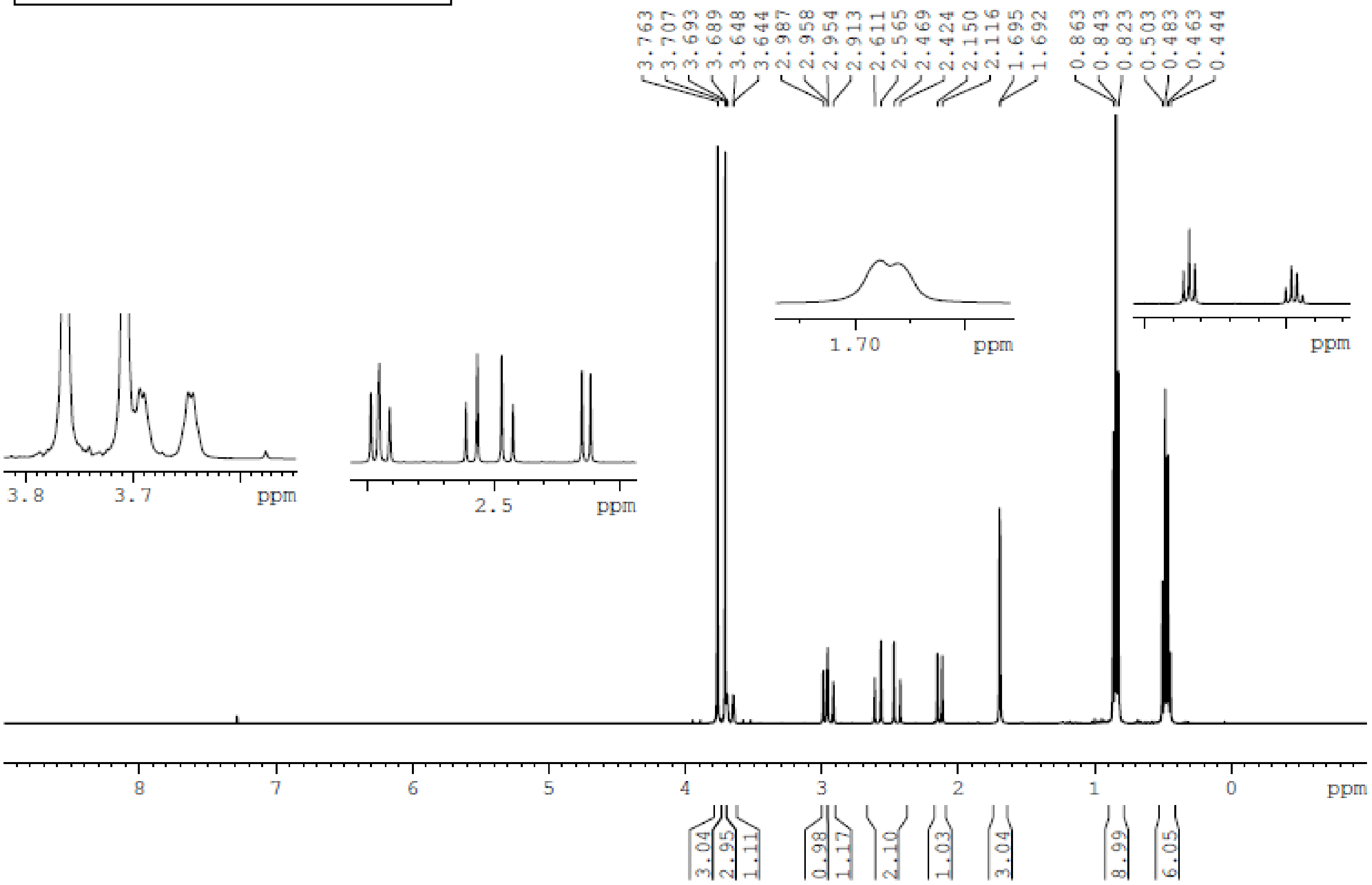

Compound **9d**.
100.6 MHz ^{13}C NMR spectrum
$CDCl_3$

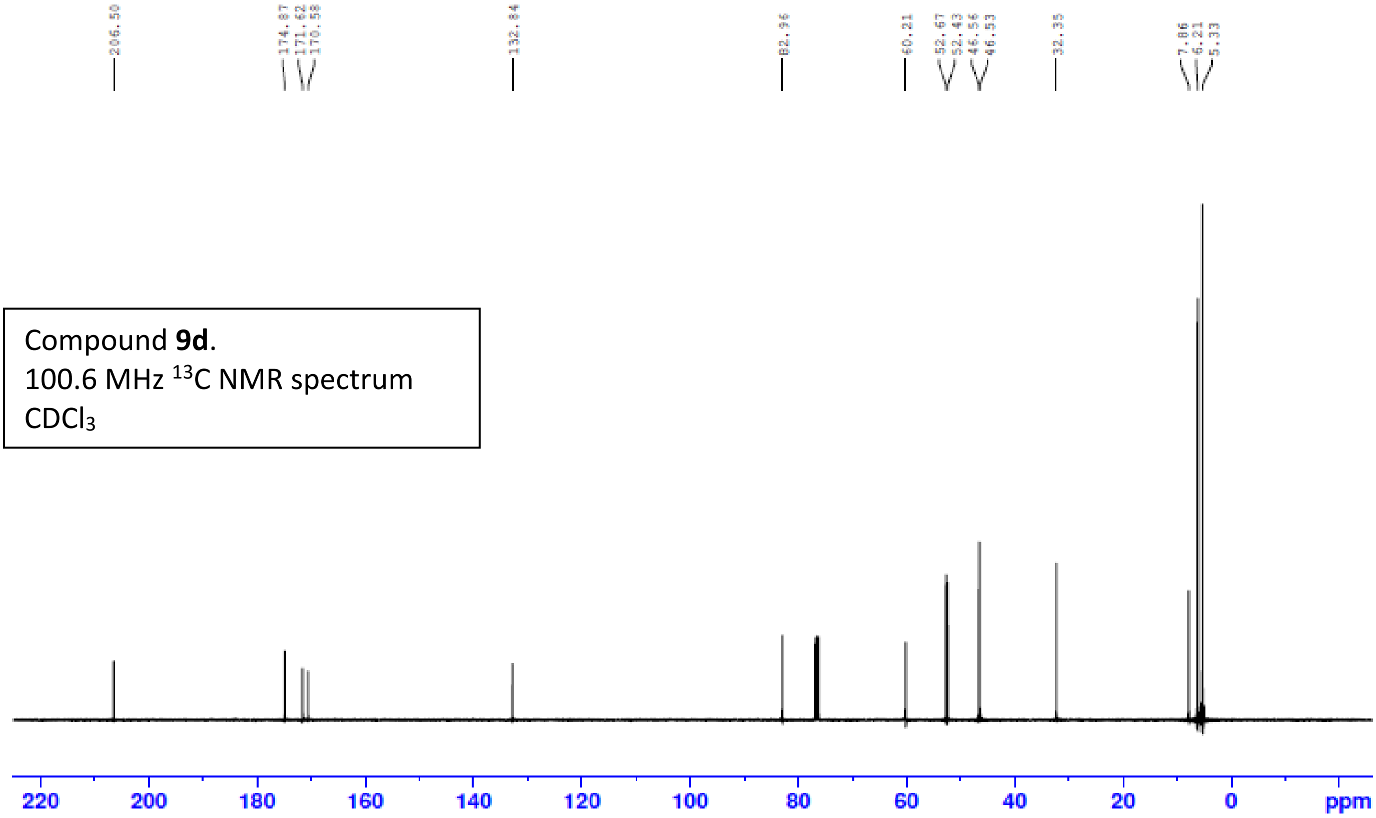

Compound **9e**.
400.13 MHz ^{1}H NMR spectrum
$CDCl_3$

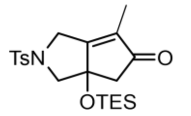

9e

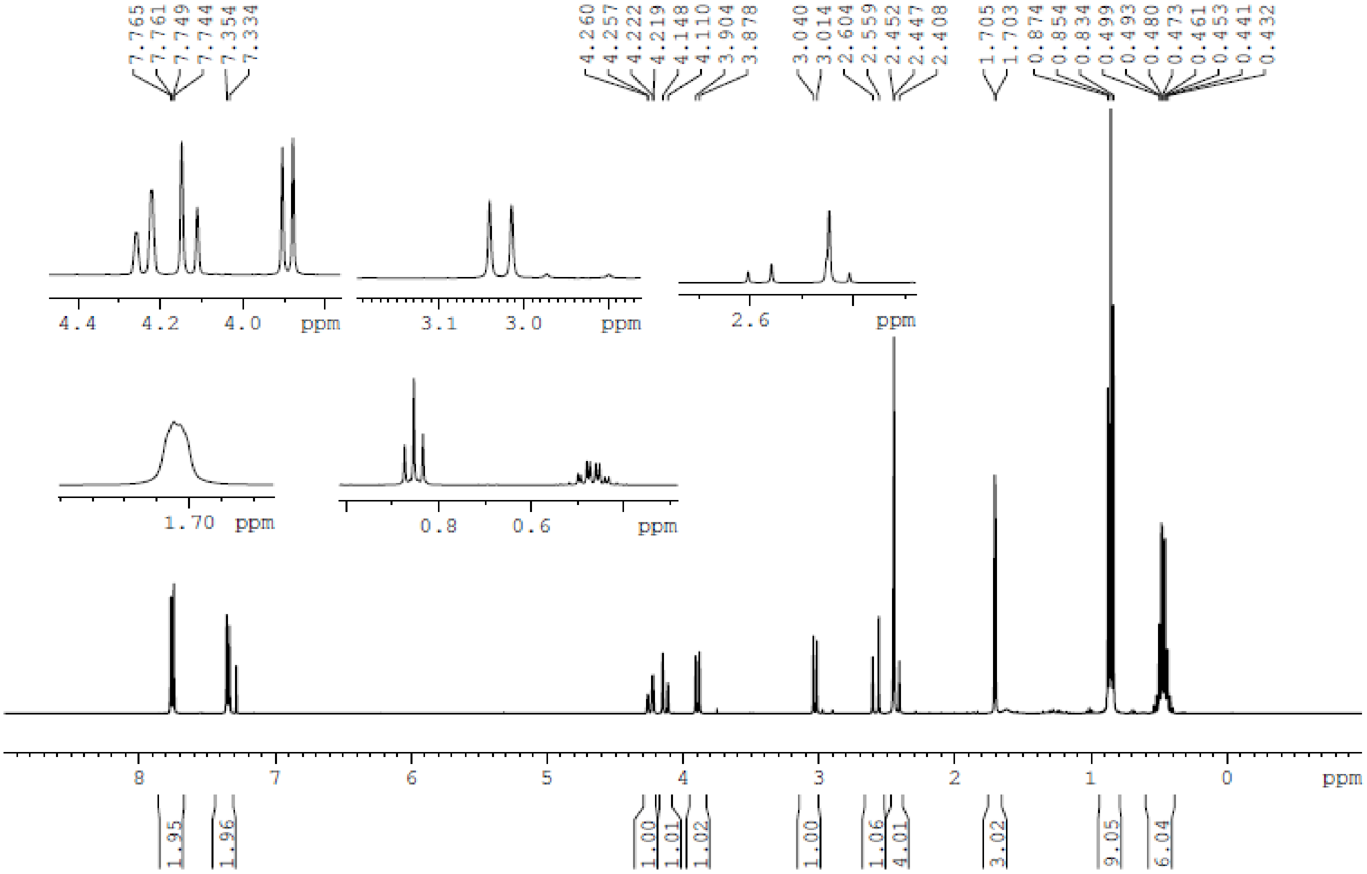

Compound **9e**.
100.6 MHz ^{13}C NMR spectrum
$CDCl_3$

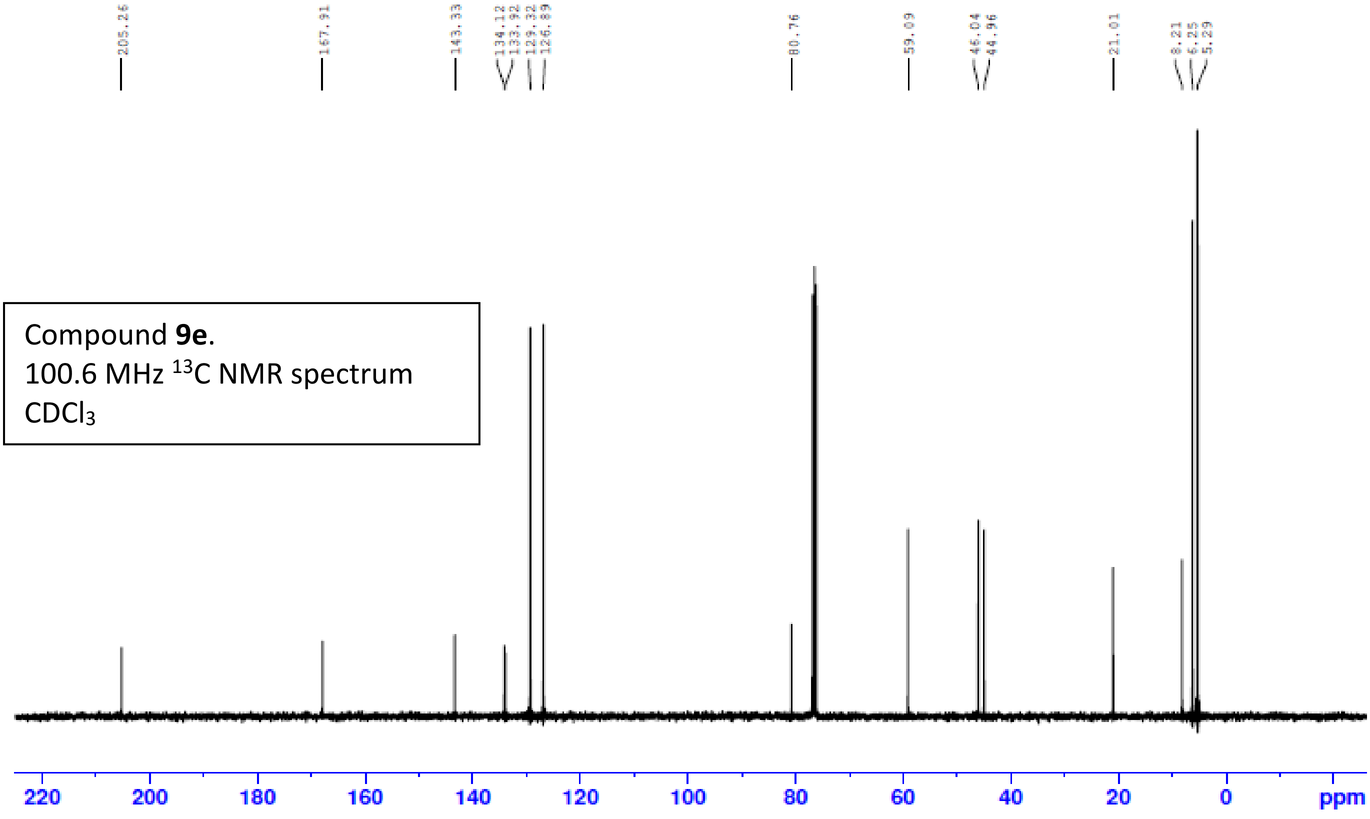

Compound **9f**.
400.13 MHz ^{1}H NMR spectrum
$CDCl_3$

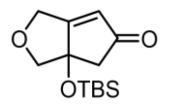

9f

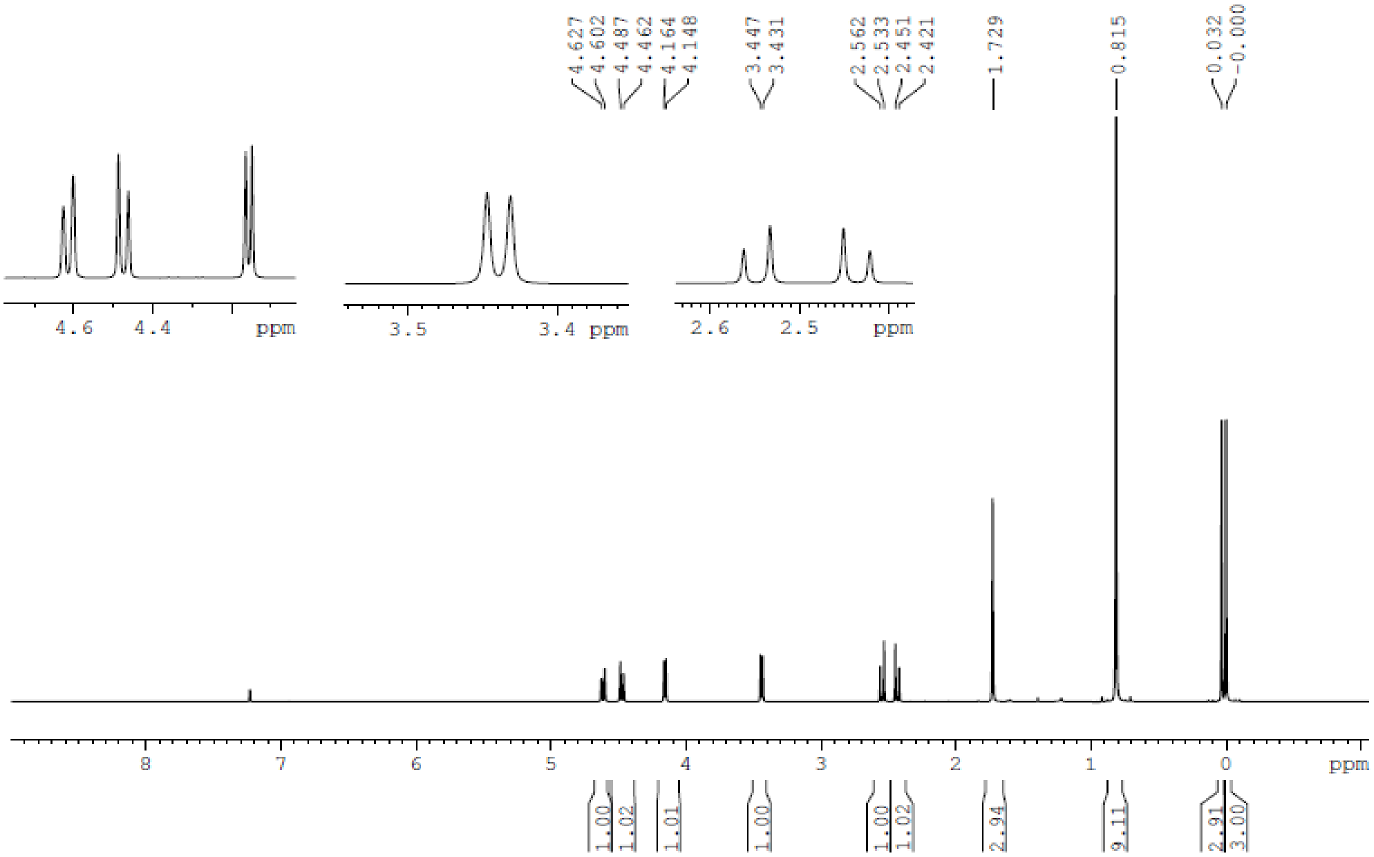

Compound **9f**.
100.6 MHz ^{13}C NMR spectrum
$CDCl_3$

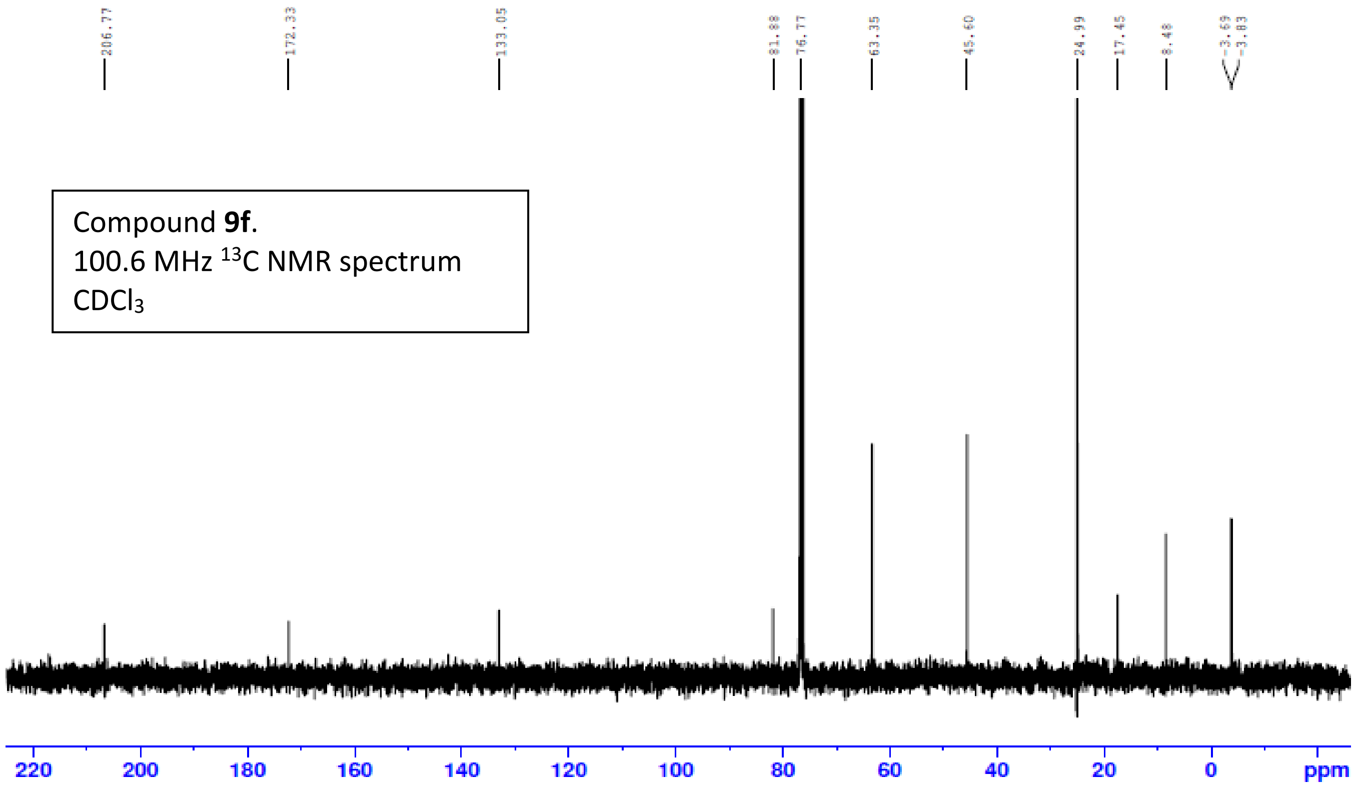

Compound **9g**.
400.13 MHz ^{1}H NMR spectrum

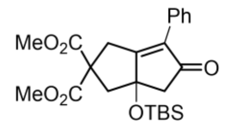

9g

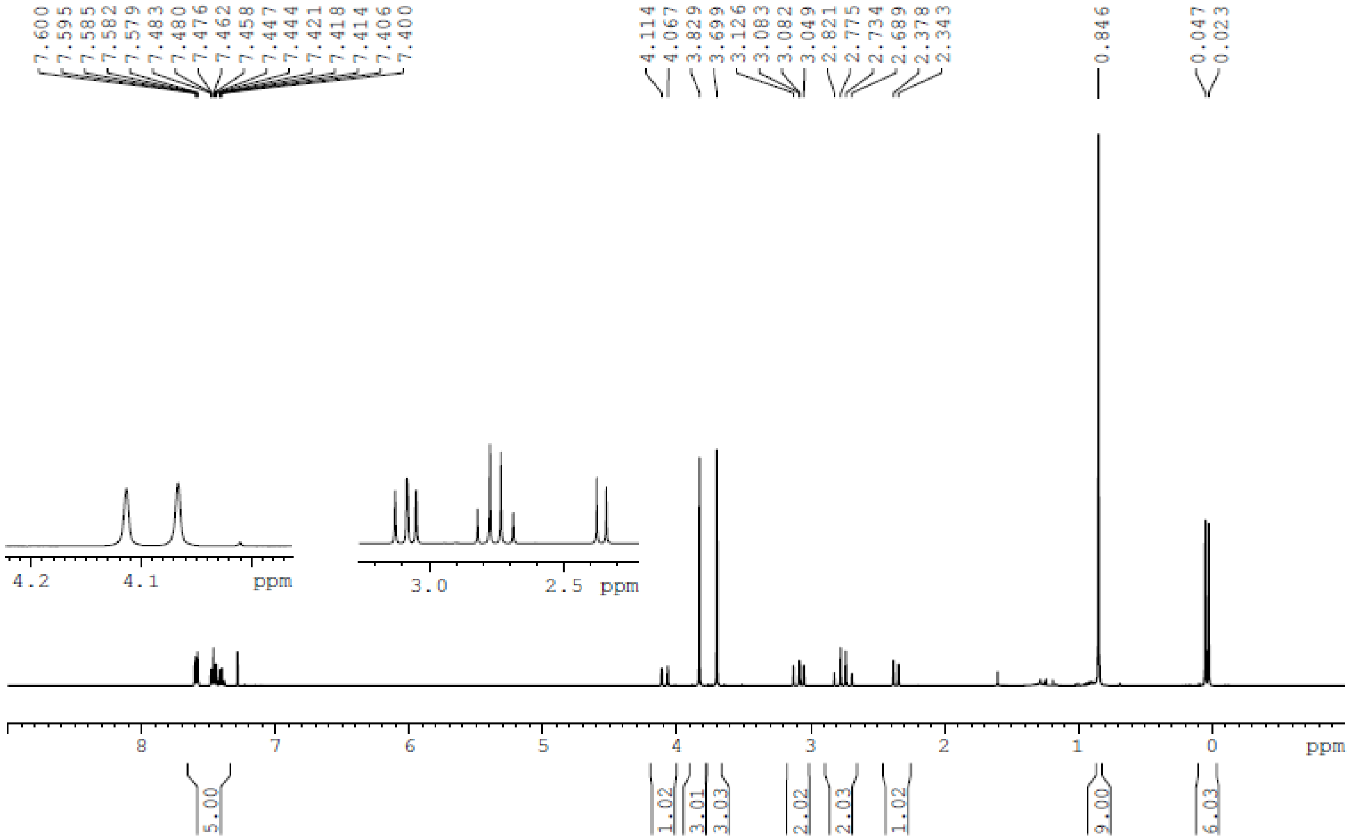

Compound **9g**.
400.13 MHz ^{1}H NMR spectrum

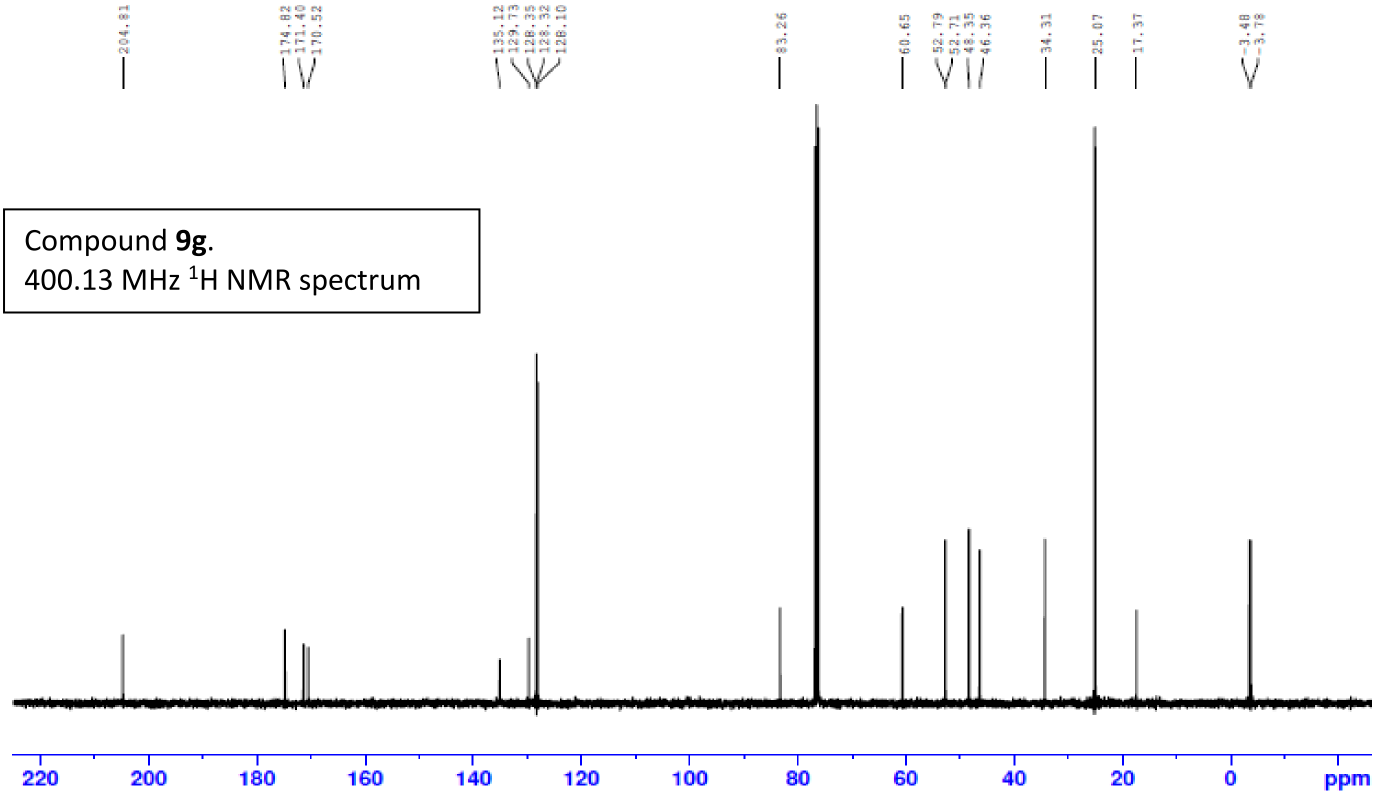

Compound **9h**.
400.13 MHz ^{1}H NMR spectrum

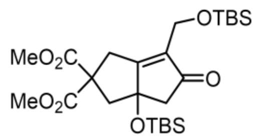

9h

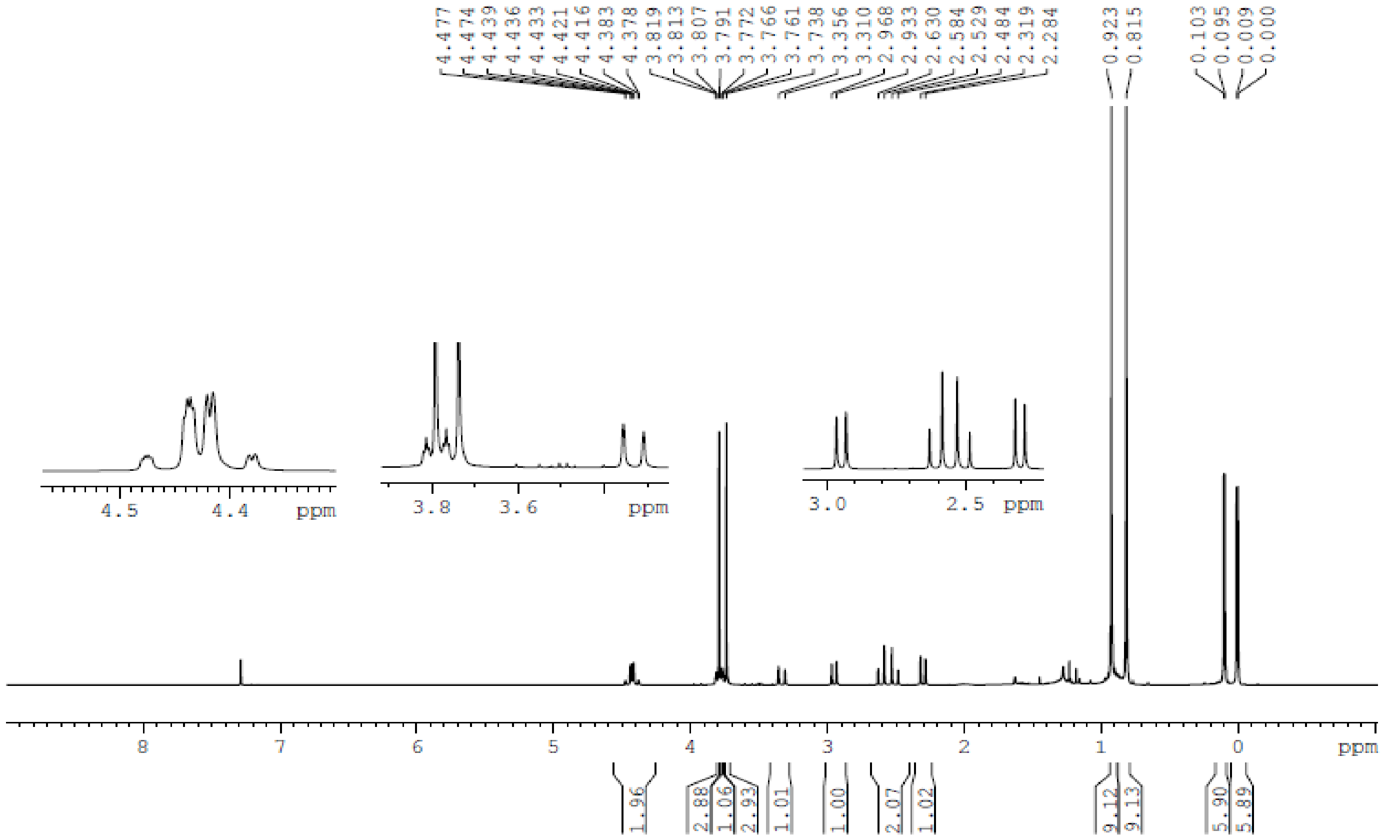

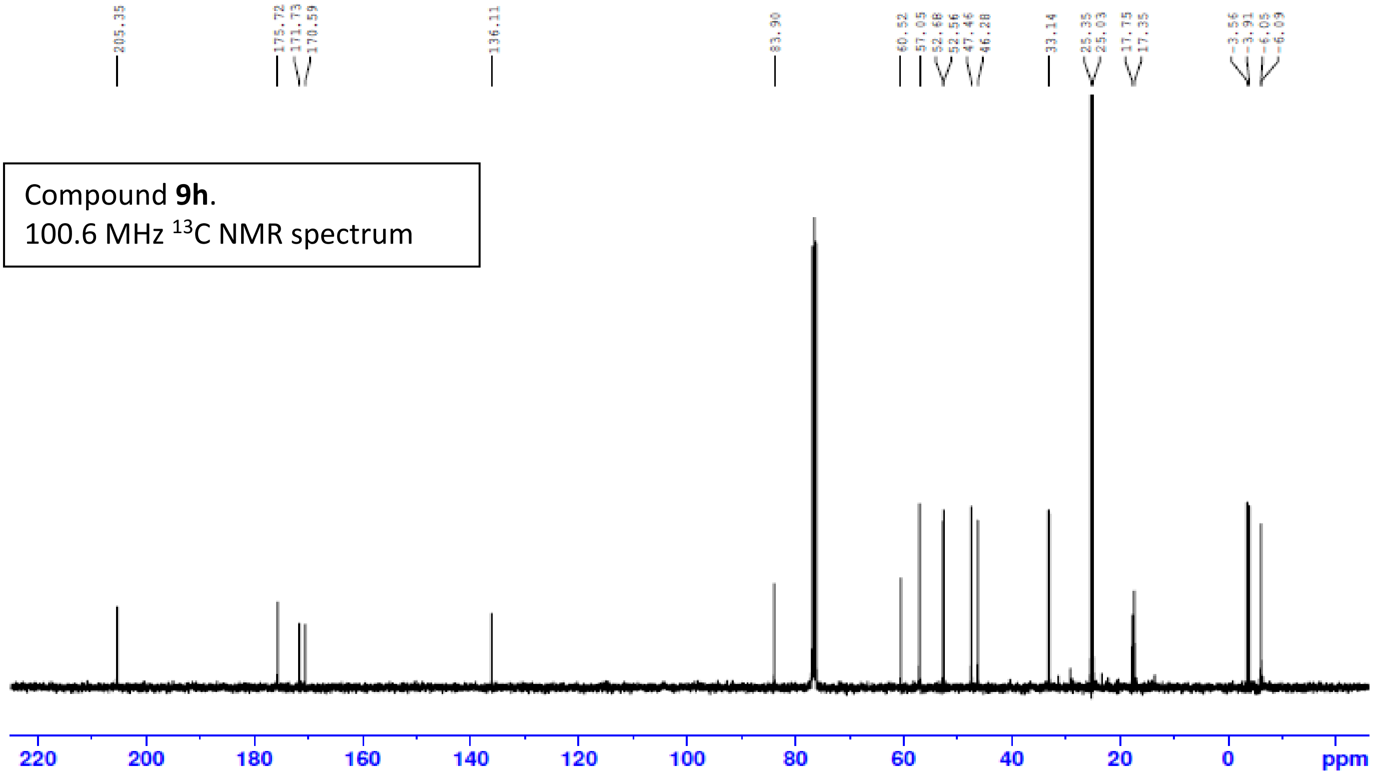

Compound **9h**.
100.6 MHz ^{13}C NMR spectrum

Compound **9i**.
400.13 MHz ^{1}H NMR spectrum
$CDCl_3$

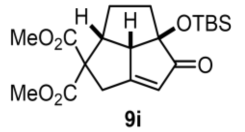

9i

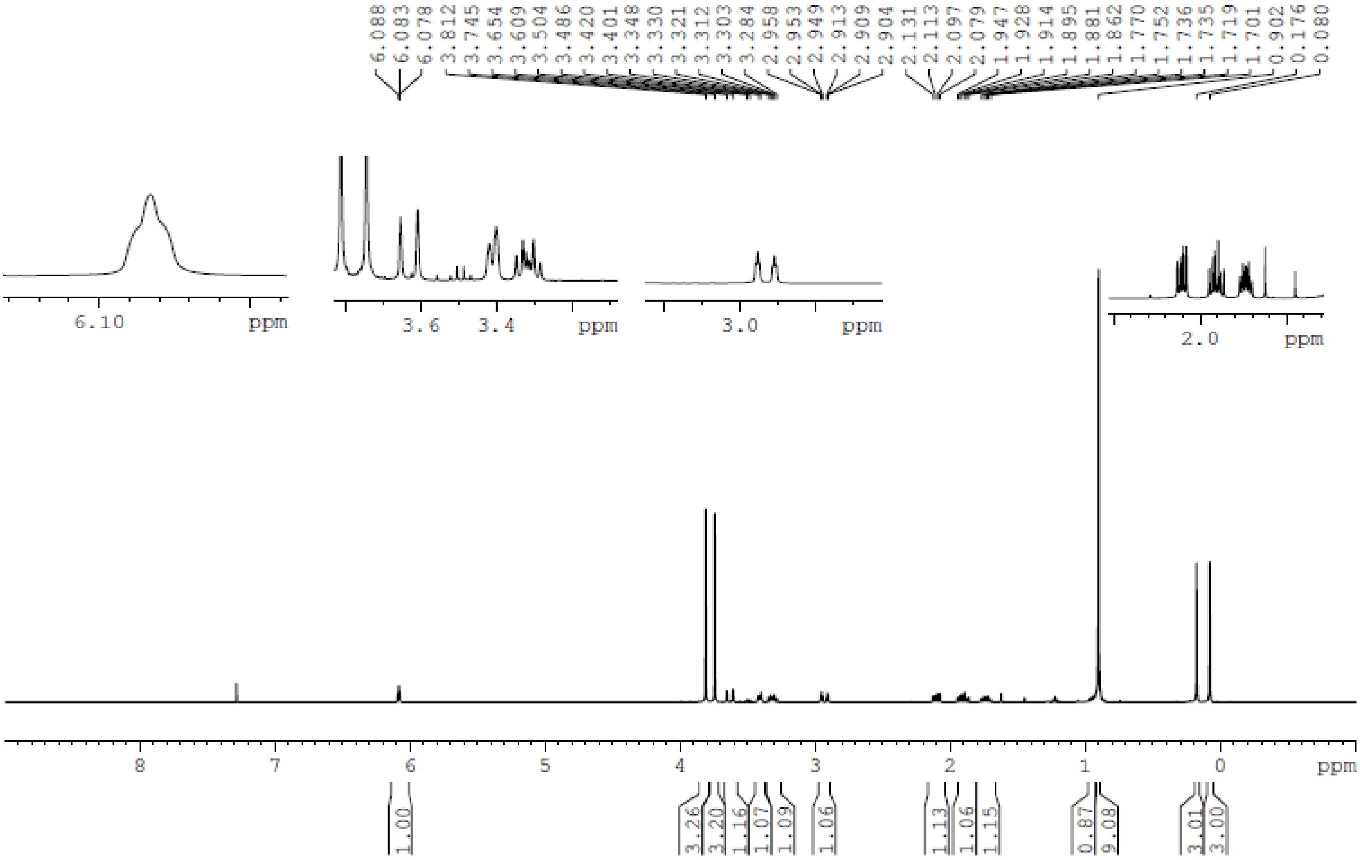

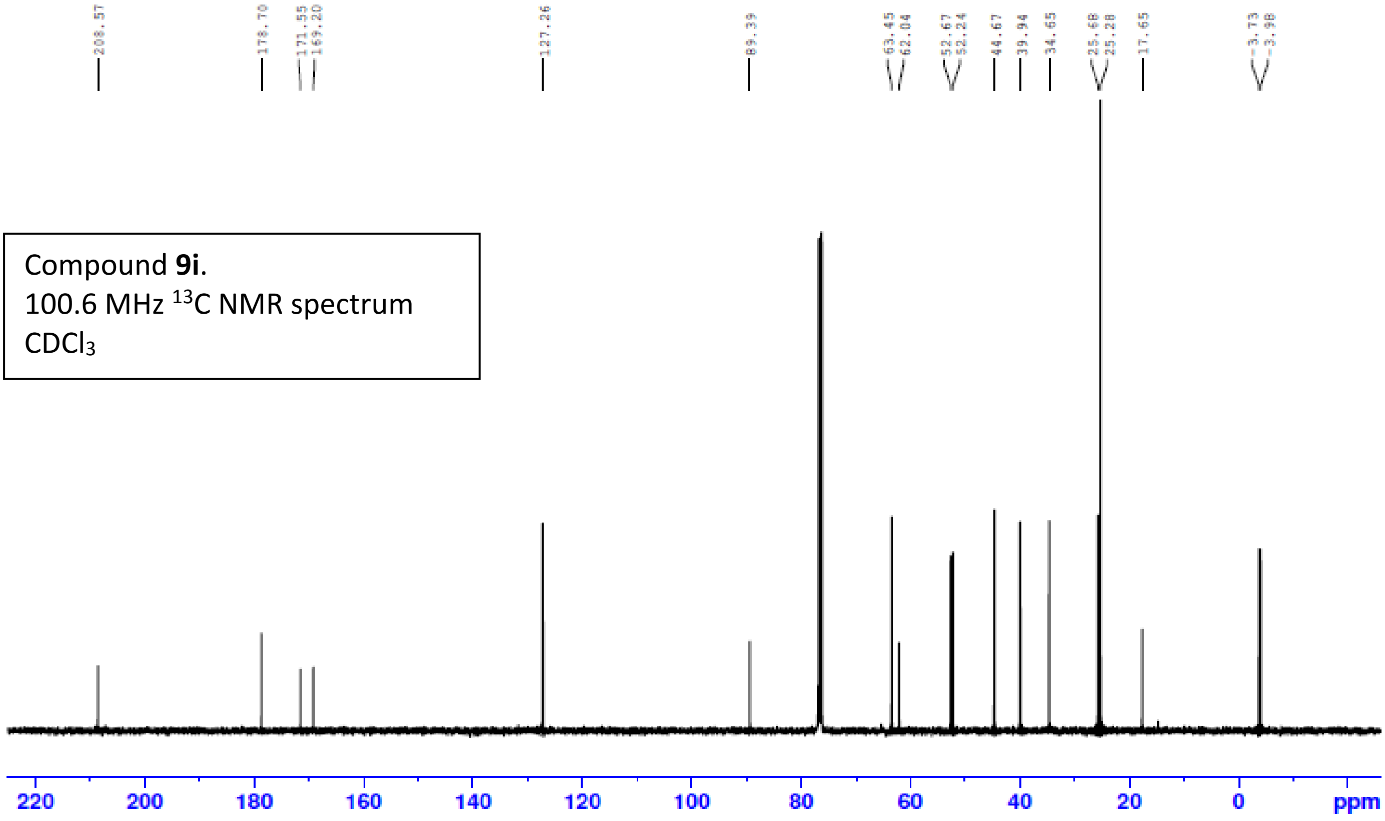

Compound **9i**.
100.6 MHz ^{13}C NMR spectrum
$CDCl_3$

Compound **9j**.
400.13 MHz ^{1}H NMR spectrum
$CDCl_3$

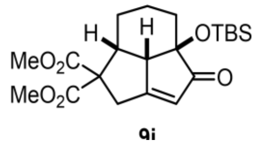

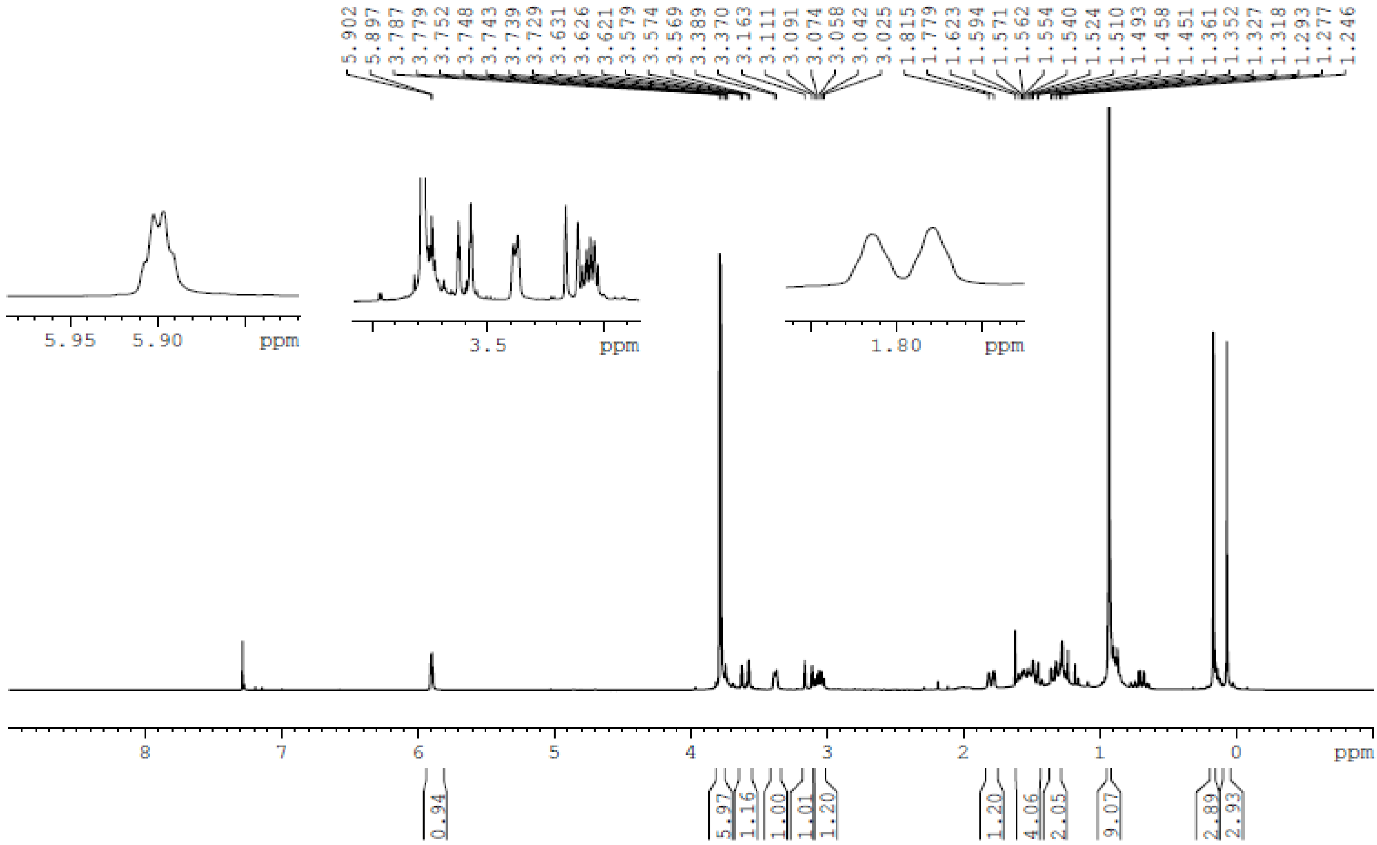

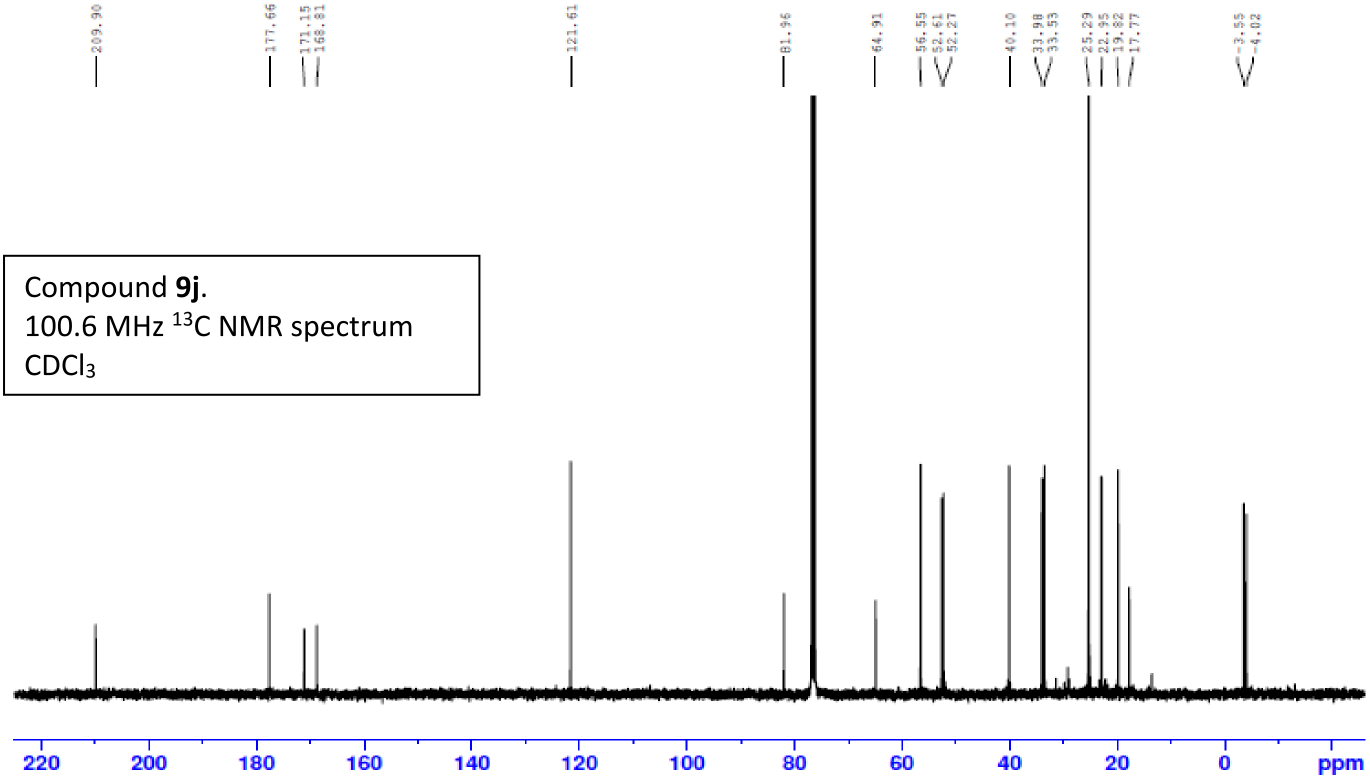

Compound **9j**.
100.6 MHz ^{13}C NMR spectrum
$CDCl_3$

Compound ***anti*-11a**.
400.13 MHz ^{1}H NMR spectrum
$CDCl_3$

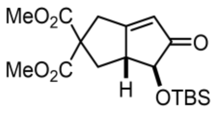

***anti*-11a**

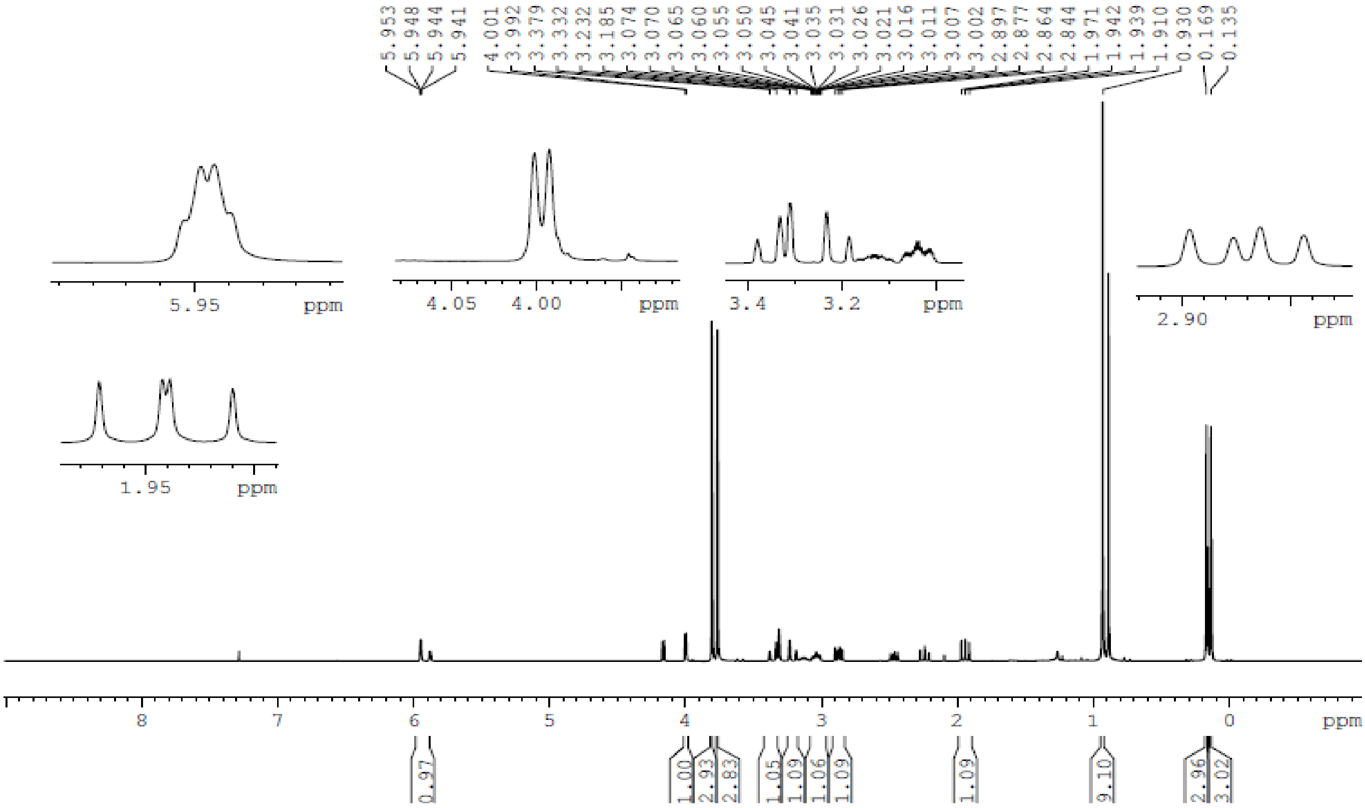

Compound ***syn*-11a**.
400.13 MHz ^{1}H NMR spectrum
$CDCl_3$

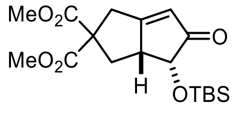

***syn*-11a**

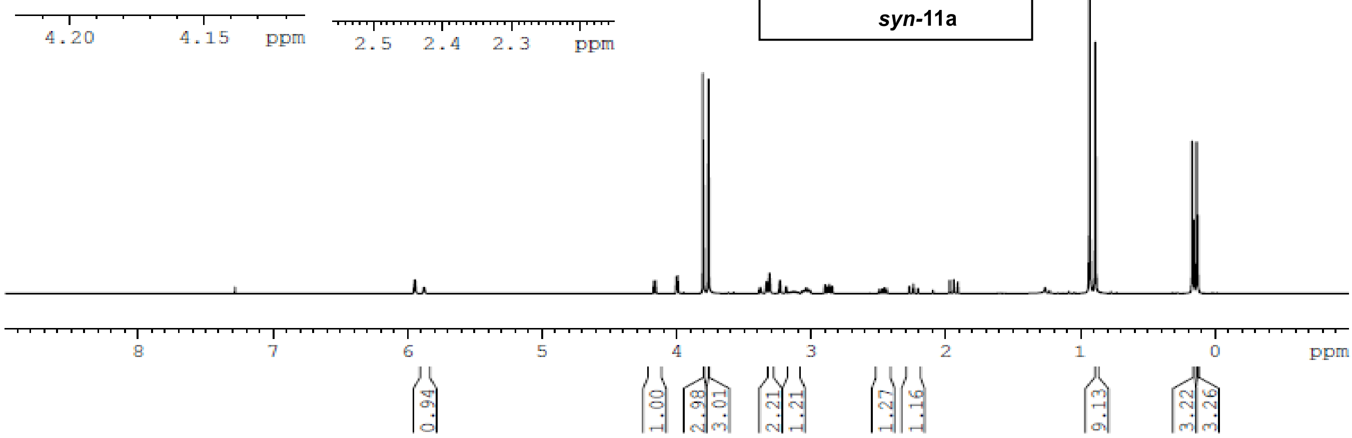

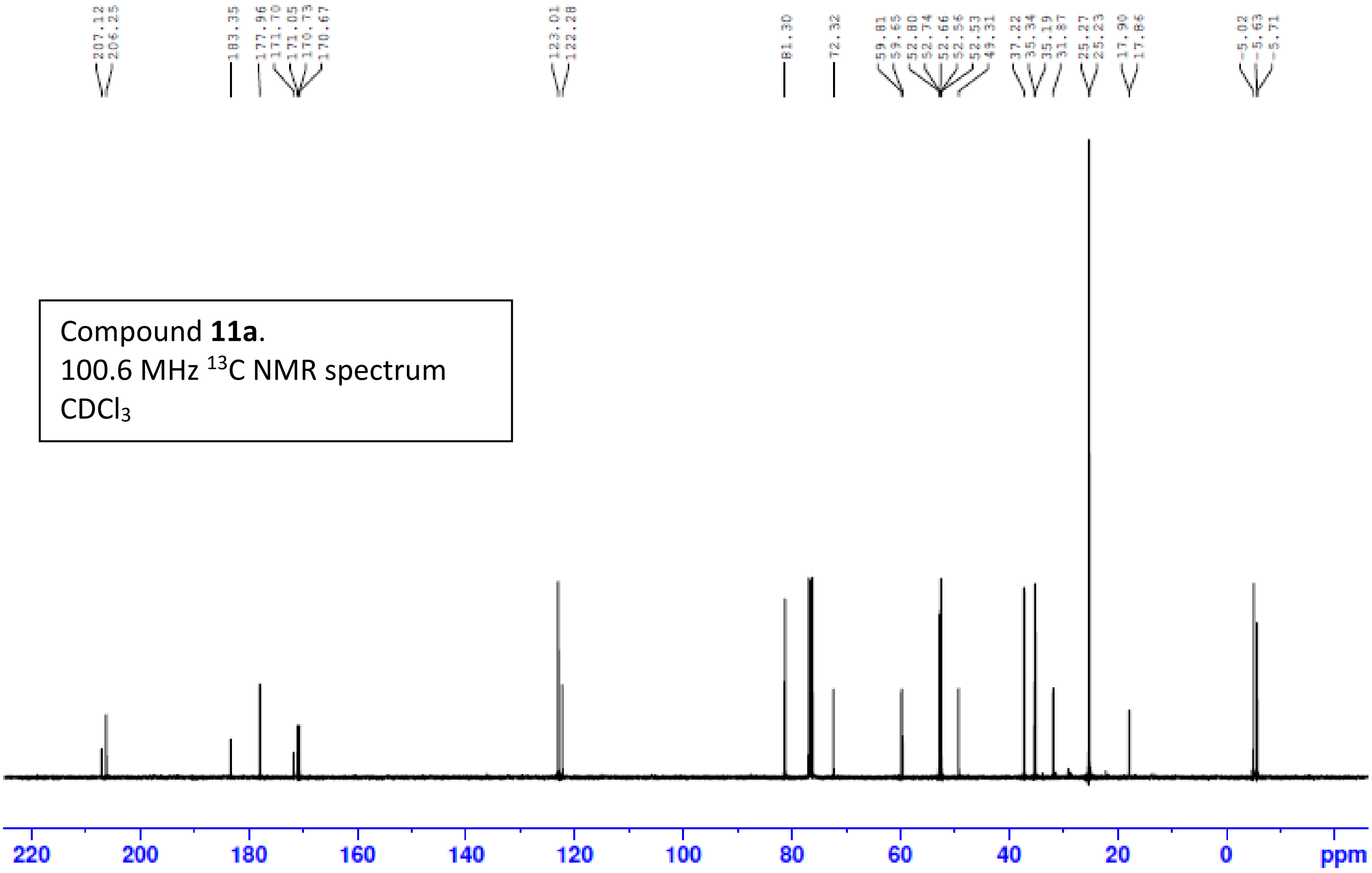
207.12
206.25
183.35
177.96
171.70
171.05
170.73
170.67
123.01
122.28
81.30
72.32
59.81
59.65
52.80
52.74
52.66
52.56
52.53
49.31
37.22
35.34
35.19
31.87
25.27
25.23
17.90
17.86
-5.02
-5.63
-5.71
Compound **11a**.
100.6 MHz ^{13}C NMR spectrum
$CDCl_3$
220
200
180
160
140
120
100
80
60
40
20
0
ppm

Compound ***anti*-11b**.
400.13 MHz ^{1}H NMR spectrum
$CDCl_3$

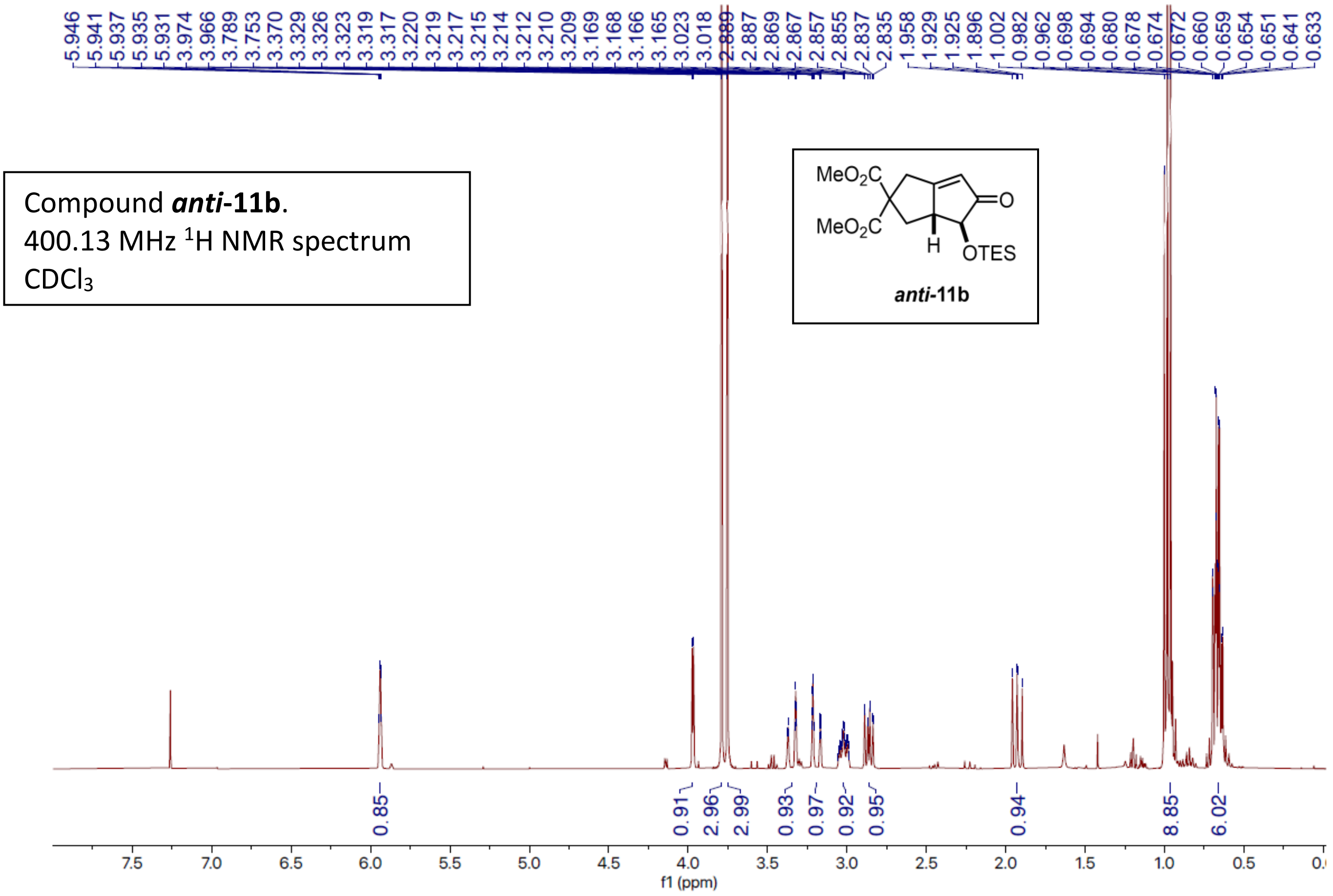

Compound ***anti*-11b**.
100.6 MHz ^{13}C NMR spectrum
$CDCl_3$

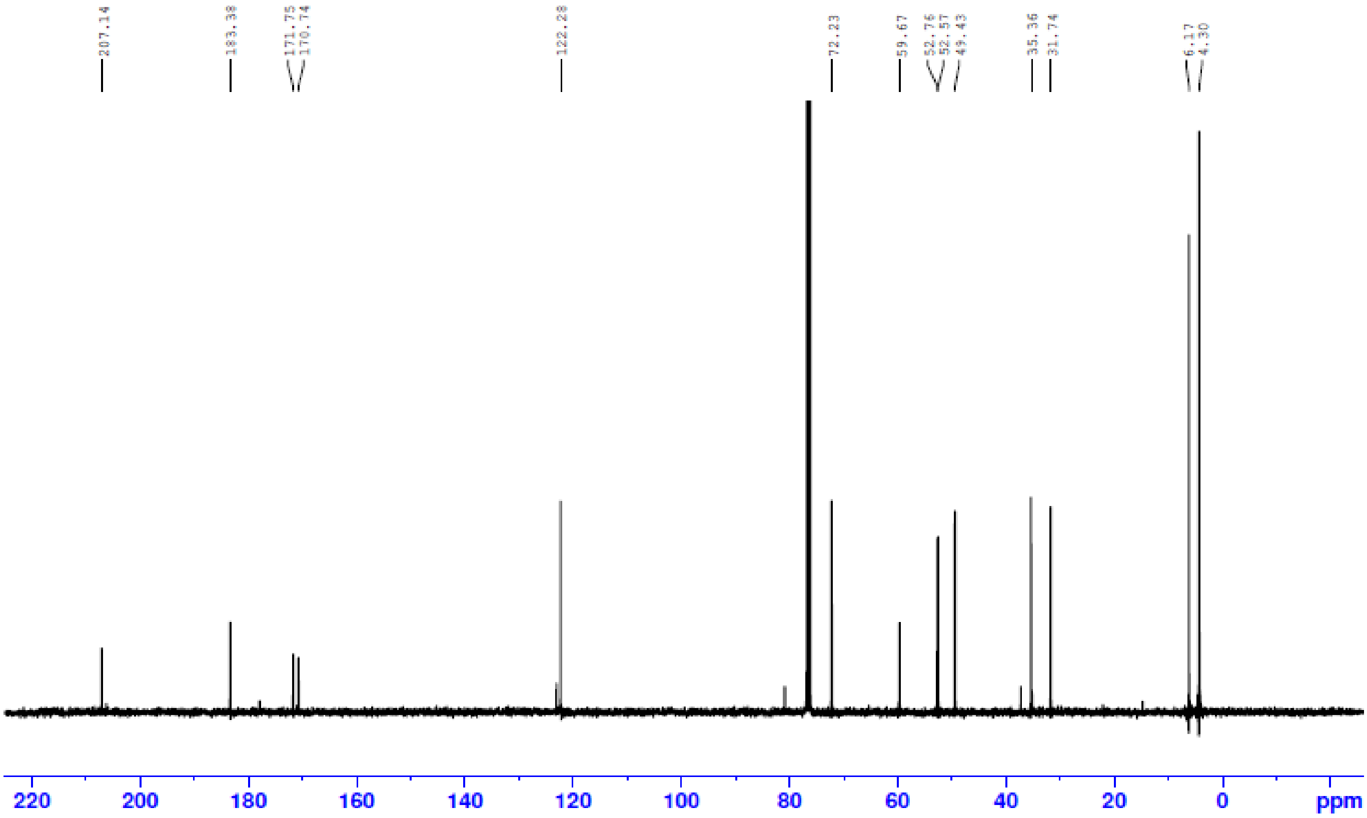

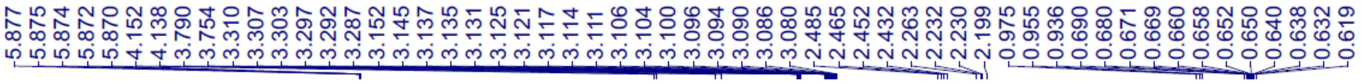

Compound ***syn*-11b**.
400.13 MHz ^{1}H NMR spectrum
$CDCl_3$

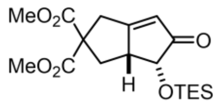

***syn*-11b**

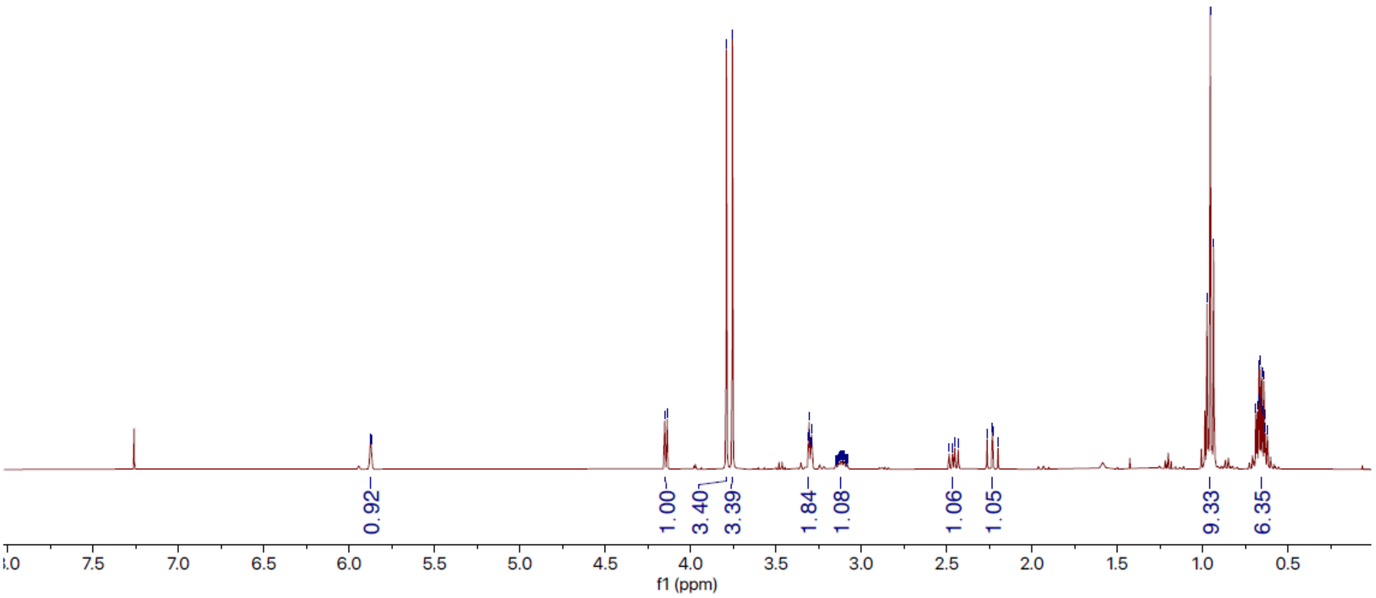

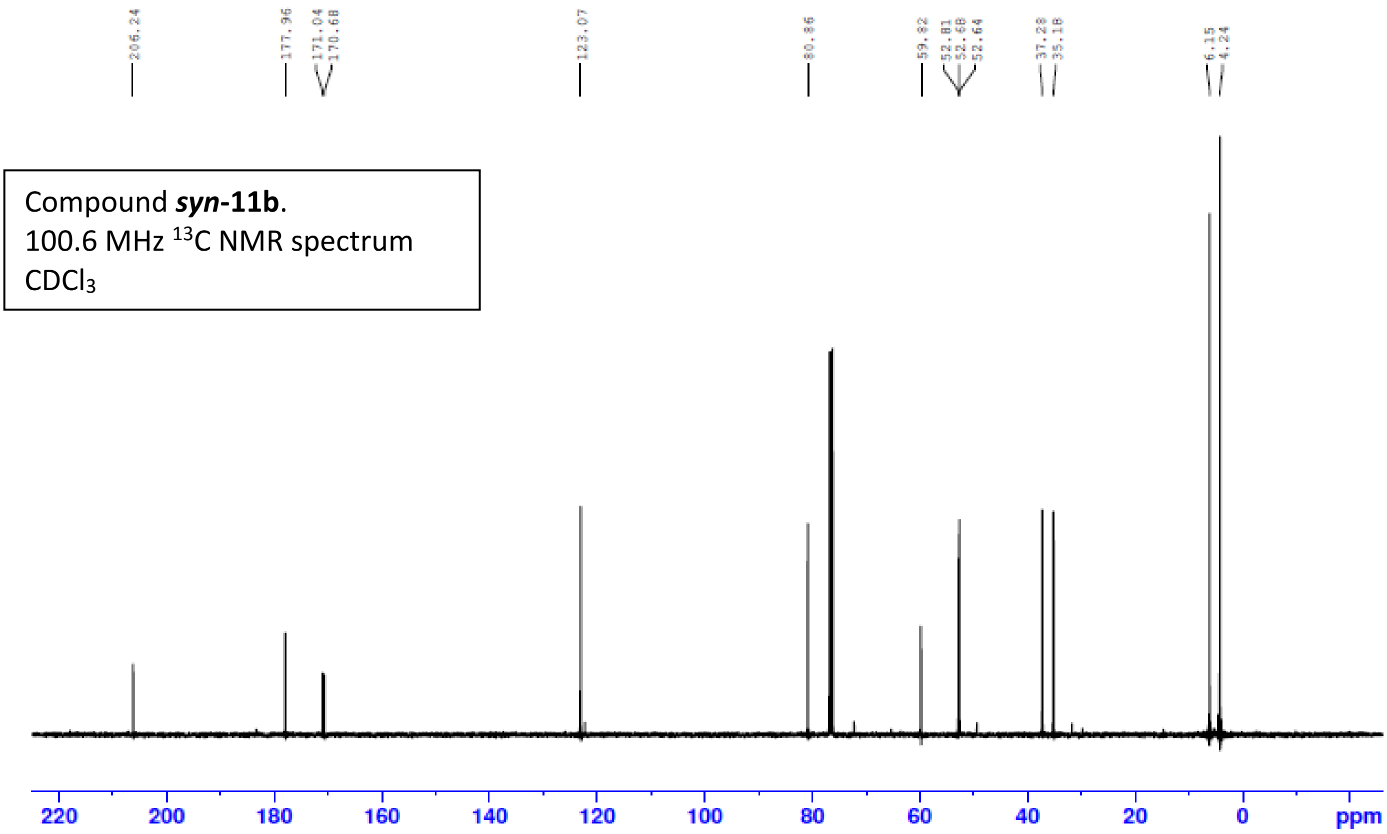

Compound ***syn*-11b**.
100.6 MHz ^{13}C NMR spectrum
$CDCl_3$

Compound ***anti*-11c**.
400.13 MHz ^{1}H NMR spectrum
$CDCl_3$

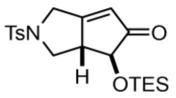

***anti*-11c**

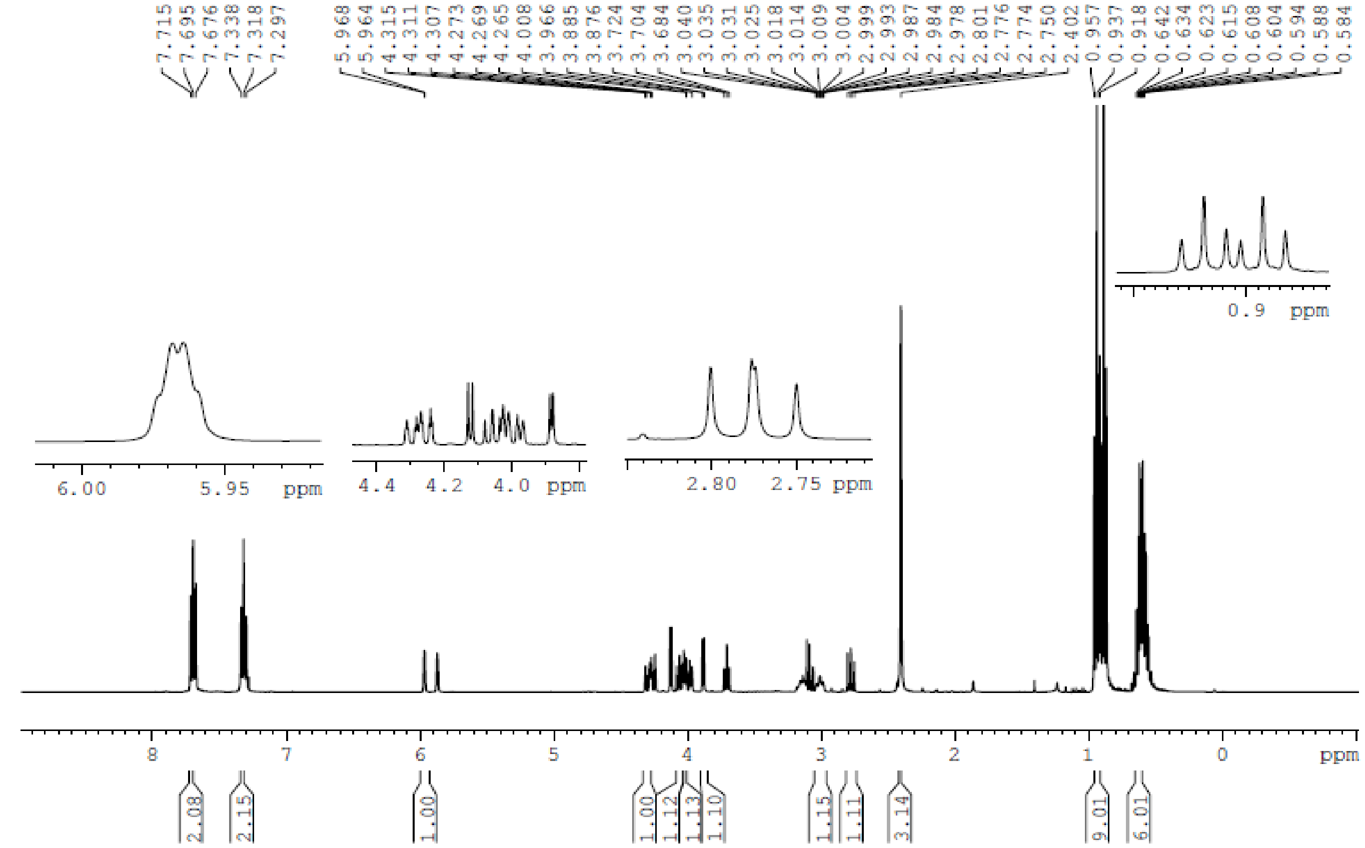

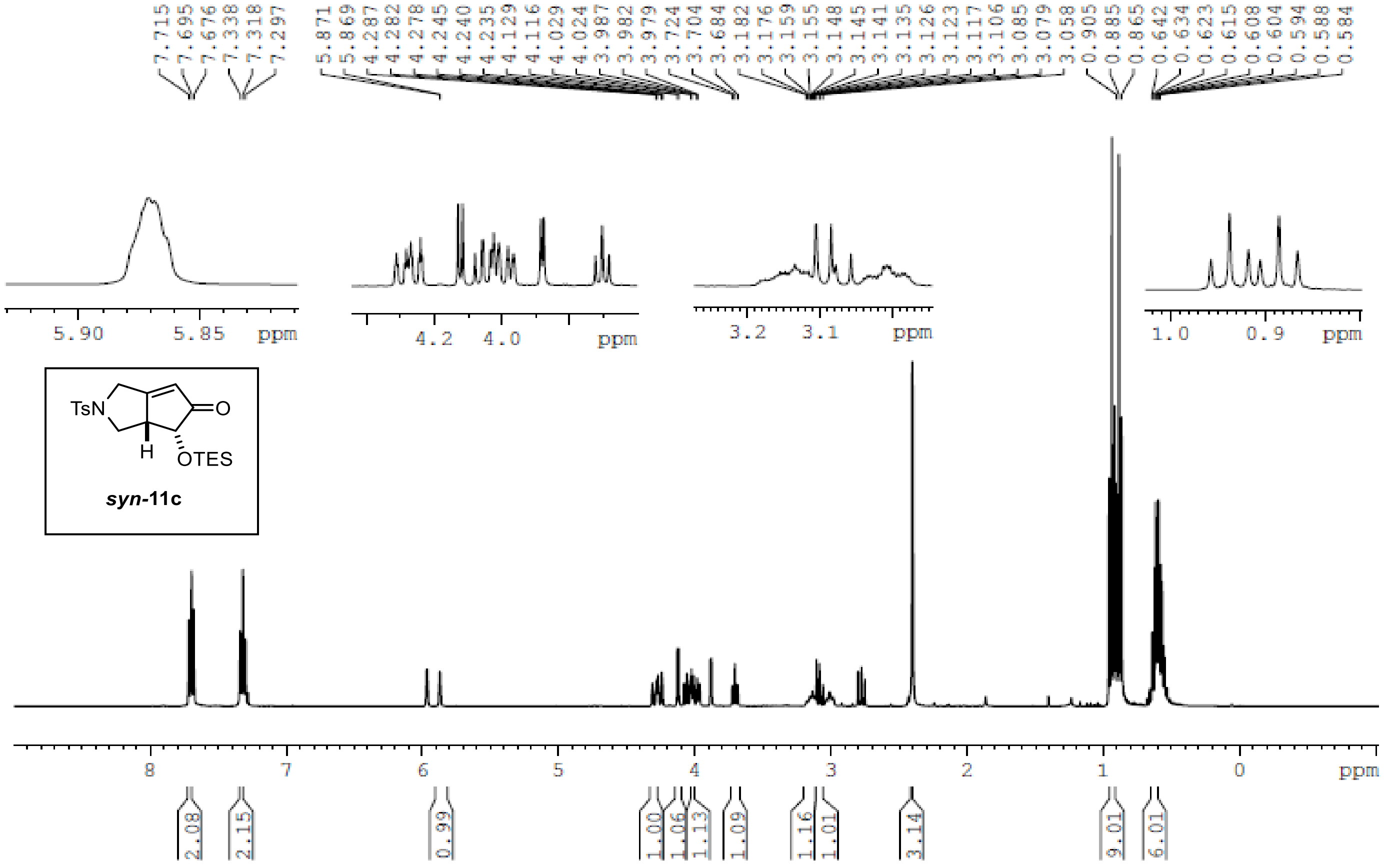

***syn*-11c**

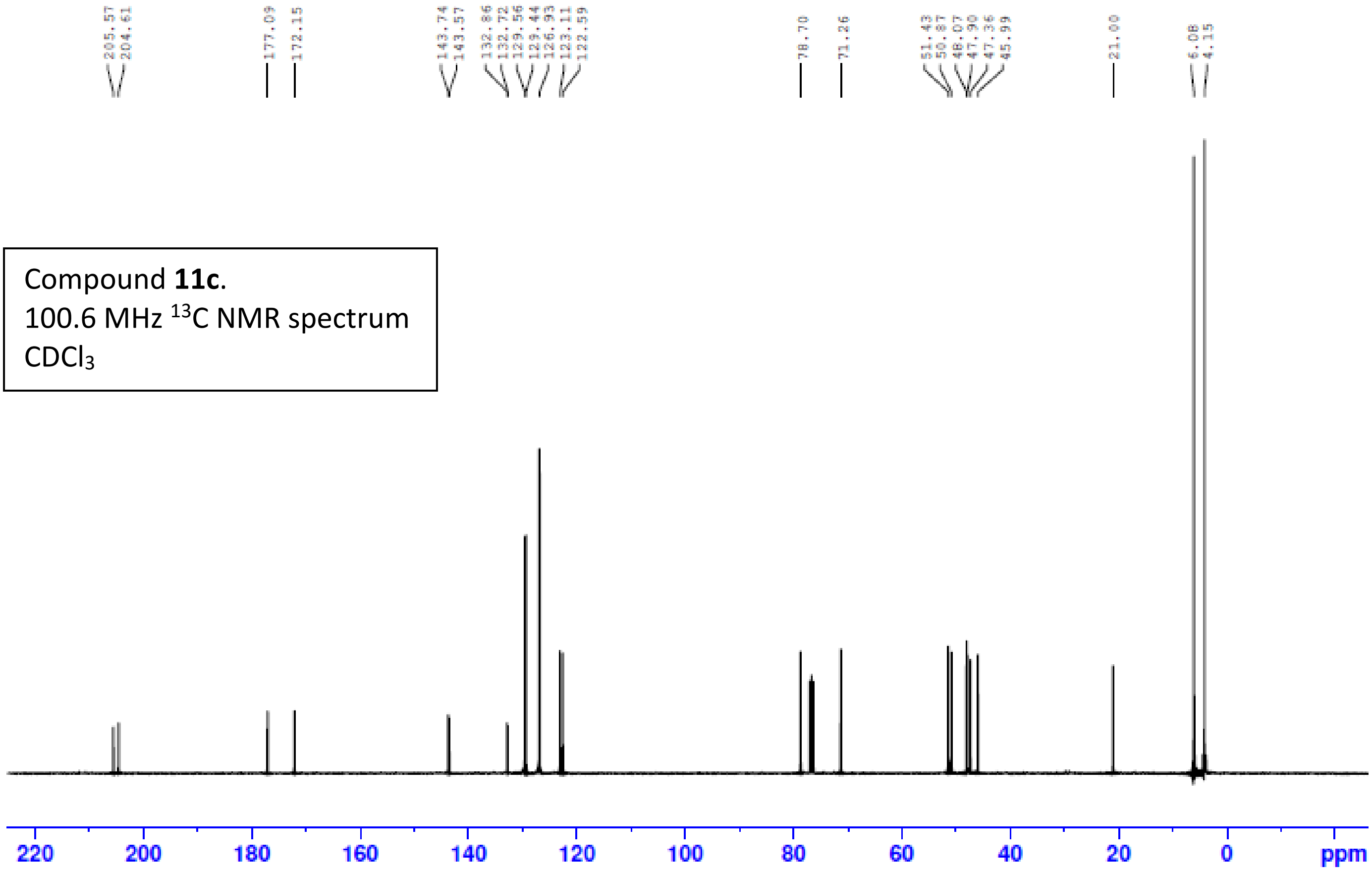
205.57
204.61
177.09
172.15
143.74
143.57
132.86
132.72
129.56
129.44
126.93
123.11
122.59
78.70
71.26
51.43
50.87
48.07
47.90
47.36
45.99
21.00
6.08
4.15
Compound **11c**.
100.6 MHz ^{13}C NMR spectrum
$CDCl_3$
220
200
180
160
140
120
100
80
60
40
20
0
ppm

Compound ***anti*-11d**.
400.13 MHz ^{1}H NMR spectrum
$CDCl_3$

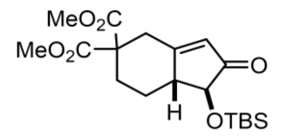

***anti*-11d**

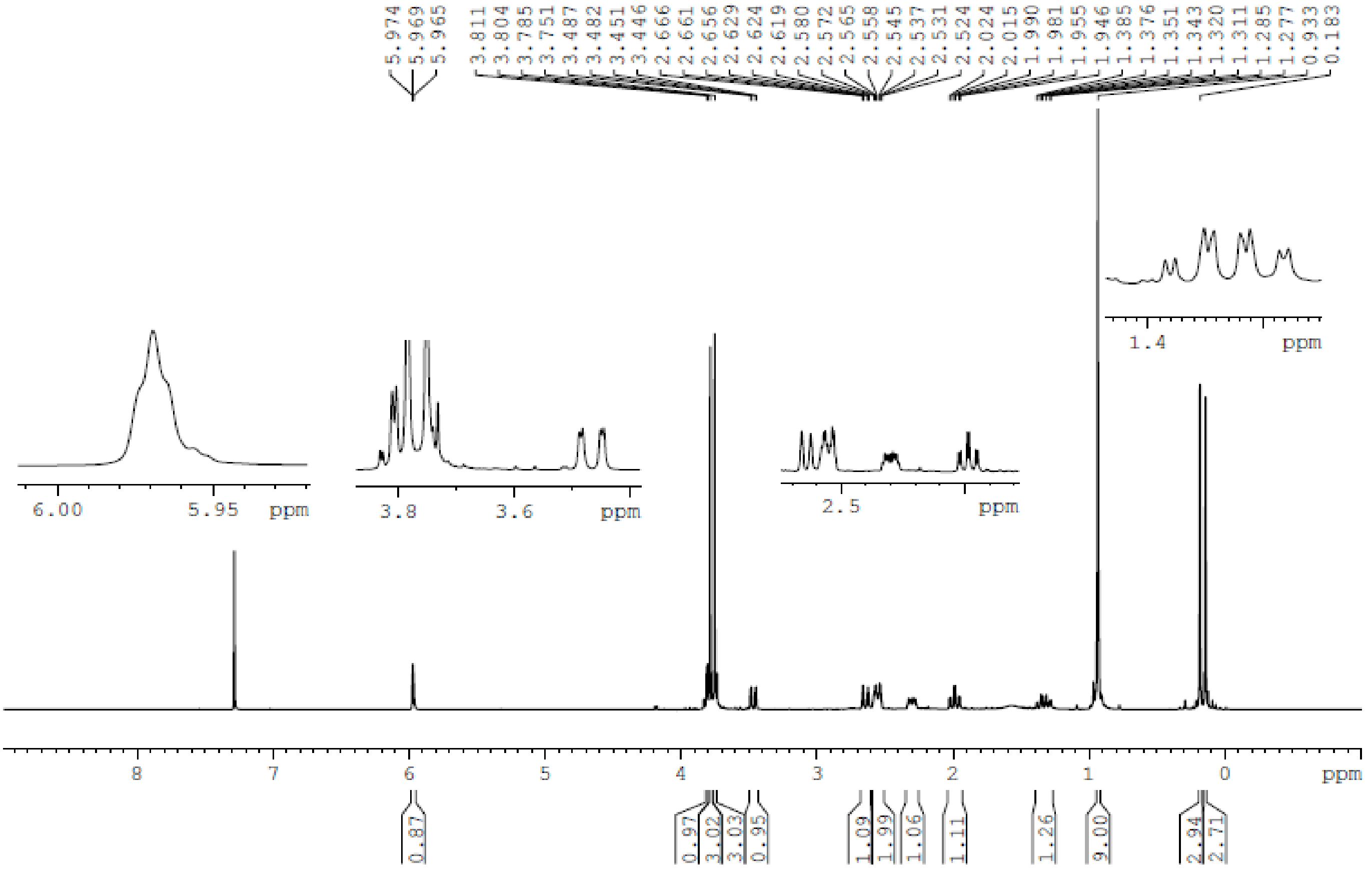

Compound **11d**.
100.6 MHz ^{13}C NMR spectrum
$CDCl_3$

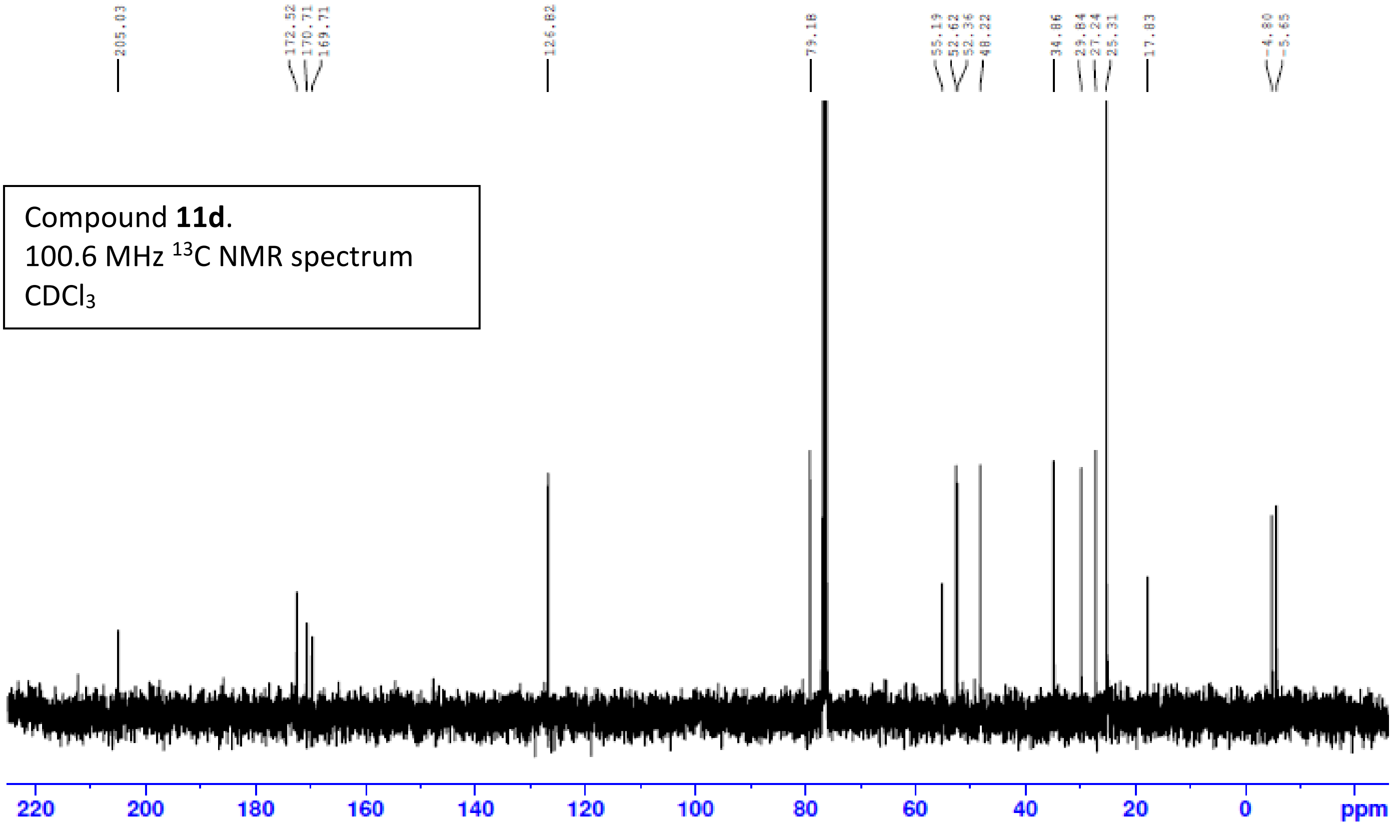

Compound **12a**.
400.13 MHz ^{1}H NMR spectrum
$CDCl_3$

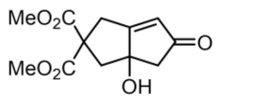

12a

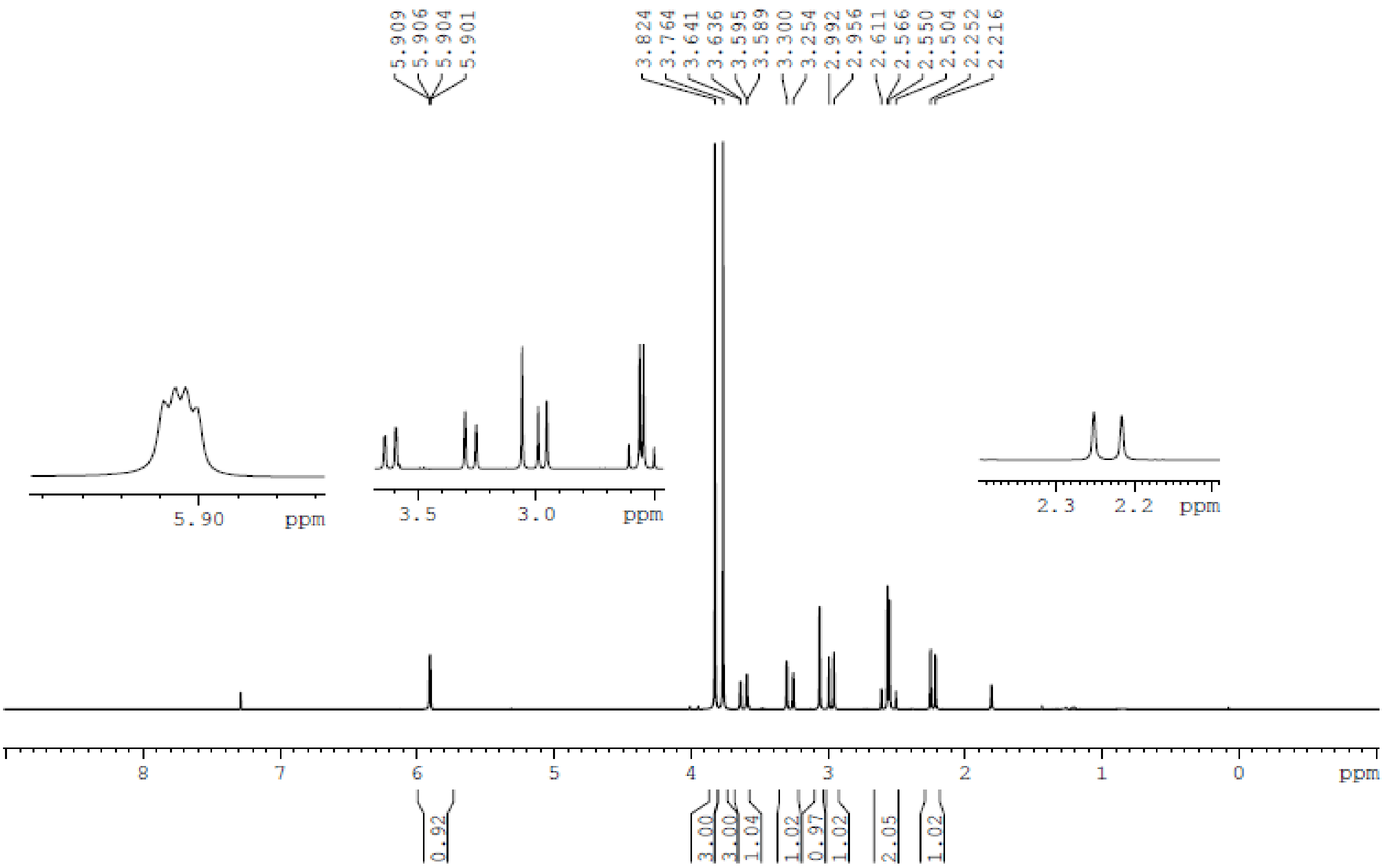

Compound **12a**.
100.6 MHz ^{13}C NMR spectrum
$CDCl_3$

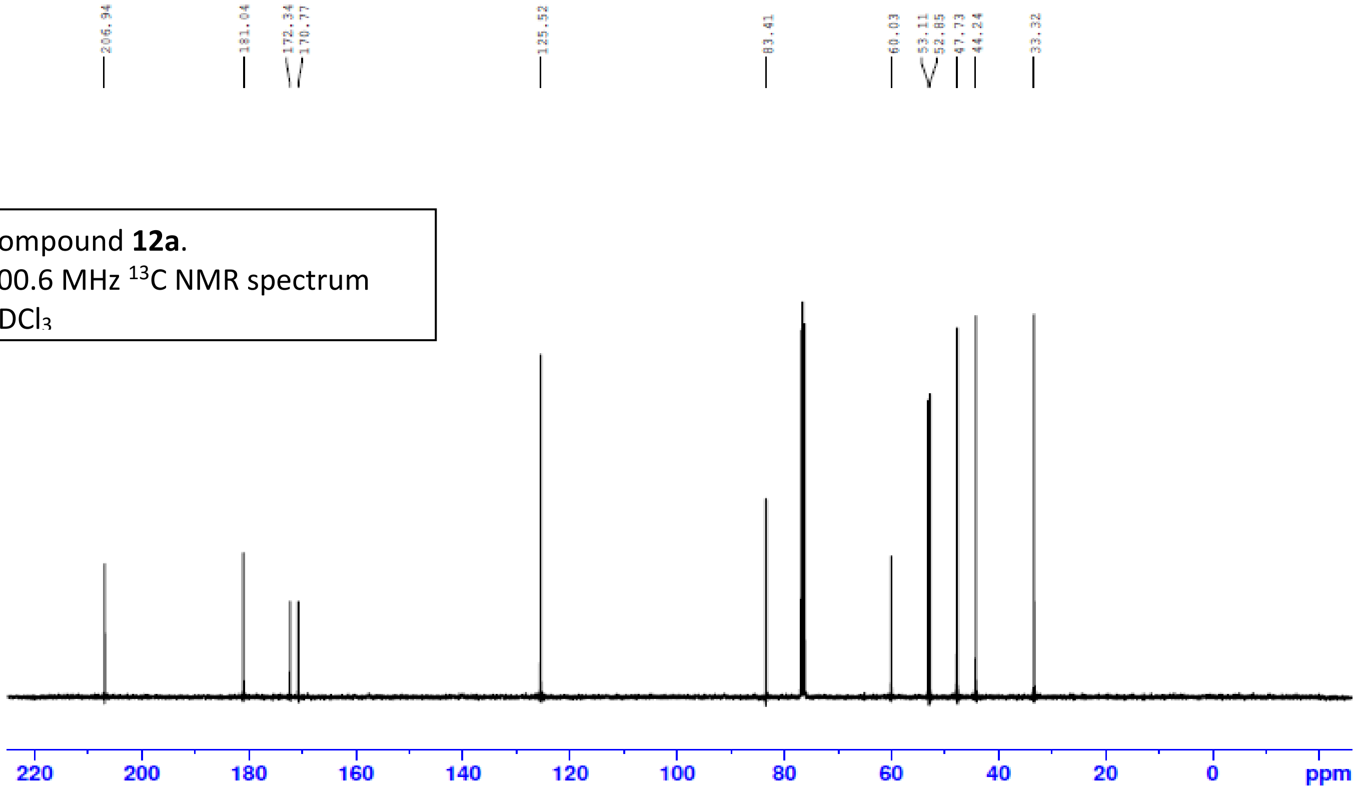

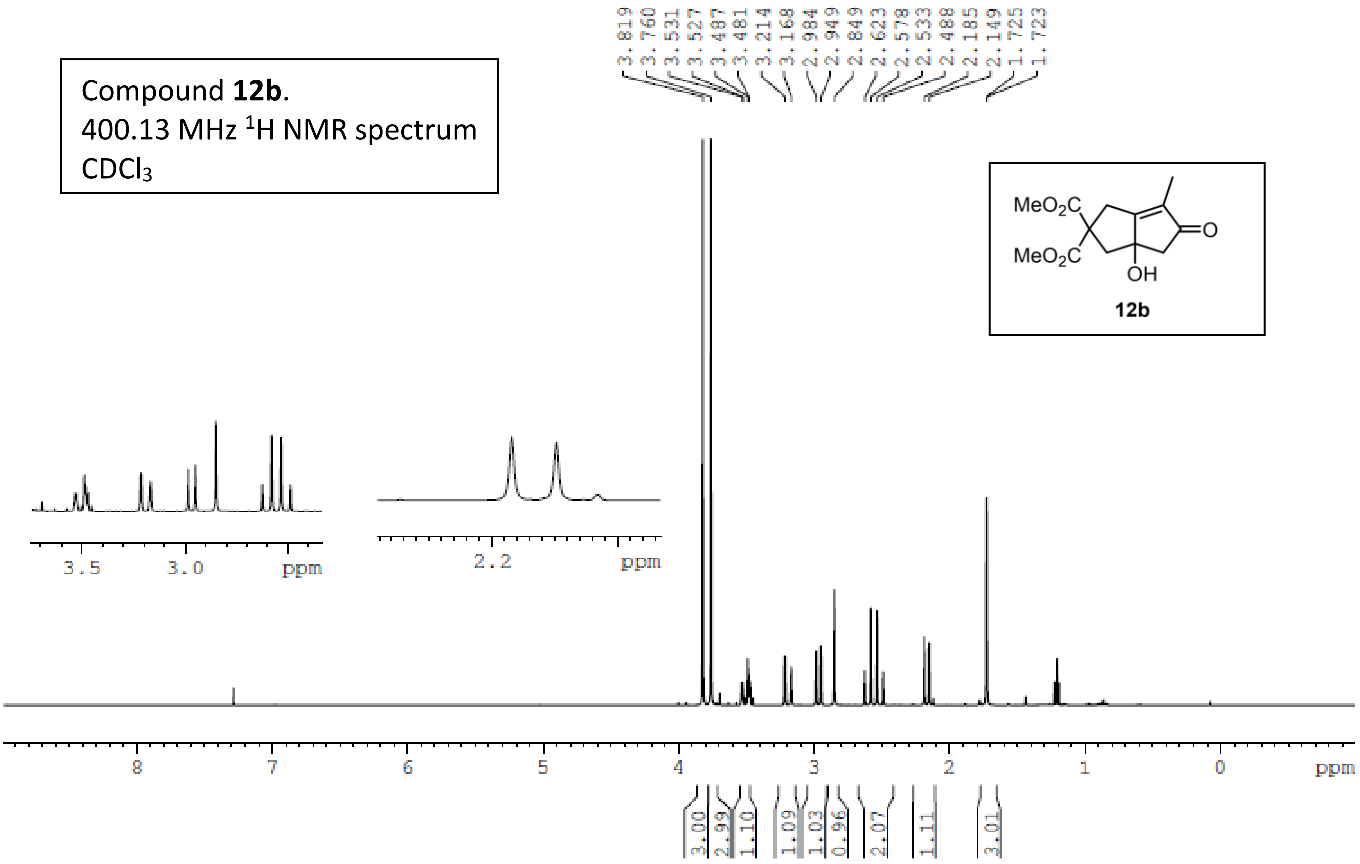

Compound **12b**.
400.13 MHz ^{1}H NMR spectrum
$CDCl_3$
3.819
3.760
3.531
3.527
3.487
3.481
3.214
3.168
2.984
2.949
2.849
2.623
2.578
2.533
2.488
2.185
2.149
1.725
1.723
MeO_2C
MeO_2C
O
OH
12b
3.5
3.0
ppm
2.2
ppm
8
7
6
5
4
3
2
1
0
ppm
3.00
2.99
1.10
1.09
1.03
0.96
2.07
1.11
3.01

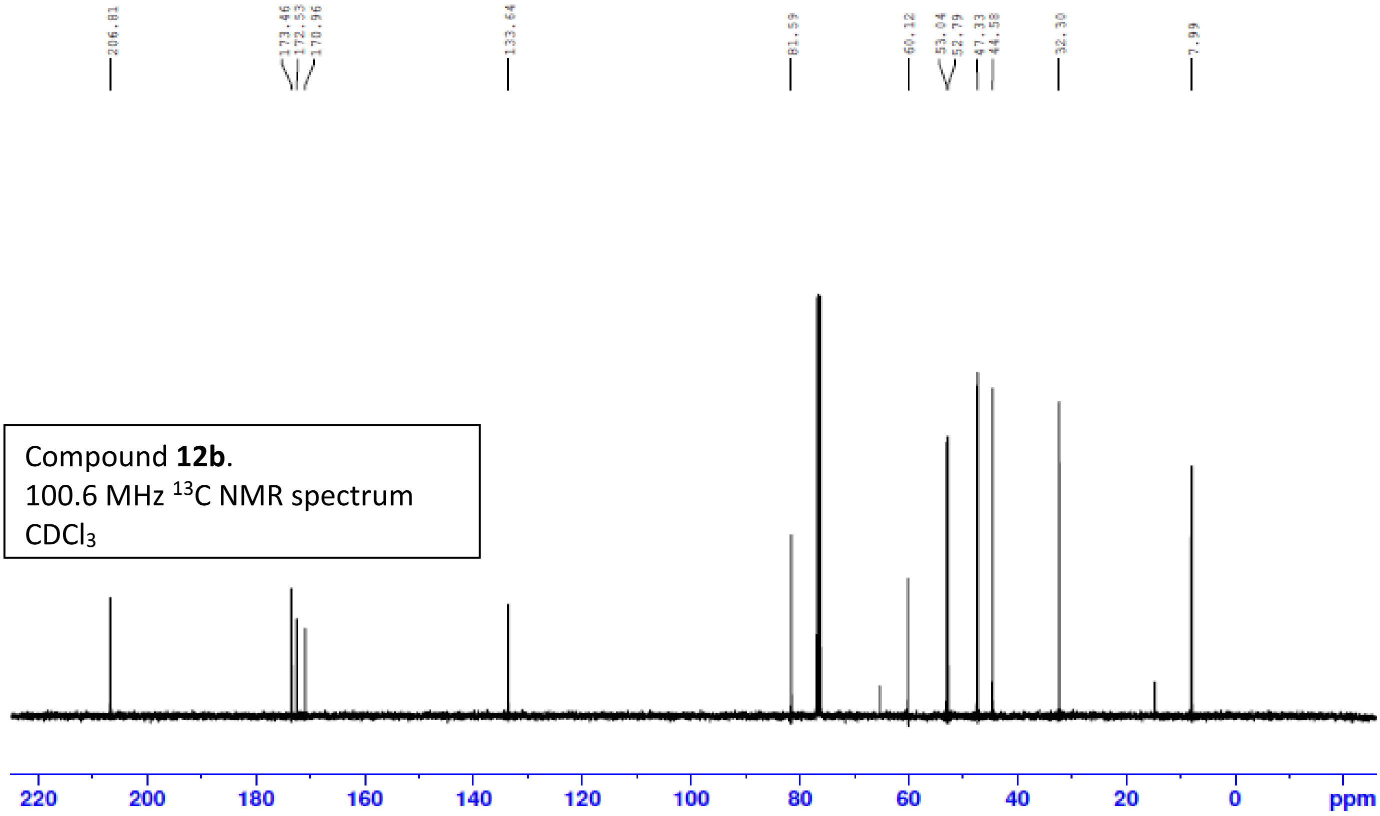

206.81
173.46
172.53
170.96
133.64
81.59
60.12
53.04
52.79
47.33
44.58
32.30
7.99
Compound **12b**.
100.6 MHz ^{13}C NMR spectrum
$CDCl_3$
220
200
180
160
140
120
100
80
60
40
20
0
ppm

Compound **12c**.
400.13 MHz ^{1}H NMR spectrum
$CDCl_3$

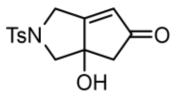

12c

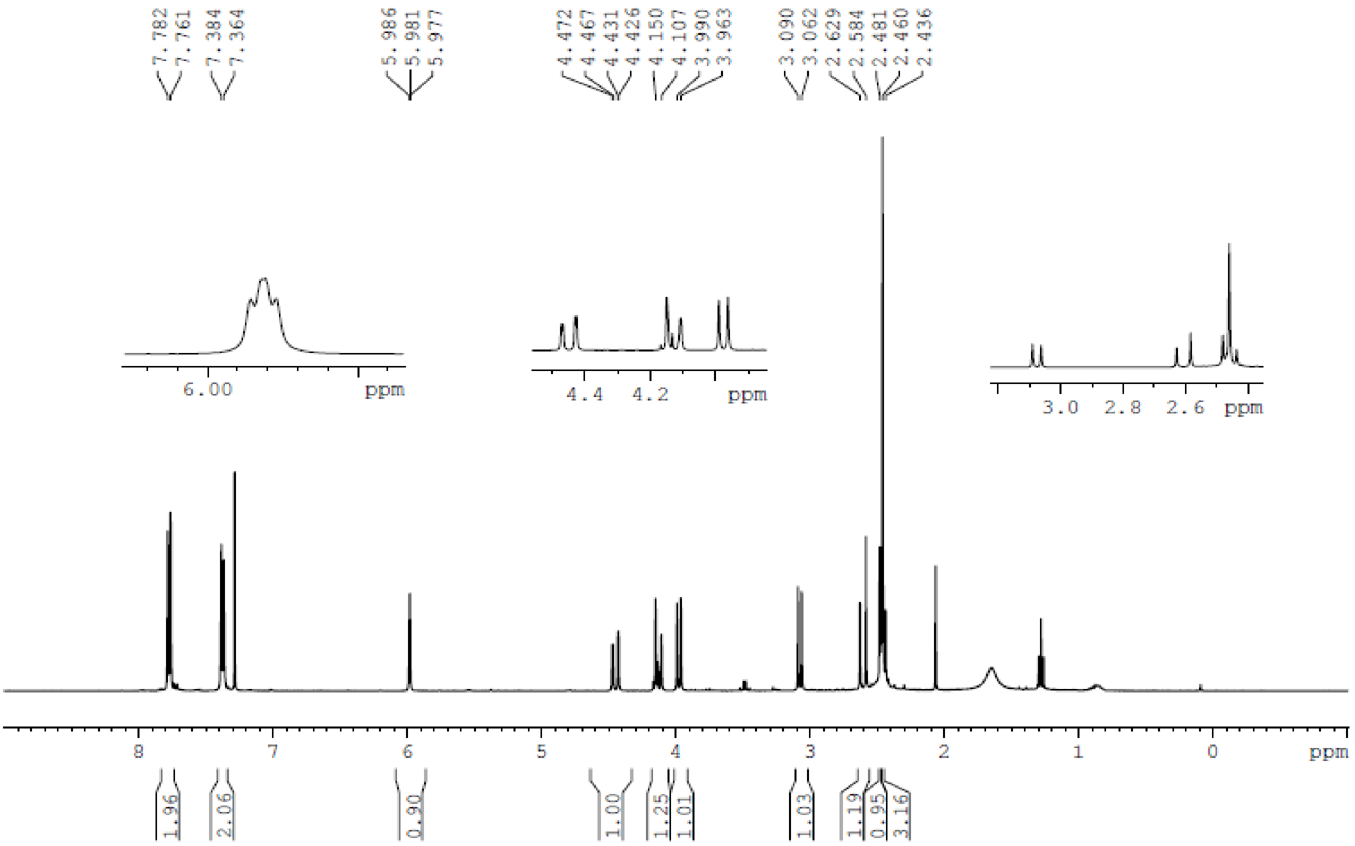

Compound **12c**.
100.6 MHz ^{13}C NMR spectrum
$CDCl_3$

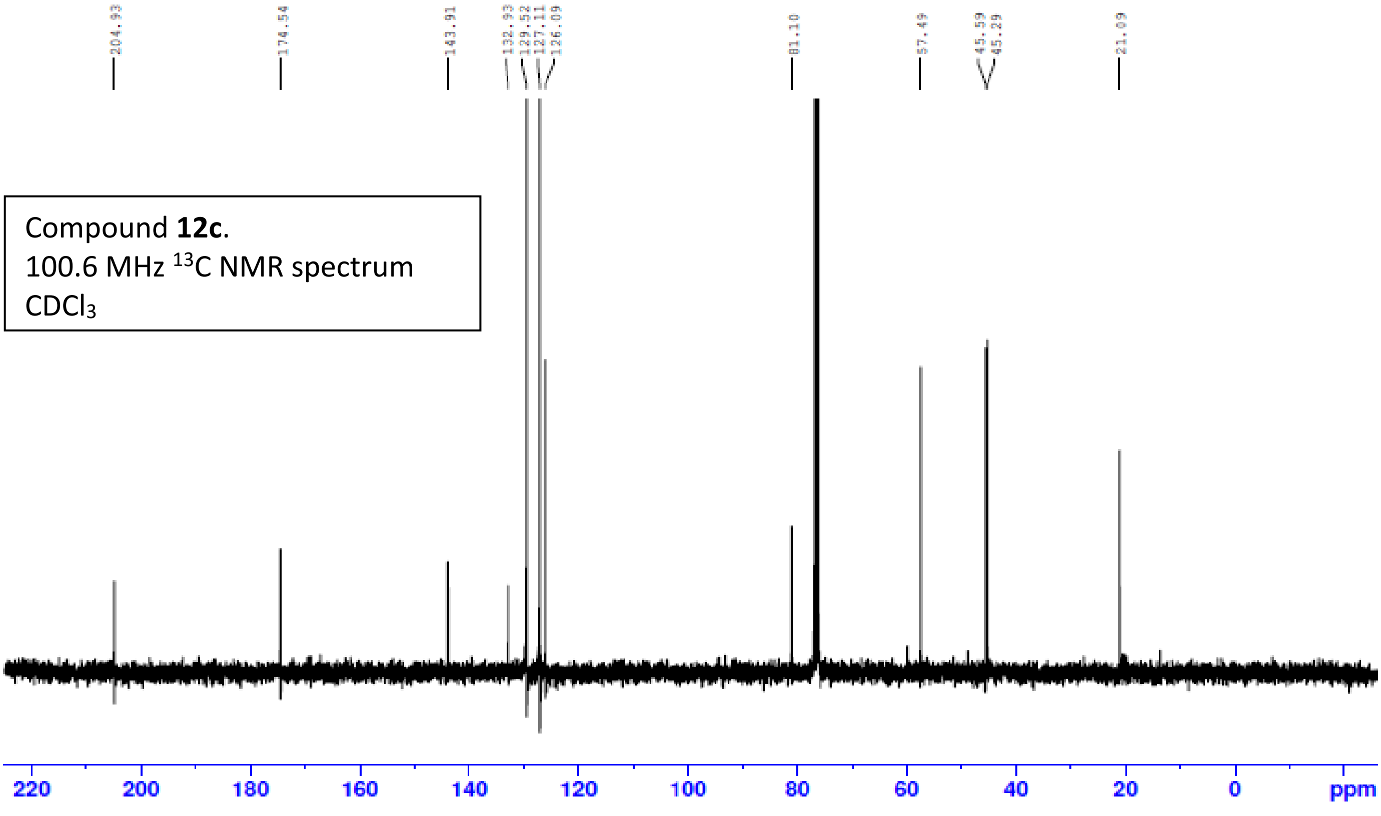

Compound **12d**.
400.13 MHz ^{1}H NMR spectrum
$CDCl_3$

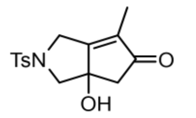

12d

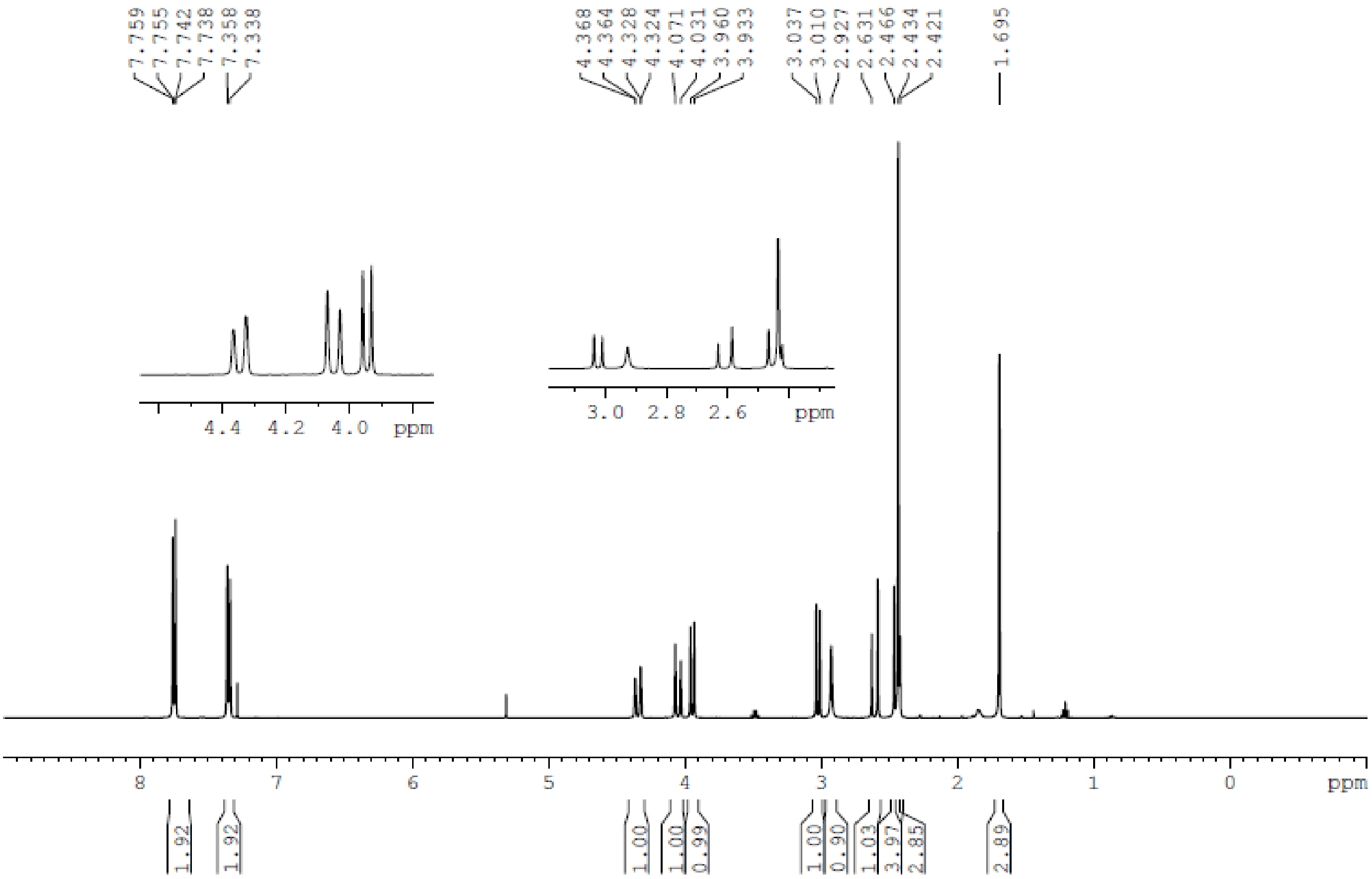

Compound **12d**.
100.6 MHz ^{13}C NMR spectrum

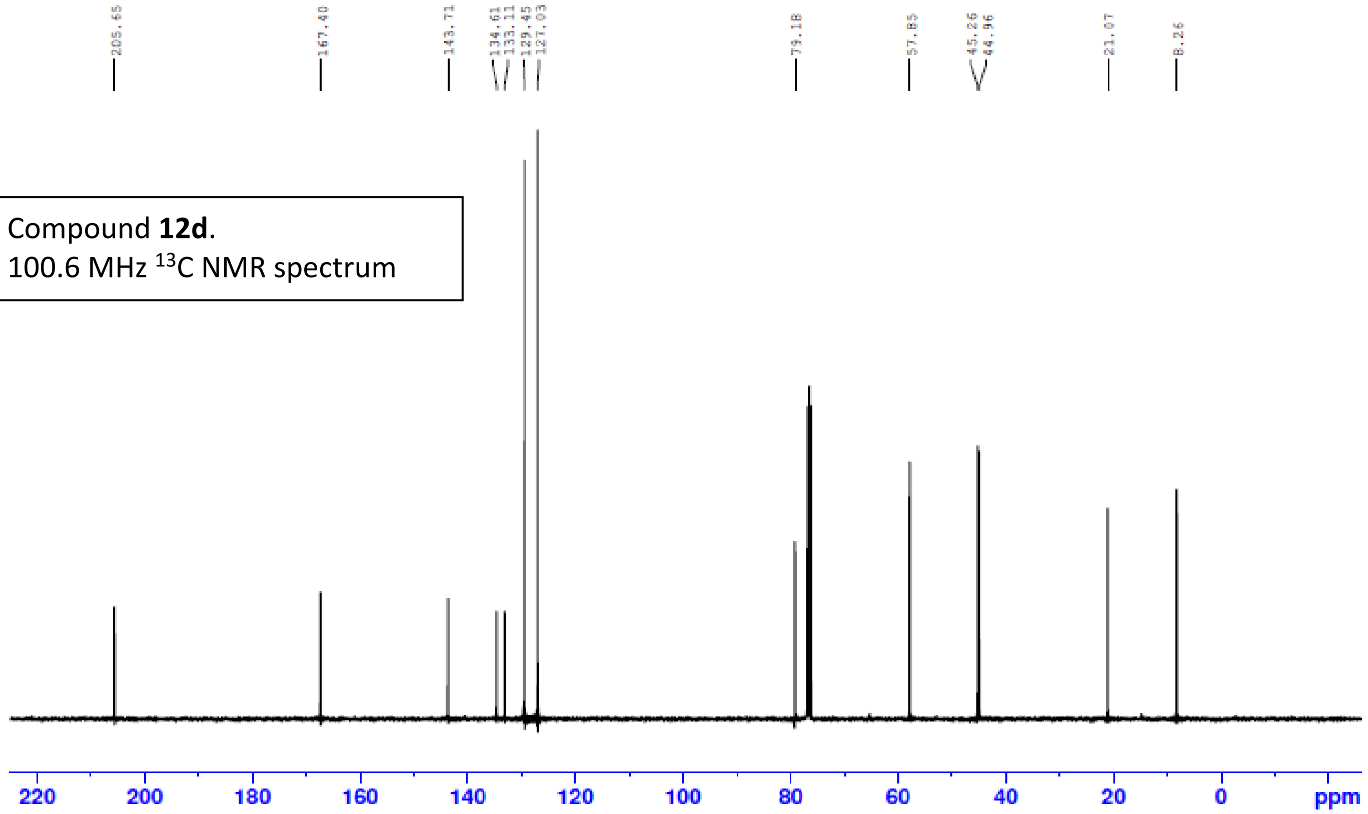

Compound **12e**. D281708 .
400.13 MHz ^{1}H NMR spectrum
$CDCl_3$

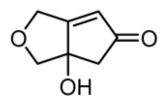

12e

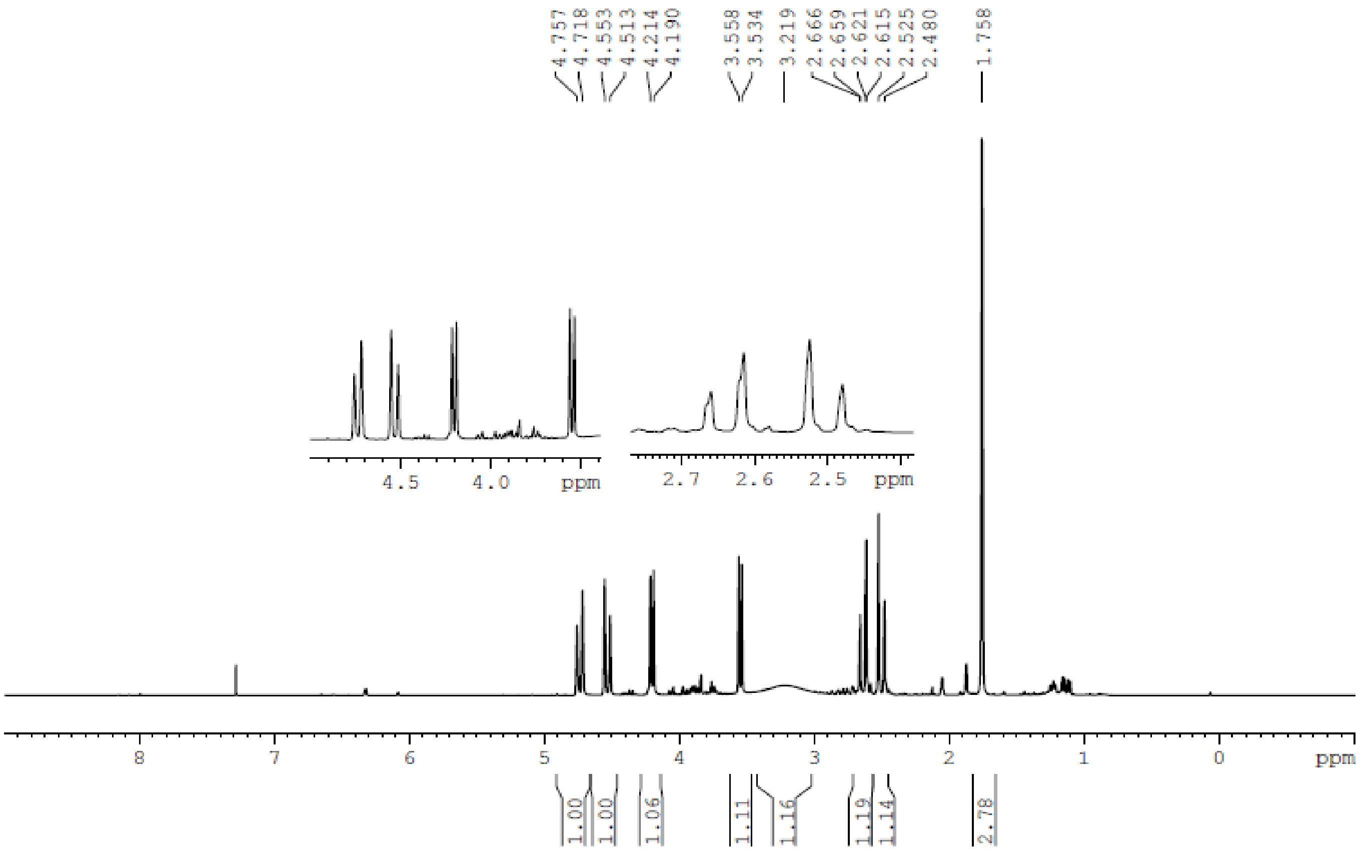

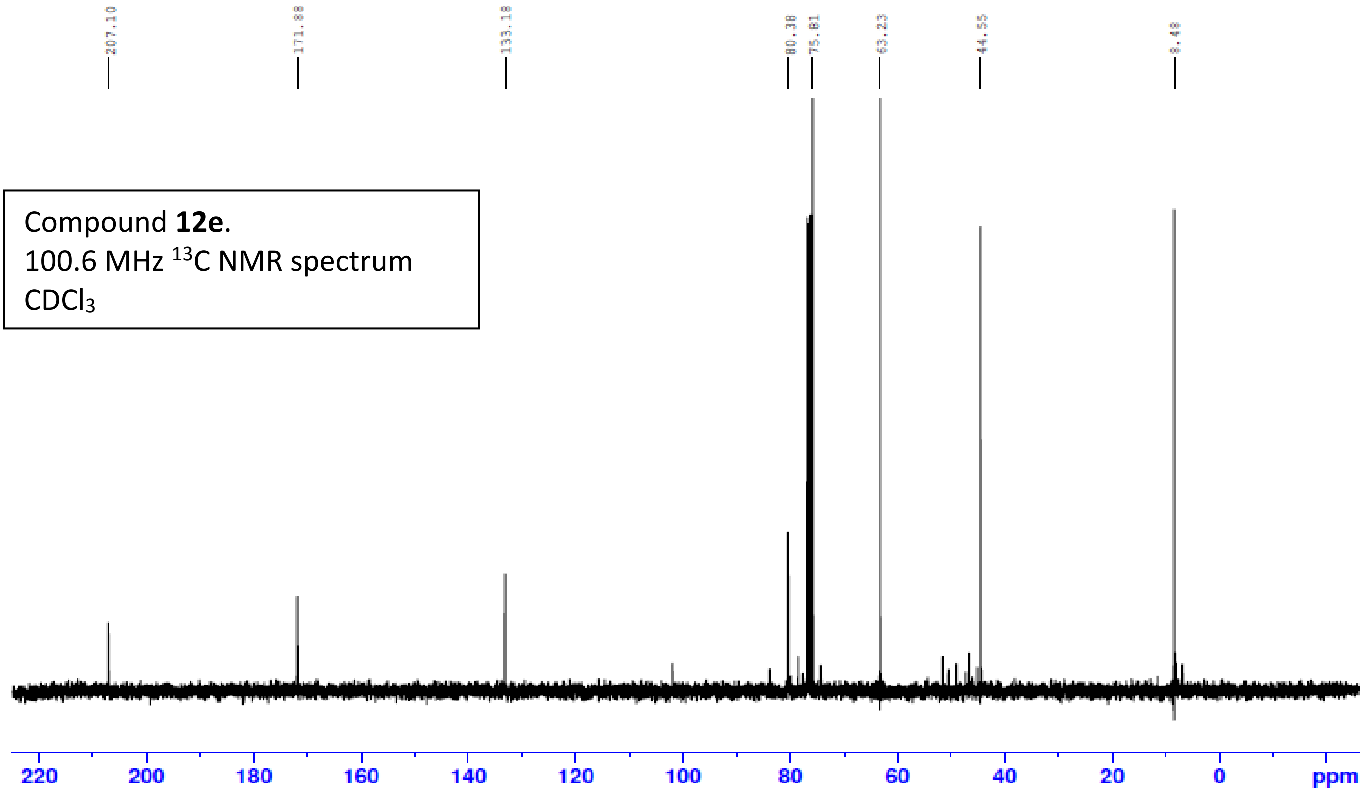

Compound **12e**.
100.6 MHz ^{13}C NMR spectrum
$CDCl_3$

Compound **13a**.
400.13 MHz ^{1}H NMR spectrum
$CDCl_3$

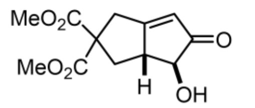

13a

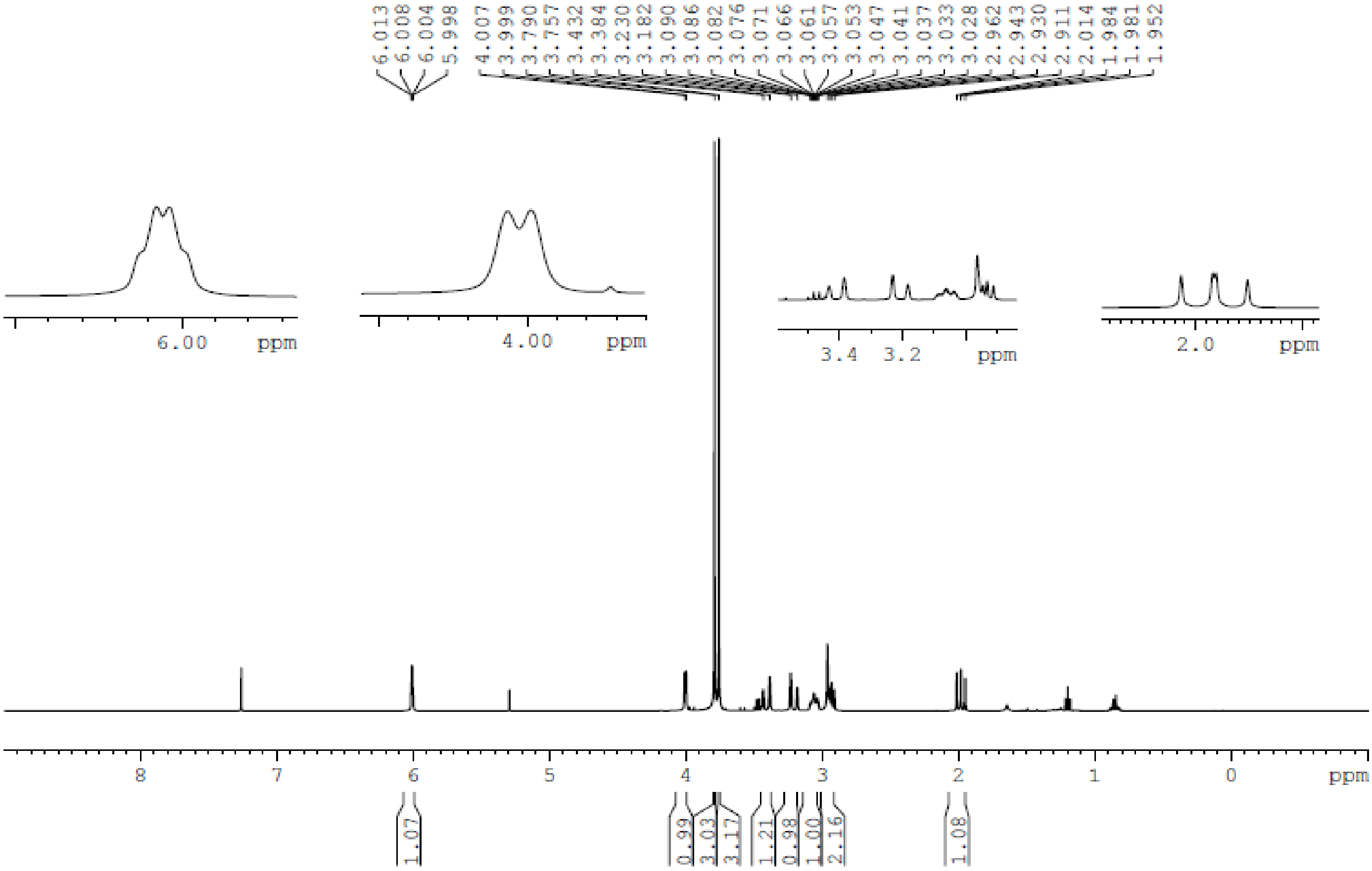

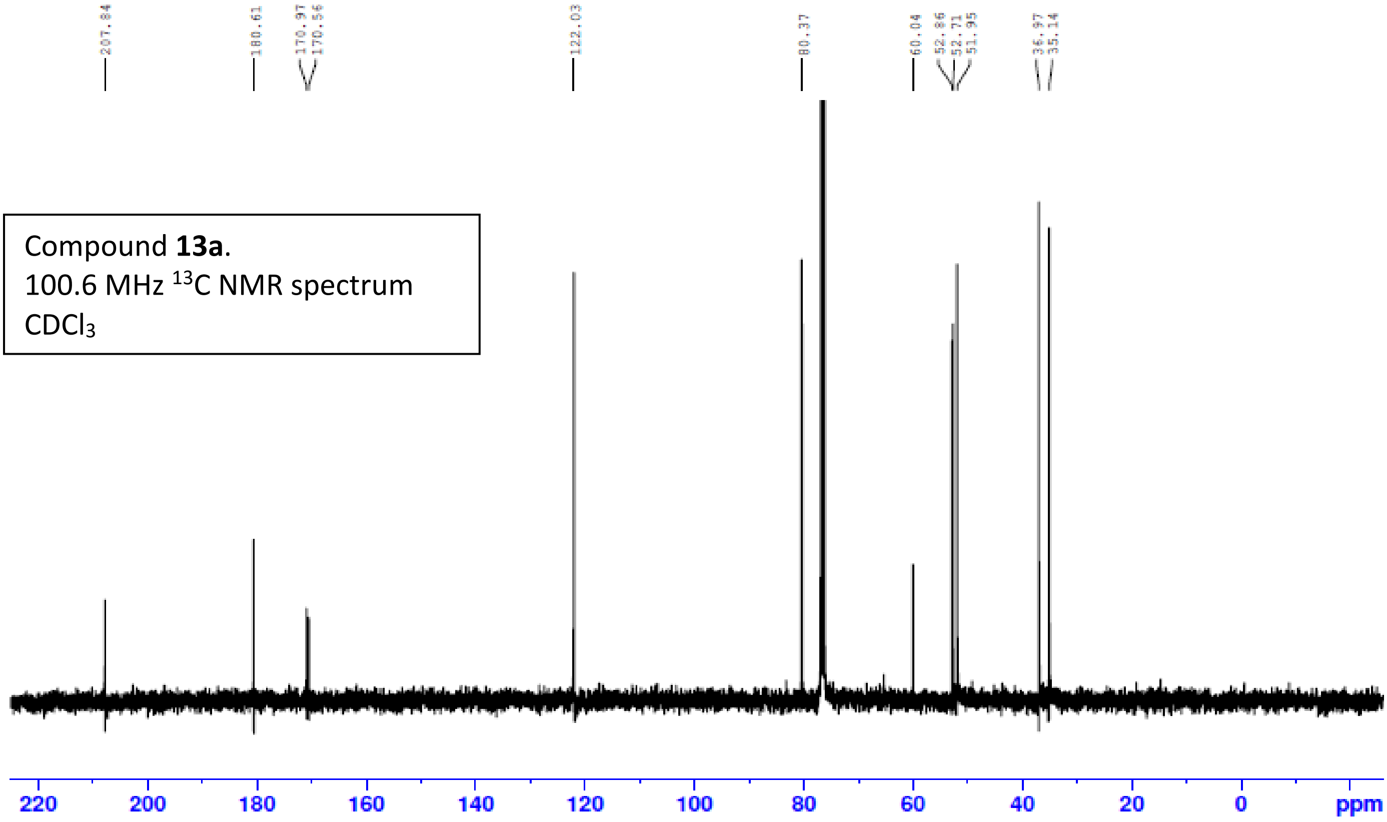

Compound **13a**.
100.6 MHz ^{13}C NMR spectrum
$CDCl_3$

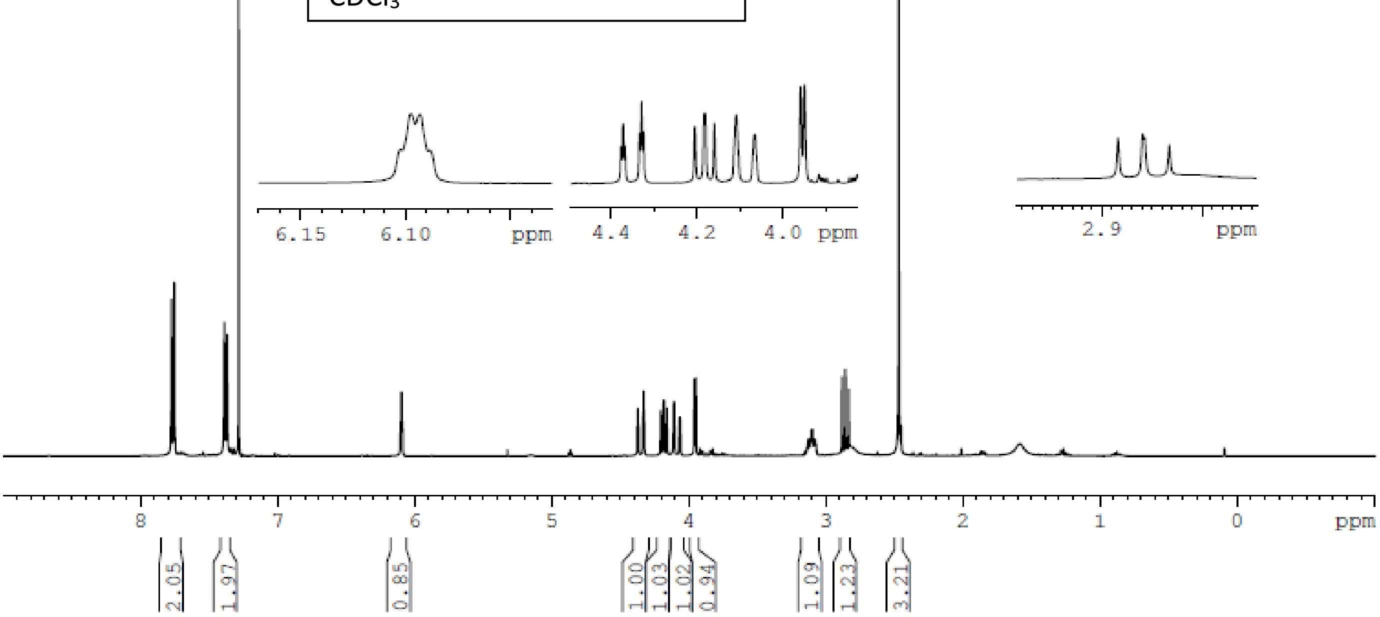
Compound 13b.
400.13 MHz 1H NMR spectrum
CDCl3
7.779 7.774 7.770 7.758 7.753 7.748 7.390 7.389 7.369 7.368 6.103 6.097 6.093 6.089 4.377 4.373 4.368 4.334 4.329 4.325 4.205 4.184 4.181 4.160 4.109 4.066 3.959 3.950 3.141 3.137 3.133 3.127 3.124 3.118 3.111 3.107 3.102 3.097 3.093 3.091 3.086 3.080 3.076 3.071 2.468
2.05 1.97 0.85 1.00 1.03 1.02 0.94 1.09 1.23 3.21
ppm

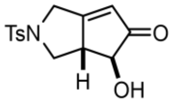
TsN
O
H
OH
13b

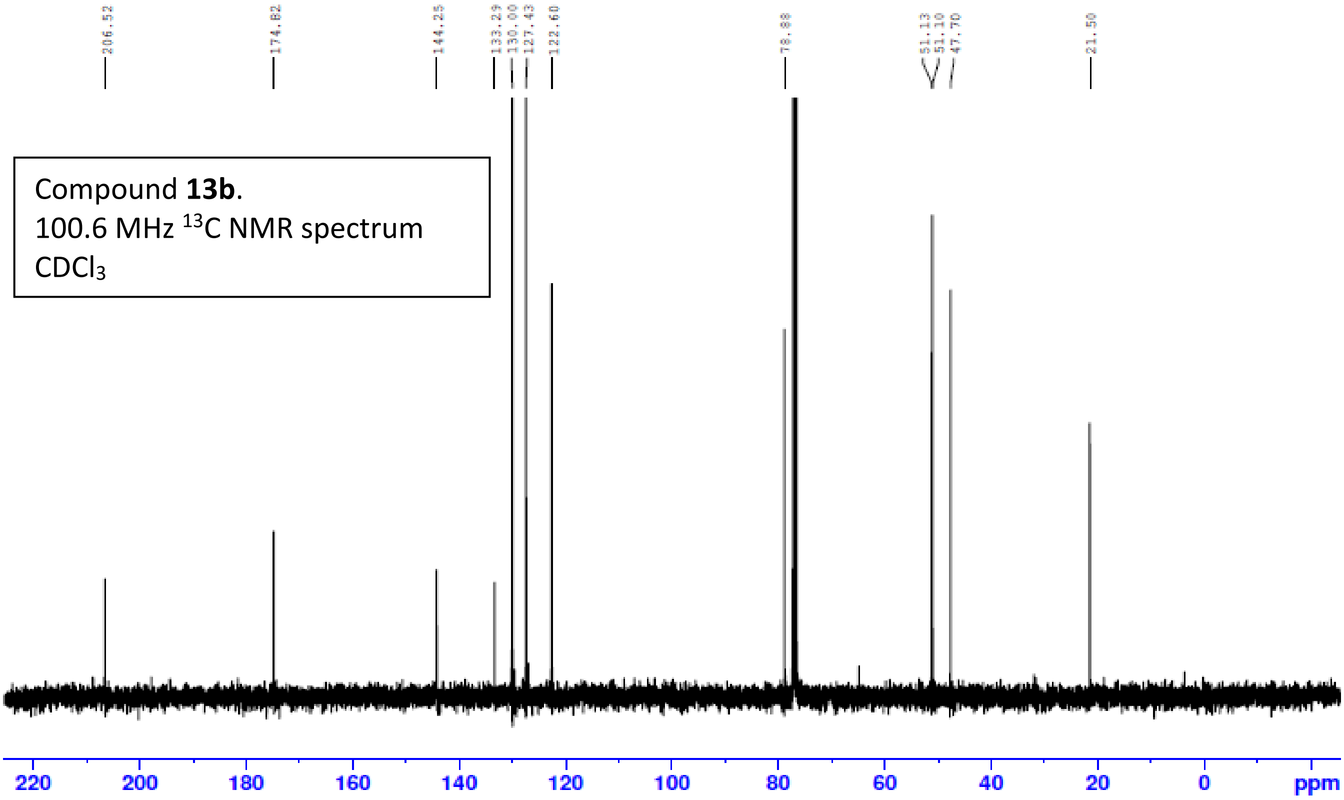
Compound 13b.
100.6 MHz 13C NMR spectrum
CDCl3
206.52 174.82 144.25 133.29 130.00 127.43 122.60 78.88 51.13 51.10 47.70 21.50
ppm

VII References

(1) Armarego, W. L. F.; Chai, C. L. L., Purification of Organic Chemicals, 5th ed. Elsevier Inc.: Oxford, 2009.

(2) Shintani, R.; Okamoto, K.; Otomaru, Y.; Ueyama, K.; Hayashi, T. Catalytic Asymmetric Arylative Cyclization of Alkynals: Phosphine-Free Rhodium/Diene Complexes as Efficient Catalysts. *J. Am. Chem. Soc.* **2005**, *127*, 54–55.

(3) Cambeiro, F.; López, S.; Varela, J. A.; Saá, C. Vinyl Dihydropyrans and Dihydrooxazines: Cyclizations of Catalytic Ruthenium Carbenes Derived from Alkynals and Alkynones. *Angew. Chem. Int. Ed.* **2014**, *53*, 5959–5963.

(4) Sperger, C.; Fiksdahl, A. Gold-Catalyzed Cyclizations of 1,6-Diynes. *Org. Lett.* **2009**, *11*, 2449–2452.

(5) Barrett, S.; O'Brien, P.; Steffens, H. C.; Towers, T. D.; Voith, M. New Route to 4-Aminocyclopent-2-en-1-ols: Synthesis and Enantioselective Rearrangement of 4-Amino-substituted Cyclopentene Oxides. *Tetrahedron* **2000**, *56*, 9633–9640.

(6) Muñoz-Bascón, J.; Hernández-Cervantes, C.; Padial, N. M.; Álvarez-Corral, M.; Rosales, A.; Rodriguez-Garcia, I.; Oltra, J. E. Ti-Catalyzed Straightforward Synthesis of Exocyclic Allenes. *Chem. Eur. J.* **2014**, *20*, 801–810.

(7) Ishizaki, M.; Hoshino, O. Unprecedented Cesium and Potassium Fluorides Catalyzed Trialkylsilylation and Tributylstannylation of Terminal Alkynes with Trifluoromethyl-Trialkylsilanes and -Tributylstannane. *Tetrahedron* **2000**, *56*, 8813–8819.

(8) Miyauchi, Y.; Noguchi, K.; Tanaka, K. Rhodium-Catalyzed One-Pot Intermolecular [2+2+2] Trimerization/Asymmetric Intramolecular [4+2] Cycloaddition of Two Aryl Ethynyl Ethers and 5-Alkynals. *Org. Lett.* **2012**, *14*, 5856–5859.

(9) Cambeiro, F.; López, S.; Varela, J. A.; Saá, C. Cyclization by Catalytic Ruthenium Carbene Insertion into C_{sp}^3-H Bonds. *Angew. Chem. Int. Ed.* **2012**, *51*, 723–727.

(10) Li, M.; Datta, S.; Barber, D. M.; Dixon, D. J. Dual Amine and Palladium Catalysis in Diastereo- and Enantioselective Allene Carbocyclization Reactions. *Org. Lett.* **2012**, *14*, 6350–6353.

(11) Cochet, T.; Bellosta, V.; Roche, D.; Ortholand, J.-Y.; Greiner, A.; Cossy, J. Rhodium(III)-catalyzed Allylic C–H Bond Amination. Synthesis of Cyclic Amines from ω-Unsaturated *N*-Sulfonylamines. *Chem. Commun.* **2012**, *48*, 10745-10747.

(12) Gibson, S. E.; Kaufmann, K. A. C.; Haycock, P. R.; White, A. J. P.; Hardick, D. J.; Tozer, M. J. Pendant Alkenes Promote Cobalt−Cobalt Bond Cleavage in (Alkyne)(binap)tetracarbonyldicobalt(0) Complexes. *Organometallics* **2007**, *26*, 1578–1580.

(13) Escalante, L.; González-Rodríguez, C.; Varela, J. A.; Saá, C. Tandem Brønsted Acid Promoted and Nazarov Carbocyclizations of Enyne Acetals to Hydroazulenones. *Angew. Chem. Int. Ed.* **2012**, *51*, 12316–12320.

(14) Shimamoto, T.; Chimori, M.; Sogawa, H.; Yamamoto, K. Cationic Palladium-Catalysed Hydrosilylative Cross-Coupling of Alkynes with Alkenes. *J. Am. Chem. Soc*. **2005**, *127*, 16410-16411.

(15) Coles, S. J.; Gale, P. A. Changing and Challenging Times for Service Crystallography. *Chem. Sci*. **2012**, *3*, 683-689.

(16) *CrysAlis PRO*. (Rigaku Oxford Diffraction, 2019).

(17) Sheldrick, G. M. Crystal Structure Refinement with SHELXL. *Acta Cryst*. **2015**, *C71*, 3-8.

(18) Farrugia, L. J. WingGX and ORTEP for Windows: An Update. *J. Appl. Cryst*. **2012**, *45*, 849-854.
